# Synthesis of Chiral Tetrahydro-3-benzazepine Motifs by Iridium-Catalyzed Asymmetric Hydrogenation of Cyclic Ene-carbamates

**DOI:** 10.1021/acs.orglett.2c00362

**Published:** 2022-03-03

**Authors:** Bram B. C. Peters, Pher G. Andersson, Somsak Ruchirawat, Winai Ieawsuwan

**Affiliations:** † Department of Organic Chemistry, Stockholm University, Svante Arrhenius väg 16C, SE-10691 Stockholm, Sweden; ‡ School of Chemistry and Physics, University of KwaZulu-Natal, Private Bag X54001, Durban 4000, South Africa; § Laboratory of Medicinal Chemistry, Chulabhorn Research Institute, 54 Kamphaeng Phet 6 Road, Bangkok 10210, Thailand; ∥ Center of Excellence on Environmental Health and Toxicology (EHT), Office of the Permanent Secretary (OPS), Ministry of Higher Education, Science, Research and Innovation (MHESI), Bangkok 10400, Thailand; ⊥ Program in Chemical Sciences, Chulabhorn Graduate Institute, Chulabhorn Royal Academy, 906 Kamphaeng Phet 6 Road, Bangkok 10210, Thailand

## Abstract

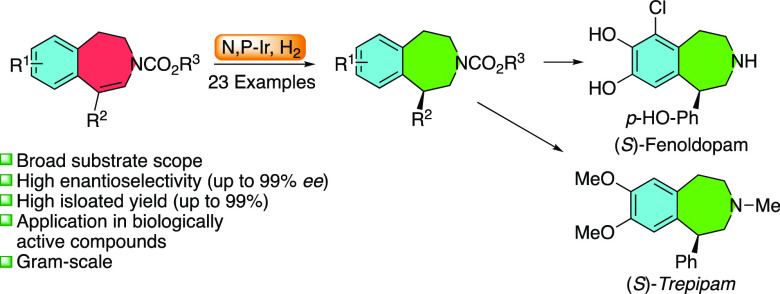

A highly efficient N,P-ligated iridium complex is presented for the simple preparation of chiral tetrahydro-3-benzazepine motifs by catalytic asymmetric hydrogenation. Substrates bearing both 1-aryl and 1-alkyl substituents were smoothly converted to the corresponding hydrogenated product with excellent enantioselectivity (91–99% *ee*) and in isolated yield (92–99%). The synthetic value of this transformation was demonstrated by a gram-scale hydrogenation and application in the syntheses of trepipam and fenoldopam.

Benzazepines represent common structural motifs in biologically active compounds. Widespread applications have been found in drug molecules, and various substituted tetrahydro-3-benzazepines have been evaluated pharmacologically in the past.^1^ Among these, several 1-substituted tetrahydro-3-benzazepines have tested positively as drug candidates against various diseases. For example, fenoldopam shows blood-pressure-reducing abilities,^2^ SCH-23390 is an excellent D_1_ receptor antagonist,^3^ and lorcaserin acts as an antiobesity drug (Figure 1).^4^

**Figure 1:**
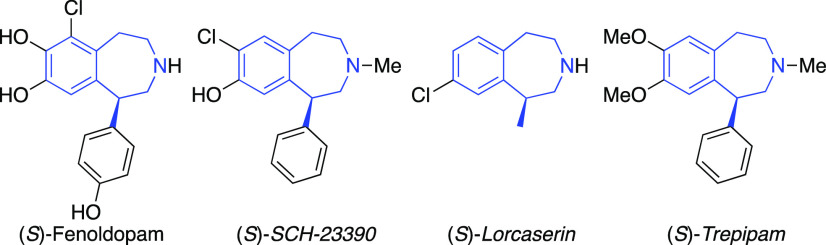
Representative chiral 1-substituted tetrahydro-3-benzazepine drugs.

Numerous racemic syntheses of 1-substituted tetrahydro-3-benzazepines have been developed, enabled mostly by intramolecular Friedel–Crafts-type alkylation,^5^ ring enlargement,^6^ reductive cyclization,^7^ or arylation.^8^ Despite their importance, fewer enantioselective methods have been developed to access enantioenriched products. The reported asymmetric methodologies mainly rely on a chiral pool approach,^9^ auxiliary strategy,^10^ or catalytic asymmetric synthesis.^11^ However, the catalytic asymmetric approaches that have been developed thus far do not focus on the synthesis of benzazepine motifs but rather show a single application of the obtained chiral products in the synthesis of a benzazepine. For example, the elegant contributions of Wu,^11a^ Riera,^11b^ and Chen and Zhang^11c^ can all yield chiral 1-substituted benzazepine motifs after several transformations but have only been demonstrated once (Scheme 1a–c).

**Scheme 1:**
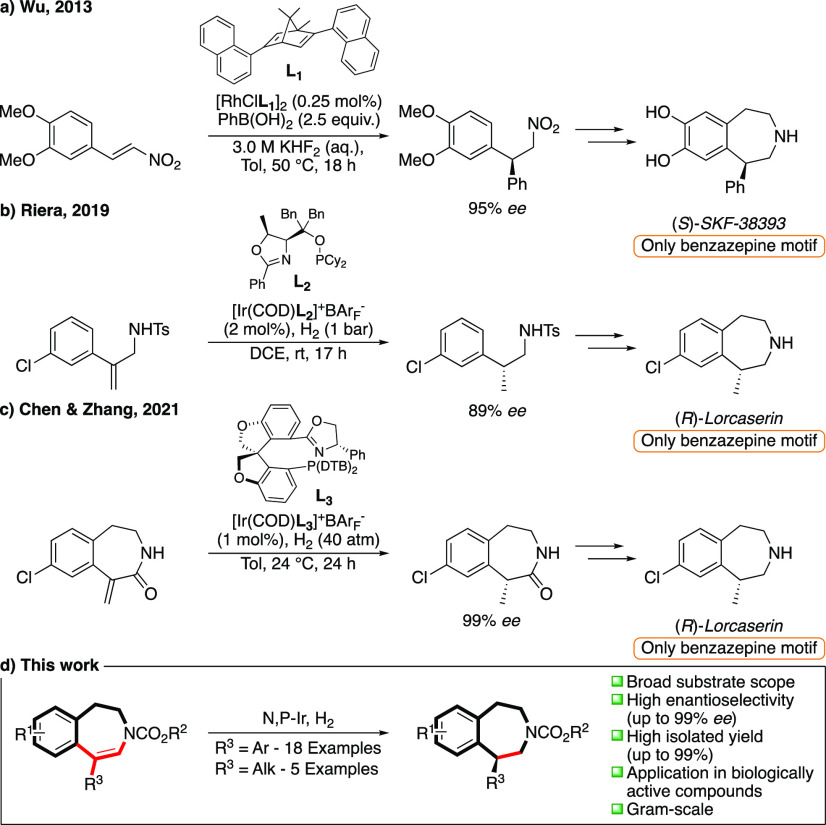
Representative Catalytic Approaches to Chiral 1-Substituted Tetrahydro-3-benzazepine Motifs and This Work

Over the past decades, asymmetric hydrogenation using hydrogen gas has proven to be one of the most efficient methods for installing chirality due to the high reactivity, enantioselectivity, and atom economy.^12^ The hydrogenation of cyclic ene-carbamate precursors, which can be prepared by a pinacol–pinacolone rearrangement, as outlined in Scheme 2,^13^ can potentially lead to the facile synthesis of valuable chiral 3-benzapine structures. Inspired by our previous success in the hydrogenation of cyclic motifs,^14^ we were encouraged to elaborate a novel asymmetric strategy for the preparation of chiral 3-benzazepines (Scheme 1d). In addition, the obtained methodology was applied in the synthesis of biologically relevant compounds.

**Scheme 2:**
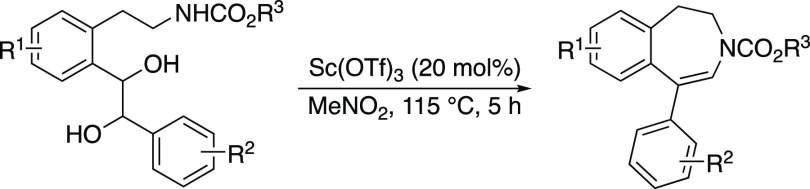
Synthesis of Cyclic Ene-carbamates

Initially, several structurally diverse chiral N,P-ligated iridium complexes were evaluated in the hydrogenation of model substrate **1a** (Table 1, entries 1–4). To our delight, catalyst **A** was shown to be very efficient and provided full and clean conversion toward the desired product **2a** with 99% *ee* when 1 mol % of catalyst was used in dichloromethane (DCM) under 100 bar of hydrogen atmosphere. Decreasing the catalyst loading or the hydrogen pressure negatively affected the conversion, whereas the high enantioselectivity was retained (entries 5 and 6).

**Table 1:**
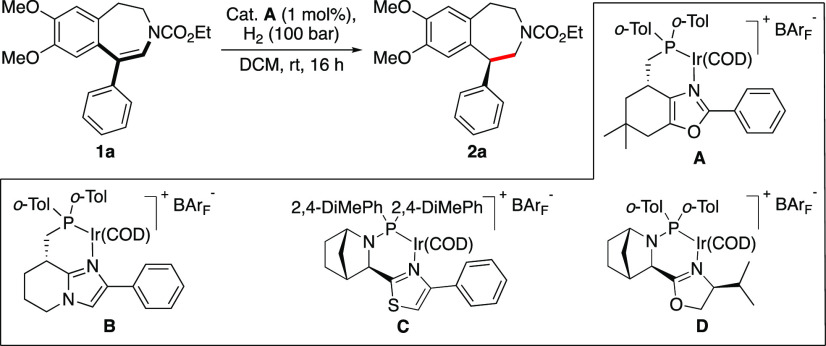
Optimization of Reaction Conditions^a^

entry	deviation from above	conv. (%)	*ee* (%)
1	no	full	99 (*S*)
2	Cat. **B** instead of Cat. **A**	80	80 (*S*)
3	Cat. **C** instead of Cat. **A**	full	97 (*R*)
4	Cat. **D** instead of Cat. **A**	71	33 (*R*)
5	0.5 mol % Cat. **4A** instead of 1 mol %	73	99 (*S*)
6	50 bar H_2_ instead of 100 bar	94	99 (*S*)

a Reactions were performed using 0.05 mmol **1a** in 1 mL of DCM. Conversion was determined using ^1^H NMR spectroscopy. The enantiomeric excess was determined by supercritical fluid chromatography (SFC) analysis using Chiralcel OJ-H chiral stationary phase. The stereochemistry was assigned by a comparison of the optical rotation with reported values after the reduction of **2a** by LiAlH_4_.

Having established an effective catalytic system, we began to investigate the generality of this iridium-catalyzed asymmetric hydrogenation of cyclic ene-carbamates (Scheme 3). Starting with electron-rich dimethoxy-substituted benzazepine motifs, both the model substrate **1a** and different para-substituted 1-aryl ene-carbamates (**1b**–**1d**) were hydrogenated with excellent enantioselectivity (96–99% *ee*) and in high isolated yield (>95%). Increasing the number of substituents did not give any change in stereoselectivity, and both phenol- and methoxy-derived benzazepines **2e** and **2f** were obtained in 99% *ee*. Changing the dimethoxy substituent pattern to a 1,3-benzodioxole motif was well tolerated, giving 95 and 96% *ee* for the hydrogenation of **1g** and **1h**, respectively. Decreasing the electron density on the benzazepine motif to monomethoxy did not affect the enantioselectity, and substrates **1i**–**1k** were hydrogenated smoothly. Further decreasing the electronic properties to a fluorine-substituted core motif slightly decreased the enantioselectivity to 94% *ee* (**2l**); however, introducing a methoxy group to the para position of the 1-aryl substituent enhanced the stereochemical outcome to 96% *ee* (**2m**). The size of the carbamate group had little effect on the reactivity or selectivity, and methyl-, ethyl-, and benzyl-ene-carbamates **1n**–**1p** were all hydrogenated with excellent enantioselectivities of 96–99% *ee*. Changing the ring size had a minor effect, and the eight-membered cyclic carbamate **2q** was obtained with 95% *ee*. Unfortunately, the hydrogenation of *N*-methyl enamine **2r** was found to inhibit the hydrogenation. The amine most likely forms a strong chelate with the catalyst, preventing hydrogenation from occurring. Alternatively, it might deprotonate the acidic iridium–dihydride complex.^15^

**Scheme 3:**
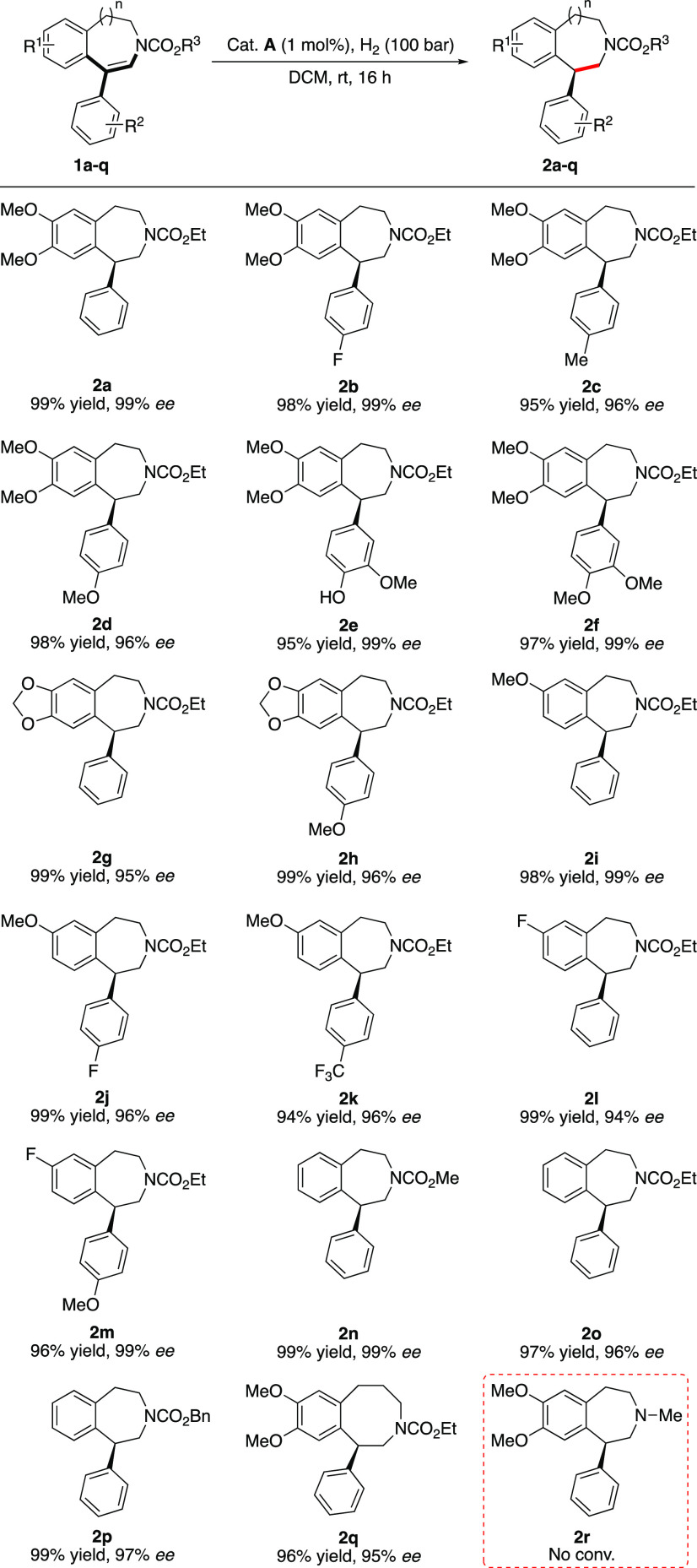
Asymmetric Hydrogenation of Aryl-Substituted Ene-carbamates Reaction conditions: 0.05 mmol substrate, 1 mol % **A**, 1 mL of DCM, 100 bar H_2_, 16 h, rt. The stereochemistry was tentatively assigned by assuming a similar hydrogenation pathway as that of **1a**, the absolute configuration of which was assigned by comparing the sign of the optical rotation with the literature value after the reduction of **2a** with LiAlH_4_. Isolated yields. Enantiomeric excess was determined by SFC analysis using chiral stationary phases.

We then further explored substrates having an alkyl substituent on the ene-carbamate to access 1-alkyl tetrahydro benzazepine scaffolds (Scheme 4).^16^ The methyl-substituted ene-carbamate **3a** was hydrogenated with 91% *ee*. Increasing the alkyl-chain length to *n*-butyl enhanced the enantioselectivity to 99% *ee* (**4b**). Both *i*-butyl- and *i*-propyl-substituted benzazepines were obtained with slightly decreased enantioselectivities of 94 and 93% *ee*, respectively (**4c** and **4d**). On the contrary, the benzyl-substituted benzazepine **4e** was accessed with an excellent enantioselectivity of 99% *ee*. Satisfactorily, all chiral alkyl-substituted benzazepines **4a**–**e** could be isolated in high yields.

**Scheme 4:**
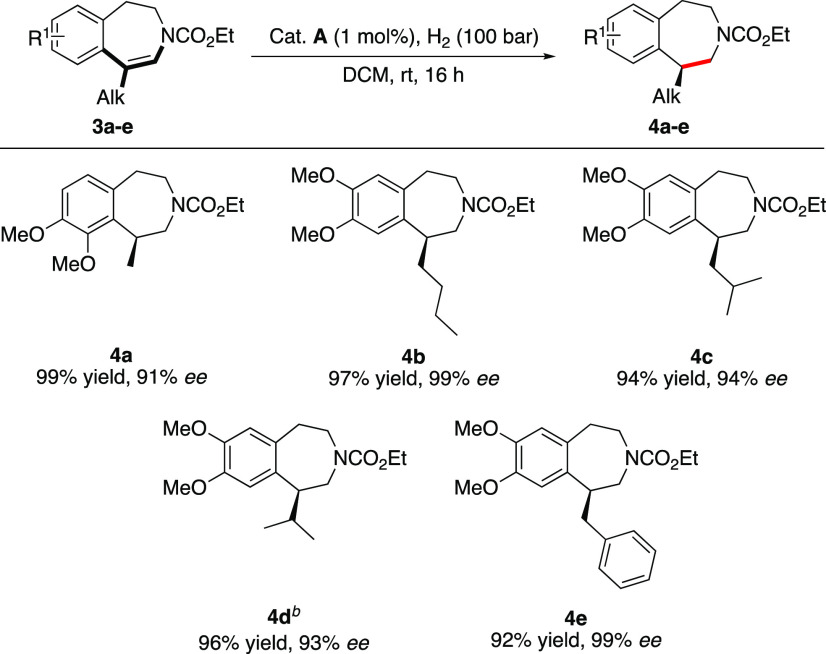
Asymmetric Hydrogenation of Alkyl-Substituted Ene-carbamates Reaction conditions: 0.05 mmol substrate, 1 mol % **A**, 1 mL of DCM, 100 bar H_2_, 16 h, rt, unless stated otherwise. The stereochemistry was tentatively assigned by assuming a similar hydrogenation pathway to **1a**, the absolute configuration of which was assigned by comparing the sign of the optical rotation with the literature value after the reduction of **2a** with LiAlH_4_. Isolated yields. Enantiomeric excess was determined by SFC analysis using chiral stationary phases. 2 mol % **A** was used.

To demonstrate the scalability of this asymmetric protocol, we carried out the gram-scale hydrogenation of ene-carbamate **1a** with the same reactivity and selectivity, and the desired chiral benzazepine **2a** was obtained in 98% yield with 99% *ee* (Scheme 5a). Treating the obtained hydrogenated product **2a** with an excess of LiAlH_4_ in MeOH reduced the carbamate group to methylamine to elaborate (*S*)-trepipam in 92% yield, exemplifying the synthetic utility of this asymmetric hydrogenation methodology. Further application was demonstrated by the synthesis of blood-pressure-reducing agent (*S*)-fenoldopam (Scheme 5b). The hydrogenation of **1s** proceeded smoothly, giving the corresponding tetrahydro-3-benzazepine **2s** with 99% *ee*. Subsequent hydrogenation of the isolated product in the presence of Pd/C led to the cleavage of the Cbz-group. Thereafter, **2t** could be transformed to (*S*)-fenoldopam, as previously described.^2b^ To the best of our knowledge, no asymmetric synthesis of fenoldopam was previously disclosed.^2b,17^

**Scheme 5:**
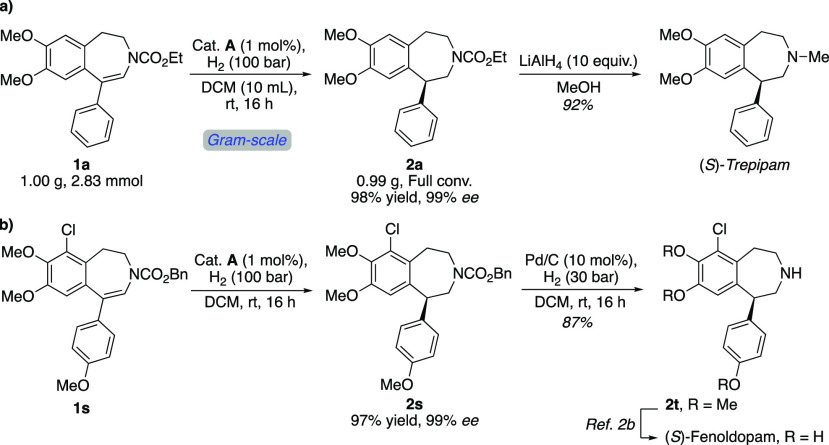
Gram-Scale Asymmetric Hydrogenation and Applications

Because the absolute configuration of trepipam is reported, we were able to confirm the stereochemical outcome of the hydrogenation by comparing the sign of optical rotation of our synthetic trepipam with that reported. This confirmed the absolute configuration of product **2a** to be the (S)-enantiomer. On the basis of computational and experimental studies, a quadrant model has been developed to predict the stereochemical outcome in the iridium-catalyzed asymmetric hydrogenation of olefins using bidentate N,P-ligands.^18^ It is suggested that olefins preferentially coordinate trans to phosphorus and that steric interactions between the ligand and the olefin are the origin of the enantioselection (Figure 2a). As a consequence of the encumbered chiral ligand around the iridium center, the coordinated olefin experiences steric hindrance from either the lower or the upper left quadrant. To minimize steric interactions, the smallest hydrogen substituent of the olefin arranges itself to point toward the bulk of the ligand, which in this case occupies the lower left quadrant iii. Thereby, the coordinated enantiotopic face is locked. The quadrant model, where the hydride is delivered from the bottom, then predicts the enantiomerical outcome of the hydrogenation (Figure 2b). Because the absolute configuration of **2a** was confirmed to be the (S)-enantiomer, we were able to validate the developed quadrant model that indeed predicted the stereochemical outcome for the hydrogenation of this class of cyclic ene-carbamates correctly (Figure 2c).

**Figure 2:**
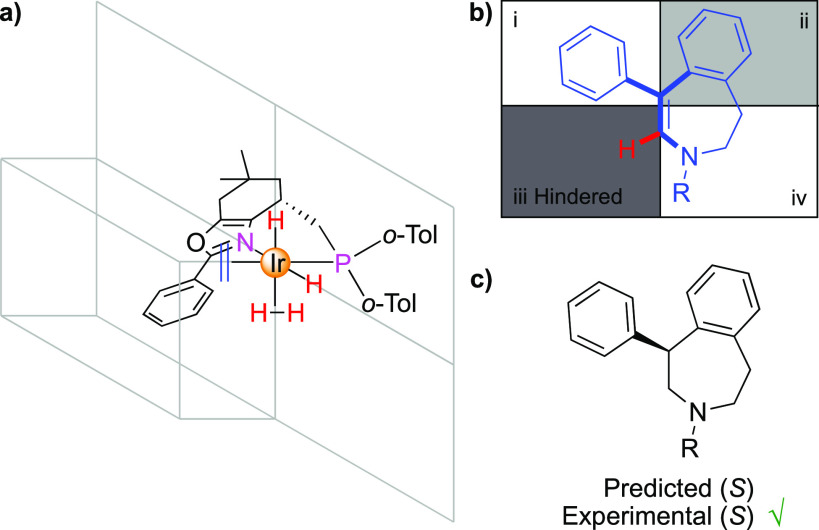
Stereoselectivity model. (a) Coordination of oxazole ligand and olefin to the iridium center. (b) Quadrant model based on the steric influence of the ligand seen from the olefin. (c) Predicted and experimental stereochemical outcomes for the hydrogenation of ene-carbamates.

In summary, we herein described the straightforward and operationally simple synthesis of chiral 3-benzazepines by the iridium-catalyzed asymmetric hydrogenation of cyclic ene-carbamates. A series of 1-aryl- and 1-alkyl-substituted benzazepines were accessed with excellent enantioselectivity (91–99% *ee*) and in high isolated yield (92–99%). The methodology was shown to be scalable to at least a gram scale. Furthermore, the synthetic utility was highlighted in the enantioselective preparation of trepipam and fenoldopam.

## Supporting Information Available

Experimental procedures, characterization data of new compounds, separation of chiral products, and NMR spectra of new compounds (PDF)
