# Supplementary material for: Synthesis of Chiral Tetrahydro-3-benzazepine Motifs by Iridium-Catalyzed Asymmetric Hydrogenation of Cyclic Ene-carbamates
Source: Org Lett. 2022 Mar 3;24(10):1969–73. doi: 10.1021/acs.orglett.2c00362 (PMC8938950; doi:10.1021/acs.orglett.2c00362)
Supplement: Supplementary file 1 — ol2c00362_si_001.pdf [file ol2c00362_si_001.pdf]

SUPPORTING INFORMATION

Synthesis of Chiral Tetrahydro-3-benzazepine Motifs by Iridium-Catalyzed Asymmetric Hydrogenation of Cyclic Ene-carbamates

Bram B. C. Peters,[a] Pher G. Andersson, *[a,b] Somsak Ruchirawat[c,d,e] and Winai Ieawsuwan*[c,d]

* Pher G. Andersson, E-mail: Pher.Andersson@su.se
* Winai Ieawsuwan, E-mail: Winai@cri.or.th

[a] Department of Organic Chemistry, Stockholm University, Svante Arrhenius väg 16C, SE-10691 Stockholm, Sweden.

[b] School of Chemistry and Physics, University of KwaZulu-Natal, Private Bag X54001, Durban, 4000, South Africa.

[c] Laboratory of Medicinal Chemistry, Chulabhorn Research Institute, 54 Kamphaeng Phet 6 Road, Bangkok, 10210, Thailand.

[d] Center of Excellence on Environmental Health and Toxicology (EHT), Office of the Permanent Secretary (OPS), Ministry of Higher Education , Science, Research and Innovation (MHESI), Bangkok, 10400, Thailand.

[e] Program in Chemical Sciences, Chulabhorn Graduate Institute, Chulabhorn Royal Academy, 906 Kamphaeng Phet 6 Road, Bangkok, 10210, Thailand.

Table of contents

General information………………………………………………………………….S-3

Experimental details and characterization data of new compounds…………………………S-4

Synthesis of ene-carbamates **1a-r**…………………………………………………S-4

Synthesis of ene-carbamate **1s** ………………………………………………………S-4

Synthesis of ene-carbamate **3a**…………………………………………………………S-6

Synthesis of ene-carbamates **3b-e** ……………………………………………………...S-8

General procedure for the asymmetric hydrogenation……………………………...S-11

Procedure for the gram-scale asymmetric hydrogenation…………………………..S-11

Characterization of hydrogenated products……………………………..........S-12

Synthesis of benzazepine **2t**………………………………………………………...S-19

NMR spectra – Ene-carbamates and intermediates………………………….…………...S-20

NMR spectra – Hydrogenated products……………………..…………………………...S-30

Separation of chiral products…………………………….………………..…………..S-59

Chromatograms……………………………………………………….…………………S-62

References……………………………………………………………………………….S-70

General information

All reaction vessels were dried in a vacuum oven (160 °C) and cooled down to room temperature under a flow of nitrogen prior to use. Dichloromethane was dried over calcium hydride and freshly distilled under nitrogen. THF was distilled from sodium-benzophenone under nitrogen. The commercially available chemicals were used directly or purified by either distillation or column chromatography. Chromatographic separations were performed on Kiesel gel 60 H silica gel [particle size: 0.063-0.200 mm (70-230 mesh ASTM) or 0.040-0.063 mm (230-400 mesh ASTM)]. Thin-layer chromatography (TLC) was performed on aluminum plates coated with Kiesel gel 60 (0.20 mm, UV 254 nm) and visualized under ultraviolet light followed by staining with potassium permanganate or phosphomolybdic acid. ^{1}H NMR spectra were recorded at 300, 400 or 500 MHz in $CDCl_3$ or DMSO-d_6 and referenced internally to the residual $CHCl_3$ (7.26 ppm) or DMSO (2.50 ppm) signal. ^{13}C NMR spectra were recorded at 75, 100 or 125 MHz in $CDCl_3$ and referenced to the central peak of $CHCl_3$ (77.16 ppm). ^{19}F NMR spectra were recorded at 376 MHz in $CDCl_3$ or CD_3OD. Chemical shifts were reported in ppm (δ scale), and coupling constants (J) were reported in Hertz (Hz). High resolution mass spectrometric (HRMS) data were obtained from a Bruker microTOF-Q II instrument operated at ambient temperatures. Melting points were determined on a Stuart SMP30 melting point apparatus and reported without correction. Optical rotation was recorded on a thermostated polarimeter using a sodium lamp (589 nm) and a 10 cm cell. Enantiomeric excesses were determined using SFC-DIAD (250 mm Chiralcel or Chiralpak columns, CO_2/MeOH) using chiral stationary phases. Racemic compounds were in all cases used for comparison.

Experimental details and characterization data of new compounds

Synthesis of ene-carbamates 1a-r

Ene-carbamates **1a-r** were prepared according to reported procedures and spectroscopic data was in agreement with the values reported therein.[1]

Synthesis of ene-carbamate 1s

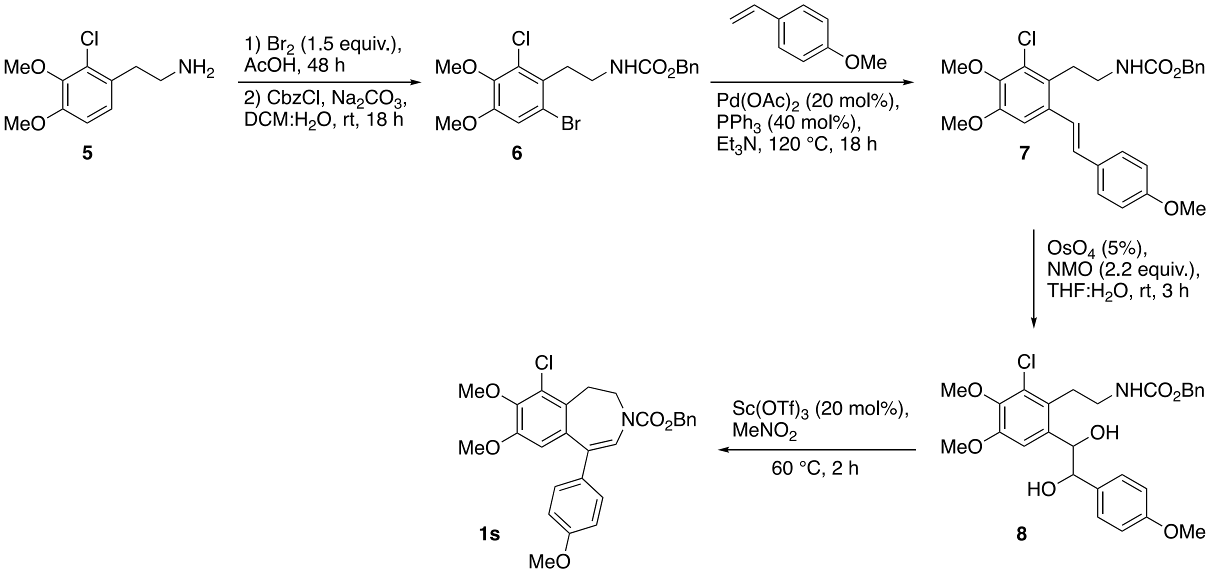

Benzyl (6-bromo-2-chloro-3,4-dimethoxyphenethyl)carbamate (6)

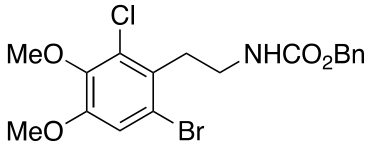

6

To a solution of amine **5** (2.75 g, 12.75 mmol, 1.0 equiv.) in glacial acetic acid (10 mL) was added Br_2 (3.06 g, 19.12 mmol, 1.5 equiv.) dropwise at room temperature and the reaction mixture was stirred for 48 h. The excess of glacial acetic acid and bromine were removed under reduced pressure. To a mixture of the obtained crude product and Na_2CO_3 (5.40 g, 50.99 mmol, 4.0 equiv.) in CH_2Cl_2 (30 mL) and H_2O (30 mL) was slowly added benzyl chloroformate (2.83 g, 16.57 mmol, 1.3 equiv.) at room temperature. After the reaction was stirred for 18 h, the resulting mixture was extracted with CH_2Cl_2 (3x30 mL). The combined organic layers were washed with 10% $NaHSO_3$ (30 mL), washed with brine (30 mL), dried over Na_2SO_4 and concentrated in *vacuo*. The crude product was purified by column chromatography (hexane/EtOAc, 90:10 to 83:17) to give carbamate **6** (3.63 g, 8.42 mmol, 66% yield over 2 steps). **Appearance:** White solid. **^{1}H NMR** (300 MHz, $CDCl_3$) δ: 7.41 - 7.24 (m, 5H), 7.02 (s, 1H), 5.09 (s, 2H), 4.99 (br.t, *J* = 5.5 Hz, 1H), 3.82 (2s, 6H), 3.42 (q, *J* = 6.6 Hz, 2H), 3.13 (t, *J* = 7.0 Hz, 2H) ppm. **^{13}C NMR** (75 MHz, $CDCl_3$) δ: 156.4, 152.5, 145.2, 136.7, 129.9, 128.8, 128.5, 128.2, 128.1, 119.3, 115.3, 66.6, 60.6, 56.3, 39.7, 34.0 ppm. **HRMS-ESI:** Found $[M+Na]^+$ = 450.0088, 452.0068, 454.0039; $C_{18}H_{19}BrClNO_4Na$ requires 450.0078, 452.0057, 454.0037. **m.p.** 99-100 °C.

Benzyl (*E*)-(2-chloro-3,4-dimethoxy-6-(4-methoxystyryl)phenethyl)carbamate (7)

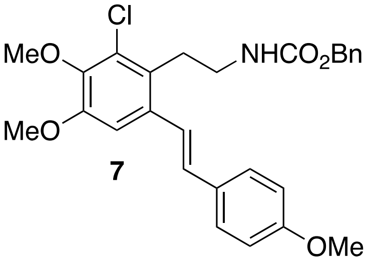

7

A 100 mL ace pressure tube was charged with carbamate **6** (1.91 g, 4.46 mmol, 1.0 equiv.), 4-methoxystyrene (1.80 g, 13.39 mmol, 3.0 equiv.), palladium(II) acetate (0.200 g, 0.89 mmol, 0.2 equiv.), PPh_3 (0.468 g, 1.79 mmol, 0.4 equiv.) and triethylamine (10 mL) under argon atmosphere and the reaction mixture was stirred at 120 °C using an oil bath for 18 h. The black suspension was quenched with water (20 mL) and EtOAc (20 mL), filtered through celite and washed with EtOAc. The organic phase was separated and the aqueous phase was extracted with EtOAc (2x30 mL). The combined organic layers were washed with brine (30 mL), dried over Na_2SO_4 and concentrated in *vacuo*. The crude product was purified by column chromatography (hexane/EtOAc, 95:5 to 80:20) to give stilbene **7** (1.34 g, 2.78 mmol, 62% yield). **Appearance:** White solid. **^{1}H NMR** (400 MHz, $CDCl_3$) δ: 7.52 (d, *J* = 8.6 Hz, 2H), 7.36 - 7.22 (m, 6H), 7.06 (s, 1H), 6.91 (d, *J* = 8.6 Hz, 2H), 6.87 (d, *J* = 16.0 Hz, 1H), 4.99 (s, 2H), 4.88 (br.t, *J* = 5.8 Hz, 1H), 3.93 (s, 3H), 3.86 (s, 3H), 3.83 (s, 3H), 3.40 (q, *J* = 6.8 Hz, 2H), 3.13 (t, *J* = 7.1 Hz, 2H) ppm. **^{13}C NMR** (100 MHz, $CDCl_3$) δ: 159.6, 156.6, 152.2, 144.9, 136.7, 134.4, 130.9, 130.1, 129.5, 128.6, 128.2, 128.1 (2C), 127.2, 123.7, 114.3, 108.1, 66.7, 60.8, 56.2, 55.5, 40.7, 30.0 ppm. **HRMS-ESI:** Found $[M+Na]^+$ = 504.1558, 506.1539; $C_{27}H_{28}ClNO_5Na$ requires 504.1548, 506.1532. **m.p.** 107-108 °C.

Benzyl (2-chloro-6-(1,2-dihydroxy-2-(4-methoxyphenyl)ethyl)-3,4-dimethoxyphenethyl)carbamate (8)

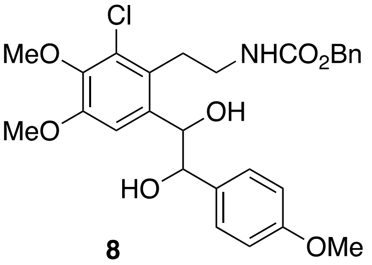

8

A mixture of stilbene **7** (1.34 g, 2.78 mmol, 1.0 equiv.), OsO_4 (5% in H_2O, 0.88 g, 0.14 mmol, 0.05 equiv.) and *N*-methymorpholine oxide (50% in H_2O, 1.44 g, 6.12 mmol, 2.2 equiv.) in THF (20 mL) and H_2O (20 mL) was stirred at room temperature for 3 h. The reaction mixture was quench with Na_2SO_3 (2.11 g, 16.7 mmol, 6.0 equiv.), diluted with H_2O (20 mL) and extracted with EtOAc (3x30 mL). The combined organic layers were washed with brine (20 mL), dried over Na_2SO_4 and concentrated in *vacuo*. The crude product was purified by column chromatography (hexane/EtOAc/Et_3N, 80:20:0.5 to 0:100:0.5) to obtain diol **8** (1.09 g, 2.11 mmol, 76% yield). **Appearance:** Colorless semi-solid. **^{1}H NMR** (300 MHz, $CDCl_3$) δ: 7.44 - 7.23 (m, 5H), 7.06 (d, *J* = 8.3 Hz, 2H), 7.00 (s, 1H), 6.73 (d, *J* = 8.3 Hz, 2H), 5.03 (s, 2H), 5.00 - 4.84 (m, 2H), 4.76 (d, *J* = 7.1 Hz, 1H), 3.86 (s, 3H), 3.80 (s, 3H), 3.74 (s, 3H), 3.57 (br.s, 1H), 3.34 - 3.00 (m, 3H), 2.77-2.60 (m, 1H), 2.55 - 2.34 (m, 1H) ppm. **^{13}C NMR** (75 MHz, $CDCl_3$) δ: 159.1, 156.7, 151.8, 144.7, 136.4, 136.0, 131.8, 128.7, 128.5, 128.1, 128.0 (2C), 127.4, 113.4, 110.3, 78.1, 75.0, 66.6, 60.5, 56.0, 55.1, 40.2, 28.9 ppm. **HRMS-ESI:** Found $[M+Na]^+$ = 538.1590, 540.1575; $C_{27}H_{30}ClNO_7Na$ requires 538.1603, 540.1587.

Benzyl 9-chloro-7,8-dimethoxy-5-(4-methoxyphenyl)-1,2-dihydro-3*H*-benzo[*d*]azepine-3-carboxylate (1s)

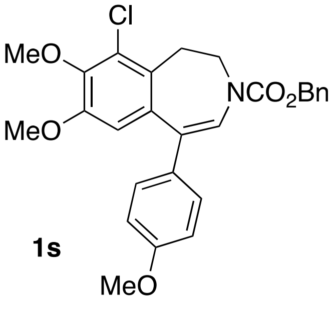

1s

A 50 mL round bottom flask was charged with diol **8** (1.09 g, 2.11 mmol, 1.0 equiv.) and $Sc(OTf)_3$ (0.21 g, 0.42 mmol, 0.2 equiv.) in nitromethane (20 mL) under argon atmosphere. The reaction mixture was stirred at 60 °C using an oil bath for 2 h and the nitromethane was then removed in *vacuo*. The crude product was directly purified by column chromatography (hexane/EtOAc, 80:20) to give ene-carbamate **1s** (0.65 g, 1.35 mmol, 64% yield). **Appearance:** Pale yellow oil. **^{1}H NMR** (400 MHz, $CDCl_3$, mixture of rotamers) δ: 7.45 - 7.28 (m, 5H), 7.28 - 7.16 (m, 2H), 7.14 and 6.99 (2s, 1H), 6.86 (d, *J* = 8.6 Hz, 2H), 6.41 (s, 1H), 5.19 (s, 2H), 4.07 - 3.96 (m, 2H), 3.86 (s, 3H), 3.81 (s, 3H), 3.61 (s, 3H), 3.24 (t, *J* = 5.0 Hz, 2H) ppm. **^{13}C NMR** (100 MHz, $CDCl_3$) δ: Major rotamer 159.0, 154.2, 151.7, 144.5, 136.1, 134.7, 134.2, 131.1, 130.1, 128.6, 128.2, 127.9, 127.1, 126.7, 126.3, 113.9, 112.3, 67.9, 60.7,

56.0, 55.3, 54.7, 28.2 ppm. Minor rotamer 154.7, 136.4, 131.4, 127.2, 125.3, 54.3 ppm. **HRMS-ESI:** Found $[M+Na]^+$ = 502.1407, 504.1389; $C_{27}H_{26}ClNO_5Na$ requires 502.1392, 504.1375.

Synthesis of ene-carbamate 3a

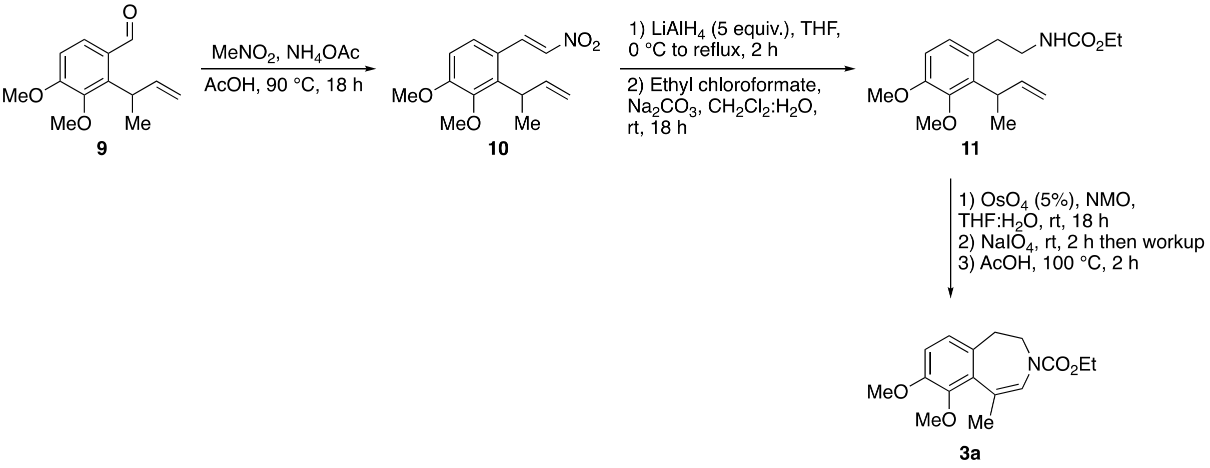

2-(But-3-en-2-yl)-3,4-dimethoxybenzaldehyde **9** was prepared according to reported procedure and spectroscopic data was in agreement with the values reported therein.[2]

(*E*)-2-(But-3-en-2-yl)-3,4-dimethoxy-1-(2-nitrovinyl)benzene (10)

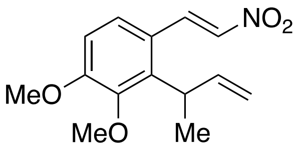

10

A solution of aldehyde **9** (3.24 g, 14.8 mmol, 1.0 equiv.), nitromethane (4.50 g, 73.8 mmol, 5.0 equiv.), and ammonium acetate (1.14 g, 14.8 mmol, 1.0 equiv.) in glacial acetic acid (15 mL) was stirred at 90 °C using an oil bath for 18 h. Then, glacial acetic acid was removed using a rotatory evaporator. The residue was neutralized with sat. $NaHCO_3$ (50 mL) and extracted with EtOAc (3x50 mL). The combined organic layers were washed with brine (50 mL) and dried over Na_2SO_4. After removal of the solvent in *vacuo*, the crude product was purified by column chromatography (hexane/EtOAc, 85:15) to obtain β-nitrostyrene **10** (2.31 g, 8.76 mmol, 59% yield). Spectroscopic data was in agreement with literature values.[3]

Ethyl (2-(but-3-en-2-yl)-3,4-dimethoxyphenethyl)carbamate (11)

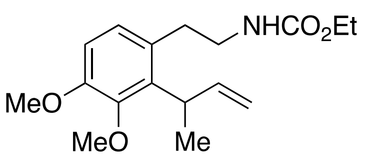

11

A suspension of $LiAlH_4$ (0.998 g, 26.3 mmol, 3.0 equiv.) in dry THF (30 mL) was cooled in an ice-bath under argon atmosphere and a solution of β-nitrostyrene **10** (2.31 g, 8.76 mmol, 1.0 equiv.) in dry THF (10 mL) was added dropwise. The reaction mixture was stirred at 0 °C for 30 min and then heated to reflux using an oil bath for an additional 2 h. After the mixture was cooled to 0 °C again, water (1.0 mL) was slowly added followed by 15% NaOH solution (1.0 mL) and water (3.0 mL), respectively. The mixture was vigorously stirred at room temperature for 30 min. The white precipitate was filtered off through celite and washed with EtOAc (50 mL). The filtrate was dried over Na_2SO_4 and concentrated in *vacuo* to give crude amine as a pale yellow oil. The crude amine was treated with Na_2CO_3 (1.86 g, 17.5 mmol, 2.0 equiv.) in a mixed solvent of CH_2Cl_2 (15 mL) and H_2O (15 mL) at room temperature. Ethyl chloroformate (1.05 g, 9.64 mmol, 1.1 equiv.) in CH_2Cl_2 (4 mL) was added dropwise and the reaction mixture was stirred at room temperature for 18 h. The resulting mixture was extracted with CH_2Cl_2 (3x30 mL). The combined organic layers were dried over Na_2SO_4 and concentrated in *vacuo*. The crude product was purified by column chromatography (hexane/EtOAc, 85:15) to give carbamate **11** (2.04 g, 6.66 mmol, 76% yield over 2 steps). **Appearance:** Pale yellow oil. **^{1}H NMR** (400 MHz, $CDCl_3$) δ: 6.85 (d, J = 8.3 Hz, 1H), 6.74 (d, J = 8.3 Hz, 1H), 6.27 - 6.13 (m, 1H), 5.08 - 4.79 (m, 3H),

4.19 - 4.04 (m, 2H), 3.91 - 3.76 (m, 1H), 3.82 (s, 3H), 3.80 (s, 3H), 3.43 - 3.20 (m, 2H), 2.81 (t, *J* = 7.3 Hz, 2H), 1.43 (d, *J* = 7.1 Hz, 3H), 1.29 - 1.18 (m, 3H) ppm. **^{13}C NMR** (100 MHz, $CDCl_3$) δ: 156.6, 151.8, 148.0, 143.2, 137.8, 129.4, 125.4, 112.8, 110.5, 60.6, 60.5, 55.6, 42.3, 36.9, 33.6, 19.4, 14.7 ppm. **HRMS-ESI:** Found $[M+Na]^+$ = 330.1683; $C_{17}H_{25}NO_4Na$ requires 330.1676.

Ethyl 6,7-dimethoxy-5-methyl-1,2-dihydro-3*H*-benzo[*d*]azepine-3-carboxylate (3a)

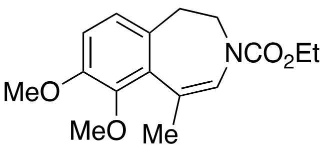

3a

A mixture of carbamate **11** (1.44 g, 4.67 mmol, 1.0 equiv.), OsO_4 (5% in H_2O, 1.48 g, 0.23 mmol, 0.05 equiv.) and *N*-methymorpholine oxide (50% in H_2O, 2.41 g, 10.3 mmol, 2.2 equiv.) in a mixed solvent of THF (20 mL) and H_2O (20 mL) was stirred at room temperature for 2 h. Then $NaIO_4$ (1.20 g, 5.61 mmol, 1.0 equiv.) was added at room temperature and the reaction mixture was stirred for an additional 1 h. The resulting solution was quenched with Na_2SO_3 (3.53 g, 28.0 mmol, 6.0 equiv.), diluted with H_2O (10 mL) and extracted with EtOAc (3x25 mL). The combined organic layers were washed with brine (20 mL), dried over Na_2SO_4 and concentrated in *vacuo*. Then, the crude aldehyde was treated with glacial acetic acid (5 mL) and stirred at 100 °C using an oil bath for 2 h. The excess of glacial acetic acid was removed using a rotatory evaporator. The residue was neutralized with sat. $NaHCO_3$ (50 mL) and extracted with EtOAc (3x25 mL). The combined organic layers were washed with brine (20 mL), dried over Na_2SO_4 and concentrated in *vacuo*. The crude product was purified by column chromatography (hexane/EtOAc, 90:10 to 88:12) to obtain ene-carbamate **3a** (0.80 g, 2.76 mmol, 59% yield over 3 steps). **Appearance:** Pale yellow oil. **^{1}H NMR** (400 MHz, $CDCl_3$, mixture of rotamers) δ: 6.87 (d, *J* = 8.2 Hz, 1H), 6.77 (d, *J* = 8.2 Hz, 1H), 6.57 and 6.39 (2s, 1H), 4.11 (q, *J* = 8.3 Hz, 2H), 3.84 (s, 3H), 3.81 (t, *J* = 5.9 Hz, 2H), 3.76 (s, 3H), 2.76 (t, *J* = 5.6 Hz, 2H), 2.14 (s, 3H), 1.22 (t, *J* = 7.1 Hz, 3H) ppm. **^{13}C NMR** (100 MHz, $CDCl_3$) δ: Major rotamer 155.2, 151.8, 146.6, 132.7, 132.2, 124.7, 124.0, 123.2, 111.3, 61.6, 60.6, 55.9, 55.3, 31.6, 19.9, 14.8 ppm. Minor rotamer 155.4, 152.0, 146.8, 132.4, 125.7, 123.0, 111.2, 60.7, 55.2, 31.9, 20.1, 14.6 ppm. **HRMS-ESI:** Found $[M+Na]^+$ = 314.1370; $C_{16}H_{21}NO_4Na$ requires 314.1363.

Synthesis of ene-carbamates 3b-e

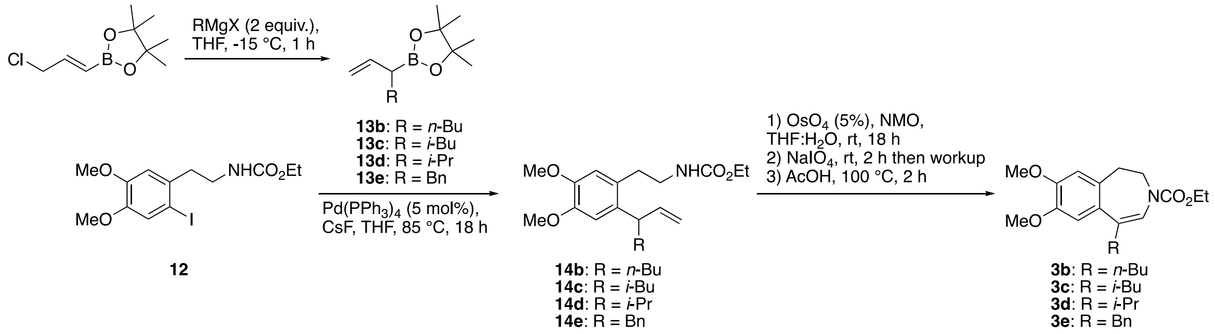

Allylboronic esters **13b-13e** were prepared according to the literature procedures and used without purification.[4]

Ethyl 5-butyl-7,8-dimethoxy-1,2-dihydro-3*H*-benzo[*d*]azepine-3-carboxylate (3b)

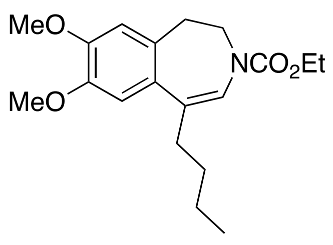

3b

A 100 mL ace pressure tube was charged with ethyl (2-iodo-4,5-dimethoxyphenethyl)carbamate **12** (0.61 g, 1.60 mmol, 1.0 equiv.), allylboronic ester **13b** (0.43 g, 1.92 mmol, 1.2 equiv.), $Pd(PPh_3)_4$ (0.13 g, 0.11 mmol, 0.07 equiv.) and CsF (0.97 g, 6.40 mmol, 3.8 equiv.) in dry THF (10 mL) under argon atmosphere. The reaction mixture was stirred at 95 °C using an oil bath for 48 h and afterwards cooled to room temperature. The resulting black suspension was quenched with water (20 mL) and EtOAc (20 mL), filtered through celite and washed with EtOAc. The organic phase was separated and the aqueous phase was extracted with EtOAc (2x30 mL). The combined organic layers were washed with brine (30 mL), dried over Na_2SO_4 and concentrated in *vacuo*. The crude carbamate **14b** was obtained and used without purification.

A mixture of crude carbamate **14b**, OsO_4 (5% in H_2O, 0.51 g, 0.08 mmol, 0.05 equiv.) and *N*-methymorpholine oxide (50% in H_2O, 0.83 g, 3.52 mmol, 2.2 equiv.) in THF (10 mL) and H_2O (10 mL) was stirred at room temperature for 18 h. Then $NaIO_4$ (0.411 g, 1.92 mmol, 1.2 equiv.) was added at room temperature and the reaction mixture was stirred for another 1 h. The resulting solution was quenched with Na_2SO_3 (1.24 g, 9.60 mmol, 6.0 equiv.), diluted with H_2O (10 mL) and extracted with EtOAc (3x25 mL). The combined organic layers were washed with brine (20 mL), dried over Na_2SO_4 and concentrated in *vacuo*. Then the crude aldehyde was treated with glacial acetic acid (4 mL) and stirred at 100 °C using an oil bath for 2 h. The excess of glacial acetic acid was removed using a rotatory evaporator. The residue was neutralized with sat. $NaHCO_3$ (30 mL) and extracted with EtOAc (3x25 mL). The combined organic layers were washed with brine (20 mL), dried over Na_2SO_4 and concentrated in *vacuo*. The crude product was purified by column chromatography (hexane/EtOAc, 90:10 to 85:15) to obtain ene-carbamate **3b** (46.8 mg, 0.14 mmol, 9% yield over 4 steps). **Appearance:** Pale yellow oil. **^{1}H NMR** (400 MHz, $CDCl_3$, mixture of rotamers) δ: 6.81 (s, 1H), 6.77 and 6.62 (2s, 1H), 6.67 (s, 1H), 4.16 (q, *J* = 6.9 Hz, 2H), 3.99 - 3.90 (m, 2H), 3.88 (s, 3H), 3.86 (s, 3H), 2.84 (t, *J* = 5.5 Hz, 2H), 2.55 - 2.43 (m, 2H), 1.40 - 1.20 (m, 7H), 0.87 (t, *J* = 6.9 Hz, 3H) ppm. **^{13}C NMR** (100 MHz, $CDCl_3$) δ: Major rotamer 154.6, 147.7 (2C), 133.4, 129.5, 124.8, 122.9, 111.6, 110.0, 62.0, 56.1, 56.0, 54.9, 35.7, 32.8, 31.4, 22.3, 14.7, 14.0 ppm. Minor rotamer 155.0, 133.1, 129.8, 125.0, 123.7, 54.4, 35.6, 32.9 ppm. **HRMS-ESI:** Found $[M+Na]^+$ = 356.1845; $C_{19}H_{27}NO_4Na$ requires 356.1832.

Ethyl 5-isobutyl-7,8-dimethoxy-1,2-dihydro-3*H*-benzo[*d*]azepine-3-carboxylate (3c)

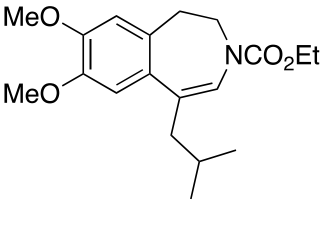

3c

A 100 mL ace pressure tube was charged with ethyl (2-iodo-4,5-dimethoxyphenethyl)carbamate **12** (1.21 g, 3.20 mmol, 1.0 equiv.), allylboronic ester **13c** (1.08 g, 4.80 mmol, 1.5 equiv.), $Pd(PPh_3)_4$ (0.19 g, 0.16 mmol, 0.05 equiv.) and CsF (1.94 g, 12.80 mmol, 4.0 equiv.) in dry THF (15 mL) under argon atmosphere. The reaction mixture was stirred at 85 °C using an oil bath for 18 h and afterwards cooled to room temperature. The resulting black suspension was quenched with water (20 mL) and EtOAc (20 mL), filtered through celite and washed with EtOAc. The organic phase was separated and the aqueous phase was extracted with EtOAc (2x30 mL). The combined organic layers were washed with brine (30 mL), dried over Na_2SO_4 and concentrated in *vacuo*. The crude carbamate **14c** was obtained and used without purification.

A mixture of crude carbamate **14c**, OsO_4 (5% in H_2O, 1.02 g, 0.16 mmol, 0.05 equiv.) and *N*-methymorpholine oxide (50% in H_2O, 1.65 g, 7.04 mmol, 2.2 equiv.) in THF (15 mL) and H_2O (15 mL) was stirred at room temperature for 18 h. Then, $NaIO_4$ (0.821 g, 3.84 mmol, 1.2 equiv.) was added at room temperature and the reaction mixture was stirred for another 1 h. The resulting solution was quenched with Na_2SO_3 (2.42 g, 19.20 mmol, 6.0 equiv.), diluted with H_2O (20 mL) and extracted with EtOAc (3x30 mL). The combined organic layers were washed with brine (30 mL), dried over Na_2SO_4 and concentrated in *vacuo*. Then, the crude aldehyde was treated with glacial acetic acid (5 mL) and stirred at 100 °C using an oil bath for 2 h. The excess of glacial acetic acid was removed using a rotatory evaporator. The residue was neutralized with sat. $NaHCO_3$ (40 mL) and extracted with EtOAc (3x25 mL). The combined organic layers were washed with brine (20 mL), dried over Na_2SO_4 and concentrated in *vacuo*. The crude product was purified by column chromatography (hexane/EtOAc, 90:10 to 85:15) to obtain ene-carbamate **3c** (75.1 mg, 0.21 mmol, 7% yield over 4 steps). **Appearance:** White solid. **^{1}H NMR** (300 MHz, $CDCl_3$, mixture of rotamers) δ: 6.80 (s, 1H), 6.75 and 6.60 (2s, 1H), 6.67 (s, 1H), 4.16 (q, *J* = 7.1 Hz, 2H), 4.00 - 2.91 (m, 2H), 3.88 (s, 3H), 3.86 (s, 3H), 2.86 (t, *J* = 5.5 Hz, 2H), 2.36 (d, *J* = 6.7 Hz, 2H), 1.59 (quintet, *J* = 6.7 Hz, 1H), 1.34 - 1.20 (m, 3H), 0.85 (d, *J* = 6.6 Hz, 6H) ppm. **^{13}C NMR** (75 MHz, $CDCl_3$) δ: Major rotamer 154.5, 147.7 (2C), 133.3, 129.6, 123.7 (2C), 111.7, 110.2, 62.0, 56.1, 55.9, 55.1, 45.4, 32.8, 27.1, 22.5 (2C), 14.7 ppm. Minor rotamer 154.9, 133.0, 129.8, 124.4, 54.5 ppm. **HRMS-ESI:** Found $[M+Na]^+$ = 356.1831; $C_{19}H_{27}NO_4Na$ requires 356.1832. **m.p.** 104-106 °C.

Ethyl 5-isopropyl-7,8-dimethoxy-1,2-dihydro-3*H*-benzo[*d*]azepine-3-carboxylate (3d)

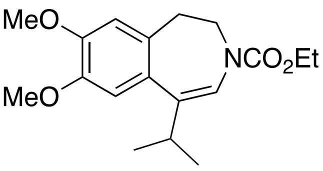

3d

A 100 mL ace pressure tube was charged with ethyl (2-iodo-4,5-dimethoxyphenethyl)carbamate **12** (0.95 g, 2.50 mmol, 1.0 equiv.), allylboronic ester **13d** (0.79 g, 3.75 mmol, 1.5 equiv.), $Pd(PPh_3)_4$ (0.14 g, 0.13 mmol, 0.05 equiv.) and CsF (1.52 g, 10.0 mmol, 4.0 equiv.) in dry THF (15 mL) under argon atmosphere. The reaction mixture was stirred at 85 °C using an oil bath for 18 h and afterwards cooled to room temperature. The resulting black suspension was quenched with water (20 mL) and EtOAc (20 mL), filtered through celite and washed with EtOAc. The organic phase was separated and the aqueous phase was extracted with EtOAc (2x30 mL). The combined organic layers were washed with brine (30 mL), dried over Na_2SO_4 and concentrated in *vacuo*. The crude product **14d** was obtained and used without purification.

A mixture of crude carbamate **14d**, OsO_4 (5% in H_2O, 0.80 g, 0.13 mmol, 0.05 equiv.) and *N*-methymorpholine oxide (50% in H_2O, 1.29 g, 5.50 mmol, 2.2 equiv.) in THF (10 mL) and H_2O (10 mL) was stirred at room temperature for 18 h. Then, $NaIO_4$ (0.642 g, 3.00 mmol, 1.2 equiv.) was added at room temperature and the reaction mixture was stirred for another 1 h. The resulting solution was quenched with Na_2SO_3 (1.89 g, 15.0 mmol, 6.0 equiv.), diluted with H_2O (10 mL) and extracted with EtOAc (3x25 mL). The combined organic layers were washed with brine (20 mL), dried over Na_2SO_4

and concentrated in *vacuo*. Then, the crude aldehyde was treated with glacial acetic acid (4 mL) and stirred at 100 °C using an oil bath for 2 h. An excess of glacial acetic acid was removed using a rotatory evaporator. The residue was neutralized with sat. $NaHCO_3$ (30 mL) and extracted with EtOAc (3x25 mL). The combined organic layers were washed with brine (20 mL), dried over Na_2SO_4 and concentrated in *vacuo*. The crude product was purified by column chromatography (hexane/EtOAc, 80:20) to obtain ene-carbamate **3d** (0.30 g, 1.01 mmol, 38% yield over 4 steps). **Appearance:** Colorless oil. **^{1}H NMR** (400 MHz, $CDCl_3$, mixture of rotamers) δ: 6.80 (s, 1H), 6.73 (s, 1H), 6.57 and 6.42 (2s, 1H), 4.13 (q, J = 7.1 Hz, 2H), 3.96 - 3.82 (m, 2H), 3.89 (s, 3H), 3.86 (s, 3H), 2.86 (quintet, J = 6.7 Hz, 1H), 2.78 (t, J = 5.7 Hz, 2H), 1.31 - 1.19 (m, 3H), 1.13 (d, J = 6.5 Hz, 6H) ppm. **^{13}C NMR** (100 MHz, $CDCl_3$) δ: Major rotamer 155.2, 147.8 (2C), 133.6, 132.9, 130.7, 121.4, 111.3, 109.7, 61.8, 56.14, 56.07, 55.9, 32.6, 32.0, 22.3 (2C), 14.6 ppm. Minor rotamer 155.5, 132.6, 131.0, 122.1, 111.1, 55.6, 32.7, 32.1 ppm. **HRMS-ESI:** Found $[M+H]^+$ = 320.1858; $C_{18}H_{26}NO_4$ requires 320.1856.

Ethyl 5-benzyl-7,8-dimethoxy-1,2-dihydro-3*H*-benzo[d]azepine-3-carboxylate (3e)

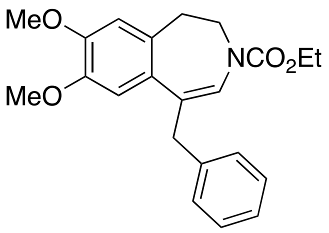

3e

A 100 mL ace pressure tube was charged with ethyl (2-iodo-4,5-dimethoxyphenethyl)carbamate **12** (0.86 g, 2.27 mmol, 1.0 equiv.), allylboronic ester **13e** (0.76 g, 2.95 mmol, 1.3 equiv.), $Pd(PPh_3)_4$ (0.18 g, 0.16 mmol, 0.07 equiv.) and CsF (1.38 g, 9.08 mmol, 4.0 equiv.) in dry THF (10 mL) under argon atmosphere. The reaction mixture was stirred at 85 °C using an oil bath for 18 h and afterwards cooled to room temperature. The resulting black suspension was quenched with water (20 mL) and EtOAc (20 mL), filtered through celite and washed with EtOAc. The organic phase was separated and the aqueous phase was extracted with EtOAc (2x30 mL). The combined organic layers were washed with brine (30 mL), dried over Na_2SO_4 and concentrated in *vacuo*. The crude carbamate **14e** was obtained and used without purification.

A mixture of crude carbamate **14e**, OsO_4 (5% in H_2O, 0.72 g, 0.11 mmol, 0.05 equiv.) and *N*-methymorpholine oxide (50% in H_2O, 1.17 g, 4.99 mmol, 2.2 equiv.) in THF (10 mL) and H_2O (10 mL) was stirred at room temperature for 18 h. Then, $NaIO_4$ (0.583 g, 2.72 mmol, 2.2 equiv.) was added at room temperature and the reaction mixture was stirred for another 1 h. The resulting solution was quenched with Na_2SO_3 (1.72 g, 13.62 mmol, 6.0 equiv.), diluted with H_2O (10 mL) and extracted with EtOAc (3x25 mL). The combined organic layers were washed with brine (20 mL), dried over Na_2SO_4 and concentrated in *vacuo*. Then, the crude aldehyde was treated with glacial acetic acid (4 mL) and stirred at 100 °C using an oil bath for 2 h. An excess of glacial acetic acid was removed using a rotatory evaporator. The residue was neutralized with sat. $NaHCO_3$ (30 mL) and extracted with EtOAc (3x25 mL). The combined organic layers were washed with brine (20 mL), dried over Na_2SO_4 and concentrated in *vacuo*. The crude product was purified by column chromatography (hexane/EtOAc, 90:10 to 85:15) to obtain ene-carbamate **3e** (0.19 g, 0.52 mmol, 23% yield over 4 steps). **Appearance:** Pale yellow oil. **^{1}H NMR** (400 MHz, $CDCl_3$, mixture of rotamers) δ: 7.27 - 7.15 (m, 4H), 7.15 - 7.07 (m, 1H), 7.04 and 6.84 (2s, 1H), 6.78 (s, 1H), 6.58 (s, 1H), 4.18 (q, J = 7.1 Hz, 2H), 4.03 - 3.90 (m, 2H), 3.79 (s, 5H), 3.68 (s, 3H), 2.83 (t, J = 5.0 Hz, 2H), 1.27 (t, J = 7.1 Hz, 3H) ppm. **^{13}C NMR** (100 MHz, $CDCl_3$) δ: Major rotamer 154.0, 147.3, 147.2, 140.4, 133.1, 129.1, 128.2, 128.1, 126.0, 125.1, 121.0, 111.5, 110.4, 62.0, 55.66, 55.65, 53.2, 42.5, 33.3, 14.5 ppm. Minor rotamer 154.5, 132.9, 125.8, 121.3, 53.0 ppm. **HRMS-ESI:** Found $[M+Na]^+$ = 390.1684; $C_{22}H_{25}NO_4Na$ requires 390.1676.

General procedure for the asymmetric hydrogenation

An oven-dried vial was charged with ene-carbamate (0.05 mmol, 1.0 equiv.) and Ir-N,P-catalyst **A** (1.0 mol%). Freshly distilled DCM (1 mL) and a magnetic stirring bar were added and the vial was placed in a high-pressure hydrogenation apparatus. The reactor was purged three times with Ar, purged three times with H_2 and then pressurized with H_2 (100 bar). The reaction was stirred at room temperature for 16 h before the H_2 pressure was released and the solvent was removed under reduced pressure. The residue was purified by flash chromatography (pentane/Et2O, 30:70) on silica gel to give the tetrahydro-3-benzazepine. The *ee* value was determined by SFC analysis on a chiral stationary phase. The corresponding racemic product was used for comparison and it was prepared on a 0.05 mmol scale using Pd/C (or racemic Ir-catalyst) as the catalyst, following the same asymmetric hydrogenation procedure. The absolute configuration of **2a** was determined after reduction of the carbamate group by $LiAlH_4$ and consecutive comparison of the sign of optical rotation with reported values. The assignment is tentative for the other products and similar reaction mechanism is assumed.

Procedure for the gram-scale asymmetric hydrogenation

An oven-dried vial was charged with ene-carbamate **1a** (1.00 g, 2.83 mmol, 1.0 equiv.) and Ir-N,P-catalyst **A** (45.8 mg, 1.0 mol%). Freshly distilled DCM (10 mL) and a magnetic stirring bar were added and the vial was placed in a high-pressure hydrogenation apparatus. The reactor was purged three times with Ar, purged three times with H_2 and then pressurized with H_2 (100 bar). The reaction was stirred at room temperature for 16 h before the H_2 pressure was released and the solvent was removed under reduced pressure. The residue was purified by flash chromatography (pentane/Et2O, 30:70) on silica gel to give the tetrahydro-3-benzazepine **2a** (0.99 g, 2.78 mmol, 98% yield). The *ee* value was determined by SFC analysis using Chiralcel OJ-H chiral stationary phase. The corresponding racemic product was used for comparison and it was prepared on a 0.05 mmol scale using Pd/C as the catalyst, following the general procedure for the asymmetric hydrogenation. The absolute configuration was determined after reduction of the carbamate group by $LiAlH_4$ and consecutive comparison of the sign of optical rotation with reported values.

Characterization of hydrogenated products

Ethyl (*S*)-7,8-dimethoxy-1-phenyl-1,2,4,5-tetrahydro-3*H*-benzo[*d*]azepine-3-carboxylate (2a)

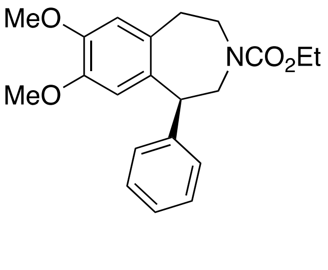

2a

Prepared according to the general procedure for the asymmetric hydrogenation on a 0.05 mmol scale (17.6 mg, 99% yield). **Appearance:** Colorless oil. **^{1}H NMR** (400 MHz, $CDCl_3$, mixture of rotamers) δ: 7.31 - 7.26 (m, 2H), 7.24 - 7.18 (m, 1H), 7.15 - 7.08 (m, 2H), 6.69 - 6.64 (m, 1H), 6.45 (d, *J* = 4.6 Hz, 1H), 4.40 (dd, *J* = 44.0, 5.6 Hz, 1H), 4.11 - 4.00 (m, 3H), 3.97 - 3.91 (m, 1H), 3.88 (s, 3H), 3.69 (s, 3H), 3.68 - 3.63 (m, 1H), 3.59 - 3.51 (m, 1H), 3.10 (ddd, *J* = 14.8, 10.1, 4.4 Hz, 1H), 2.78 (dd, *J* = 28.5, 15.3 Hz, 1H), 1.20 (m, 3H) ppm. **^{13}C NMR** (125 MHz, $CDCl_3$) δ: Major rotamer 156.2, 147.4, 147.3, 142.4, 133.1, 131.5, 128.5, 128.2, 126.5, 114.5, 113.9, 61.3, 56.0, 55.9, 52.0, 50.0, 46.8, 35.2, 14.8 ppm. Minor rotamer 156.4, 147.5, 147.2, 142.8, 133.3, 130.8, 128.2, 113.6, 61.3, 51.1, 49.4, 46.5, 34.6, 14.7 ppm. **HRMS-ESI:** Found $[M+Na]^+$ = 378.1694; $C_{21}H_{25}NO_4Na$ requires 378.1676. **$[\alpha]_D^{25}$** = +33 (c = 0.1, $CHCl_3$).

Ethyl (*S*)-1-(4-fluorophenyl)-7,8-dimethoxy-1,2,4,5-tetrahydro-3*H*-benzo[*d*]azepine-3-carboxylate (2b)

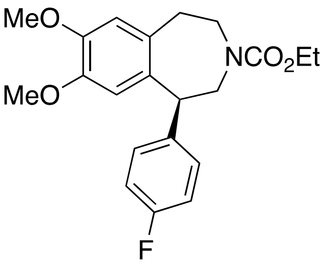

2b

Prepared according to the general procedure for the asymmetric hydrogenation on a 0.05 mmol scale (18.3 mg, 98% yield). **Appearance:** White solid. **^{1}H NMR** (400 MHz, $CDCl_3$, mixture of rotamers) δ: 7.07 (q, 2H), 7.02 - 6.92 (m, 2H), 6.66 (d, *J* = 6.2 Hz, 1H), 6.43 (d, *J* = 3.8 Hz, 1H), 4.36 (d, *J* = 35.9 Hz, 1H), 4.05 (dd, *J* = 14.8, 6.2 Hz, 2H), 3.94 (d, *J* = 9.3 Hz, 2H), 3.88 (s, 3H), 3.71 (s, 3H), 3.64 (d, *J* = 24.3 Hz, 2H), 3.05 (dd, *J* = 15.4, 8.5 Hz, 1H), 2.78 (dd, *J* = 29.4, 15.3 Hz, 1H), 1.20 (m, 3H) ppm. **^{13}C NMR** (125 MHz, $CDCl_3$) δ: major rotamer 161.5 (d, *J* = 244.8 Hz), 156.1, 147.4, 147.3, 138.0, 132.9, 131.4, 129.7 (d, *J* = 7.2 Hz), 115.2 (d, *J* = 21.0 Hz), 114.4, 114.0, 61.3, 55.9, 55.8, 51.3, 49.9, 46.7, 35.1, 14.7 ppm. Minor rotamer 156.3, 147.5, 147.2, 138.3, 133.2, 130.9, 113.8, 61.3, 50.5, 49.3, 46.5, 34.7, 14.6 ppm. **^{19}F NMR** (376 MHz, $CDCl_3$, mixture of rotamers) δ: -116.60, -116.74 ppm. **^{19}F NMR** (376 MHz, CD_3OD) δ: -118.91 ppm. **HRMS-ESI:** Found $[M+Na]^+$ = 396.1588; $C_{21}H_{24}FNO_4Na$ requires 396.1582. **$[\alpha]_D^{25}$** = +37 (c = 0.1, $CHCl_3$). **m.p.** 102 °C.

Ethyl (*S*)-7,8-dimethoxy-1-(*p*-tolyl)-1,2,4,5-tetrahydro-3*H*-benzo[*d*]azepine-3-carboxylate (2c)

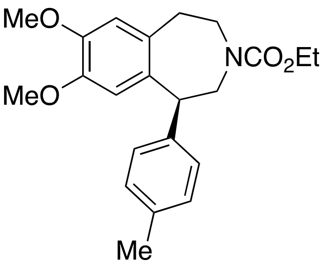

2c

Prepared according to the general procedure for the asymmetric hydrogenation on a 0.05 mmol scale (17.5 mg, 95% yield). **Appearance:** Colorless oil. **^{1}H NMR** (400 MHz, $CDCl_3$, mixture of rotamers) δ: 7.09 (d, *J* = 7.9 Hz, 2H), 7.01 (t, *J* = 9.6 Hz, 2H), 6.65 (d, *J* = 7.1 Hz, 1H), 6.46 (d, *J* = 6.7 Hz, 1H), 4.47 - 4.28 (m, 1H), 4.12 - 3.99 (m, 3H), 3.92 - 3.89 (m, 1H), 3.88 (s, 3H), 3.70 (s, 3H), 3.68 - 3.52 (m, 2H), 3.10 (t, *J* = 13.0 Hz, 1H), 2.77 (dd, *J* = 28.9, 15.4 Hz, 1H), 2.32 (s, 3H), 1.20 (m, 3H) ppm. **^{13}C NMR** (125 MHz, $CDCl_3$) δ: Major rotamer 156.3, 147.4, 147.2, 139.4, 136.0, 133.4, 131.4, 129.2, 128.1, 114.6, 113.9, 61.3, 56.0, 55.9, 51.6, 50.0, 46.7, 35.1, 21.1, 14.8 ppm. Minor rotamer 156.4, 147.5, 147.1, 139.8, 136.0, 133.5, 130.7, 128.0, 114.5, 113.6, 61.3, 50.8, 49.4, 46.5, 34.6, 14.7 ppm. **HRMS-ESI:** Found $[M+Na]^+$ = 392.1856; $C_{22}H_{27}NO_4Na$ requires 392.1832. **$[\alpha]_D^{26}$** = +29 (c = 0.1, $CHCl_3$).

Ethyl (*S*)-7,8-dimethoxy-1-(4-methoxyphenyl)-1,2,4,5-tetrahydro-3*H*-benzo[*d*]azepine-3-carboxylate (2d)

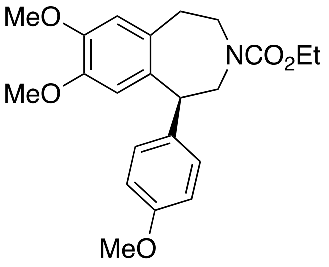

2d

Prepared according to the general procedure for the asymmetric hydrogenation on a 0.05 mmol scale (18.9 mg, 98% yield). **Appearance:** Colorless oil. **^{1}H NMR** (400 MHz, $CDCl_3$, mixture of rotamers) δ: 7.02 (t, *J* = 10.4 Hz, 2H), 6.82 (dt, *J* = 8.7, 2.2 Hz, 2H), 6.65 (d, *J* = 6.9 Hz, 1H), 6.45 (d, *J* = 4.9 Hz, 1H), 4.43 - 4.26 (m, 1H), 4.11 - 3.98 (m, 3H), 3.92 - 3.89 (m, 1H), 3.88 (s, 3H), 3.78 (s, 3H), 3.70 (s, 3H), 3.67 - 3.55 (m, 2H), 3.13 - 3.02 (m, 1H), 2.77 (dd, *J* = 29.8, 15.2 Hz, 1H), 1.19 (m, 3H) ppm. **^{13}C NMR** (125 MHz, $CDCl_3$) δ: Major rotamer 158.2, 156.2, 147.4, 147.2, 134.4, 133.6, 131.4, 129.2, 114.5, 113.9, 113.9, 61.3, 56.0, 55.9, 55.3, 51.2, 50.2, 46.7, 35.2, 14.8 ppm. Minor rotamer 156.4, 147.5, 147.1, 134.9, 133.7, 130.8, 129.1, 113.7, 50.4, 49.5, 46.5, 34.7, 14.7 ppm. **HRMS-ESI:** Found $[M+Na]^+$ = 408.1787; $C_{22}H_{27}NO_5Na$ requires 408.1781. **$[\alpha]_D$**^{26} = +30 (c = 0.1, $CHCl_3$).

Ethyl (*S*)-1-(4-hydroxy-3-methoxyphenyl)-7,8-dimethoxy-1,2,4,5-tetrahydro-3*H*-benzo[*d*]azepine-3-carboxylate (2e)

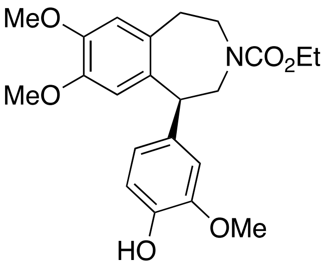

2e

Prepared according to the general procedure for the asymmetric hydrogenation on a 0.05 mmol scale (19.0 mg, 95% yield). **Appearance:** Colorless oil. **^{1}H NMR** (500 MHz, DMSO-d_6, 80 °C) δ 6.79 (s, 1H), 6.75 (d, *J* = 2.1 Hz, 1H), 6.72 (d, *J* = 8.1 Hz, 1H), 6.52 - 6.49 (m, 2H), 4.26 (t, *J* = 6.6 Hz, 1H), 4.02 - 3.95 (m, 2H), 3.85 (d, *J* = 6.5 Hz, 2H), 3.77 (s, 3H), 3.74 (s, 3H), 3.61 (s, 3H), 3.58 - 3.49 (m, 2H), 3.06 - 2.97 (m, 1H), 2.86 - 2.79 (m, 1H), 1.12 (t, *J* = 7.1 Hz, 3H) ppm. **^{13}C NMR** (125 MHz, $CDCl_3$) δ: Major rotamer 156.5, 147.5, 147.2, 146.6, 144.2, 134.6, 133.9, 131.1, 120.8, 114.6, 114.4, 113.8, 110.9, 61.5, 56.1, 56.0, 50.8, 49.5, 46.6, 34.9, 14.8 ppm. Minor rotamer 156.4, 147.4, 147.3, 144.3, 133.7, 131.4, 121.1, 113.9, 51.6, 50.4, 46.8, 35.3, 14.8 ppm. **HRMS-ESI:** Found $[M+Na]^+$ = 424.1730; $C_{22}H_{27}NO_6Na$ requires 424.1731. **$[\alpha]_D$**^{25} = +35 (c = 0.1, $CHCl_3$).

Ethyl (*S*)-1-(3,4-dimethoxyphenyl)-7,8-dimethoxy-1,2,4,5-tetrahydro-3*H*-benzo[*d*]azepine-3-carboxylate (2f)

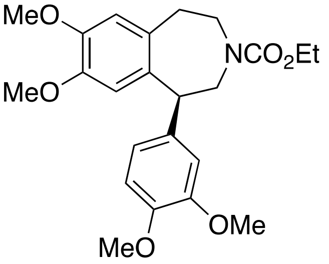

2f

Prepared according to the general procedure for the asymmetric hydrogenation on a 0.05 mmol scale (20.1 mg, 97% yield). **Appearance:** Yellow oil. **^{1}H NMR** (400 MHz, $CDCl_3$, mixture of rotamers) δ: 6.84 - 6.73 (m, 2H), 6.68 - 6.62 (m, 2H), 6.57 - 6.42 (m, 1H), 4.42 - 4.24 (m, 1H), 4.14 - 3.93 (m, 4H), 3.88 (s, 3H), 3.85 (s, 3H), 3.82 (s, 3H), 3.71 (d, *J* = 8.4 Hz, 3H), 3.66 - 3.54 (m, 2H), 3.13 - 2.96 (m, 1H), 2.78 (dd, *J* = 52.2, 15.5 Hz, 1H), 1.19 (t, *J* = 7.1 Hz, 3H) ppm. **^{13}C NMR** (125 MHz, $CDCl_3$) δ: Major rotamer 156.4, 148.8, 147.5, 147.4, 147.1, 135.1, 133.8, 131.1, 120.0, 114.5, 113.7, 111.5, 111.1, 61.4, 56.0, 55.9, 55.9, 50.7, 49.3, 46.4, 34.8, 14.7 ppm. Minor rotamer 156.2, 148.9, 147.7, 147.3, 147.2, 133.5, 131.4, 120.3, 114.3, 113.9, 111.7, 111.2, 61.3, 51.4, 50.3, 46.7, 35.2, 14.8 ppm. **HRMS-ESI:** Found $[M+Na]^+$ = 438.1884; $C_{23}H_{29}NO_6Na$ requires 438.1887. **$[\alpha]_D$**^{25} = +31 (c = 0.1, $CHCl_3$).

Ethyl (*S*)-5-phenyl-5,6,8,9-tetrahydro-7*H*-[1,3]dioxolo[4',5':4,5]benzo[1,2-*d*]azepine-7-carboxylate (2g)

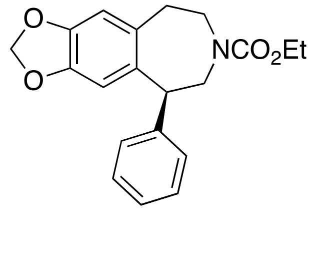

2g

Prepared according to the general procedure for the asymmetric hydrogenation on a 0.05 mmol scale (16.8 mg, 99% yield). **Appearance:** Colorless oil. **^{1}H NMR** (400 MHz, $CDCl_3$, mixture of rotamers) δ: 7.30 (tt, *J* = 7.6, 1.4 Hz, 2H), 7.22 (tt, *J* = 7.3, 1.2 Hz, 1H), 7.17 - 7.09 (m, 2H), 6.65 (s, 1H), 6.40 (s, 1H), 5.88 (s, 2H), 4.45 - 4.29 (m, 1H), 4.14 - 3.94 (m, 3H), 3.81 (dd, *J* = 14.5, 9.0 Hz, 1H), 3.76 - 3.67 (m, 1H), 3.58 - 3.48 (m, 1H), 3.05 (ddd, *J* = 14.6, 10.4, 3.8 Hz, 1H), 2.80 (t, *J* = 17.8 Hz, 1H), 1.21 (m, 3H) ppm. **^{13}C NMR** (125 MHz, $CDCl_3$) δ: Major rotamer 156.2, 146.3, 146.0, 142.3, 134.5, 132.3, 128.5, 128.1, 126.6, 110.9, 110.6, 100.9, 61.3, 51.8, 49.7, 46.5, 35.1, 14.7 ppm. Minor rotamer 156.3, 145.9, 142.5, 134.8, 131.9, 111.1, 110.3, 51.0, 49.3, 46.2, 34.8, 14.7 ppm. **HRMS-ESI:** Found $[M+Na]^+$ = 362.1353; $C_{20}H_{21}NO_4Na$ requires 362.1363. **$[\alpha]_D$**^{26} = +18 (c = 0.1, $CHCl_3$).

Ethyl (*S*)-5-(4-methoxyphenyl)-5,6,8,9-tetrahydro-7*H*-[1,3]dioxolo[4',5':4,5]benzo[1,2-*d*]azepine-7- carboxylate (2h)

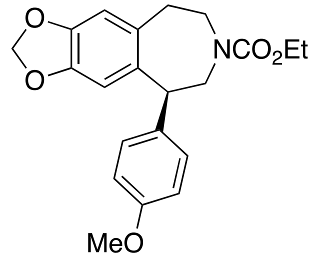

2h

Prepared according to the general procedure for the asymmetric hydrogenation on a 0.05 mmol scale (18.3 mg, 99% yield). **Appearance:** Colorless oil. **^{1}H NMR** (400 MHz, $CDCl_3$, mixture of rotamers) δ: 7.09 - 7.00 (m, 2H), 6.88 - 6.81 (m, 2H), 6.63 (s, 1H), 6.39 (s, 1H), 5.88 (s, 2H), 4.37 - 4.23 (m, 1H), 4.12 - 3.91 (m, 3H), 3.83 - 3.75 (m, 1H), 3.78 (s, 3H), 3.74 - 3.63 (m, 1H), 3.53 (dt, *J* = 13.2, 4.7 Hz, 1H), 3.03 (ddd, *J* = 14.3, 10.2, 3.8 Hz, 1H), 2.79 (t, *J* = 18.5 Hz, 1H), 1.21 (t, *J* = 7.2 Hz, 3H) ppm. **^{13}C NMR** (125 MHz, $CDCl_3$) δ: Major rotamer 158.3, 156.3, 146.4, 146.0, 135.0, 134.4, 132.3, 129.1, 114.0, 110.9, 110.6, 101.0, 61.3, 55.3, 51.0, 50.0, 46.5, 35.2, 14.8 ppm. Minor rotamer 156.4, 145.9, 135.3, 134.6, 131.9, 111.1, 110.3, 50.3, 49.5, 46.3, 34.9, 14.7 ppm. **HRMS-ESI:** Found $[M+Na]^+$ = 392.1476; $C_{21}H_{23}NO_5Na$ requires 392.1468. **$[\alpha]_D$**^{25} = +14 (c = 0.1, $CHCl_3$).

Ethyl (*S*)-7-methoxy-1-phenyl-1,2,4,5-tetrahydro-3*H*-benzo[*d*]azepine-3-carboxylate (2i)

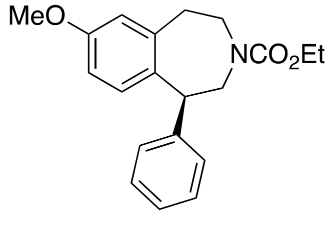

2i

Prepared according to the general procedure for the asymmetric hydrogenation on a 0.05 mmol scale (15.9 mg, 98% yield). **Appearance:** Colorless oil. **^{1}H NMR** (400 MHz, $CDCl_3$, mixture of rotamers) δ: 7.29 (tt, *J* = 7.6, 1.3 Hz, 2H), 7.21 (tt, *J* = 7.3, 1.3 Hz, 1H), 7.16 - 7.08 (m, 2H), 6.83 (d, *J* = 8.3 Hz, 1H), 6.72 (s, 1H), 6.66 (dd, *J* = 8.5, 2.8 Hz, 1H), 4.46 - 4.32 (m, 1H), 4.12 - 4.00 (m, 3H), 3.97 - 3.91 (m, 1H), 3.79 (s, 3H), 3.74 - 3.56 (m, 2H), 3.09 (t, *J* = 14.3 Hz, 1H), 2.86 (t, *J* = 18.6 Hz, 1H), 1.23 - 1.16 (m, 3H) ppm. **^{13}C NMR** (125 MHz, $CDCl_3$) δ: Major rotamer 158.2, 156.3, 142.6, 140.9, 133.7, 131.9, 128.5 , 128.3 , 126.5 , 116.2 , 111.7, 61.4, 55.3, 51.5, 50.4, 46.6, 36.2, 14.8 ppm. Minor rotamer 158.2, 156.3, 142.8, 140.4, 133.9, 132.0, 111.6, 50.6, 49.8, 46.4, 35.8 ppm. **HRMS-ESI:** Found $[M+Na]^+$ = 348.1575; $C_{20}H_{23}NO_3Na$ requires 348.1581. **$[\alpha]_D$**^{27} = +27 (c = 0.1, $CHCl_3$).

Ethyl (*S*)-1-(4-fluorophenyl)-7-methoxy-1,2,4,5-tetrahydro-3*H*-benzo[*d*]azepine-3-carboxylate (2j)

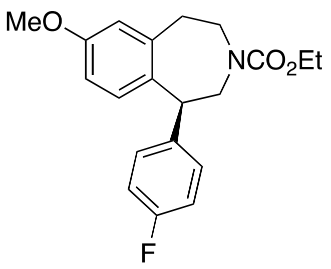

2j

Prepared according to the general procedure for the asymmetric hydrogenation on a 0.05 mmol scale (17.0 mg, 99% yield). **Appearance:** Colorless oil. **^{1}H NMR** (400 MHz, $CDCl_3$, mixture of rotamers) δ: 7.12 - 7.03 (m, 2H), 7.01 - 6.93 (m, 2H), 6.82 (d, *J* = 8.4 Hz, 1H), 6.72 (s, 1H), 6.67 (dd, *J* = 8.4, 2.7 Hz, 1H), 4.42 - 4.28 (m, 1H), 4.12 - 3.86 (m, 4H), 3.79 (s, 3H), 3.69 - 3.57 (m, 2H), 3.10 - 2.96 (m, 1H), 2.90 - 2.76 (m, 1H), 1.24 - 1.16 (m, 3H) ppm. **^{13}C NMR** (125 MHz, $CDCl_3$) δ: Major rotamer 161.5 (d, *J* = 244.7 Hz), 158.3, 156.1, 140.8, 138.1, 133.4, 131.8, 129.7 (d, *J* = 7.7 Hz), 116.3, 115.2 (d, *J* = 21.0 Hz), 111.8, 61.4, 55.2, 50.3, 50.0, 46.5, 36.2, 14.8 ppm. Minor rotamer 158.3, 156.3, 140.5, 138.3, 133.7, 111.6, 61.4, 50.8, 49.8, 46.3, 35.9, 14.7 ppm. **^{19}F NMR** (376 MHz, $CDCl_3$, mixture of rotamers) δ: -116.75. -116.91 ppm. **HRMS-ESI:** Found $[M+Na]^+$ = 366.1494; $C_{20}H_{22}FNO_3Na$ requires 366.1476. **$[\alpha]_D$**^{26} = +31 (c = 0.1, $CHCl_3$).

Ethyl (*S*)-7-methoxy-1-(4-(trifluoromethyl)phenyl)-1,2,4,5-tetrahydro-3*H*-benzo[*d*]azepine-3-carboxylate (2k)

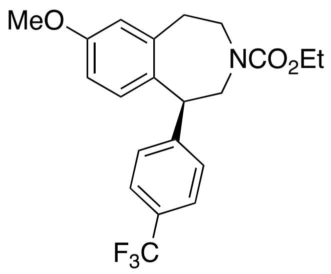

2k

Prepared according to the general procedure for the asymmetric hydrogenation on a 0.05 mmol scale (18.5 mg, 94% yield). **Appearance:** Colorless oil. **^{1}H NMR** (400 MHz, $CDCl_3$, mixture of rotamers) δ: 7.54 (d, *J* = 7.7 Hz, 2H), 7.22 (s, 2H), 6.86 - 6.79 (m, 1H), 6.74 (s, 1H), 6.69 (d, *J* = 8.6 Hz, 1H), 4.50 - 4.37 (m, 1H), 4.10 - 4.01 (m, 3H), 3.99 - 3.87 (m, 1H), 3.80 (s, 3H), 3.75 - 3.55 (m, 2H), 3.01 (q, *J* = 13.4 Hz, 1H), 2.92 - 2.75 (m, 1H), 1.22 - 1.15 (m, 3H) ppm. **^{13}C NMR** (125 MHz, $CDCl_3$) δ: Major rotamer 158.6, 156.1, 146.7, 141.0, 132.6, 132.0, 128.8 (q, *J* = 35.1 Hz), 128.7, 125.5 (q, *J* = 3.8 Hz), 123.8 (q, *J* = 271.3 Hz), 116.6, 111.9, 61.5, 55.4, 51.5, 50.1, 46.7, 36.3, 14.8 ppm. Minor rotamer 158.5, 156.4, 140.7, 133.0, 111.7, 61.6, 50.7, 49.5, 46.4, 35.9, 14.7 ppm. **^{19}F NMR** (376 MHz, $CDCl_3$, mixture of rotamers) δ: -62.40, -62.44 ppm. **HRMS-ESI:** Found $[M+Na]^+$ = 416.1459; $C_{21}H_{22}F_3NO_3Na$ requires 416.1455. **$[\alpha]_D$**^{27} = +27 (c = 0.1, $CHCl_3$).

Ethyl (*S*)-7-fluoro-1-phenyl-1,2,4,5-tetrahydro-3*H*-benzo[*d*]azepine-3-carboxylate (2l)

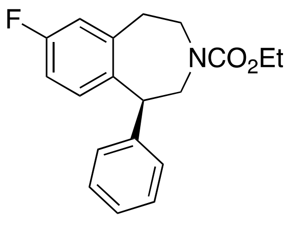

2l

Prepared according to the general procedure for the asymmetric hydrogenation on a 0.05 mmol scale (15.5 mg, 99% yield). **Appearance:** Colorless oil. **^{1}H NMR** (400 MHz, $CDCl_3$, mixture of rotamers) δ: 7.35 - 7.26 (m, 2H), 7.27 - 7.18 (m, 1H), 7.09 (d, *J* = 9.9 Hz, 2H), 6.91 - 6.82 (m, 2H), 6.80 (td, *J* = 8.3, 2.6 Hz, 1H), 4.42 (d, *J* = 30.9 Hz, 1H), 4.13 - 4.00 (m, 3H), 3.92 (dd, *J* = 19.8, 11.4 Hz, 1H), 3.78 - 3.65 (m, 1H), 3.61 (d, *J* = 15.2 Hz, 1H), 3.09 (t, *J* = 12.8 Hz, 1H), 2.87 (d, *J* = 17.4 Hz, 1H), 1.19 (q, *J* = 7.0 Hz, 3H) ppm. **^{13}C NMR** (125 MHz, $CDCl_3$) δ: Major rotamer 161.5 (d, *J* = 245.8 Hz), 156.3, 142.1, 141.6, 137.4 (d, *J* = 22.4 Hz), 132.4 (d, *J* = 7.7 Hz), 128.7, 128.2, 126.7, 117.3 (d, *J* = 20.3 Hz), 113.5 (d, *J* = 17.8 Hz), 61.5, 51.5, 50.2, 46.3, 35.8, 14.8 ppm. Minor rotamer 156.4, 142.3, 141.1, 116.9 (d, *J* = 20.7 Hz), 50.7, 49.6, 46.1, 35.4, 14.8 ppm. **^{19}F NMR** (376 MHz, $CDCl_3$, mixture of rotamers) δ: -116.91, -117.20 ppm. **HRMS-ESI:** Found $[M+Na]^+$ = 336.1373; $C_{19}H_{20}FNO_2Na$ requires 336.1370. **$[\alpha]_D$**^{26} = +44 (c = 0.1, $CHCl_3$).

Ethyl (*S*)-7-fluoro-1-(4-methoxyphenyl)-1,2,4,5-tetrahydro-3*H*-benzo[*d*]azepine-3-carboxylate (2m)

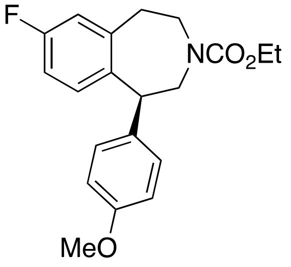

2m

Prepared according to the general procedure for the asymmetric hydrogenation on a 0.05 mmol scale (16.5 mg, 96% yield). **Appearance:** Yellow oil. **^{1}H NMR** (400 MHz, $CDCl_3$, mixture of rotamers) δ: 7.08 - 6.98 (m, 2H), 6.92 - 6.77 (m, 5H), 4.44 - 4.29 (m, 1H), 4.14 - 3.86 (m, 4H), 3.79 (s, 3H), 3.73 - 3.57 (m, 2H), 3.13 - 3.01 (m, 1H), 2.91 - 2.77 (m, 1H), 1.25 - 1.14 (m, 3H) ppm. **^{13}C NMR** (125 MHz, $CDCl_3$) δ: Major rotamer 161.4 (d, *J* = 245.1 Hz), 158.4, 156.3, 141.1, 137.8 (d, *J* = 18.5 Hz), 134.1, 132.3 (d, *J* = 7.2 Hz), 129.2, 117.2 (d, *J* = 21.6 Hz), 114.0, 113.4 (d, *J* = 20.7 Hz), 61.5, 55.3, 50.7, 49.9, 46.2, 35.8, 14.8 ppm. Minor rotamer 156.4, 141.5, 134.3, 116.9 (d, *J* = 21.0 Hz), 50.3, 49.7, 46.0, 35.4, 14.7 ppm. **^{19}F NMR** (376 MHz, $CDCl_3$, mixture of rotamers) δ: -117.05, -117.33 ppm. **HRMS-ESI:** Found $[M+Na]^+$ = 366.1480; $C_{20}H_{22}FNO_3Na$ requires 366.1476. $[\alpha]_D^{26}$ = +37 (c = 0.1, $CHCl_3$).

Methyl (*S*)-1-phenyl-1,2,4,5-tetrahydro-3*H*-benzo[*d*]azepine-3-carboxylate (2n)

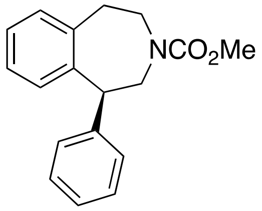

2n

Prepared according to the general procedure for the asymmetric hydrogenation on a 0.05 mmol scale (13.9 mg, 99% yield). **Appearance:** Colorless oil. **^{1}H NMR** (400 MHz, $CDCl_3$, mixture of rotamers) δ: 7.31 (dd, *J* = 8.2, 6.7 Hz, 2H), 7.23 (t, *J* = 7.3 Hz, 1H), 7.19 - 7.09 (m, 5H), 6.96 - 6.87 (m, 1H), 4.54 - 4.38 (m, 1H), 4.15 - 4.00 (m, 1H), 3.95 - 3.68 (m, 2H), 3.63 (s, 3H), 3.57 (ddd, *J* = 13.3, 6.0, 3.6 Hz, 1H), 3.19 - 3.09 (m, 1H), 2.97 - 2.82 (m, 1H) ppm. **^{13}C NMR** (125 MHz, $CDCl_3$) δ: Major rotamer 156.6, 142.2, 141.5, 139.3, 130.6, 130.6, 128.6, 128.3, 126.9, 126.6, 52.6, 52.1, 50.2, 46.7, 35.8 ppm. Minor rotamer 156.7, 142.4, 141.7, 138.9, 130.9, 130.3, 127.0, 126.8, 51.4, 49.6, 46.4, 35.4 ppm. **HRMS-ESI:** Found $[M+Na]^+$ = 304.1306; $C_{18}H_{19}NO_2Na$ requires 304.1308. $[\alpha]_D^{26}$ = +54 (c = 0.1, $CHCl_3$).

Ethyl (*S*)-1-phenyl-1,2,4,5-tetrahydro-3*H*-benzo[*d*]azepine-3-carboxylate (2o)

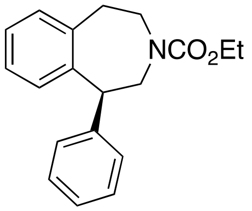

2o

Prepared according to the general procedure for the asymmetric hydrogenation on a 0.05 mmol scale (14.3 mg, 97% yield). **Appearance:** Colorless oil. **^{1}H NMR** (400 MHz, $CDCl_3$, mixture of rotamers) δ: 7.30 (t, *J* = 7.6 Hz, 2H), 7.22 (t, *J* = 7.3 Hz, 1H), 7.14 (dt, *J* = 11.9, 4.7 Hz, 5H), 6.92 (d, *J* = 7.5 Hz, 1H), 4.46 (d, *J* = 31.2 Hz, 1H), 4.06 (t, *J* = 7.2 Hz, 3H), 3.96 (d, *J* = 8.5 Hz, 1H), 3.72 (t, *J* = 11.0 Hz, 1H), 3.62 (d, *J* = 16.5 Hz, 1H), 3.20 - 3.06 (m, 1H), 2.91 (d, *J* = 19.1 Hz, 1H), 1.21 (d, *J* = 7.7 Hz, 3H) ppm. **^{13}C NMR** (125 MHz, $CDCl_3$) δ: Major rotamer 156.3, 142.3, 141.5, 139.4, 130.8, 130.7, 128.6, 128.3, 126.9, 126.6, 61.4, 52.2, 50.1, 46.6, 35.9, 14.8 ppm. Minor rotamer 156.4, 142.5, 141.8, 139.0, 130.9, 130.3, 127.0, 126.8, 51.4, 49.6, 46.4, 35.5, 14.7 ppm. **HRMS-ESI:** Found $[M+Na]^+$ = 318.1489; $C_{19}H_{21}NO_2Na$ requires 318.1465. $[\alpha]_D^{26}$ = +51 (c = 0.1, $CHCl_3$).

Benzyl (*S*)-1-phenyl-1,2,4,5-tetrahydro-3*H*-benzo[*d*]azepine-3-carboxylate (2p)

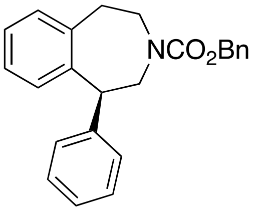

2p

Prepared according to the general procedure for the asymmetric hydrogenation on a 0.05 mmol scale (17.7 mg, 99% yield). **Appearance:** Yellow oil. **^{1}H NMR** (400 MHz, $CDCl_3$, mixture of rotamers) δ: 7.37 - 7.11 (m, 12H), 7.08 - 7.05 (m, 1H), 6.97 - 6.87 (m, 1H), 5.16 - 4.96 (m, 2H), 4.58 - 4.37 (m, 1H), 4.16 - 4.02 (m, 1H), 3.98 - 3.90 (m, 1H), 3.84 - 3.71 (m, 1H), 3.68 - 3.59 (m, 1H), 3.19 - 3.08 (m, 1H), 2.99 - 2.82 (m, 1H) ppm. **^{13}C NMR** (125 MHz, $CDCl_3$) δ: Major rotamer 156.0, 142.2, 141.5, 139.2, 136.9, 130.7, 130.7, 128.6, 128.6, 128.3, 127.9, 127.9, 127.0 (2C), 126.6, 67.1, 52.2, 50.3, 46.8, 35.8 ppm. Minor rotamer 156.2, 142.4, 141.6, 138.9, 137.0, 131.0, 130.4, 128.0, 127.1, 126.9, 67.1, 51.4, 49.8, 46.5, 35.4. ppm **HRMS-ESI:** Found $[M+Na]^+$ = 380.1635; $C_{24}H_{23}NO_2Na$ requires 380.1621. $[\alpha]_D^{26}$ = +47 (c = 0.1, $CHCl_3$).

Ethyl (*S*)-8,9-dimethoxy-1-phenyl-1,4,5,6-tetrahydrobenzo[*d*]azocine-3(2*H*)-carboxylate (2q)

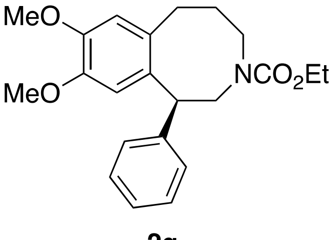

2q

Prepared according to the general procedure for the asymmetric hydrogenation on a 0.05 mmol scale (17.7 mg, 96% yield). **Appearance:** Colorless oil. **^{1}H NMR** (400 MHz, $CDCl_3$, mixture of rotamers) δ: 7.40 - 7.31 (m, 2H), 7.31 - 7.22 (m, 3H), 6.64 (d, *J* = 5.3 Hz, 1H), 6.29 (d, *J* = 9.6 Hz, 1H), 4.45 (td, *J* = 13.2, 12.3, 4.0 Hz, 1H), 4.07 (q, *J* = 7.1 Hz, 1H), 3.97 - 3.87 (m, 2H), 3.86 - 3.78 (m, 1H), 3.84 (s, 3H), 3.57 (s, 3H), 3.45 - 3.07 (m, 2H), 3.03 - 2.91 (m, 1H), 2.71 (tt, *J* = 13.0, 4.0 Hz, 1H), 2.14 - 1.74 (m, 2H), 1.12 (2t, *J* = 7.1 Hz, 3H) ppm. **^{13}C NMR** (125 MHz, $CDCl_3$) δ: Major rotamer 155.5, 147.4, 147.3, 142.5, 133.6, 132.7, 128.5, 128.5, 126.7, 112.2, 111.3, 61.1, 60.9, 55.8, 55.8, 53.5, 48.3, 47.0, 30.9, 14.7 ppm. Minor rotamer 156.4, 147.4, 147.2, 142.1, 134.3, 133.5, 126.6, 112.1, 111.6, 55.9, 55.7, 53.9, 29.3, 14.8 ppm. **HRMS-ESI:** Found $[M+Na]^+$ = 392.1833; $C_{22}H_{27}NO_4Na$ requires 392.1832. **$[\alpha]_D^{27}$** = -98 (c = 0.1, $CHCl_3$).

Benzyl (*S*)-6-chloro-7,8-dimethoxy-1-(4-methoxyphenyl)-1,2,4,5-tetrahydro-3*H*-benzo[*d*]azepine-3-carboxylate (2s)

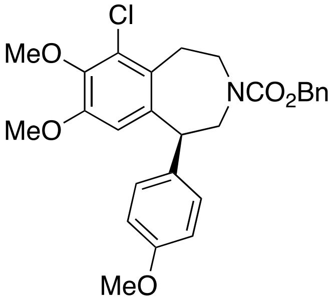

2s

Prepared according to the general procedure for the asymmetric hydrogenation on a 0.05 mmol scale (23.3 mg, 97% yield). **Appearance:** Colorless oil. **^{1}H NMR** (400 MHz, $CDCl_3$, mixture of rotamers) δ: 7.37 - 7.27 (m, 4H), 7.21 - 7.17 (m, 1H), 7.09 - 7.02 (m, 1H), 6.99 - 6.93 (m, 1H), 6.88 - 6.83 (m, 1H), 6.82 - 6.77 (m, 1H), 6.45 - 6.35 (m, 1H), 5.16 - 5.02 (m, 2H), 4.53 - 4.32 (m, 1H), 4.13 - 4.02 (m, 1H), 3.85 (s, 3H), 3.79 (s, 3H), 3.83 - 3.64 (m, 2H), 3.71 - 3.68 (m, 3H), 3.61 - 3.51 (m, 1H), 3.36 - 3.23 (m, 1H), 3.15 - 3.03 (m, 1H) ppm. **^{13}C NMR** (100 MHz, $CDCl_3$) δ: Major rotamer 158.5, 156.4, 151.9, 144.0, 138.3, 136.9, 134.1, 129.1, 128.7, 128.6, 128.1, 128.0, 127.5, 114.2, 113.6, 67.2, 60.6, 56.0, 55.4, 50.6, 49.5, 45.5, 29.1 ppm. Minor rotamer 156.1, 151.8, 144.2, 138.2, 137.0, 134.0, 129.1, 113.5, 51.4, 49.8, 45.9, 29.2 ppm. **HRMS-ESI:** Found $[M+Na]^+$ = 504.1549, 506.1526; $C_{27}H_{28}ClNO_5Na$ requires 504.1548, 506.1521. **$[\alpha]_D^{27}$** = +10 (c = 0.1, $CHCl_3$).

Ethyl (*S*)-8,9-dimethoxy-1-methyl-1,2,4,5-tetrahydro-3*H*-benzo[*d*]azepine-3-carboxylate (4a)

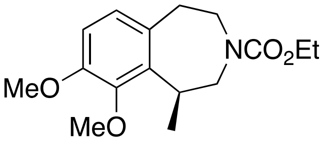

4a

Prepared according to the general procedure for the asymmetric hydrogenation on a 0.05 mmol scale (14.5 mg, 99% yield). **Appearance:** Yellow oil. **^{1}H NMR** (500 MHz, $CDCl_3$, mixture of rotamers) δ: 6.77 (d, *J* = 8.3 Hz, 1H), 6.66 (d, *J* = 8.3 Hz, 1H), 4.28 - 4.03 (m, 4H), 3.86 - 3.83 (m, 1H), 3.82 (s, 3H), 3.77 (s, 3H), 3.24 - 3.10 (m, 2H), 3.03 - 2.92 (m, 1H), 2.70 - 2.64 (m, 1H), 1.28 - 1.24 (m, 3H), 1.23 - 1.19 (m, 3H) ppm. **^{13}C NMR** (125 MHz, $CDCl_3$) δ: Major rotamer 156.5, 151.4, 146.6, 138.8, 132.5, 126.0, 109.8, 61.3, 61.0, 55.7, 50.5, 47.3, 36.0, 32.7, 16.9, 14.9 ppm. Minor rotamer 156.3, 151.5, 146.7, 139.1, 125.9, 109.7, 61.3, 61.1, 36.3, 32.8, 17.0, 14.8 ppm. **HRMS-ESI:** Found $[M+Na]^+$ = 316.1524; $C_{16}H_{23}NO_4Na$ requires 316.1519. **$[\alpha]_D^{25}$** = -33 (c = 0.1, $CHCl_3$).

Ethyl (*S*)-1-butyl-7,8-dimethoxy-1,2,4,5-tetrahydro-3*H*-benzo[*d*]azepine-3-carboxylate (4b)

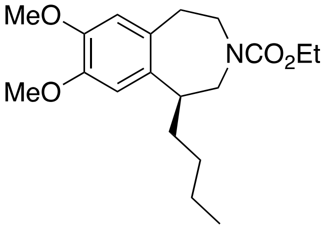

4b

Prepared according to the general procedure for the asymmetric hydrogenation on a 0.05 mmol scale (16.2 mg, 97% yield). **Appearance:** Colorless oil. **^{1}H NMR** (500 MHz, $CDCl_3$, mixture of rotamers) δ: 6.58 - 6.54 (m, 2H), 4.16 - 4.06 (m, 3H), 4.00 - 3.91 (m, 1H), 3.81 (s, 3H), 3.80 (s, 3H), 3.31 - 3.25 (m, 1H), 3.13 - 2.98 (m, 2H), 2.82 - 2.57 (m, 2H), 1.66 - 1.51 (m, 2H), 1.34 - 1.10 (m, 7H), 0.85 - 0.79 (m, 3H) ppm. **^{13}C NMR** (125 MHz, $CDCl_3$) δ: Major rotamer 156.3, 146.7, 146.6, 135.9, 131.7, 114.3, 114.0, 61.2, 56.0, 55.9, 49.8, 47.8, 47.1, 36.1, 30.9, 30.1, 22.8, 14.7, 14.0 ppm. Minor rotamer 156.2, 131.3, 114.2, 49.8, 47.4, 47.0, 31.2, 30.0 ppm. **HRMS-ESI:** Found $[M+Na]^+$ = 358.1992; $C_{19}H_{29}NO_4Na$ requires 358.1989. **$[\alpha]_D^{24}$** = -2 (c = 0.1, $CHCl_3$).

Ethyl (*S*)-1-isobutyl-7,8-dimethoxy-1,2,4,5-tetrahydro-3*H*-benzo[*d*]azepine-3-carboxylate (4c)

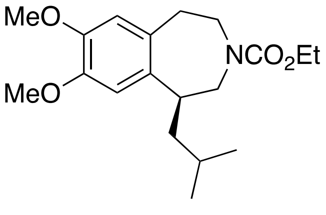

4c

Prepared according to the general procedure for the asymmetric hydrogenation on a 0.05 mmol scale (15.7 mg, 94% yield). **Appearance:** Colorless oil. **^{1}H NMR** (400 MHz, $CDCl_3$, mixture of rotamers) δ: 6.63 - 6.58 (m, 2H), 4.21 - 4.10 (m, 2H), 4.07 - 3.89 (m, 2H), 3.85 (s, 3H), 3.85 (s, 3H), 3.34 - 3.28 (m, 1H), 3.13 - 3.05 (m, 2H), 2.97 - 2.82 (m, 1H), 2.68 (s, 1H), 1.55 - 1.47 (m, 3H), 1.30 - 1.23 (m, 3H), 0.96 - 0.83 (m, 6H). **^{13}C NMR** (100 MHz, $CDCl_3$) δ: Major rotamer 156.4, 146.8, 136.1, 132.0, 125.4, 114.4, 113.8, 61.4, 56.2, 56.0, 50.2, 47.3, 45.3, 40.6, 36.3, 25.5, 23.1, 14.9 ppm. Minor rotamer 156.3, 136.2, 131.5, 114.4, 113.9, 50.1, 47.1, 45.1, 40.9, 22.9 ppm. **HRMS-ESI:** Found $[M+Na]^+$ = 358.1989; $C_{19}H_{29}NO_4Na$ requires 358.1989. **$[\alpha]_D^{24}$** = +3 (c = 0.1, $CHCl_3$).

Ethyl (*S*)-1-isopropyl-7,8-dimethoxy-1,2,4,5-tetrahydro-3*H*-benzo[*d*]azepine-3-carboxylate (4d)

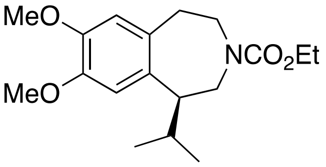

4d

Prepared according to the general procedure for the asymmetric hydrogenation on a 0.05 mmol scale (15.4 mg, 96% yield). **Appearance:** Colorless oil. **^{1}H NMR** (500 MHz, $CDCl_3$, mixture of rotamers) δ: 6.55 (s, 1H), 6.53 (s, 1H), 4.18 - 3.97 (m, 4H), 3.82 (s, 3H), 3.82 (s, 3H), 3.34 - 3.25 (m, 1H), 3.10 - 2.98 (m, 2H), 2.62 - 2.39 (m, 2H), 2.06 - 1.96 (m, 1H), 1.27 - 1.17 (m, 3H), 1.08 - 1.02 (m, 3H), 0.71 - 0.60 (m, 3H) ppm. **^{13}C NMR** (125 MHz, $CDCl_3$) δ: Major rotamer 156.5, 146.8, 146.6, 135.0, 131.5, 115.2, 113.8, 61.3, 55.9, 55.0, 47.5, 47.1, 35.6, 27.9, 22.0, 21.4, 14.7 ppm. Minor rotamer 156.3, 146.8, 147.7, 131.0, 56.0, 54.5, 47.2, 28.7, 21.3 ppm. **HRMS-ESI:** Found $[M+Na]^+$ = 344.1838; $C_{18}H_{27}NO_4Na$ requires 344.1832. **$[\alpha]_D^{24}$** = +2 (c = 0.1, $CHCl_3$).

Ethyl (*S*)-1-benzyl-7,8-dimethoxy-1,2,4,5-tetrahydro-3*H*-benzo[*d*]azepine-3-carboxylate (4e)

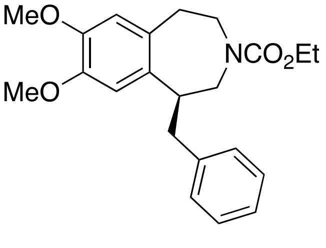

4e

Prepared according to the general procedure for the asymmetric hydrogenation on a 0.05 mmol scale (17.0 mg, 92% yield). **Appearance:** White solid. **^{1}H NMR** (500 MHz, $CDCl_3$, mixture of rotamers) δ: 7.20 - 7.09 (m, 3H), 6.96 - 6.89 (m, 2H), 6.62 - 6.57 (m, 1H), 6.17 - 6.08 (m, 1H), 4.14 - 3.96 (m, 4H), 3.83 (s, 3H), 3.63 - 3.53 (m, 3H), 3.49 - 3.41 (m, 1H), 3.28 - 3.07 (m, 3H), 3.04 - 2.82 (m, 2H), 2.79 - 2.66 (m, 1H), 1.26 - 1.17 (m, 3H). **^{13}C NMR** (125 MHz, $CDCl_3$) δ: Major rotamer 156.4, 146.8, 146.5, 140.2, 134.0, 131.5, 129.2, 128.1, 126.0, 114.5, 114.0, 61.4, 55.9, 55.9, 55.8, 49.7, 47.2, 38.4, 36.1, 14.8 ppm. Minor rotamer 146.7, 133.9, 130.8, 129.3, 114.9, 113.9, 49.8, 47.1, 38.8, 36.0 ppm. **HRMS-ESI:** Found $[M+Na]^+$ = 392.1825; $C_{22}H_{27}NO_4Na$ requires 392.1832. **$[\alpha]_D^{25}$** = +49 (c = 0.1, $CHCl_3$). **m.p.** 134 °C.

Synthesis of benzazepine 2t

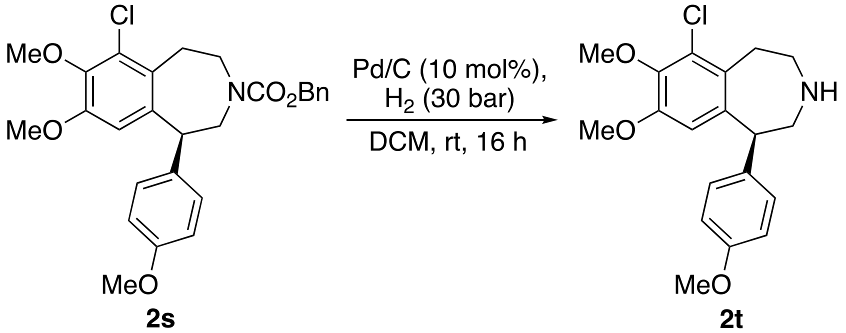

(*S*)-6-chloro-7,8-dimethoxy-1-(4-methoxyphenyl)-2,3,4,5-tetrahydro-1*H*-benzo[*d*]azepine (2t)

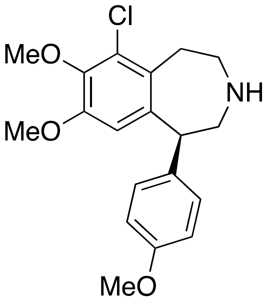

2t

An oven-dried vial was charged with tetrahydro-3-benzazepine **2s** (24.1 mg, 0.05 mmol, 1.0 equiv.) and Pd/C (10 wt.%, 5.3 mg, 10.0 mol%). Freshly distilled DCM (1 mL) and a magnetic stirring bar were added and the vial was placed in a high-pressure hydrogenation apparatus. The reactor was purged three times with Ar, purged three times with H_2 and then pressurized with H_2 (30 bar). The reaction was stirred at room temperature for 16 h before the H_2 pressure was released. Then, the mixture was passed through a short plug of celite and the solvent was removed under reduced pressure. The residue was purified by flash chromatography (DCM/MeOH, 90:10) on silica gel to give the tetrahydro-3-benzazepine **2t** (15.1 mg, 87% yield). **Appearance:** Brown oil. **^{1}H NMR** (400 MHz, $CDCl_3$) δ: 7.06 (d, *J* = 8.7 Hz, 2H), 6.90 (d, *J* = 8.7 Hz, 2H), 6.18 (s, 1H), 4.64 (d, *J* = 8.9 Hz, 1H), 3.81 (s, 3H), 3.80 (s, 3H), 3.65 (d, *J* = 12.7 Hz, 1H), 3.58 (s, 3H), 3.48 - 3.28 (m, 4H), 2.94 - 2.84 (m, 1H) ppm. **^{13}C NMR** (100 MHz, $CDCl_3$) δ: 158.8, 151.6, 144.1, 140.0, 132.4, 129.5, 129.4, 128.9, 114.5, 112.1, 60.6, 55.9, 55.4, 51.1, 47.4, 45.6, 28.8 ppm. **HRMS-ESI:** Found $[M+H]^+$ = 348.1363, 350.1334; $C_{19}H_{23}ClNO_3$ requires 348.1361, 350.1333. $[\alpha]_D^{26}$ = -8 (c = 0.1, $CHCl_3$).

NMR spectra – Ene-carbamates and intermediates

^{1}H NMR (300 MHz, $CDCl_3$)

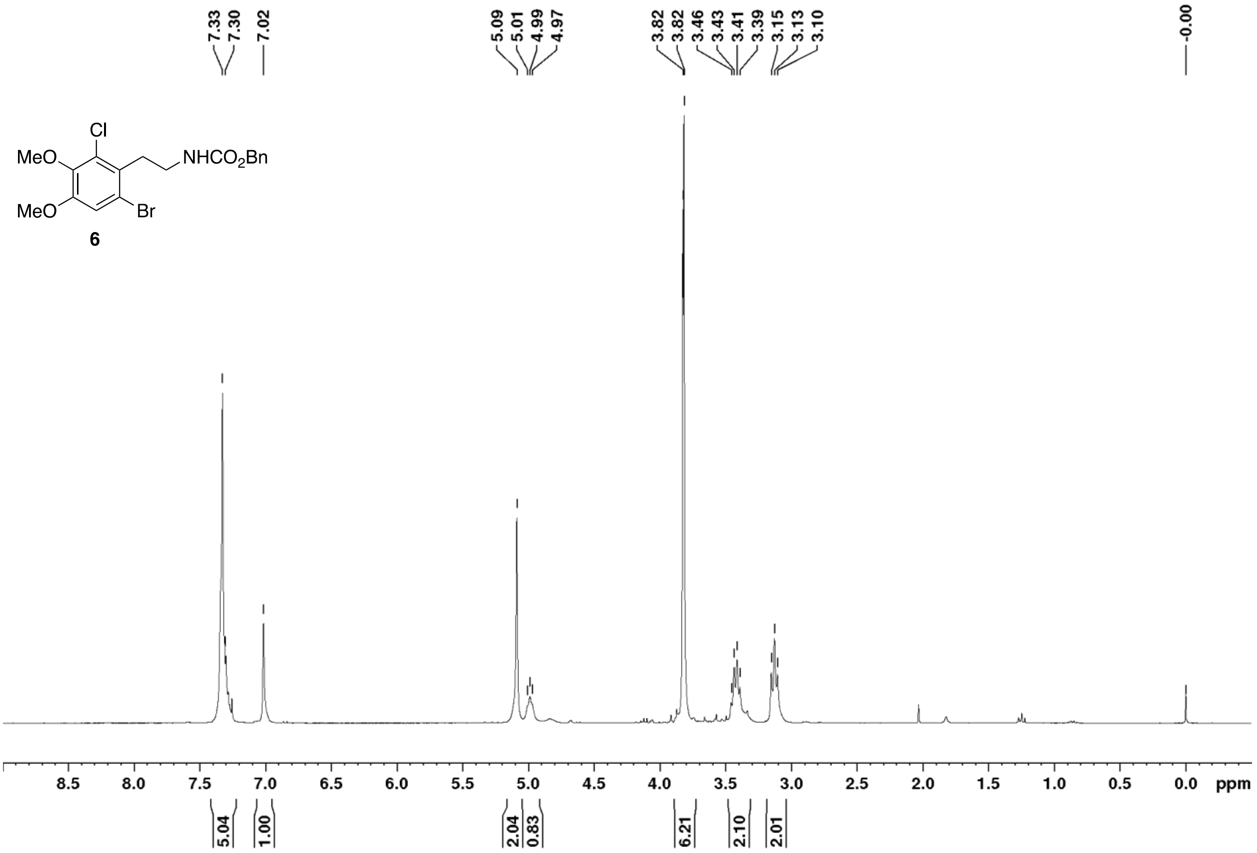

^{13}C NMR (75 MHz, $CDCl_3$)

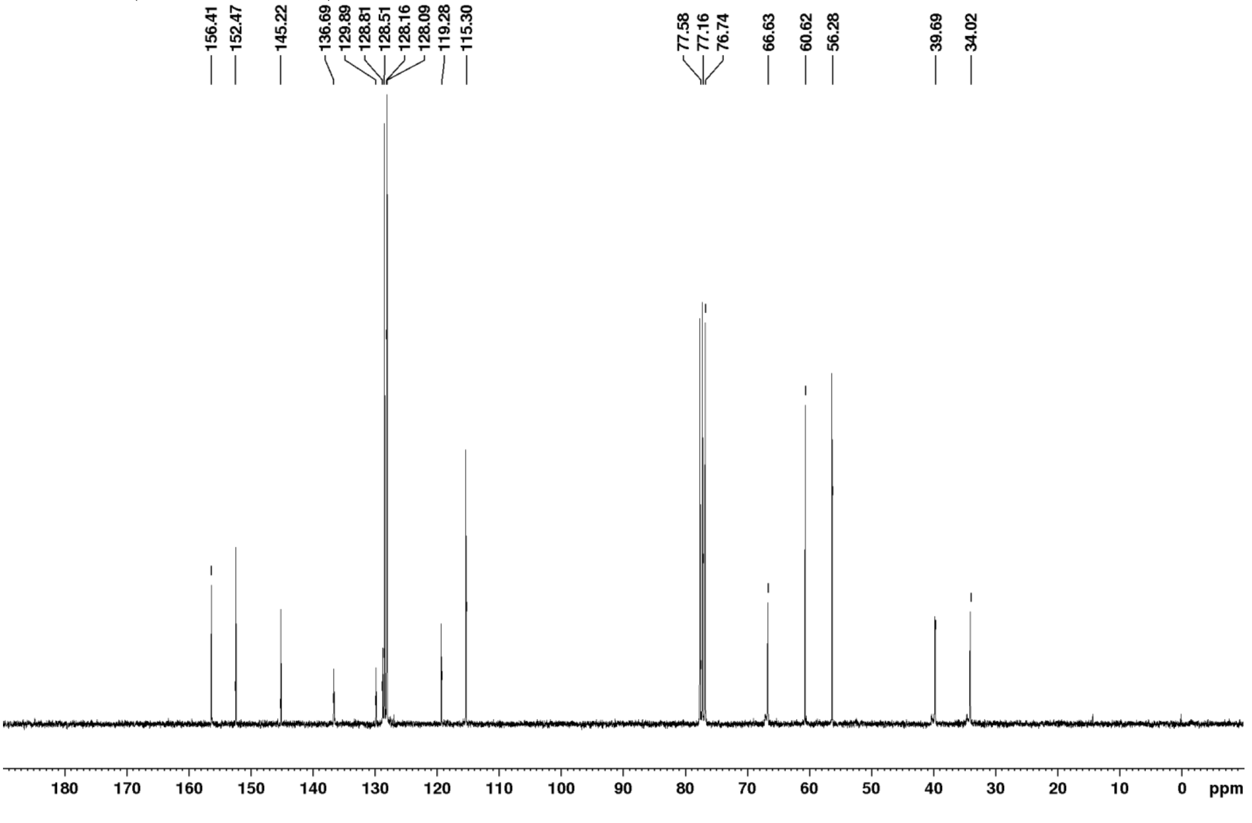

^{1}H NMR (400 MHz, $CDCl_3$)

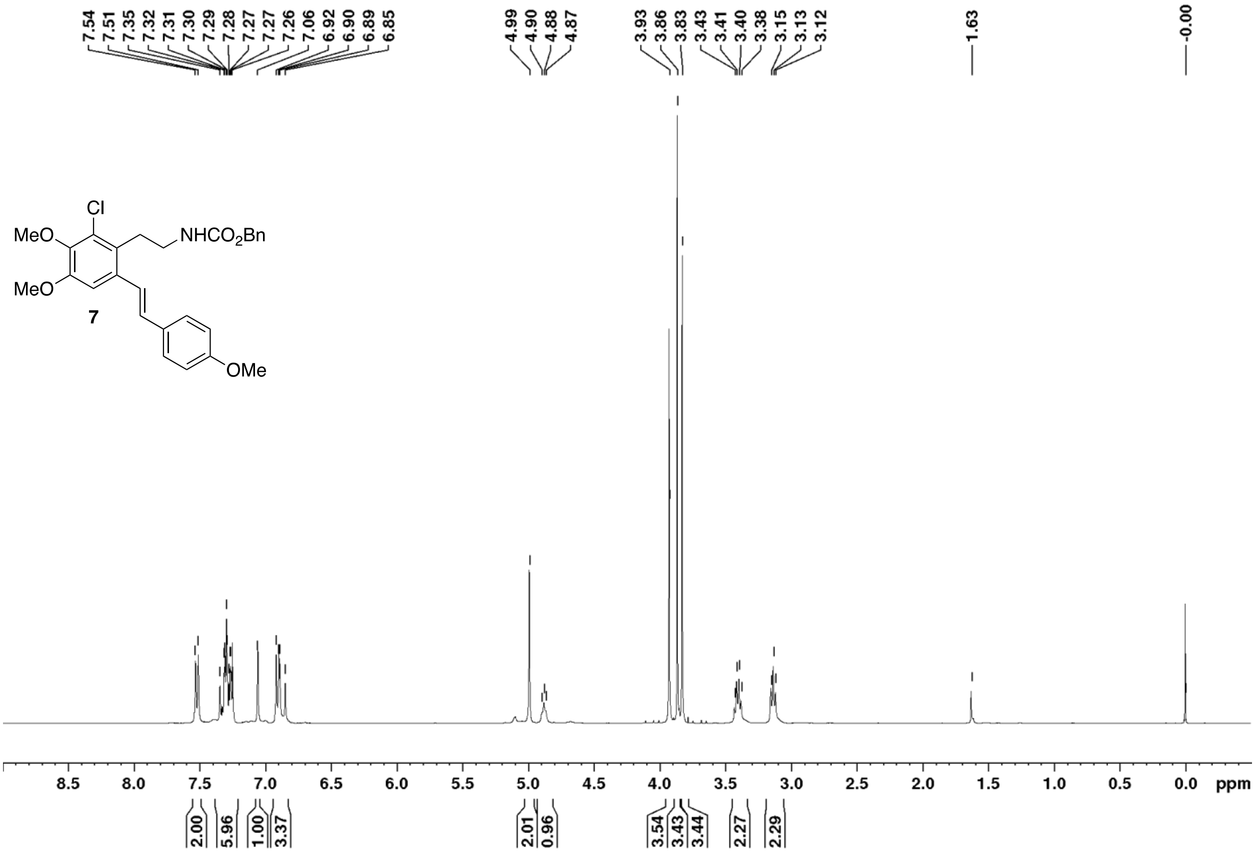

^{13}C NMR (100 MHz, $CDCl_3$)

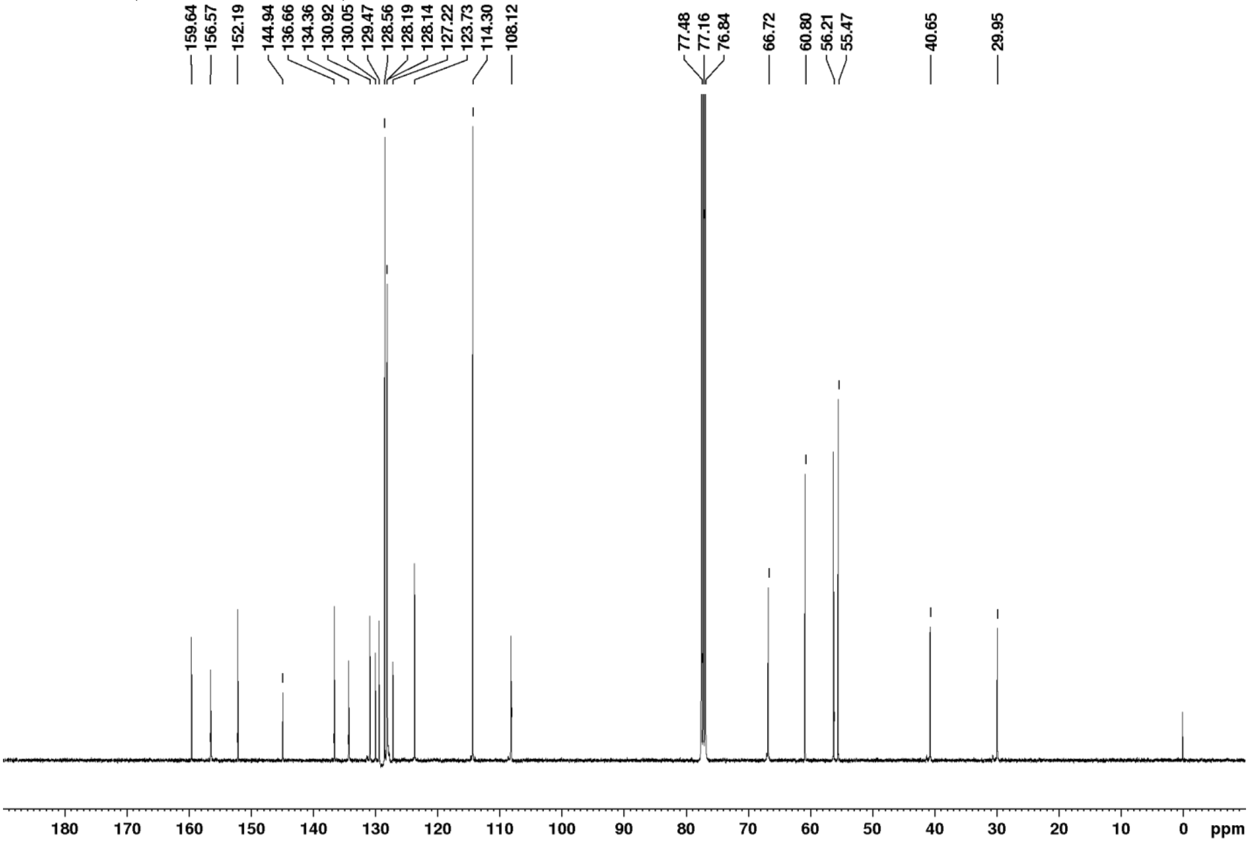

^{1}H NMR (300 MHz, $CDCl_3$)

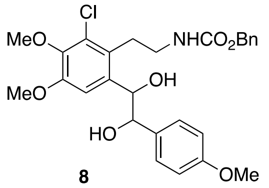

8

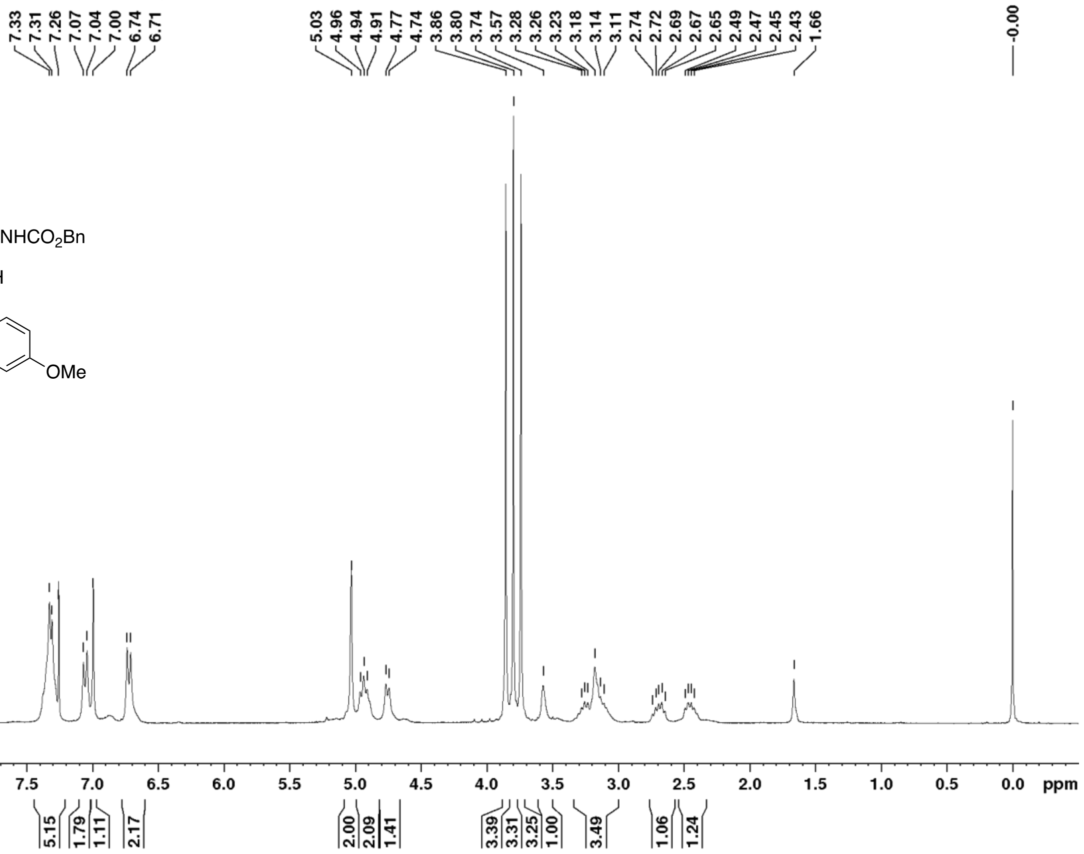

^{13}C NMR (75 MHz, $CDCl_3$)

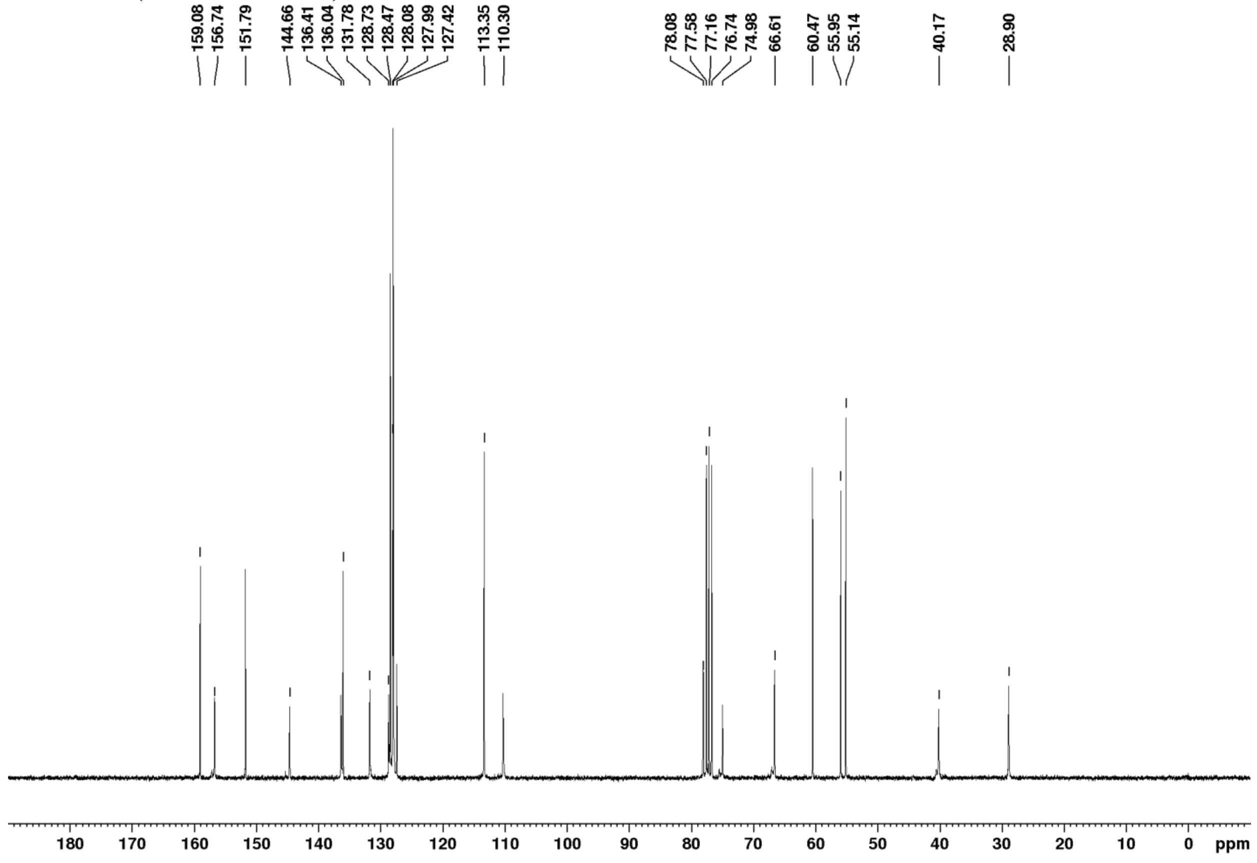

^{1}H NMR (400 MHz, $CDCl_3$)

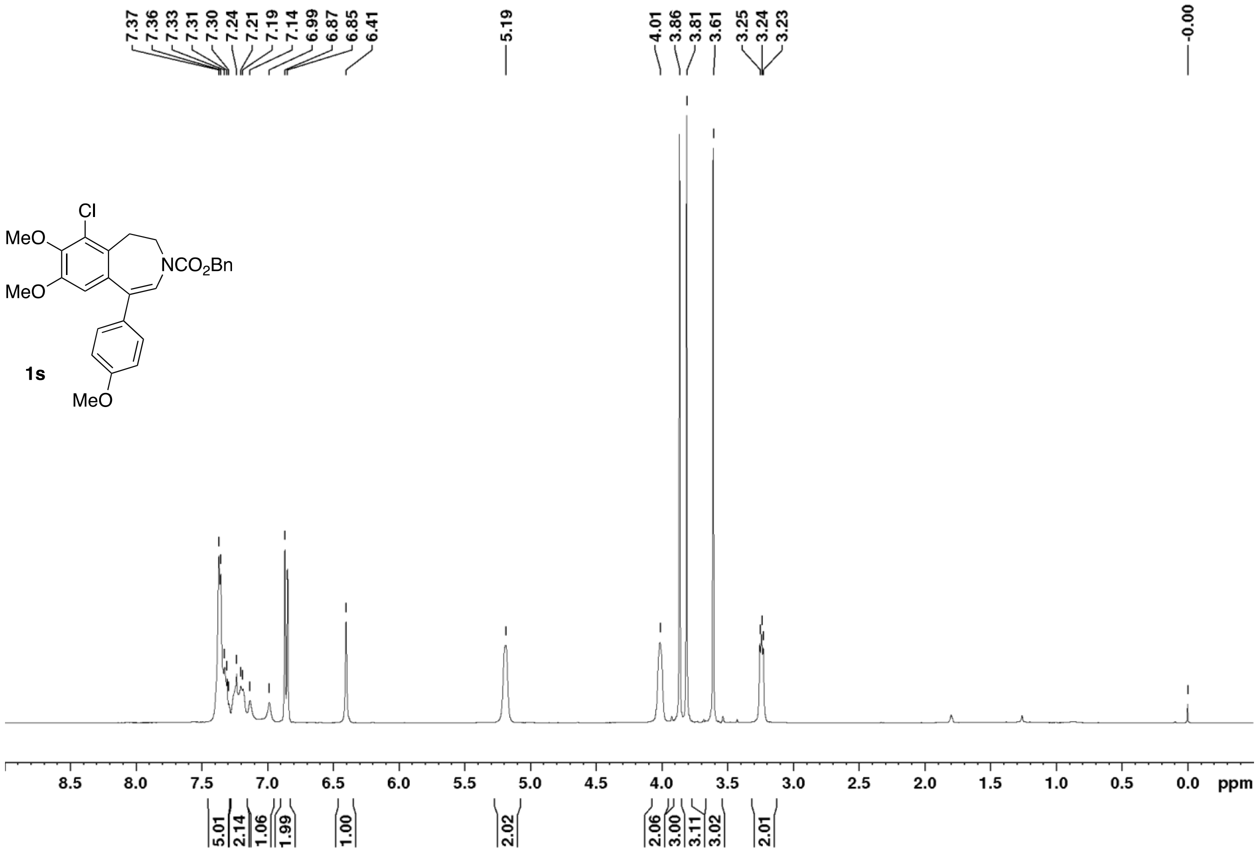

^{13}C NMR (100 MHz, $CDCl_3$)

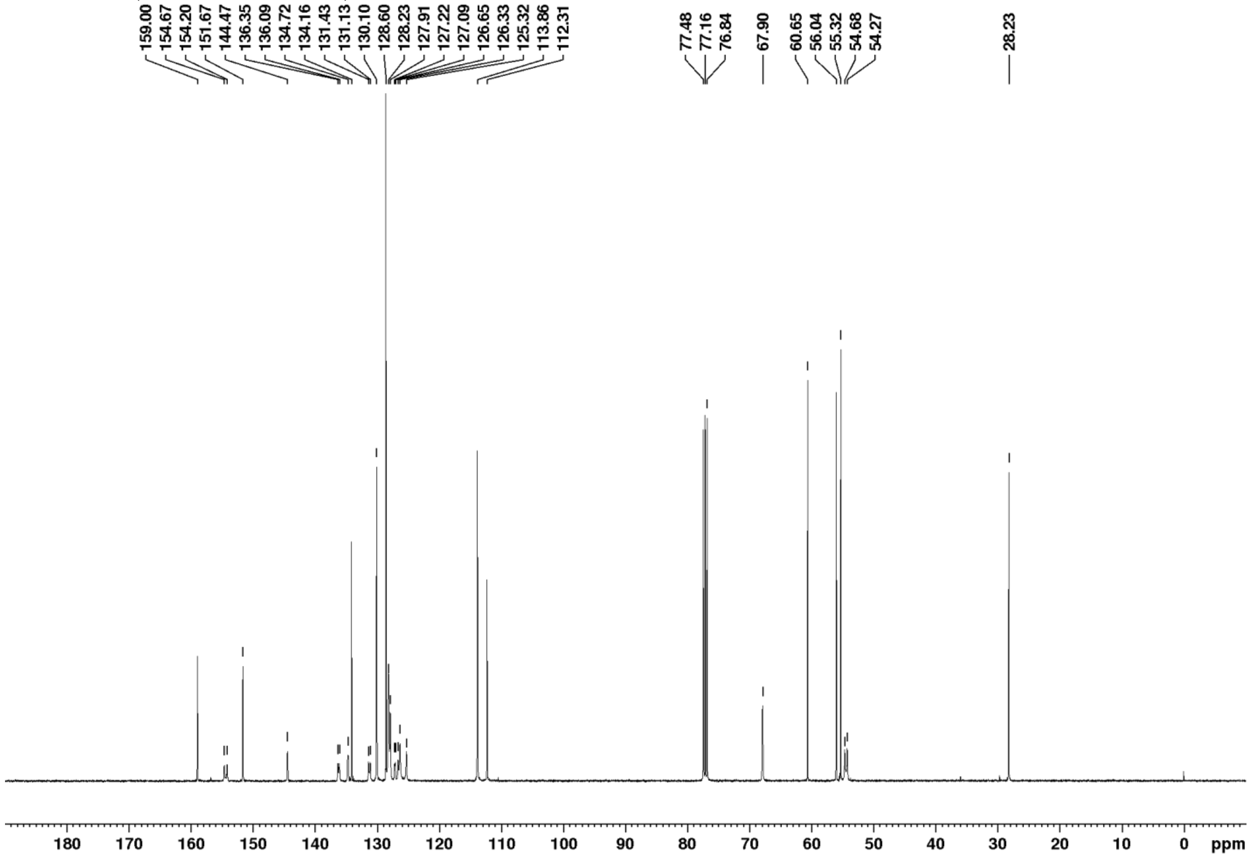

^{1}H NMR (400 MHz, $CDCl_3$)

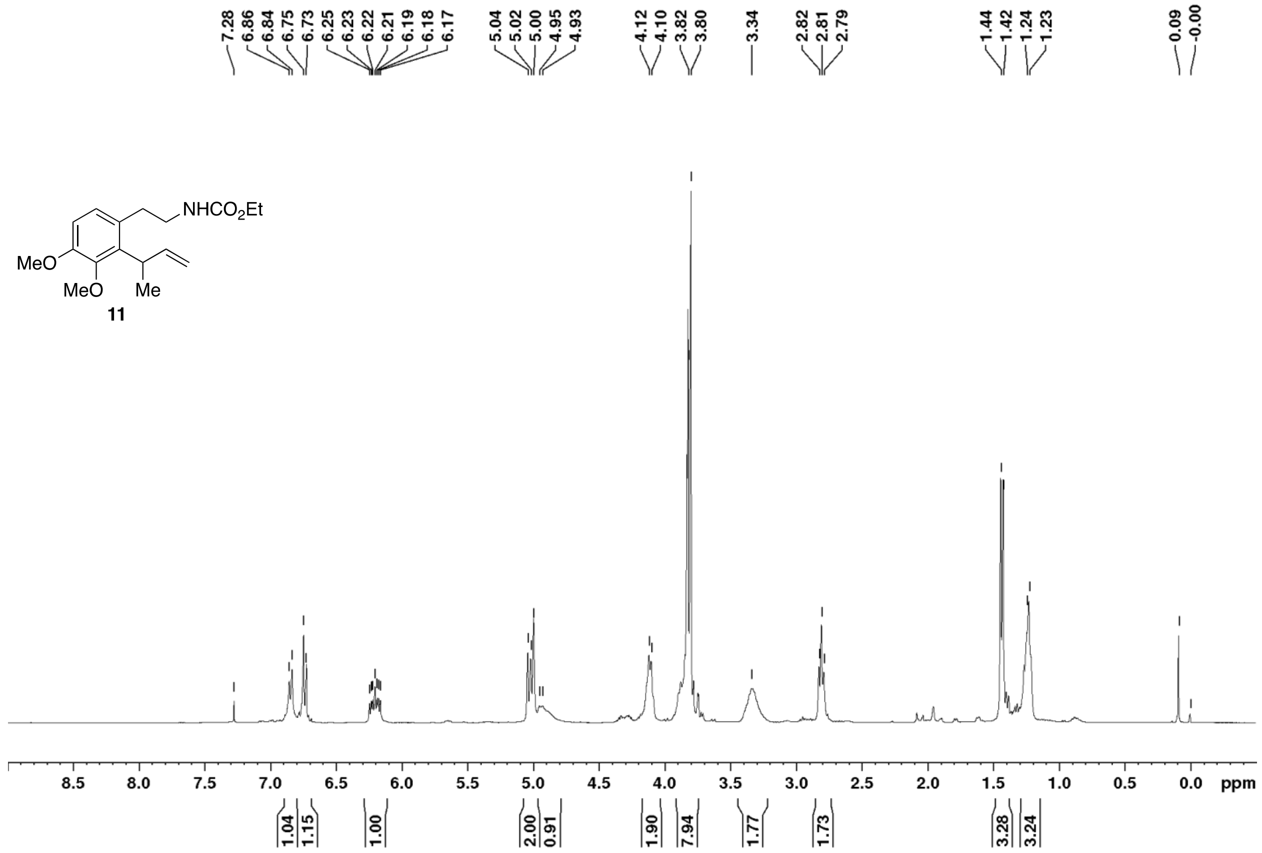

^{13}C NMR (100 MHz, $CDCl_3$)

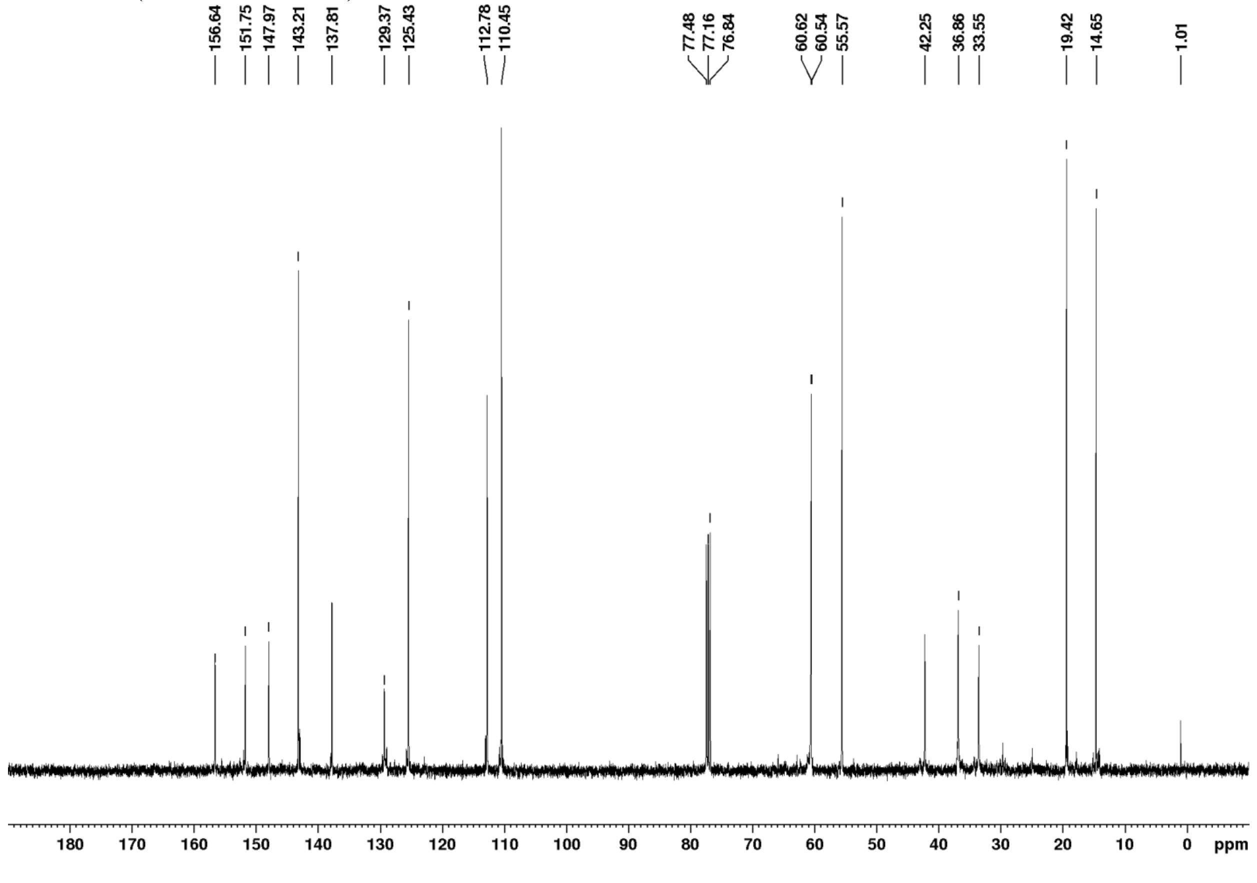

^{1}H NMR (400 MHz, $CDCl_3$)

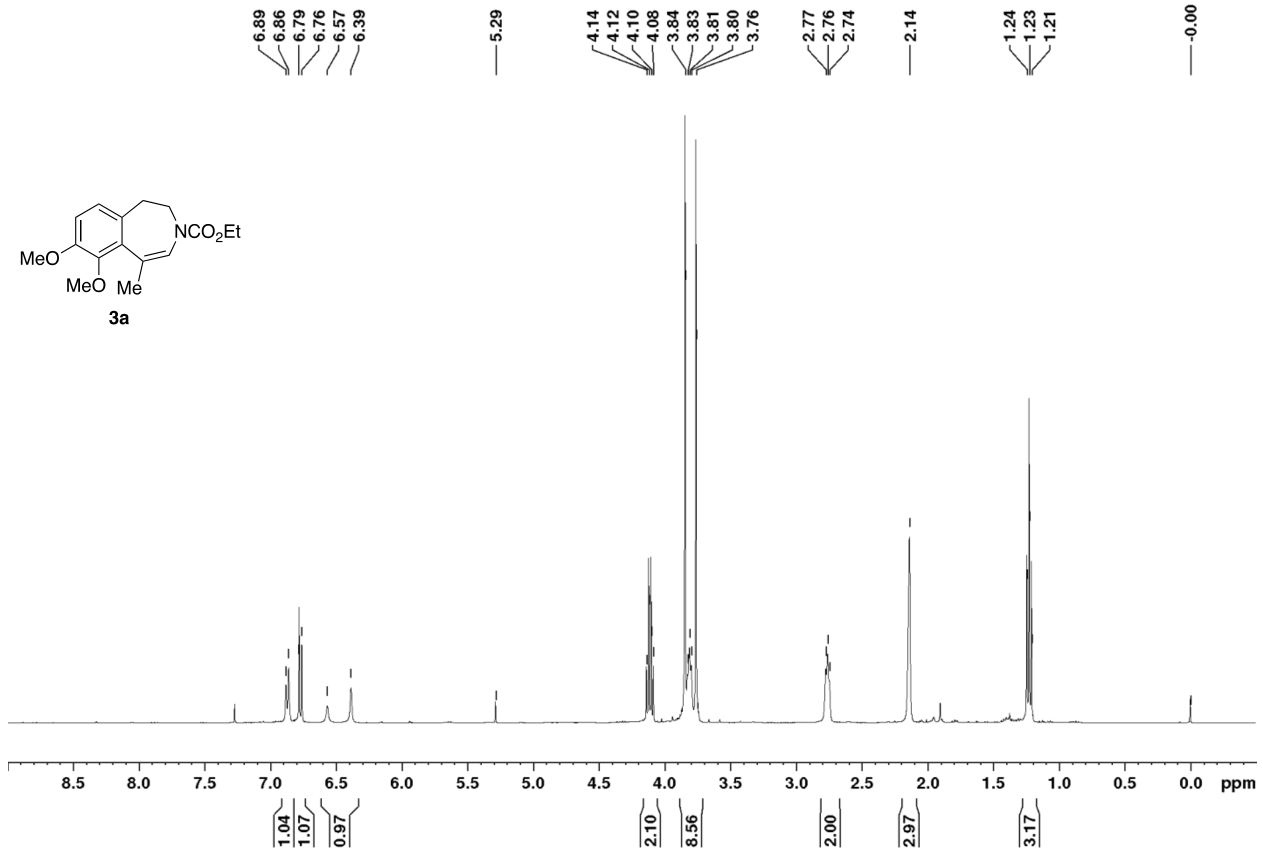

^{13}C NMR (100 MHz, $CDCl_3$)

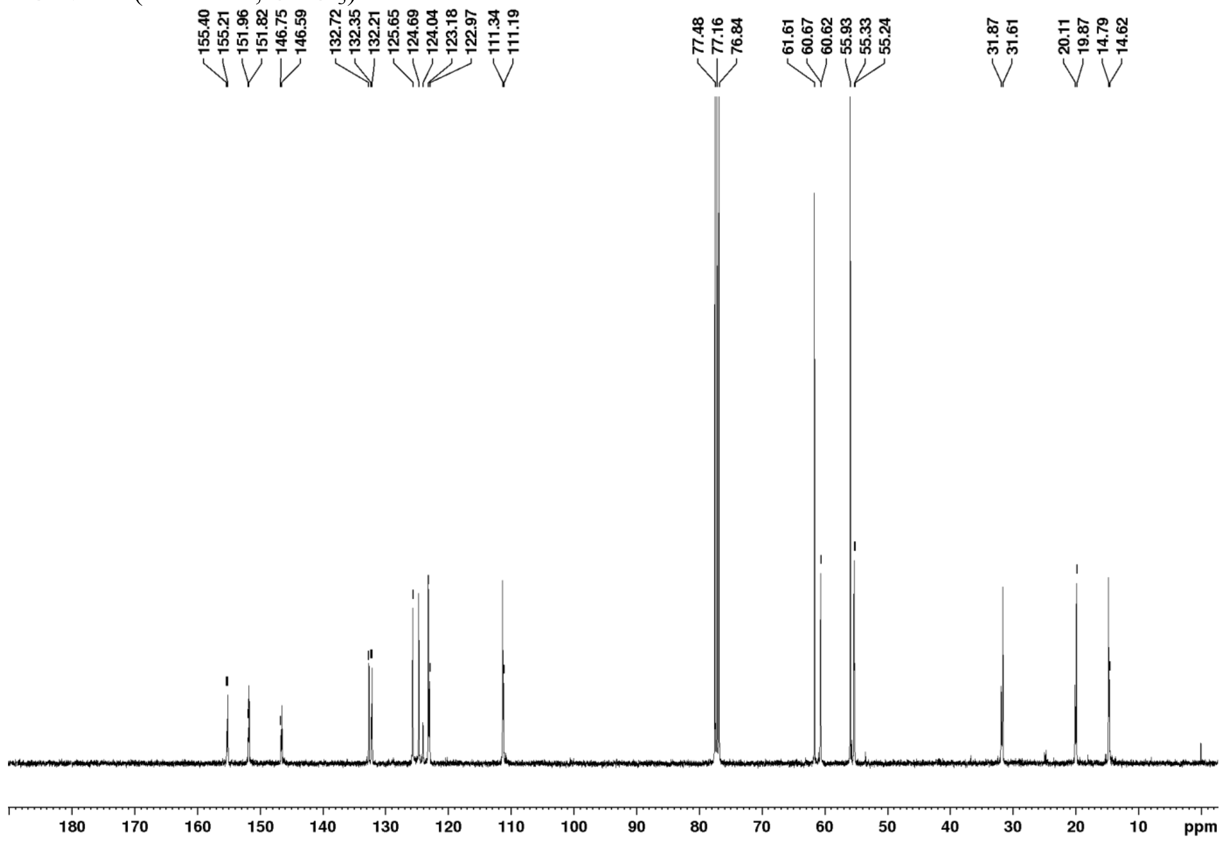

^{1}H NMR (400 MHz, $CDCl_3$)

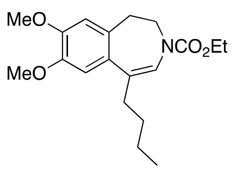

3b

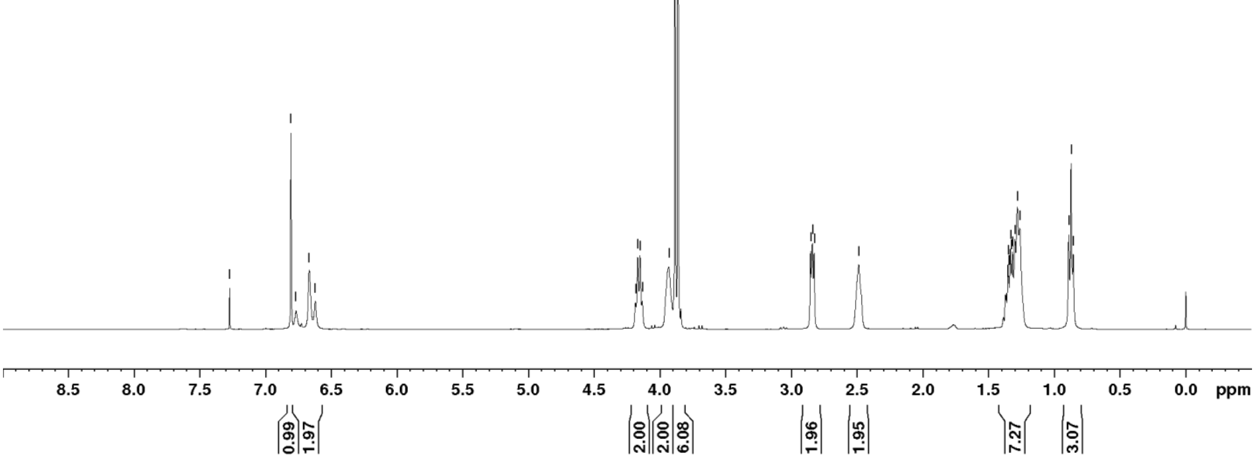

^{13}C NMR (100 MHz, $CDCl_3$)

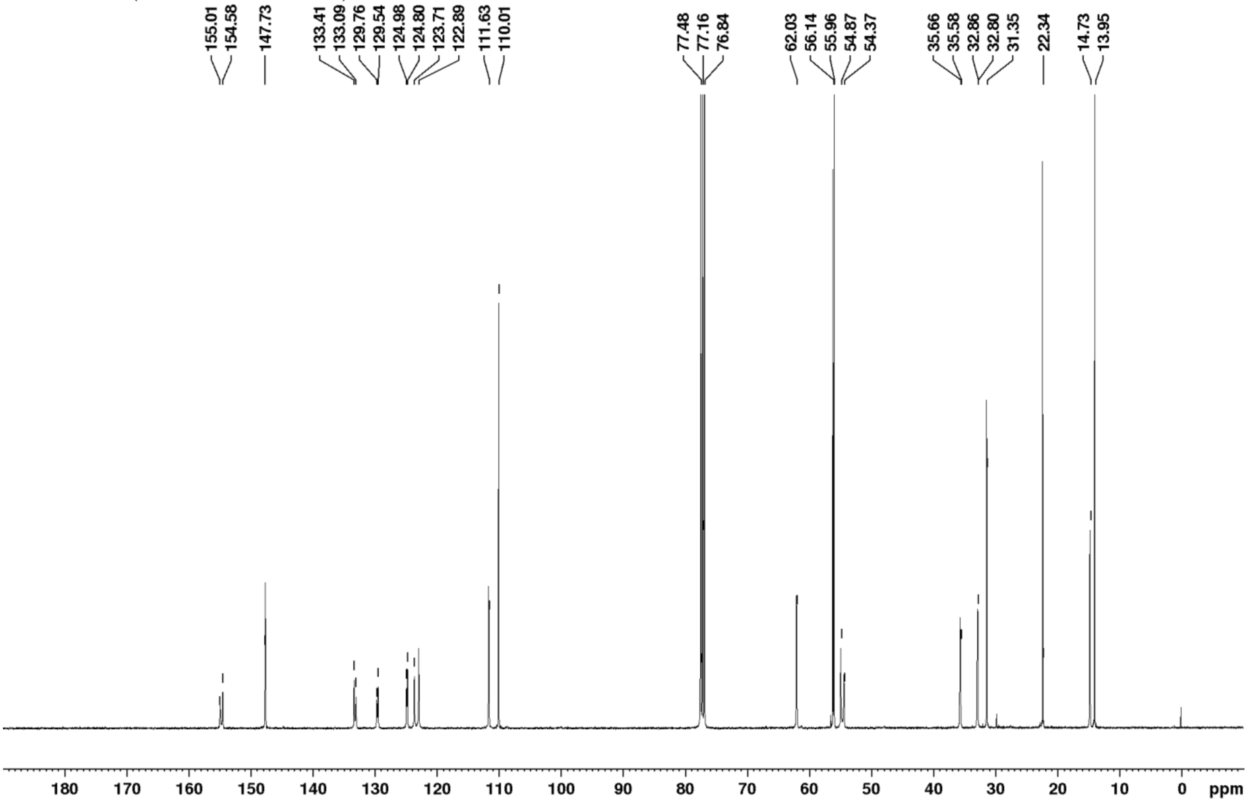

^{1}**H NMR** (300 MHz, $CDCl_3$)

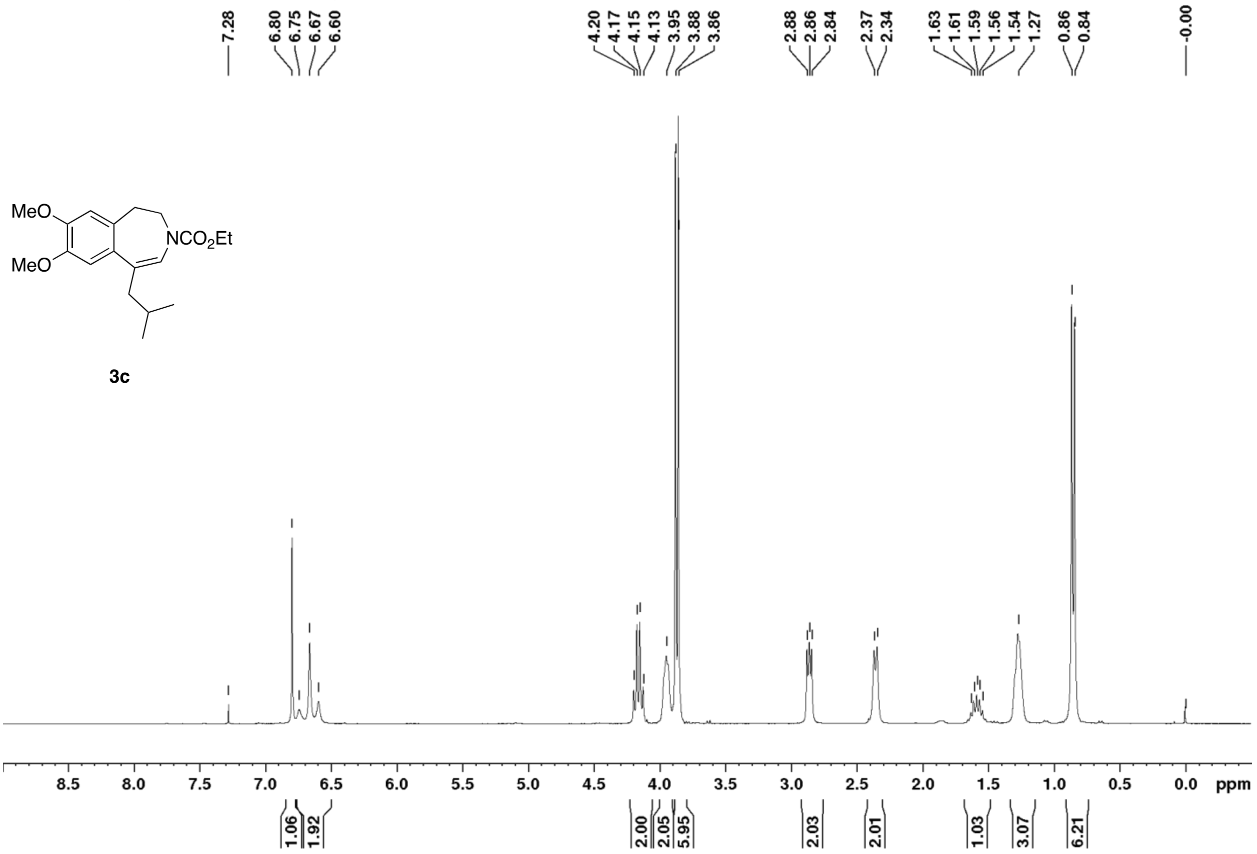

^{13}**C NMR** (75 MHz, $CDCl_3$)

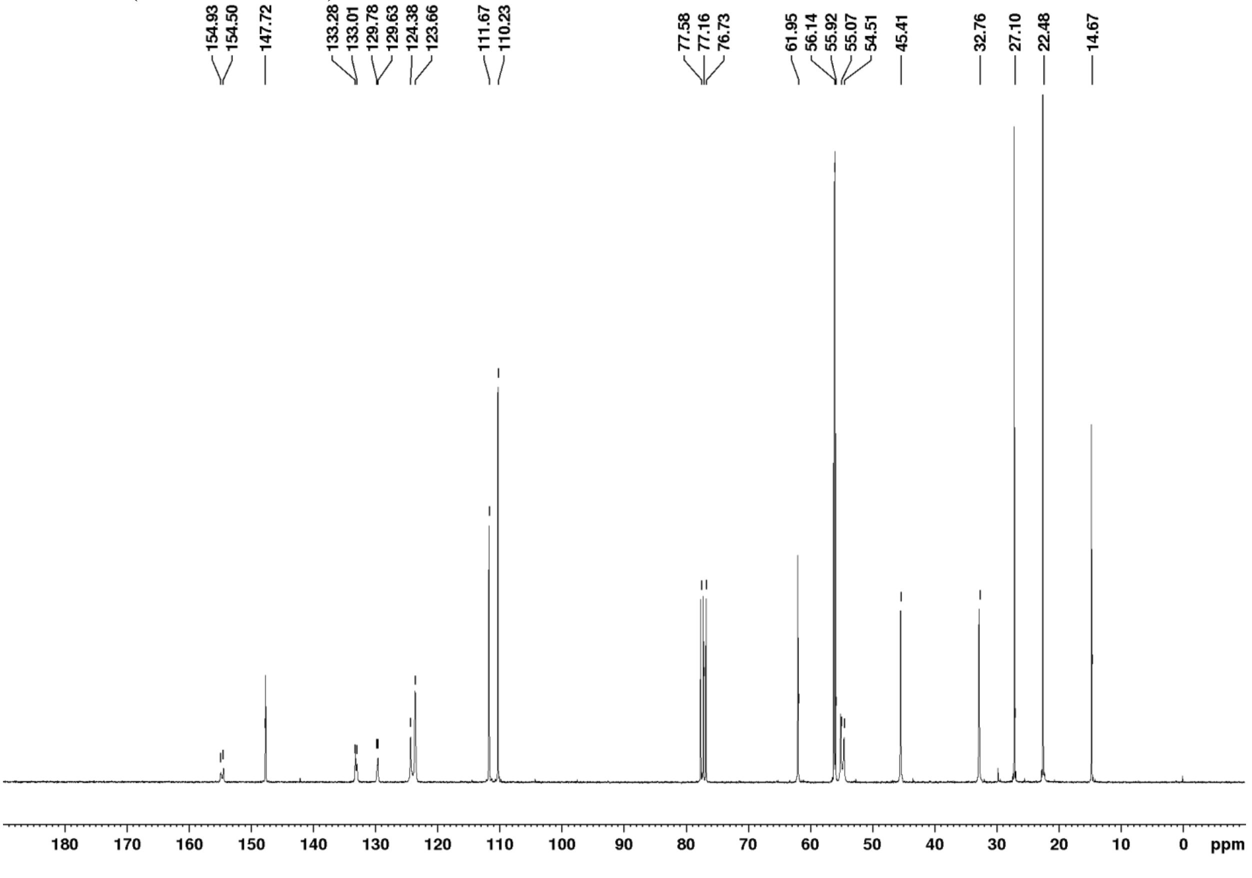

^{1}H NMR (400 MHz, $CDCl_3$)

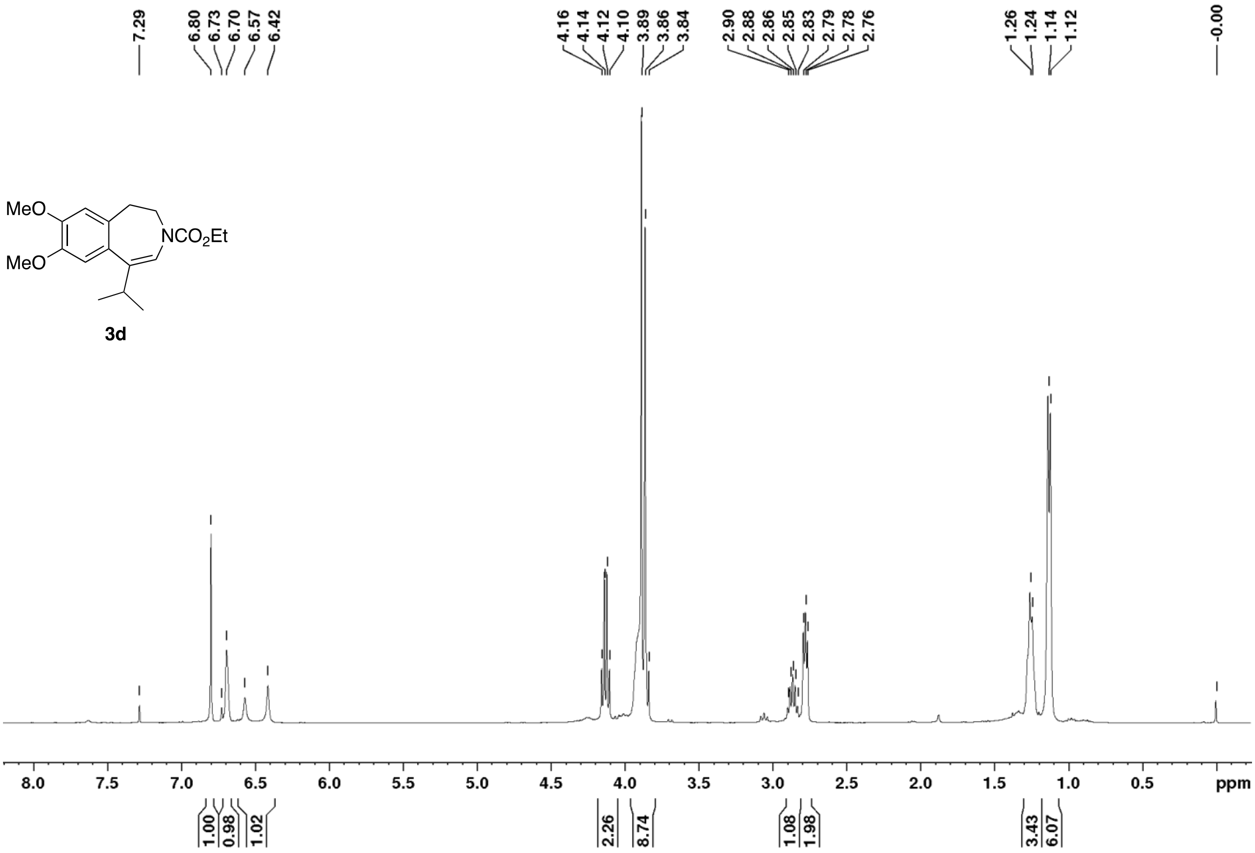

^{13}C NMR (100 MHz, $CDCl_3$)

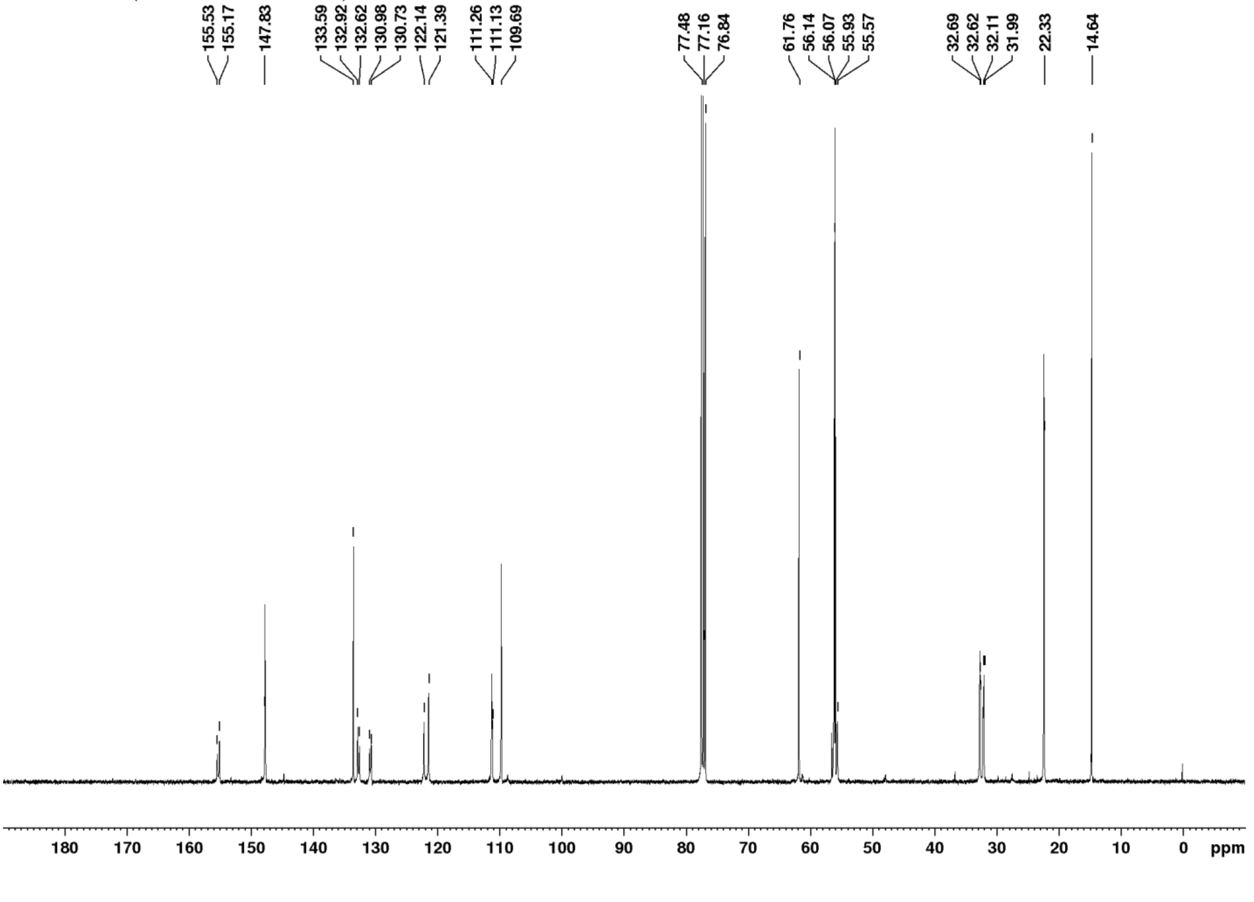

^{1}H NMR (400 MHz, $CDCl_3$)

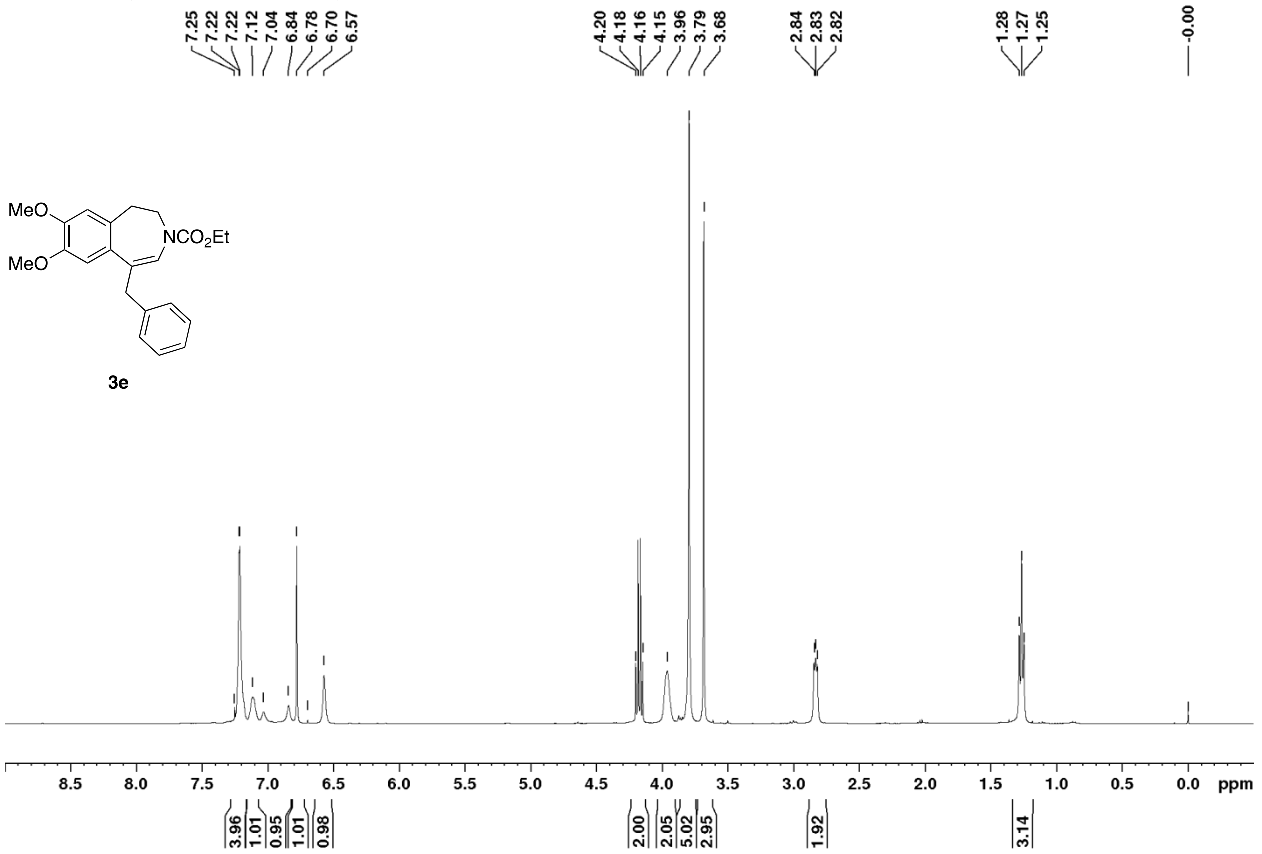

^{13}C NMR (100 MHz, $CDCl_3$)

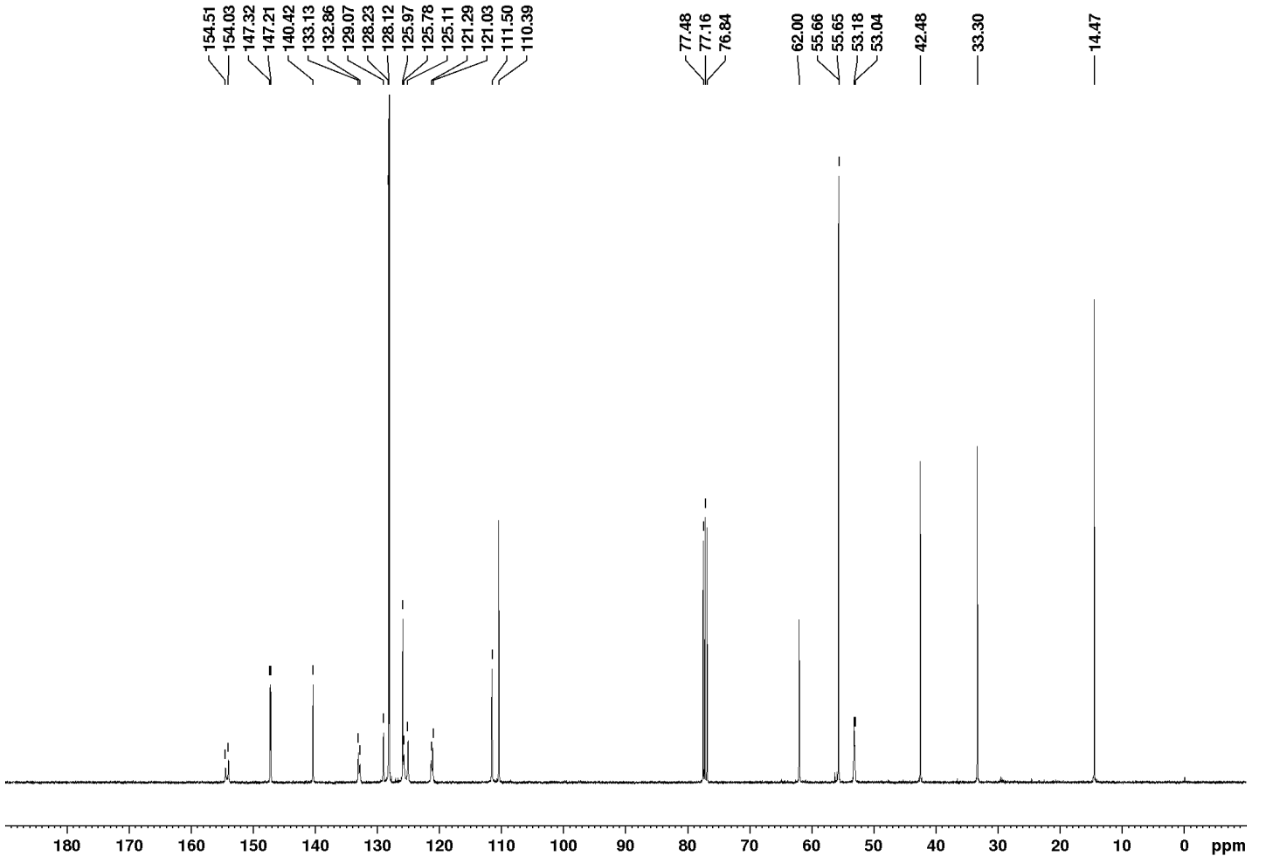

NMR spectra – Hydrogenated products

^{1}H NMR (400 MHz, $CDCl_3$)

BRP4-462-A/40

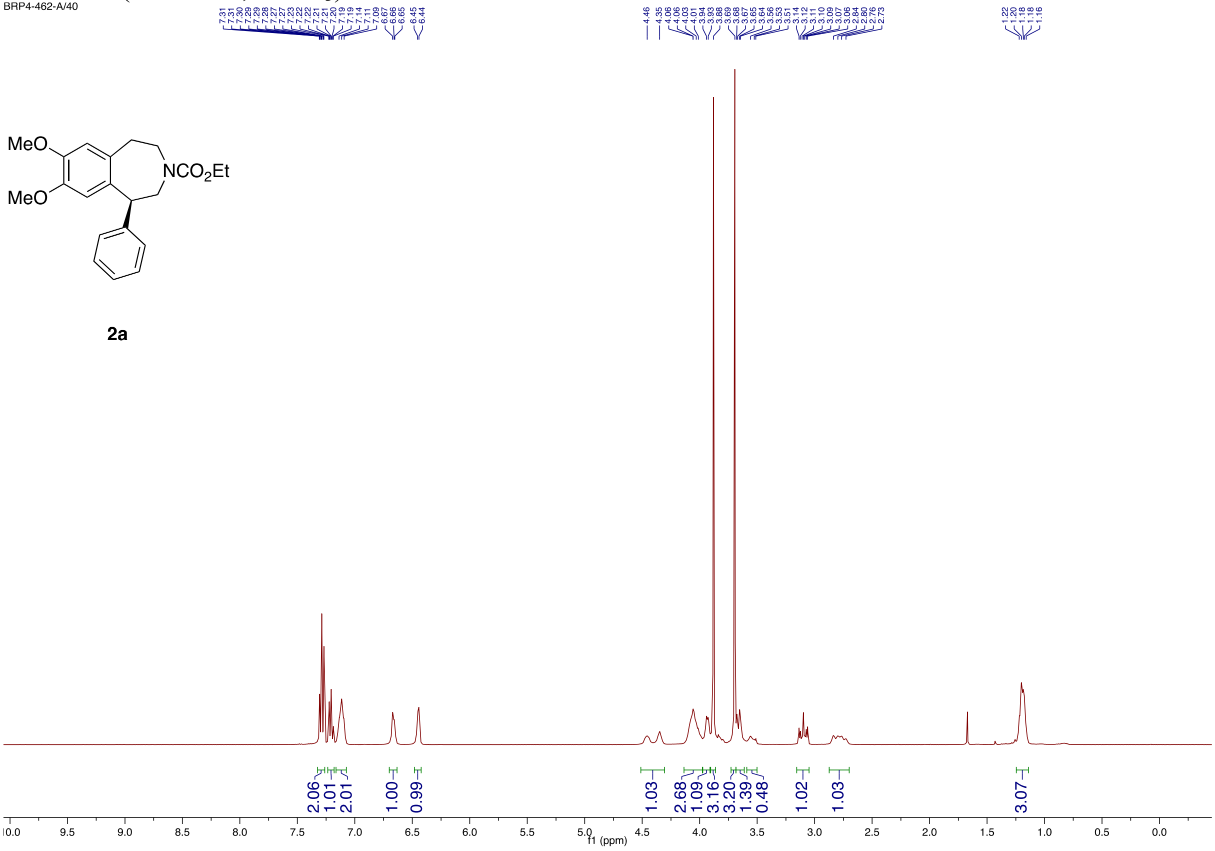

^{13}C NMR (125 MHz, $CDCl_3$)

BRP4-462-A/2
BRP4-461-A

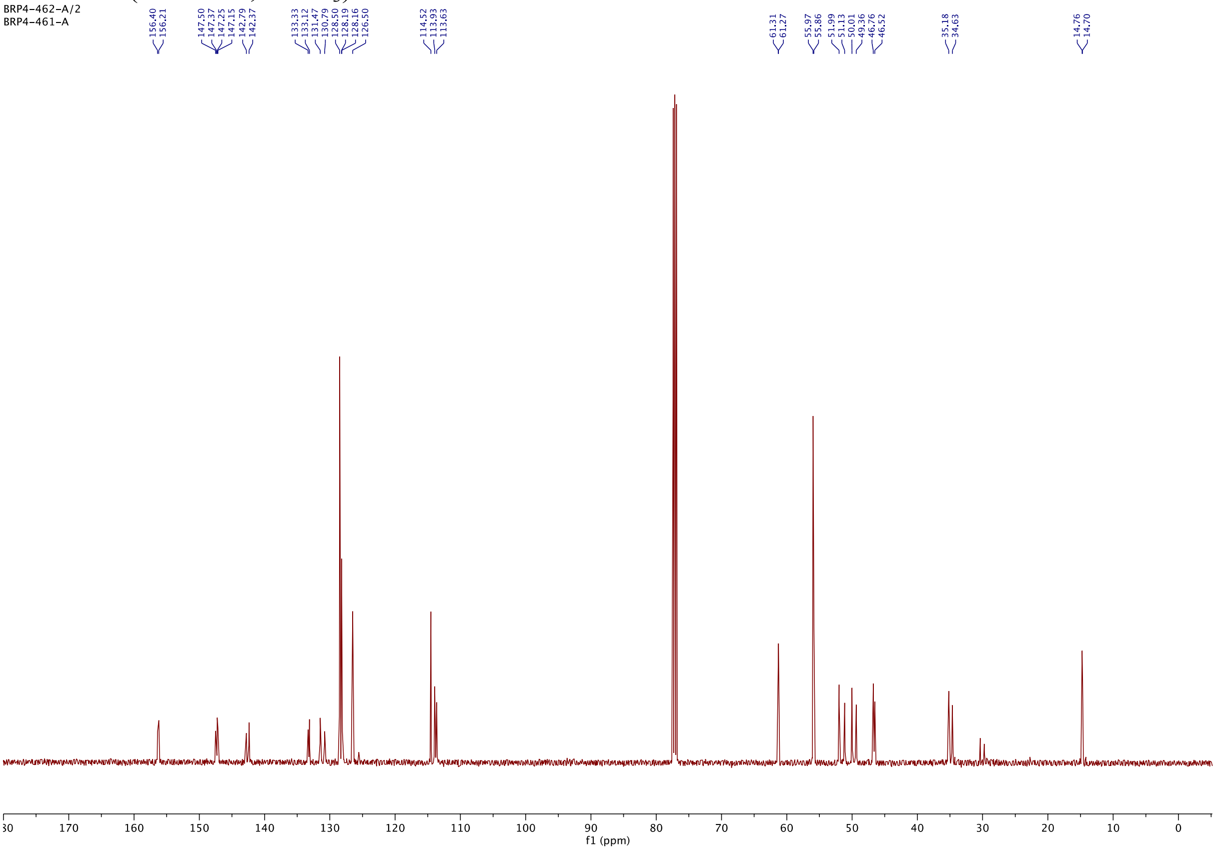

^{1}H NMR (400 MHz, $CDCl_3$)

BRP4-462-B/50

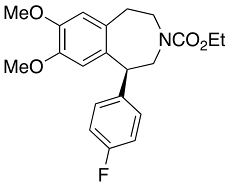

2b

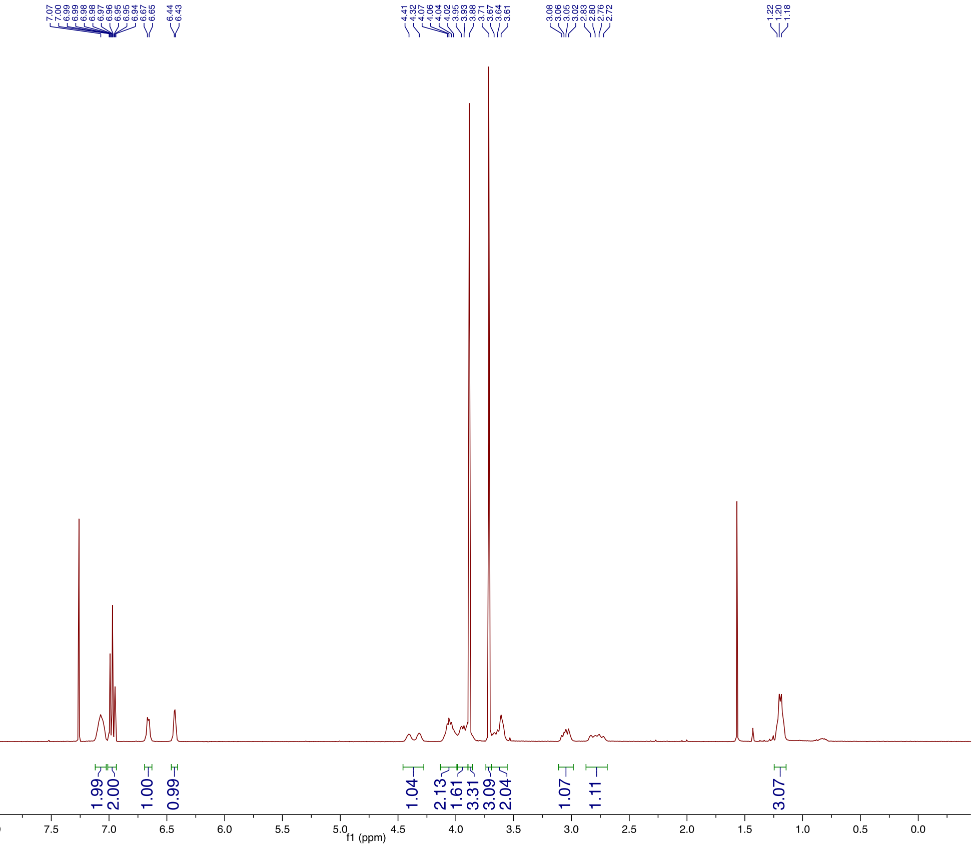

^{13}C NMR (125 MHz, $CDCl_3$)

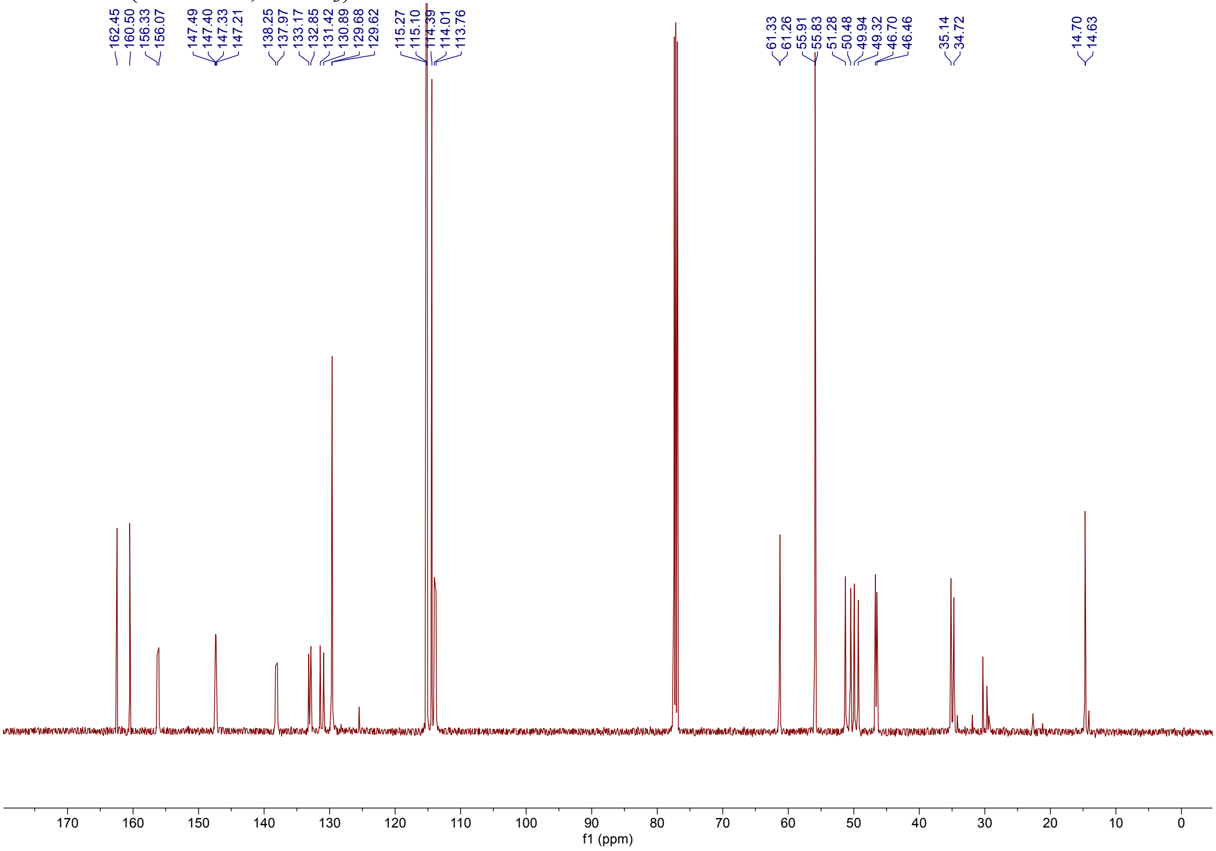

^{19}F NMR (376 MHz, $CDCl_3$)

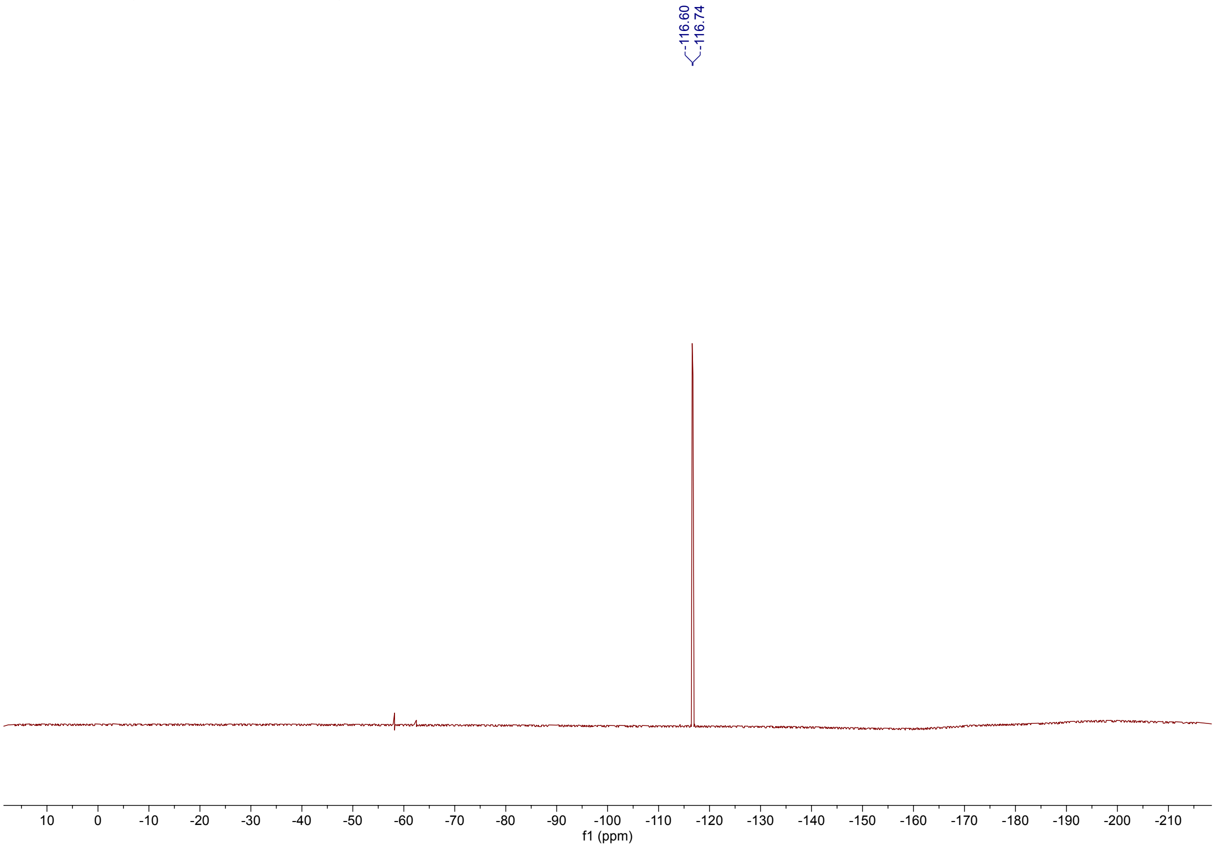

^{19}F NMR (376 MHz, CD_3OD)

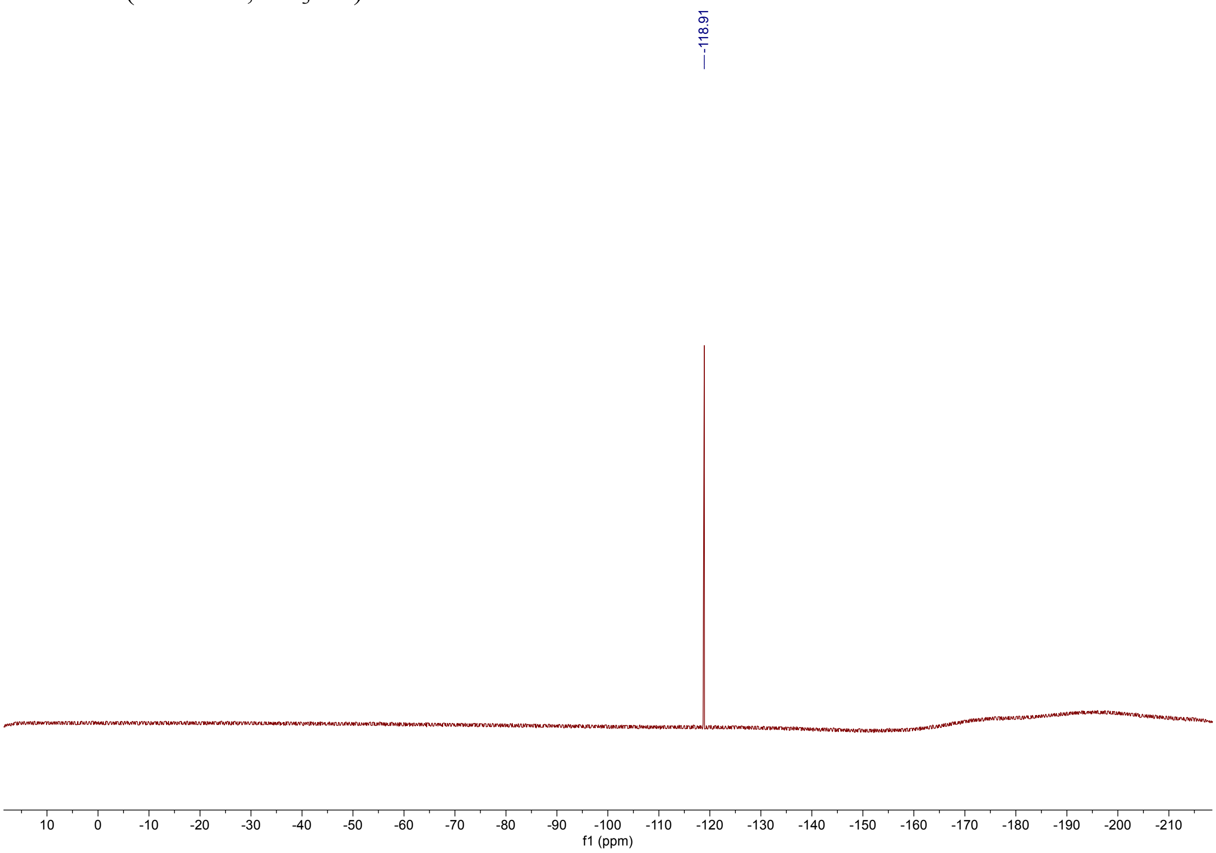

^{1}H NMR (400 MHz, $CDCl_3$)

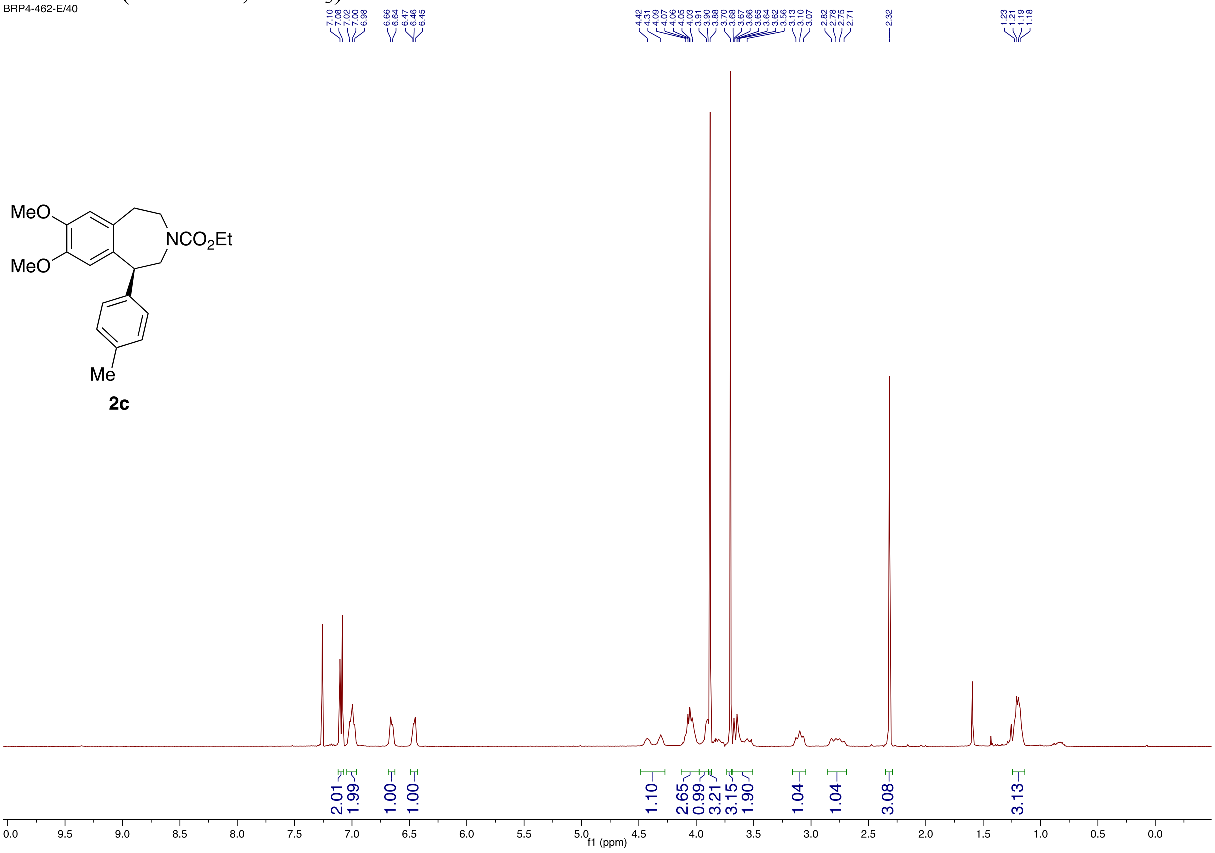

2c

^{13}C NMR (125 MHz, $CDCl_3$)

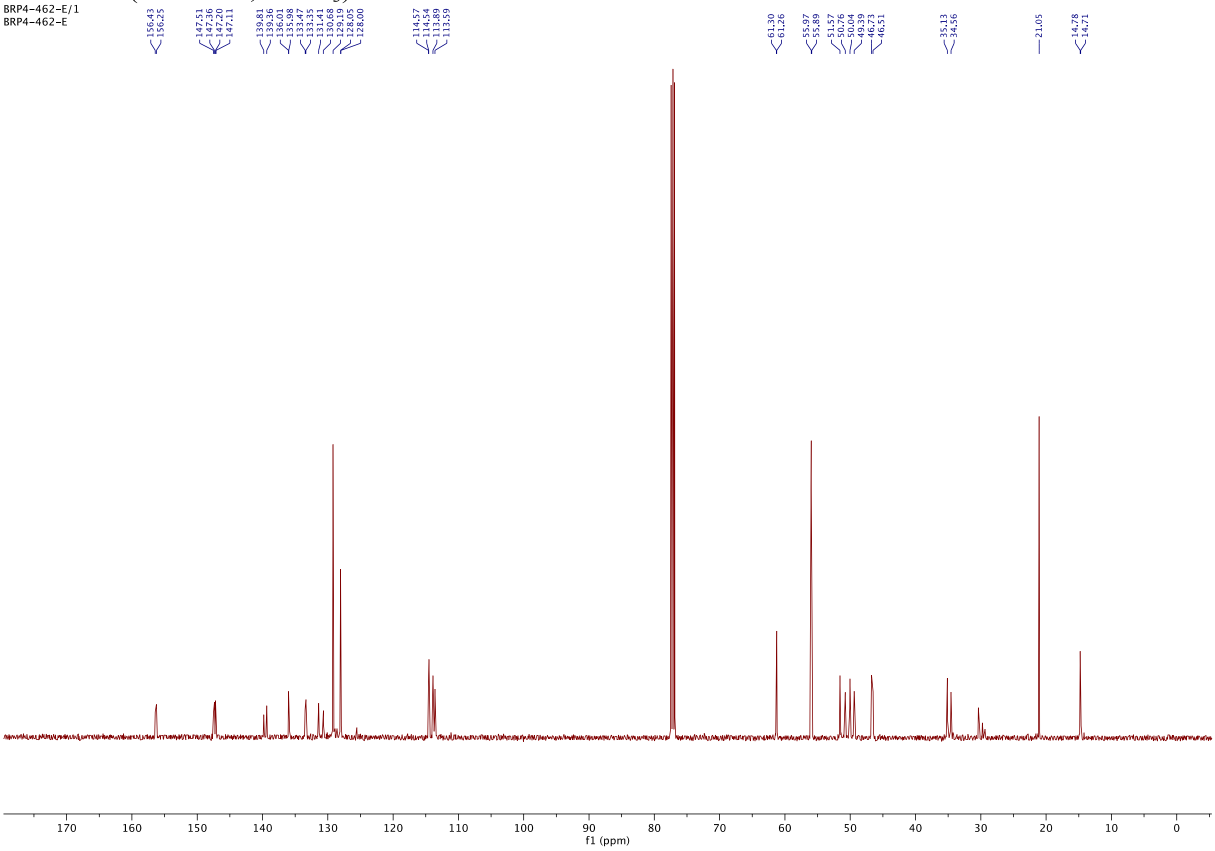

^{1}H NMR (400 MHz, $CDCl_3$)

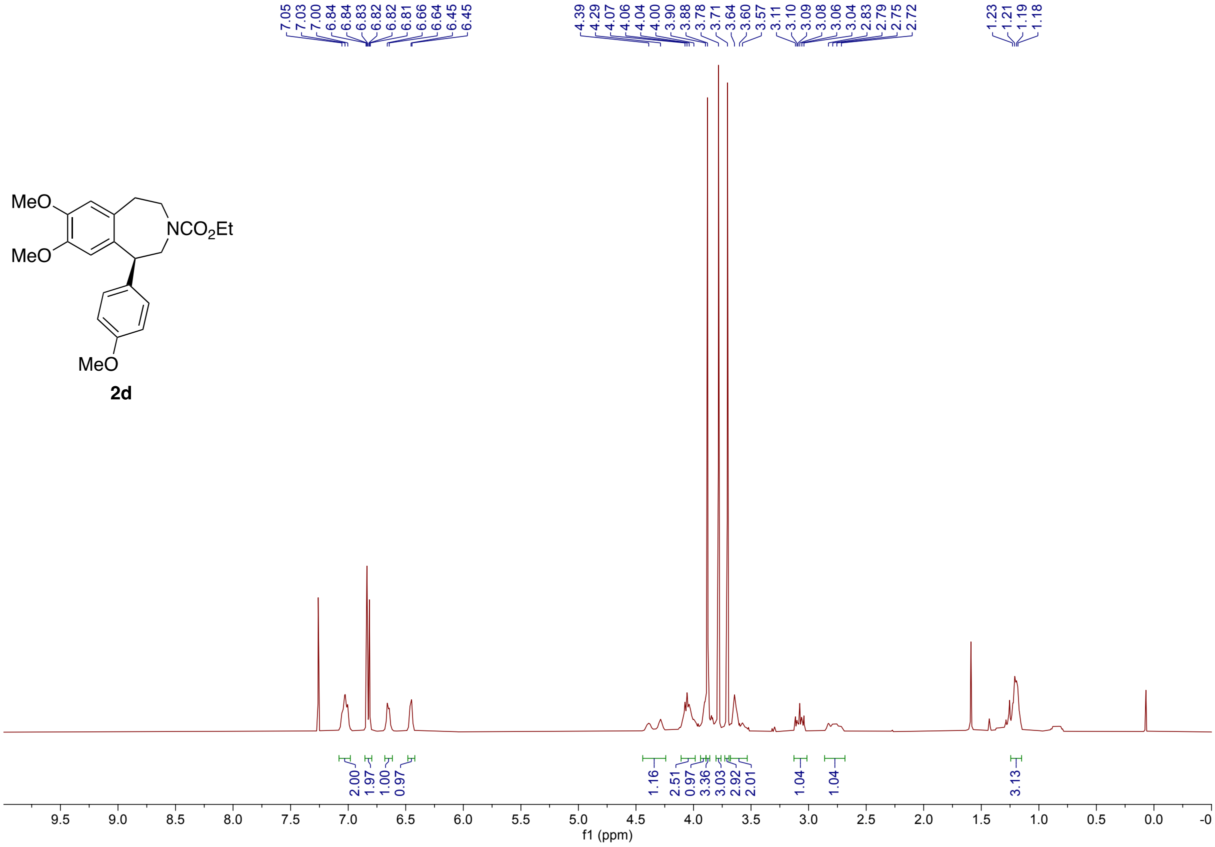

^{13}C NMR (125 MHz, $CDCl_3$)

BRP4-462-F/1
BRP4-462-F

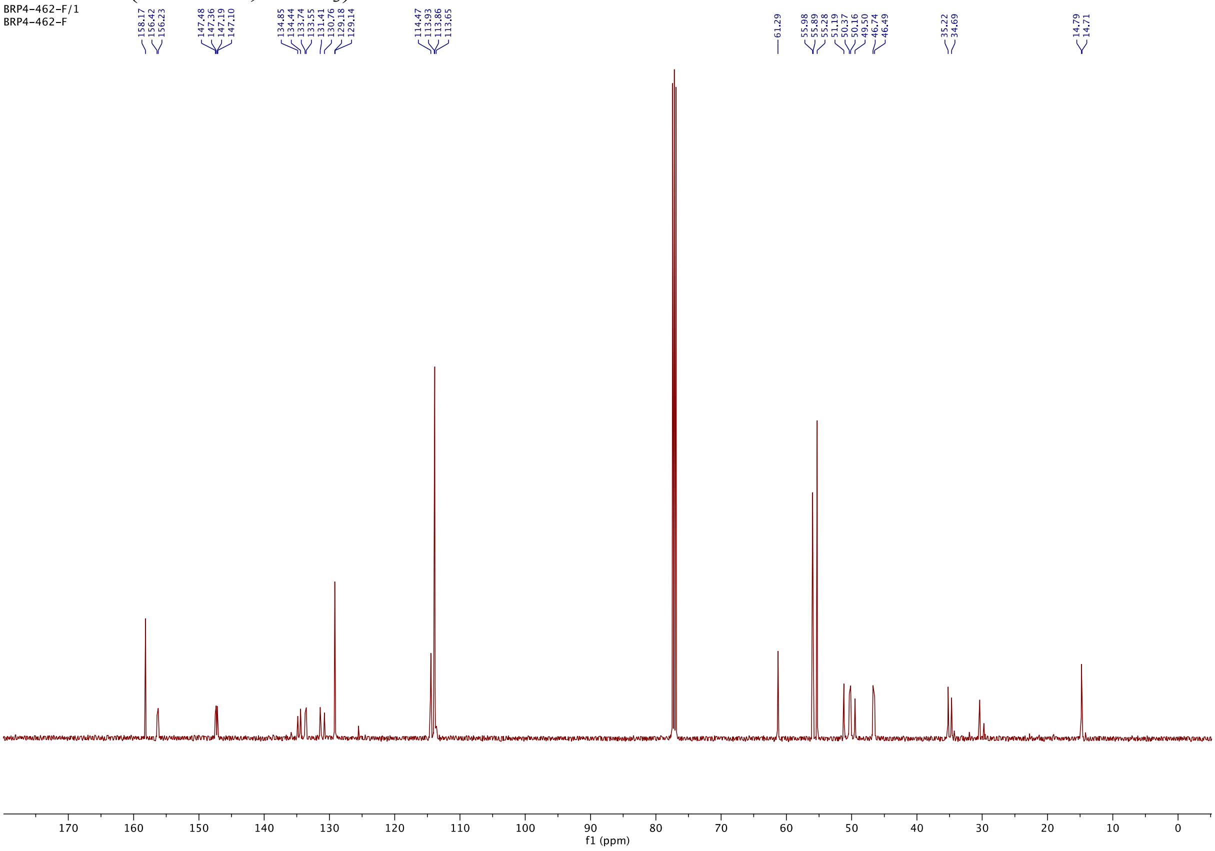

^{1}H NMR (500 MHz, DMSO-d_6, 80 °C)

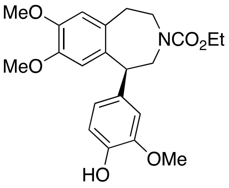

2e

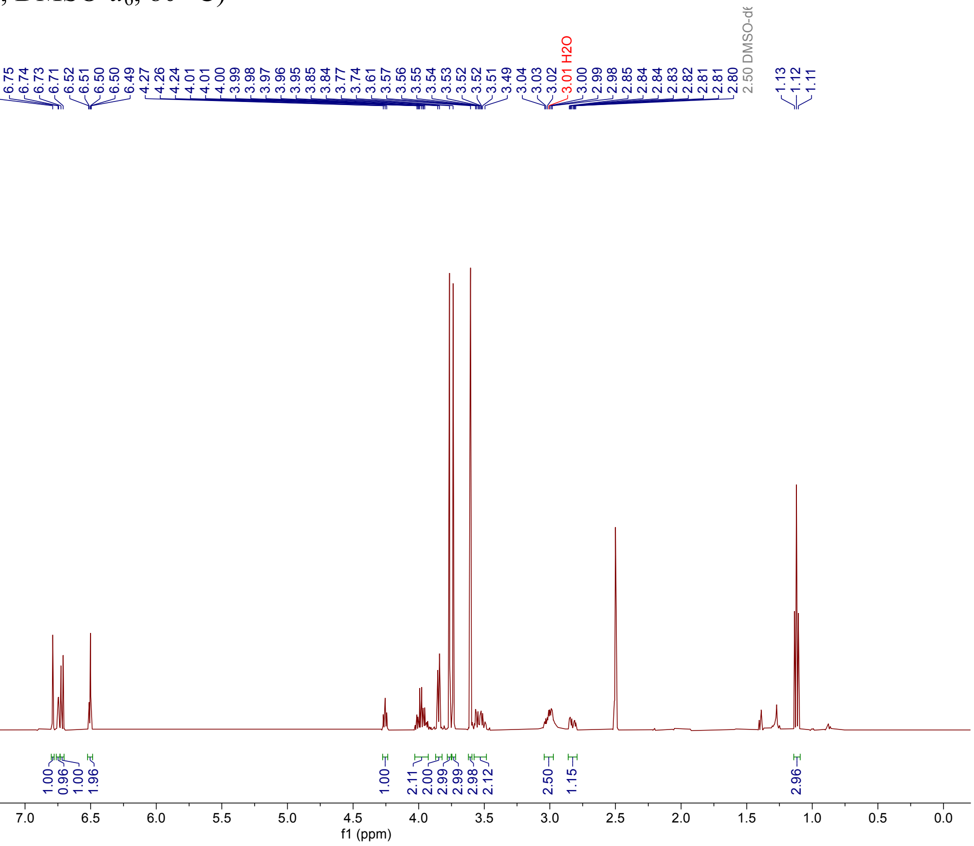

^{13}C NMR (125 MHz, $CDCl_3$)

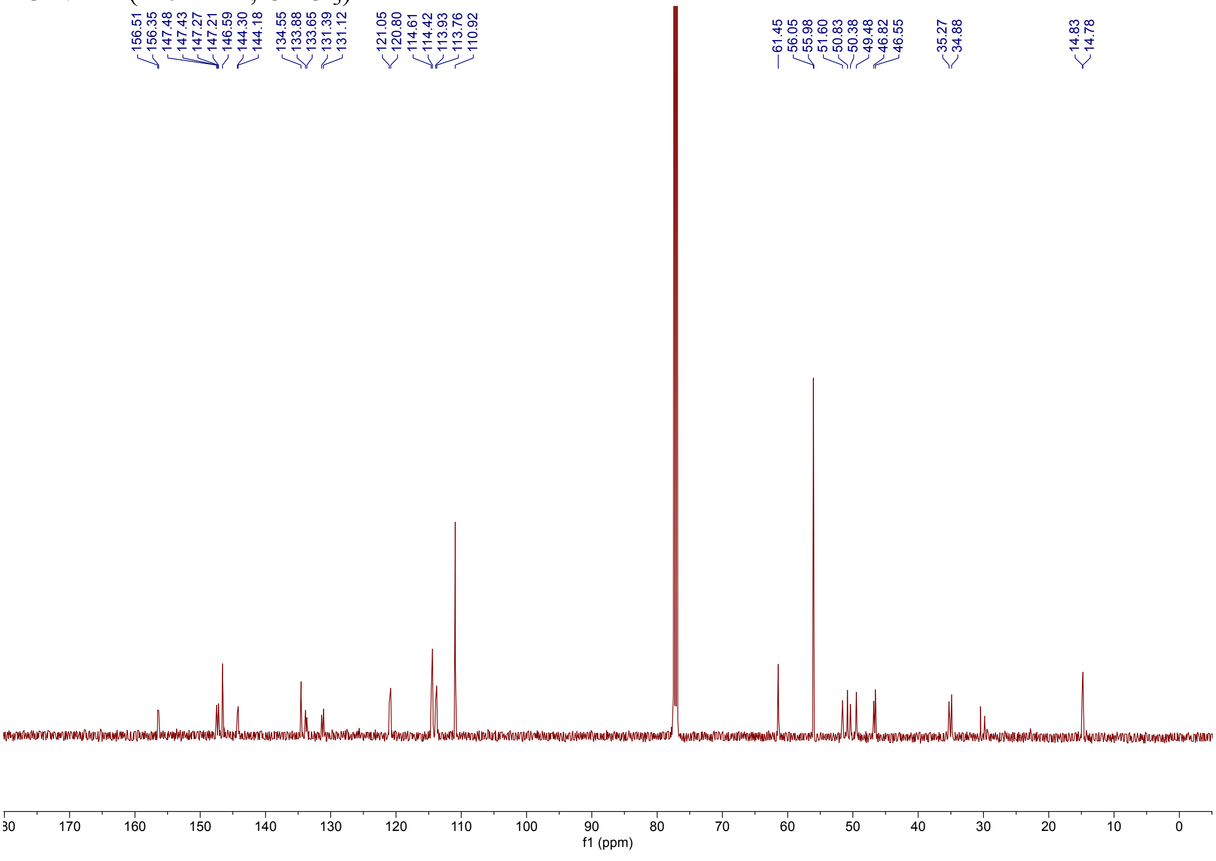

¹H NMR (400 MHz, CDCl₃)

BRP4-462-D/30

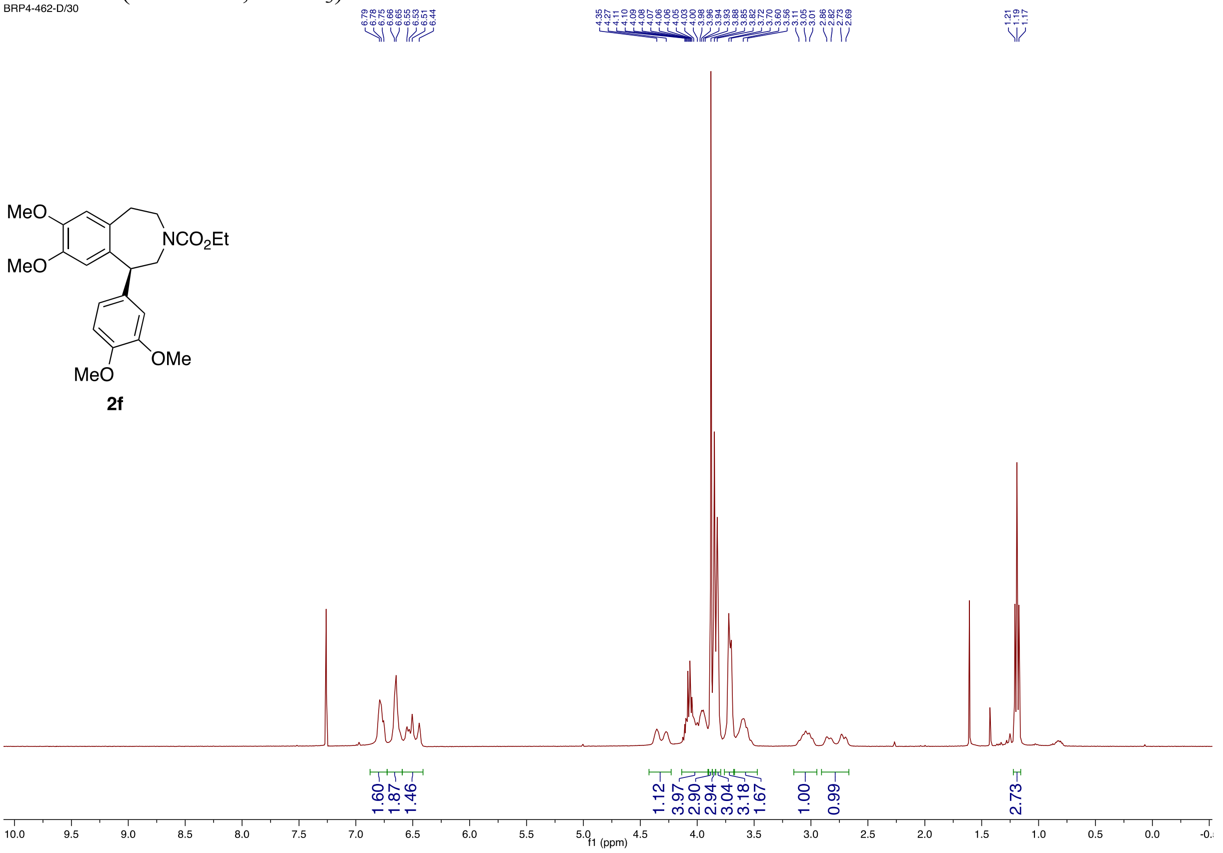

2f

¹³C NMR (125 MHz, CDCl₃)

BRP4-462-D/1
BRP4-462-D

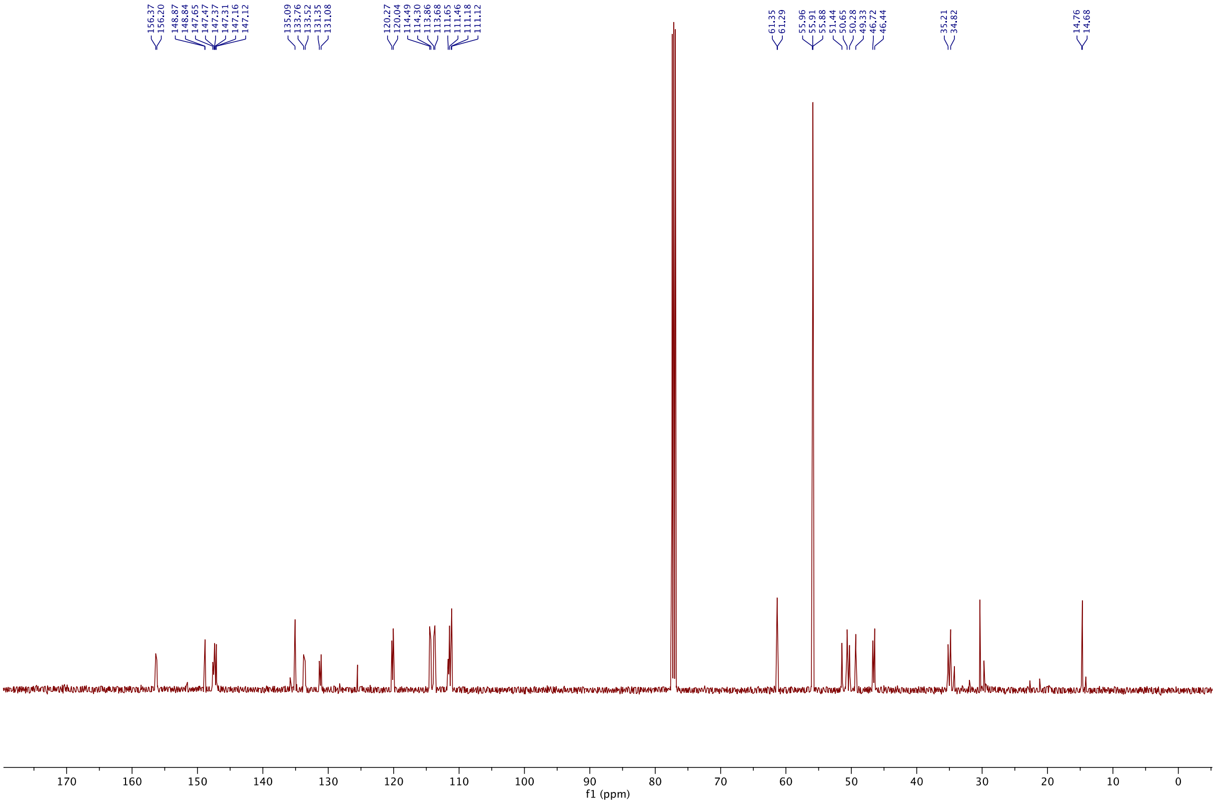

^{1}H NMR (400 MHz, $CDCl_3$)

BRP4-462-G/10

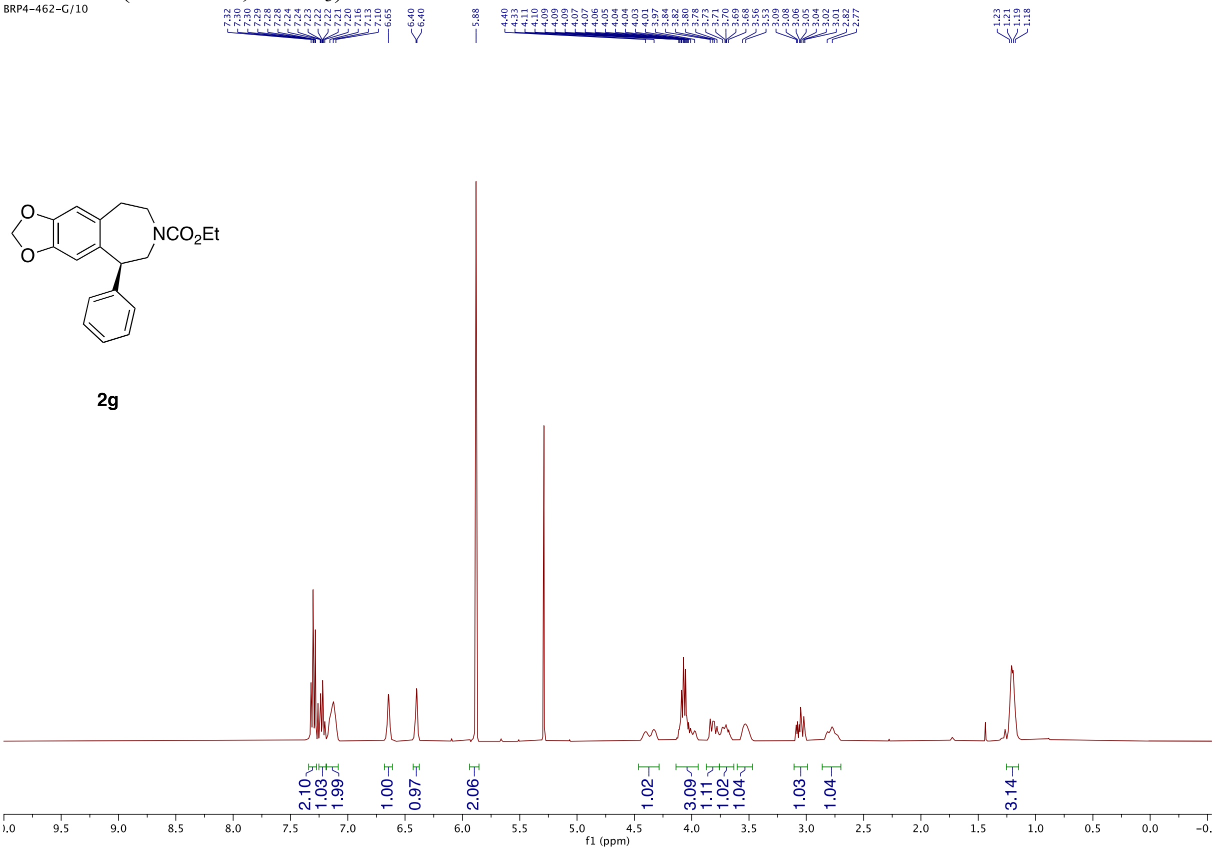

2g

^{13}C NMR (125 MHz, $CDCl_3$)

BRP4-462-G/1
BRP4-462-G

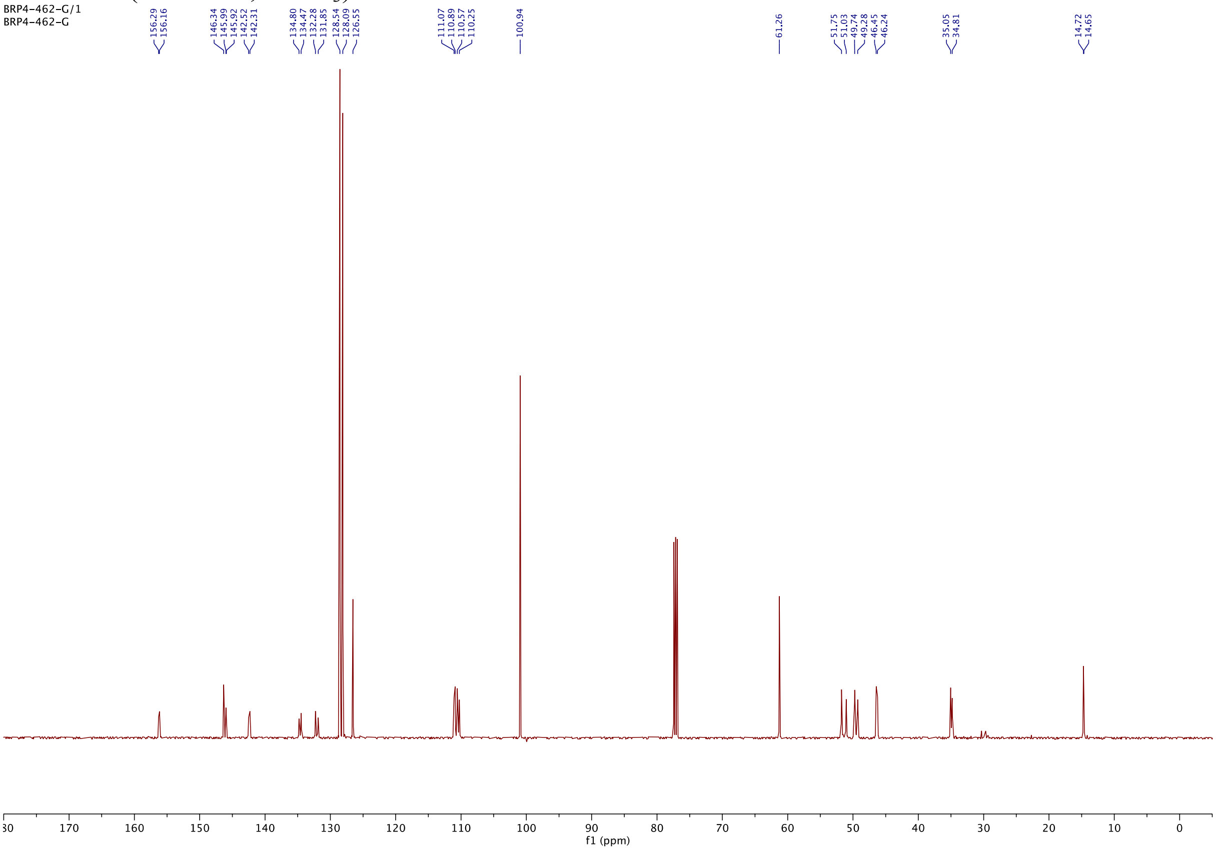

^{1}H NMR (400 MHz, $CDCl_3$)

BRP4-462-H/30

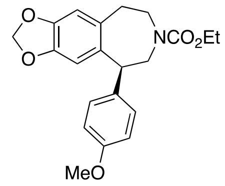

2h

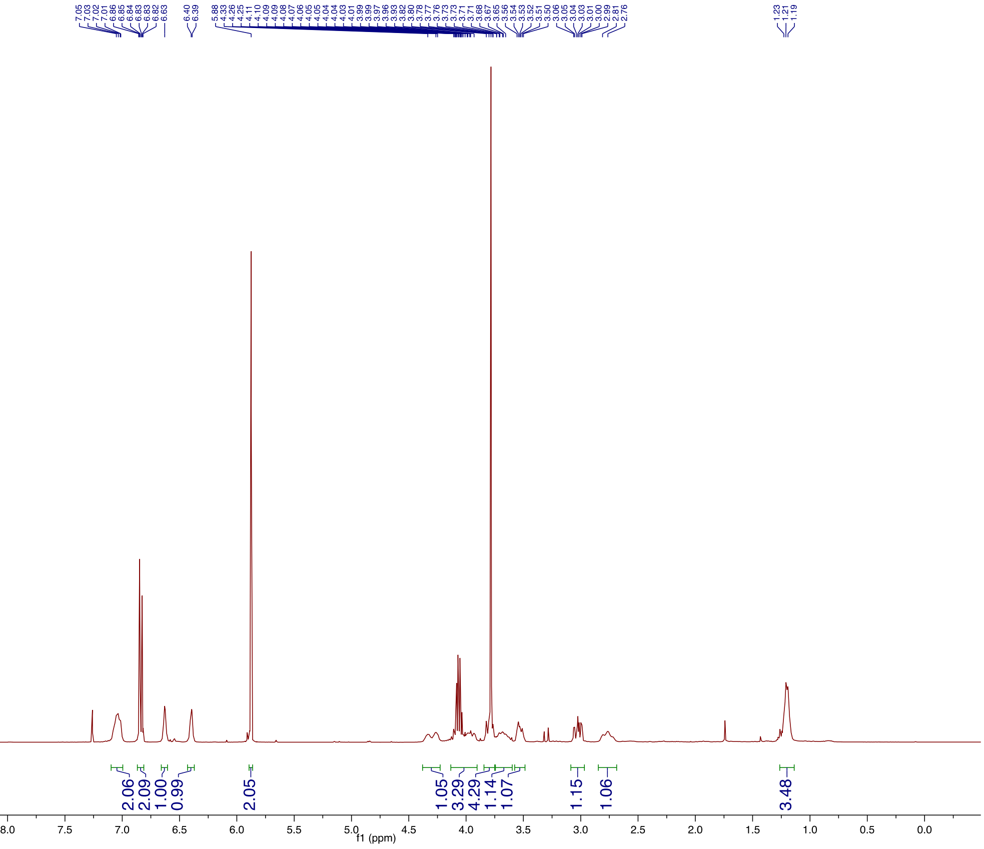

^{13}C NMR (125 MHz, $CDCl_3$)

BRP4-462-H/1
BRP4-462-H

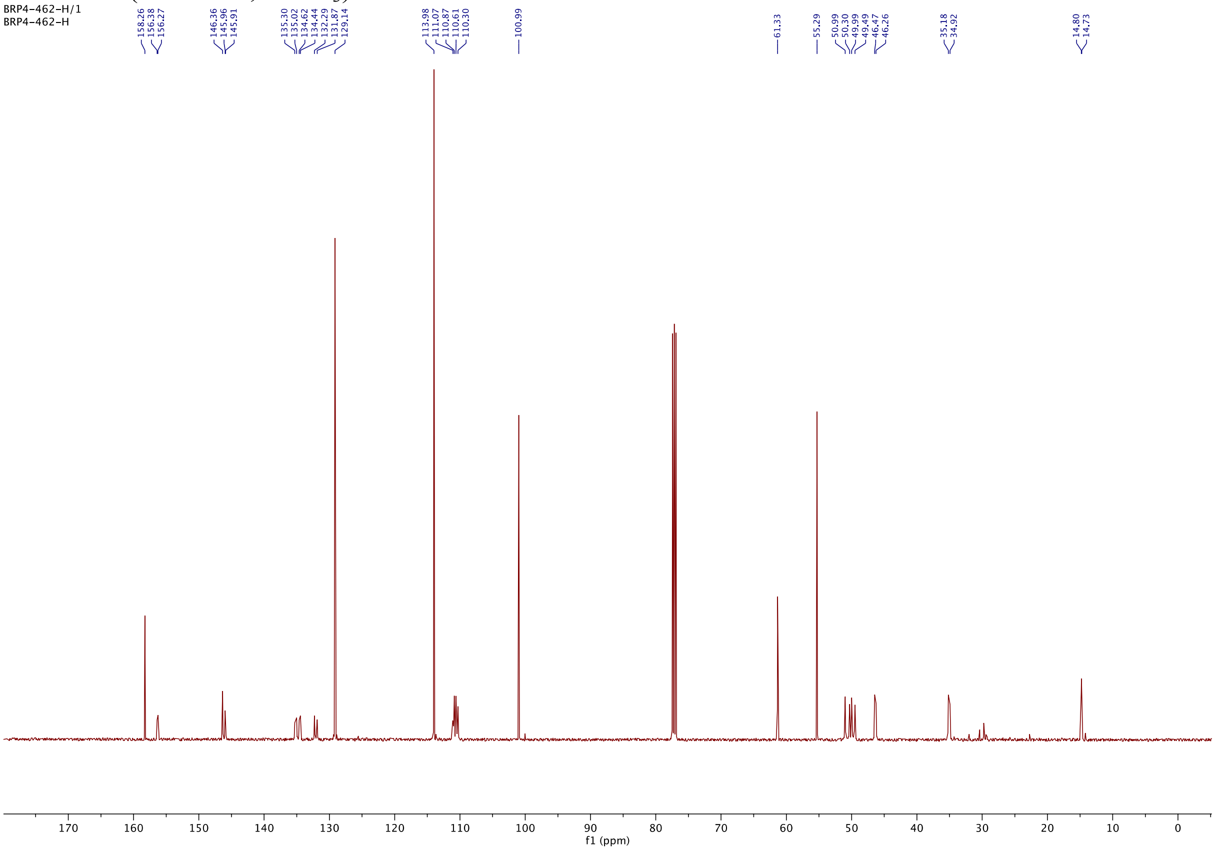

^{1}H NMR (400 MHz, $CDCl_3$)

BRP4-462-I/20

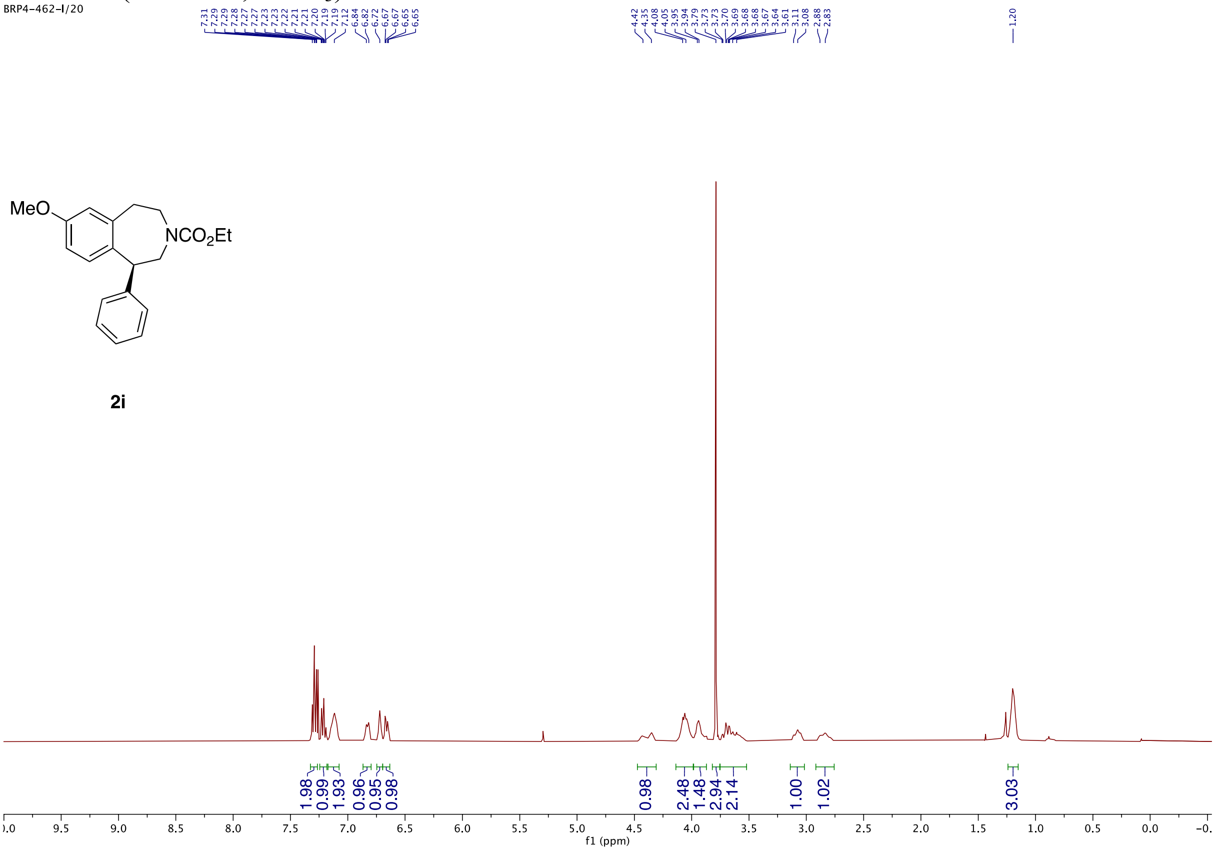

2i

^{13}C NMR (125 MHz, $CDCl_3$)

BRP4-462-I/10
BRP4-462-I

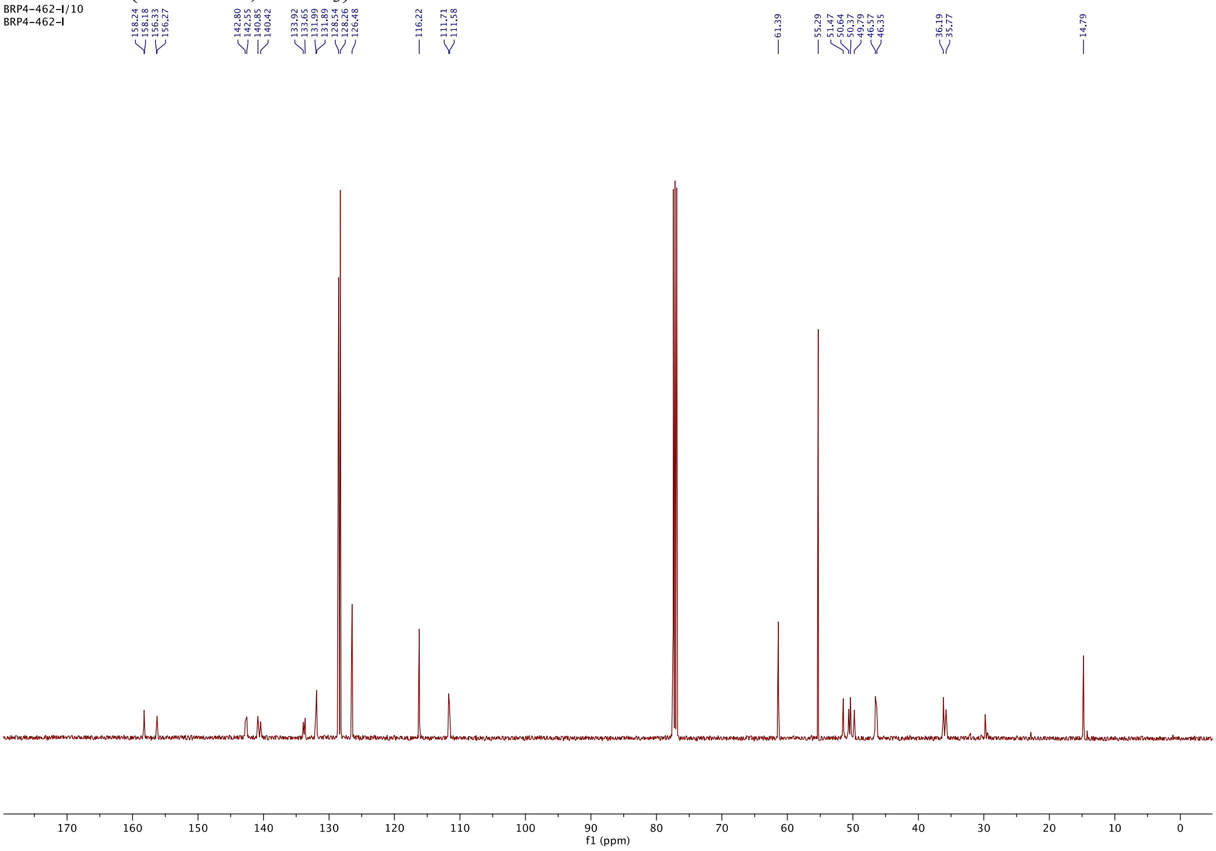

^{1}H NMR (400 MHz, $CDCl_3$)

BRP4-462-K/20

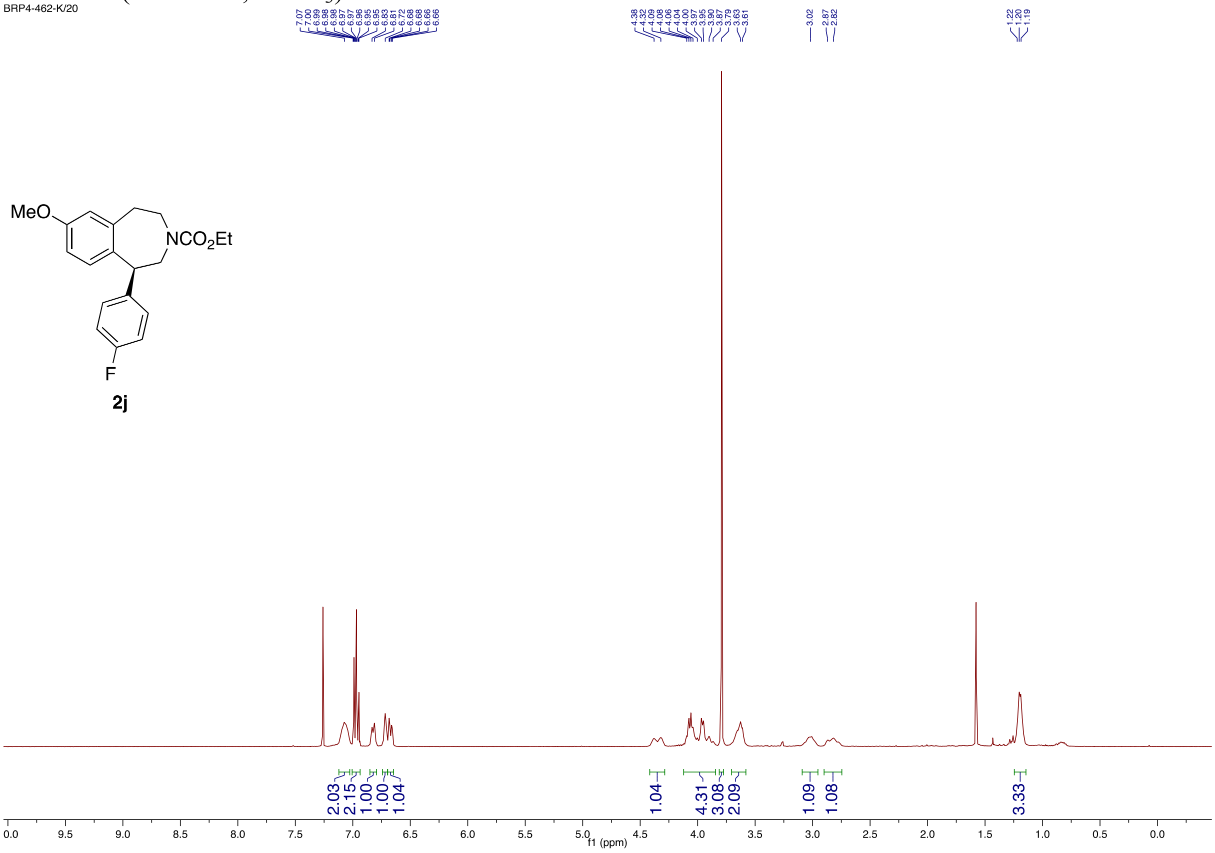

^{13}C NMR (125 MHz, $CDCl_3$)

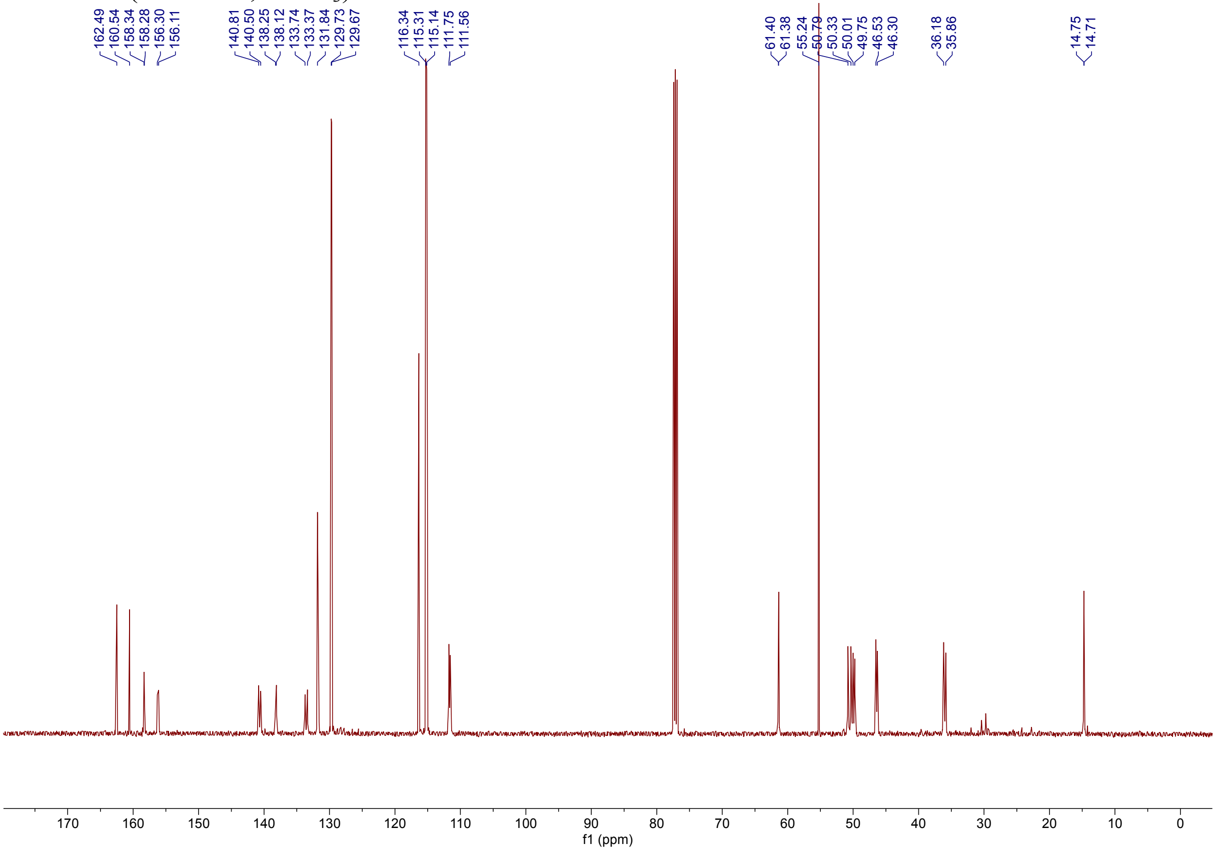

^{19}F NMR (376 MHz, $CDCl_3$)

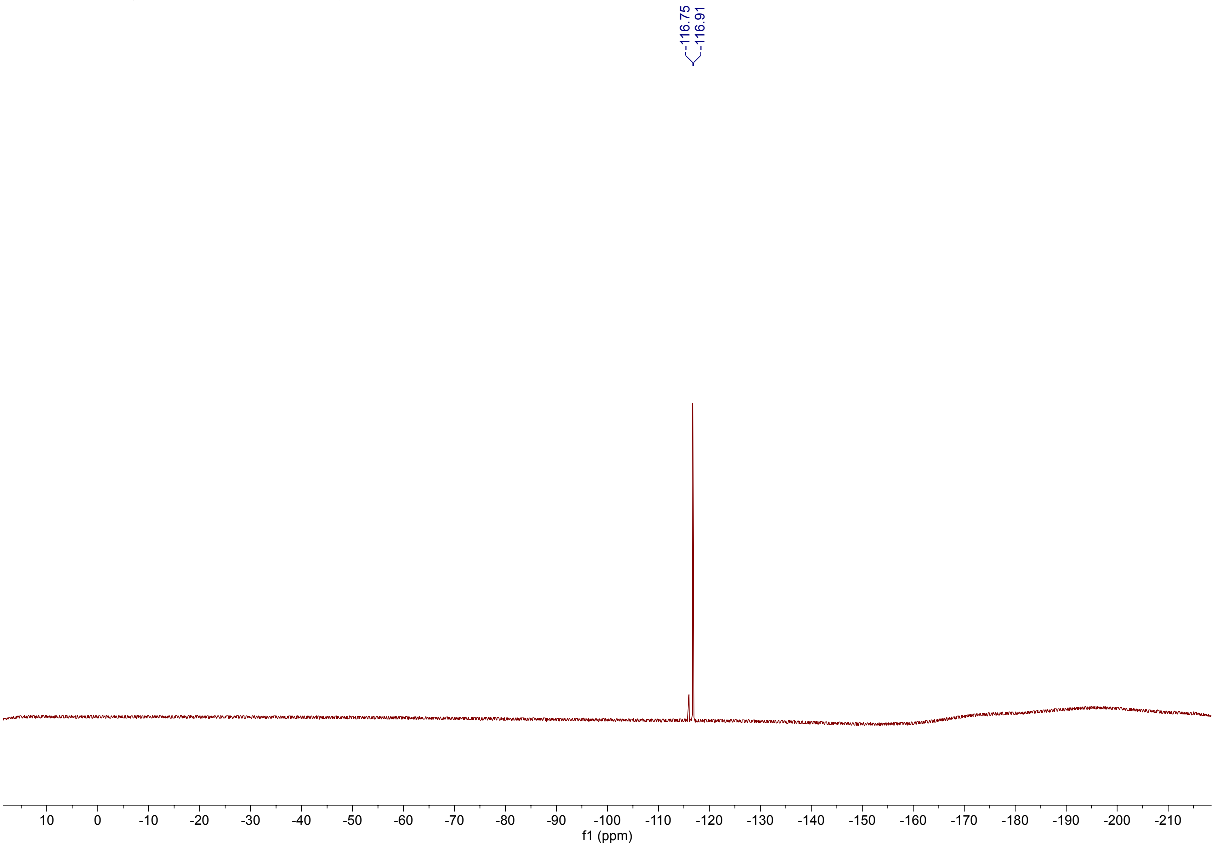

^{1}H NMR (400 MHz, $CDCl_3$)

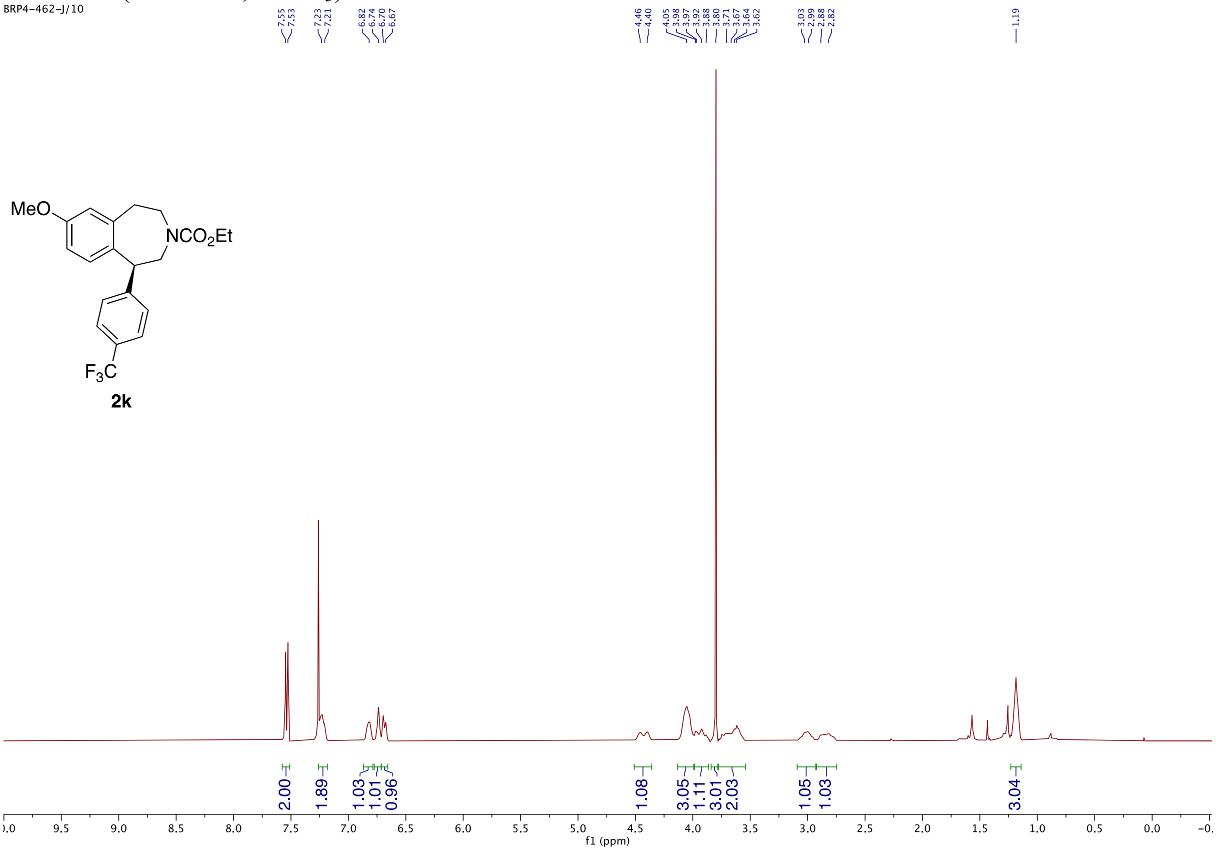

2k

^{13}C NMR (125 MHz, $CDCl_3$)

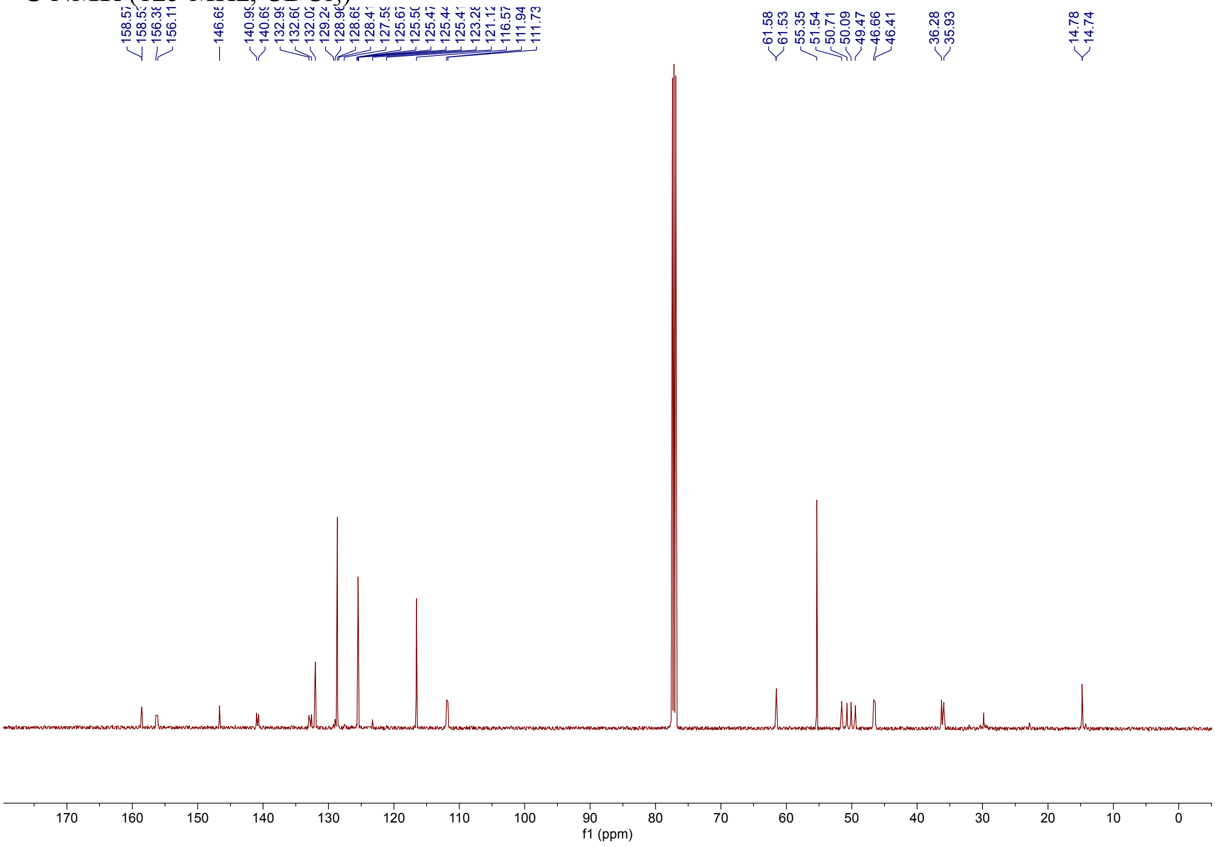

^{19}F NMR (376 MHz, $CDCl_3$)

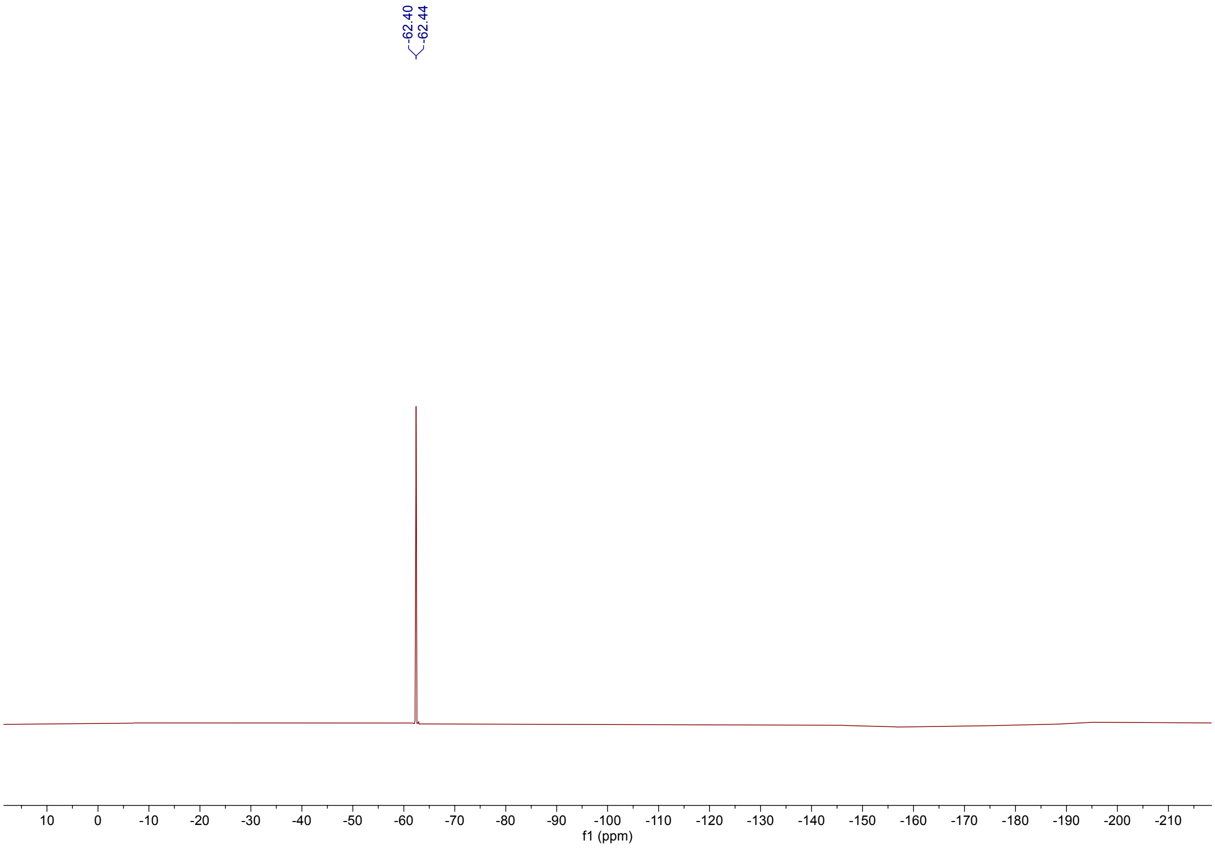

^{1}H NMR (400 MHz, $CDCl_3$)

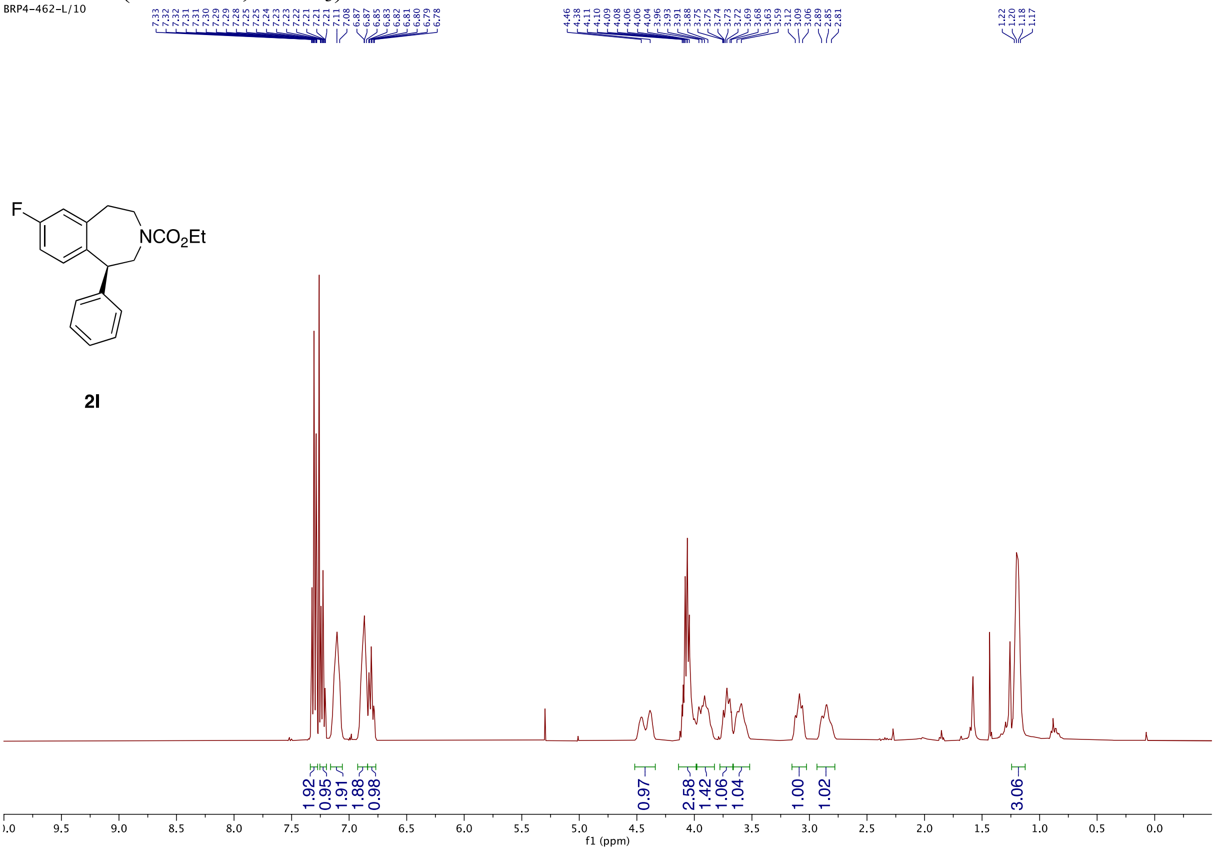

^{13}C NMR (125 MHz, $CDCl_3$)

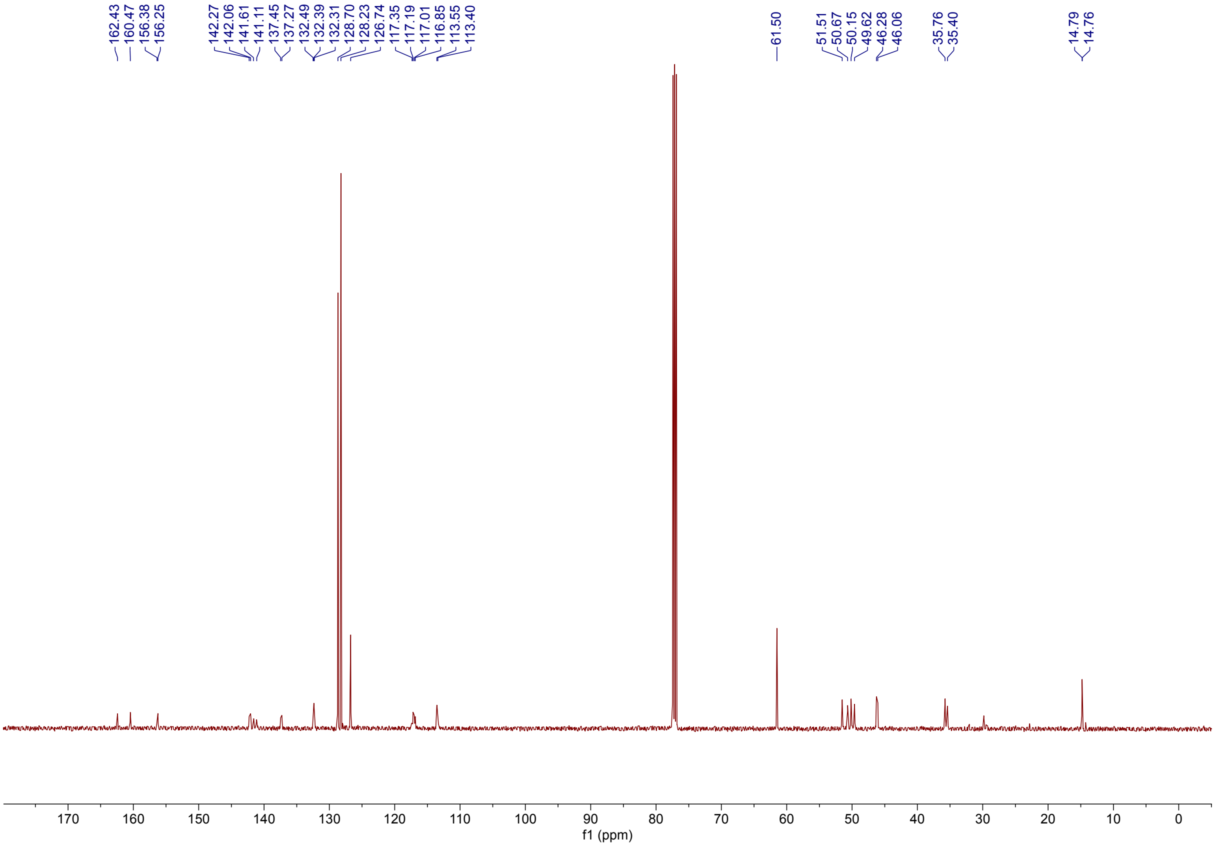

^{19}F NMR (376 MHz, $CDCl_3$)

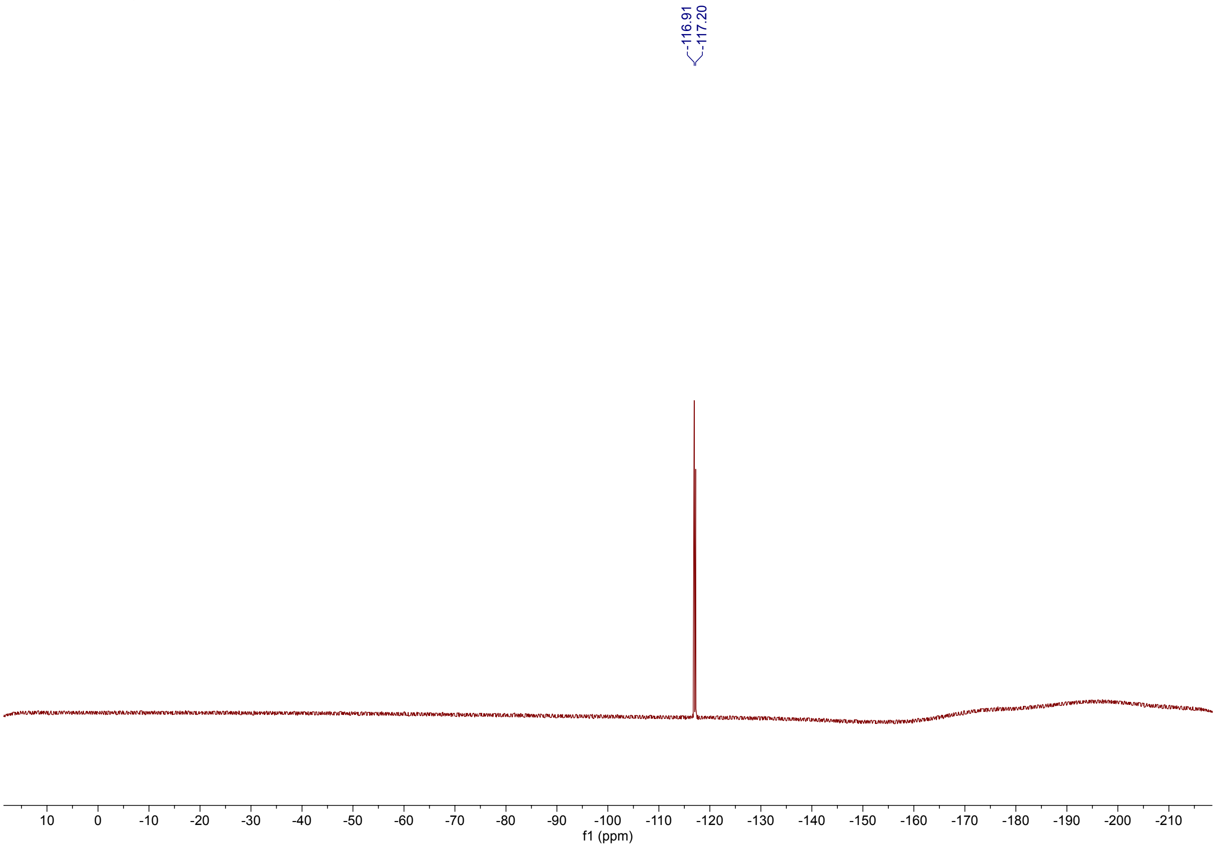

^{1}H NMR (400 MHz, $CDCl_3$)

BRP4-462-M/30

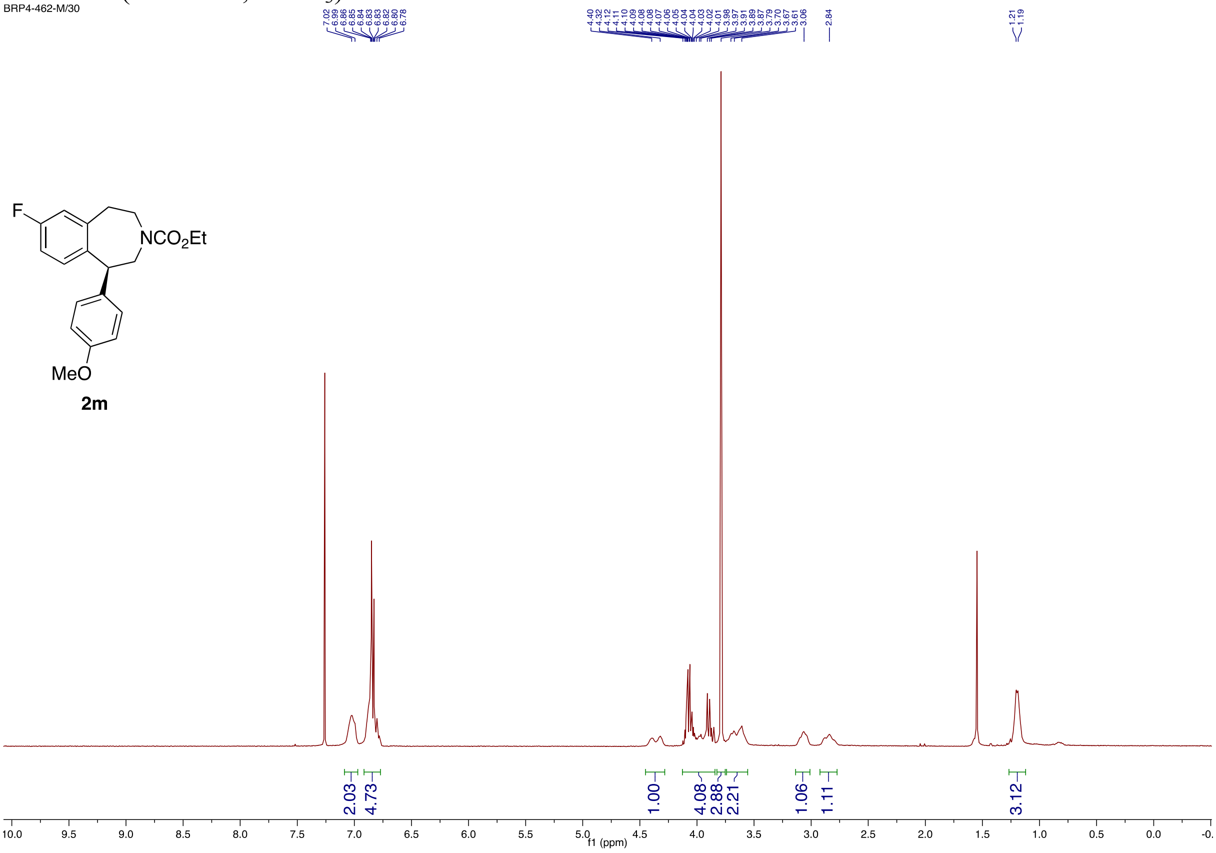

^{13}C NMR (125 MHz, $CDCl_3$)

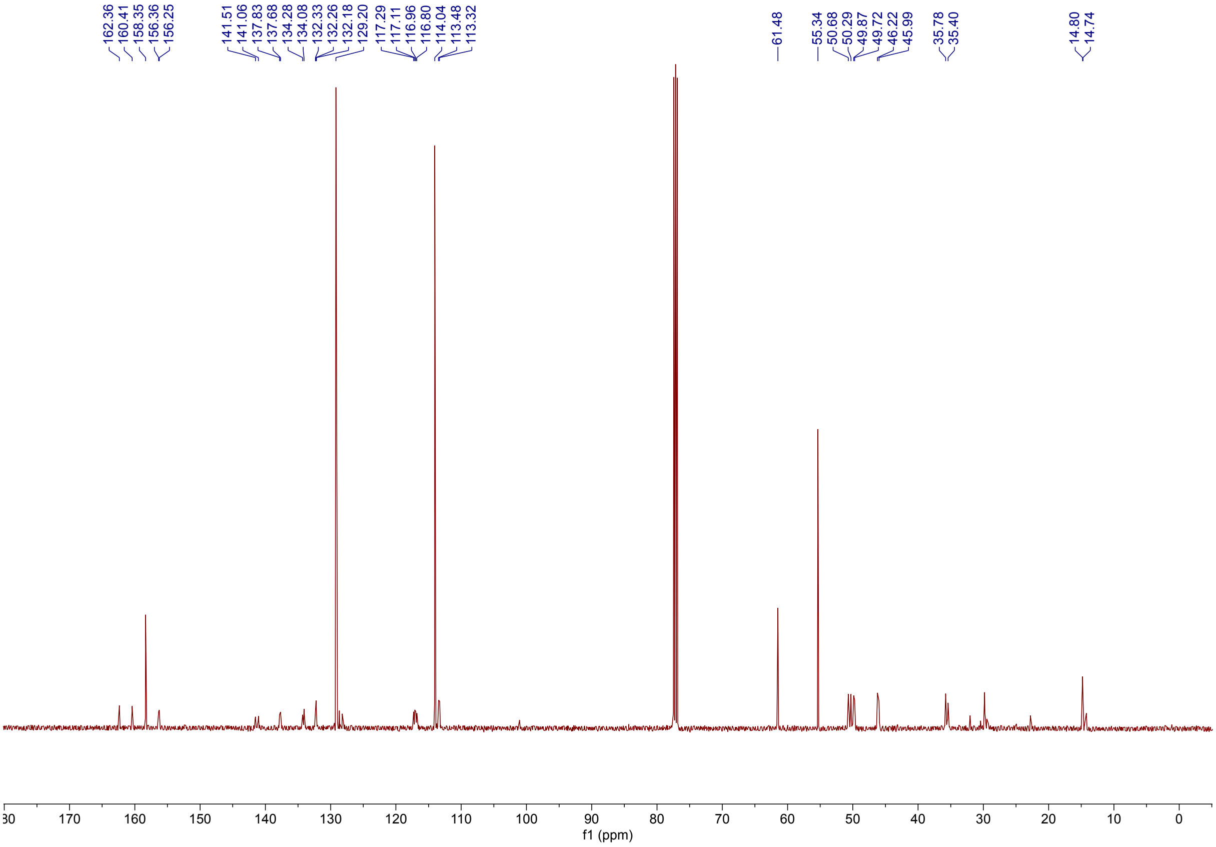

^{19}F NMR (376 MHz, $CDCl_3$)

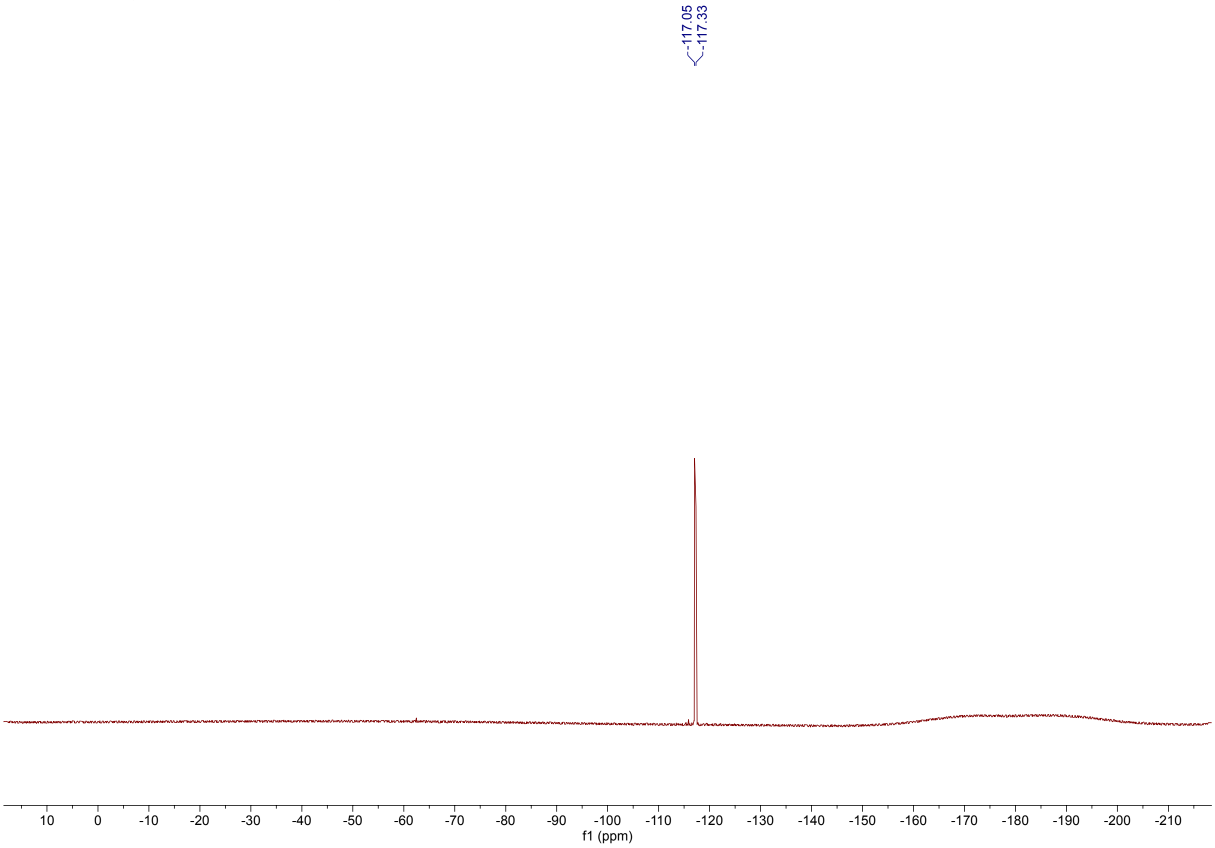

^{1}H NMR (400 MHz, $CDCl_3$)

BRP4-462-N/20

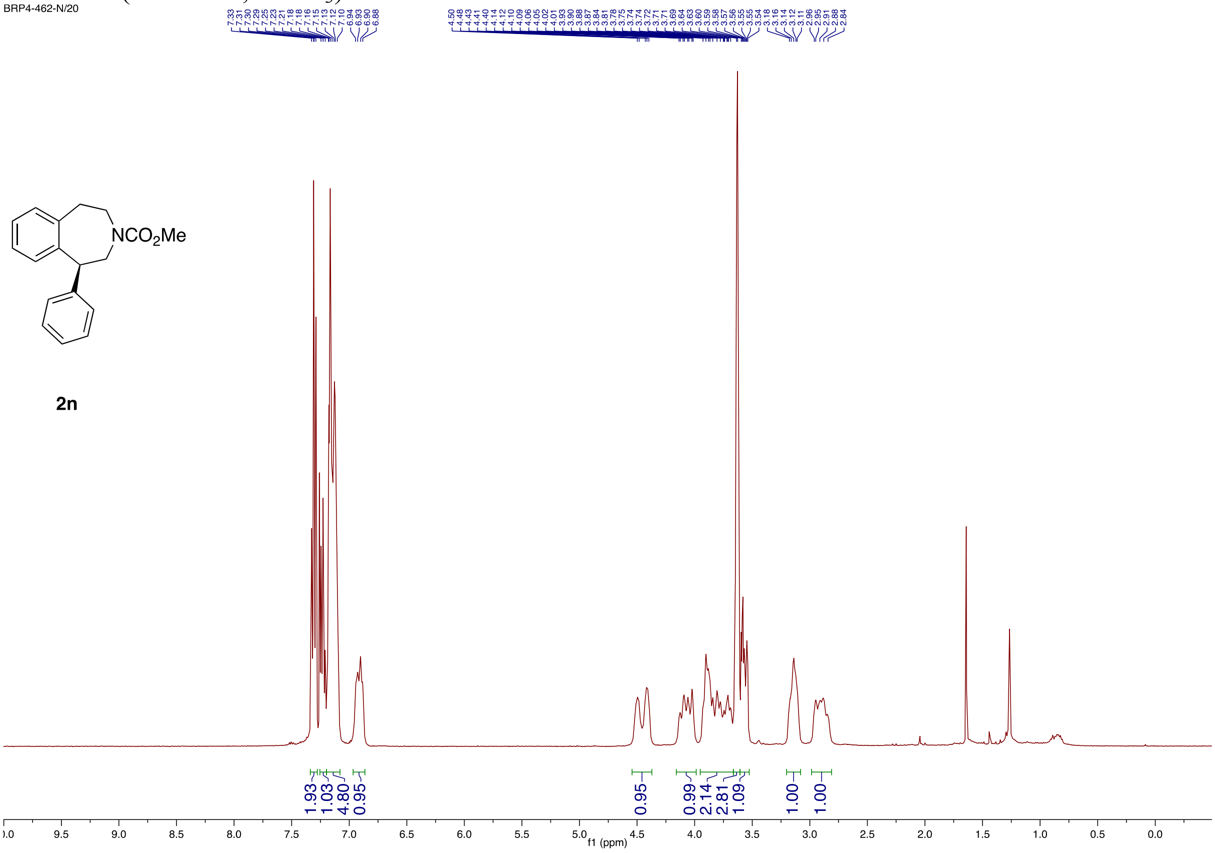

^{13}C NMR (125 MHz, $CDCl_3$)

BRP4-462-N/1
BRP4-462-N

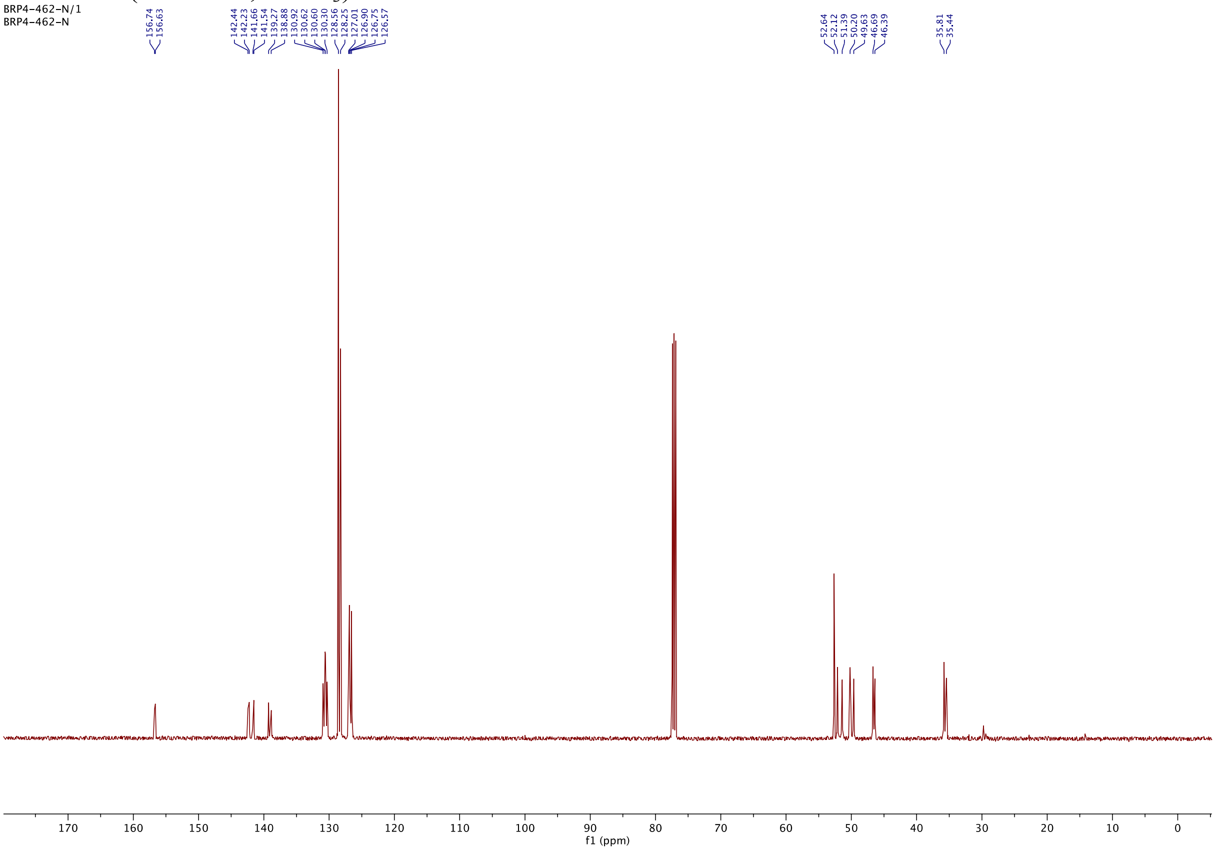

^{1}H NMR (400 MHz, $CDCl_3$)

BRP4-462-P/10

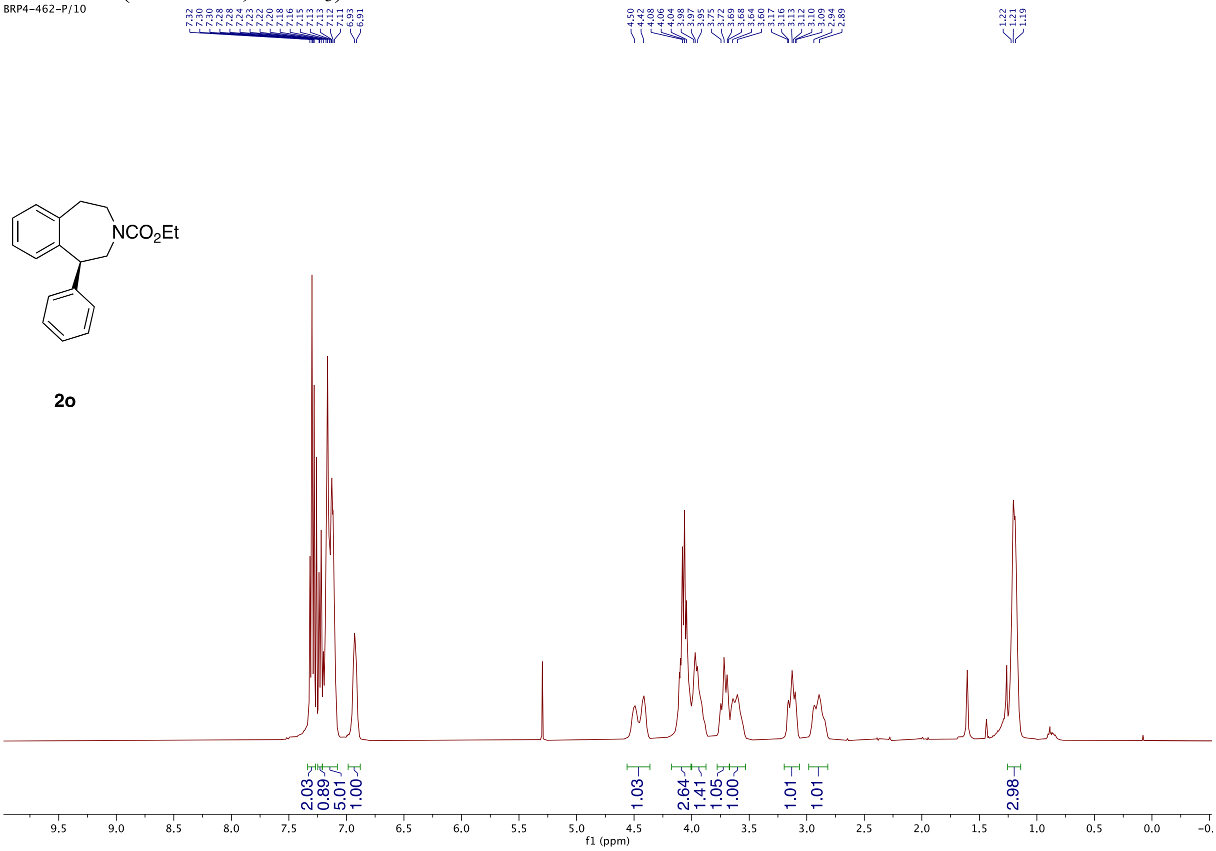

^{13}C NMR (125 MHz, $CDCl_3$)

BRP4-462-P/1

BRP4-462-P

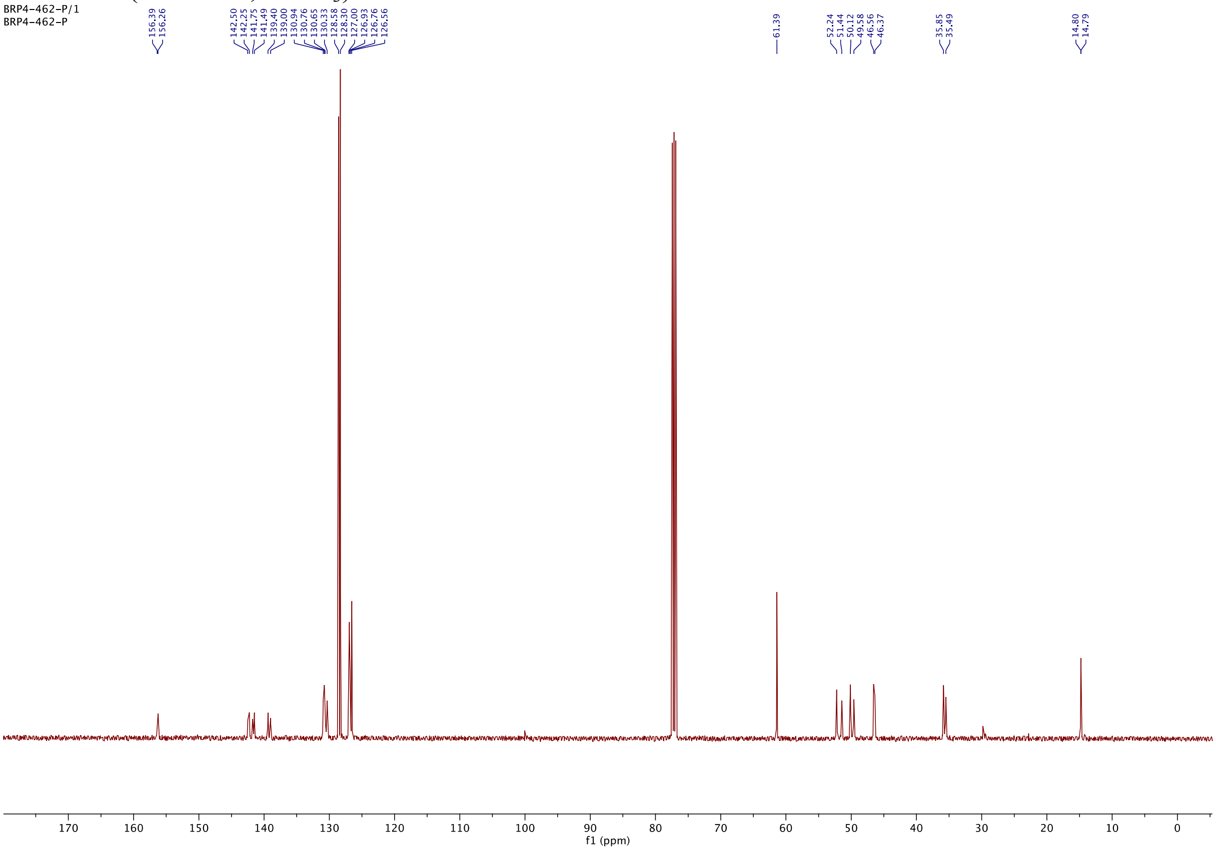

^{1}H NMR (400 MHz, $CDCl_3$)

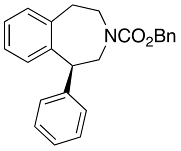

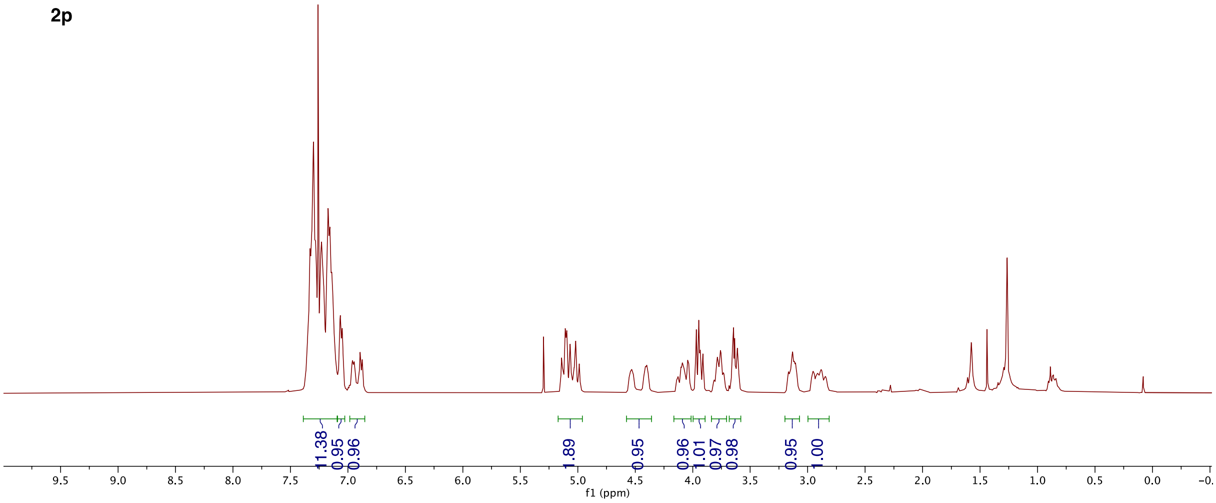

2p

^{13}C NMR (125 MHz, $CDCl_3$)

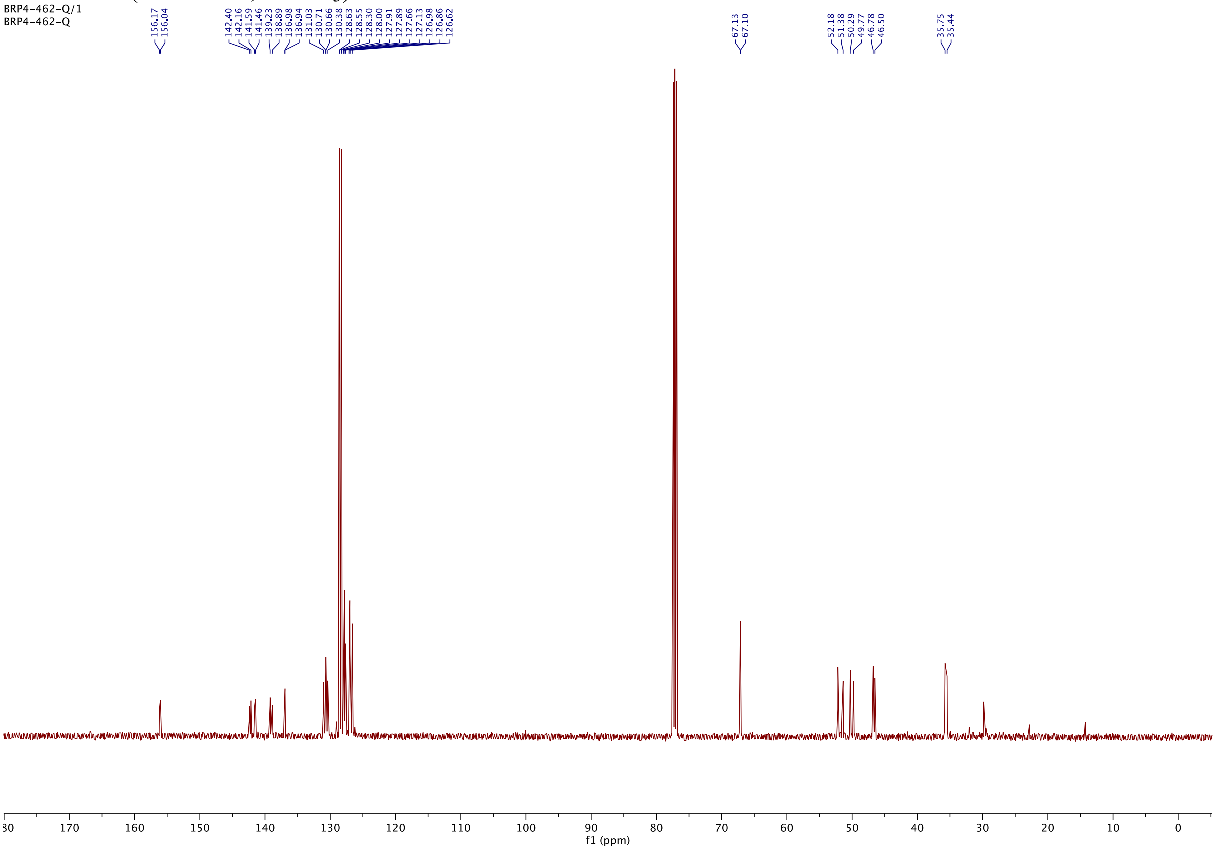

^{1}H NMR (400 MHz, $CDCl_3$)

BRP4-462-R/20

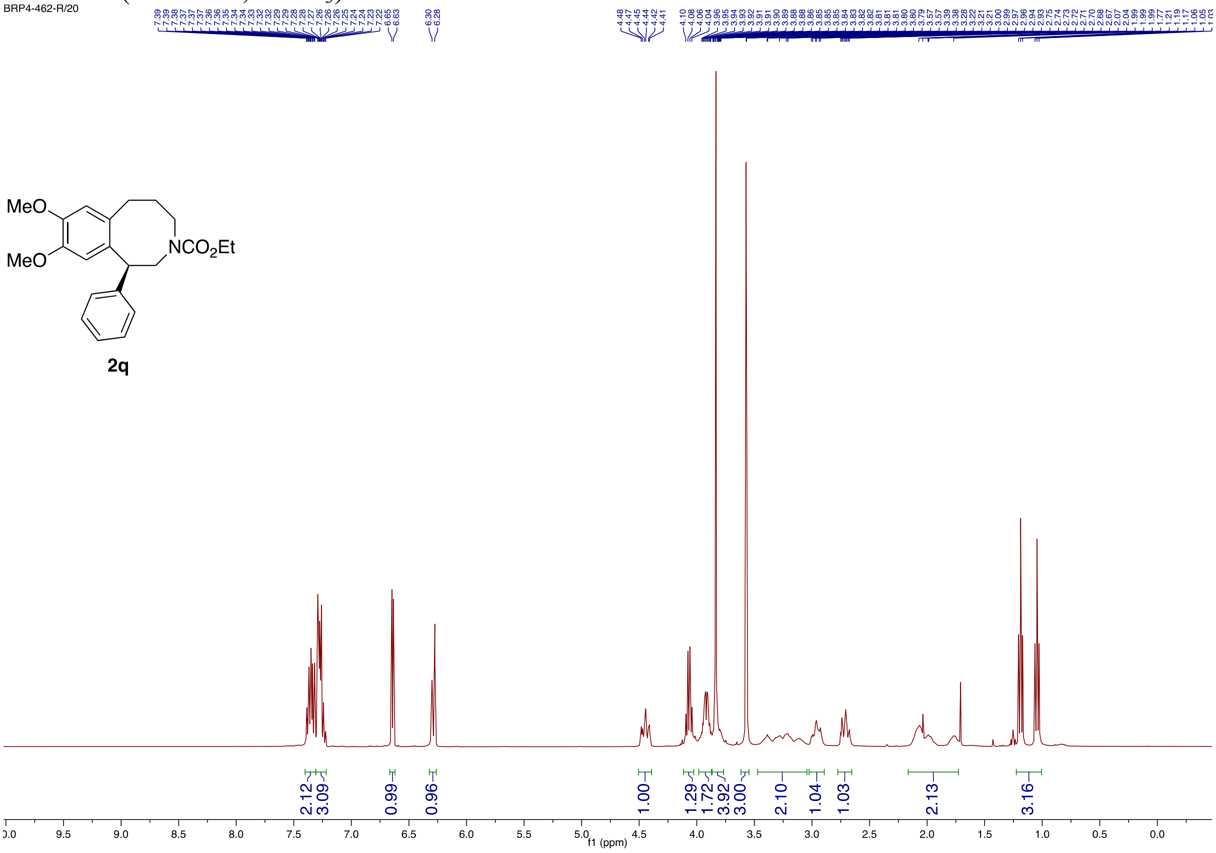

^{13}C NMR (125 MHz, $CDCl_3$)

BRP4-462-R/1
BRP4-462-R

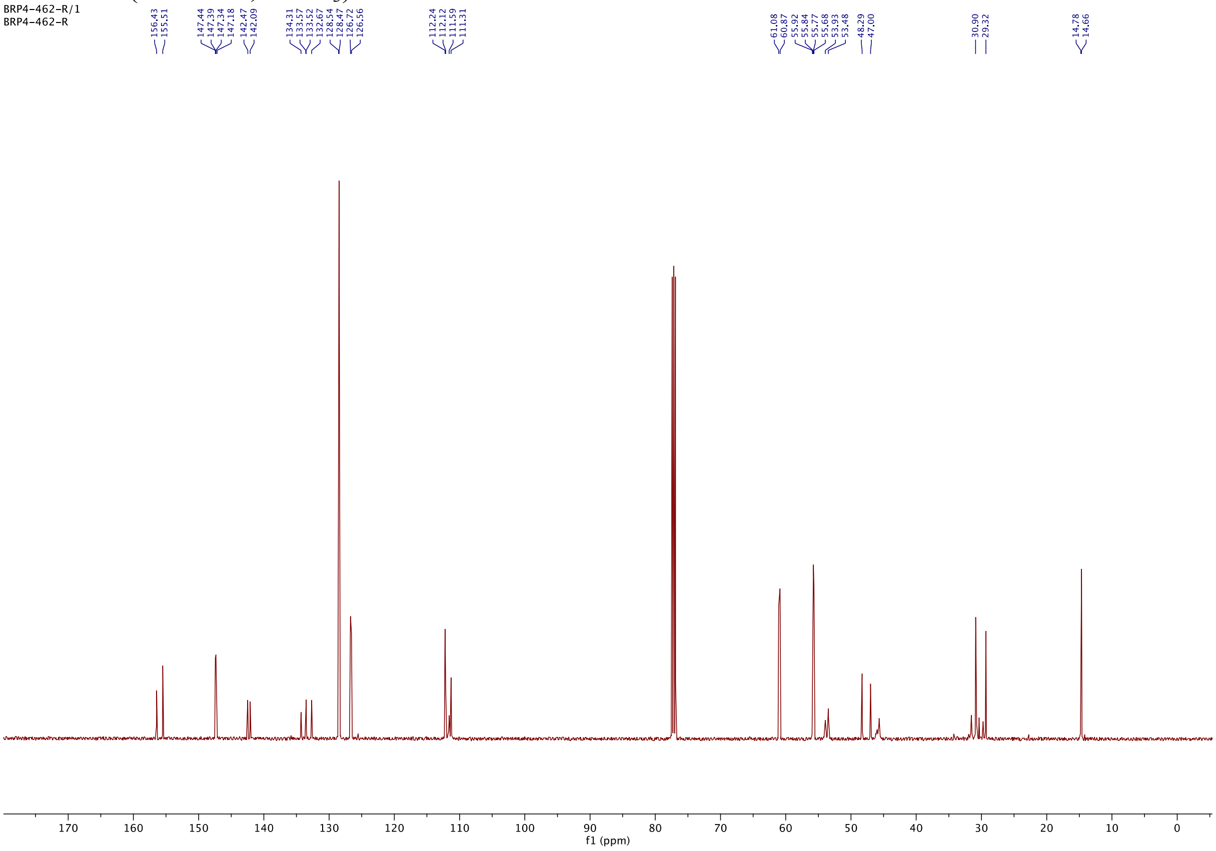

^{1}H NMR (400 MHz, $CDCl_3$)

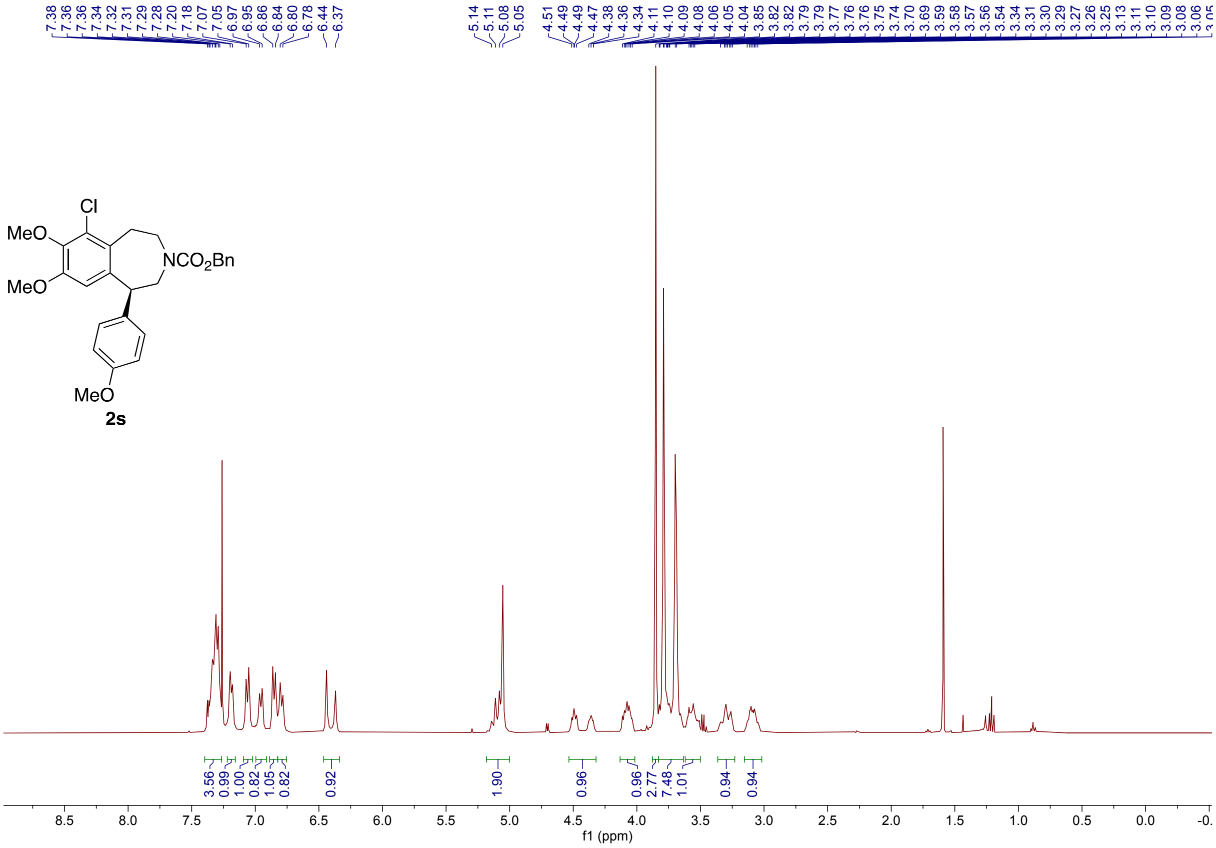

2s

^{13}C NMR (100 MHz, $CDCl_3$)

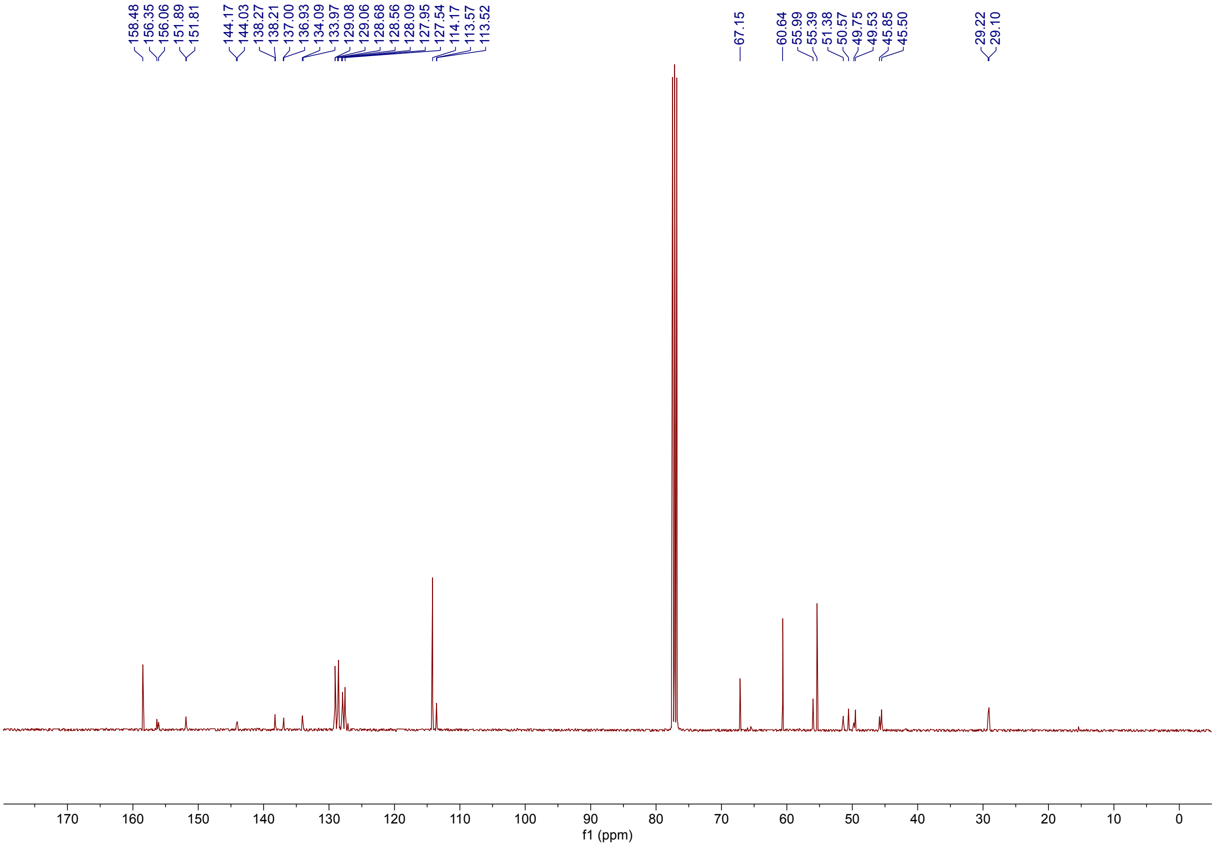

^{1}H NMR (400 MHz, $CDCl_3$)

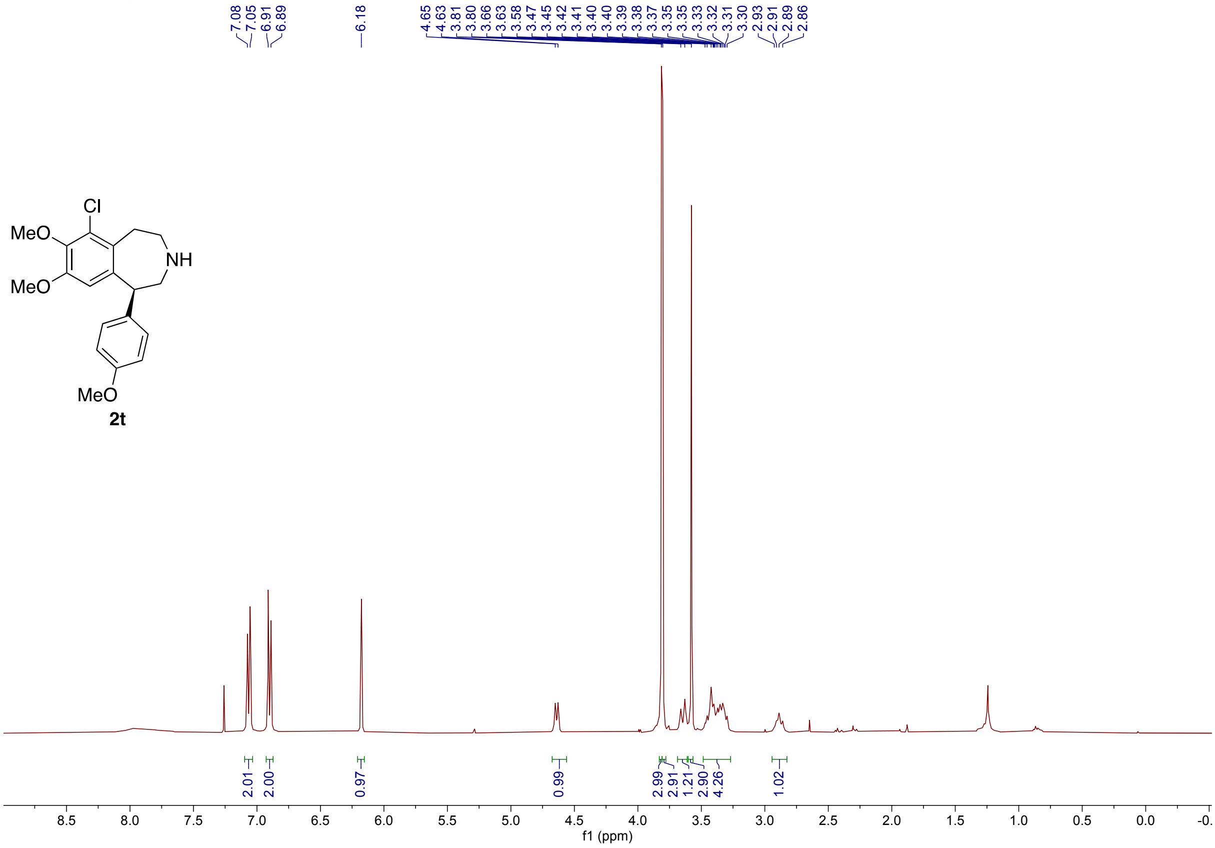

2t

^{13}C NMR (100 MHz, $CDCl_3$)

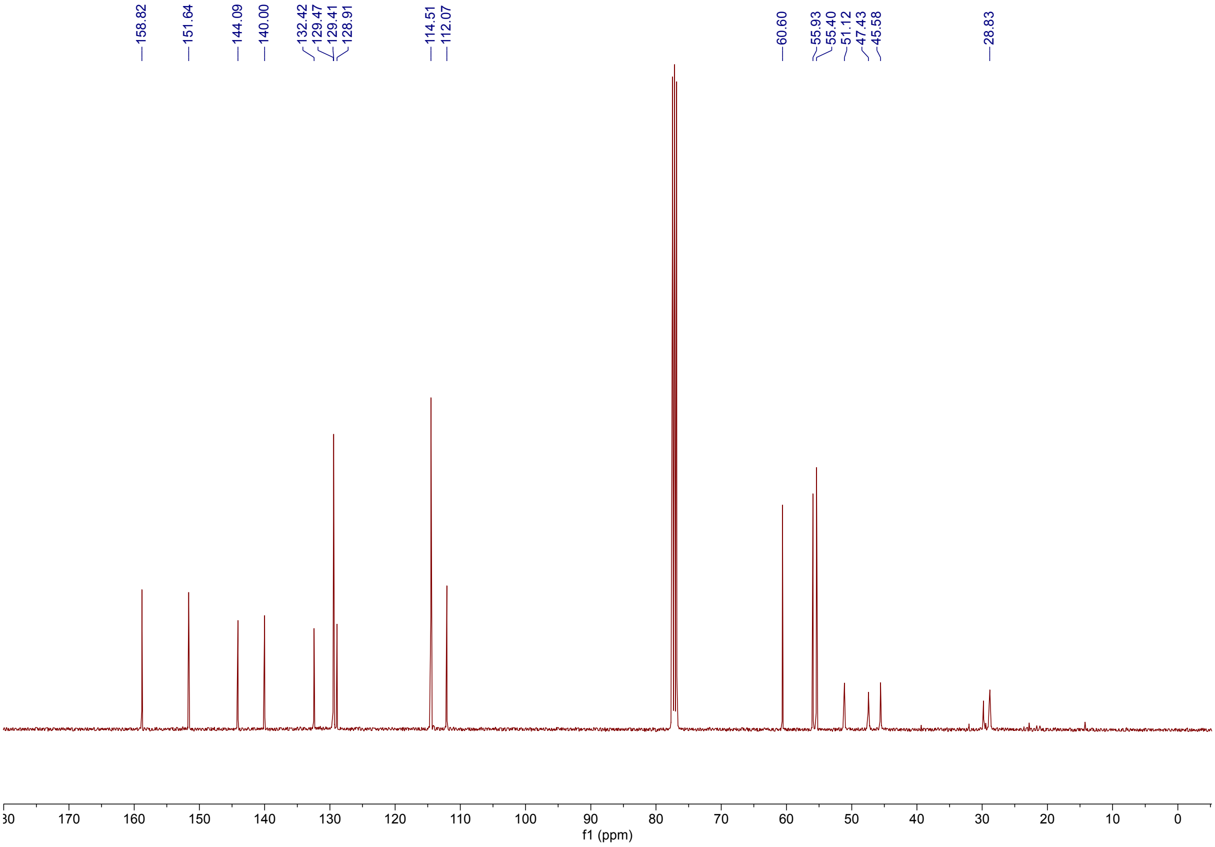

^{1}H NMR (500 MHz, $CDCl_3$)

BRP5-689-K/2
BRP5-689-K 1H

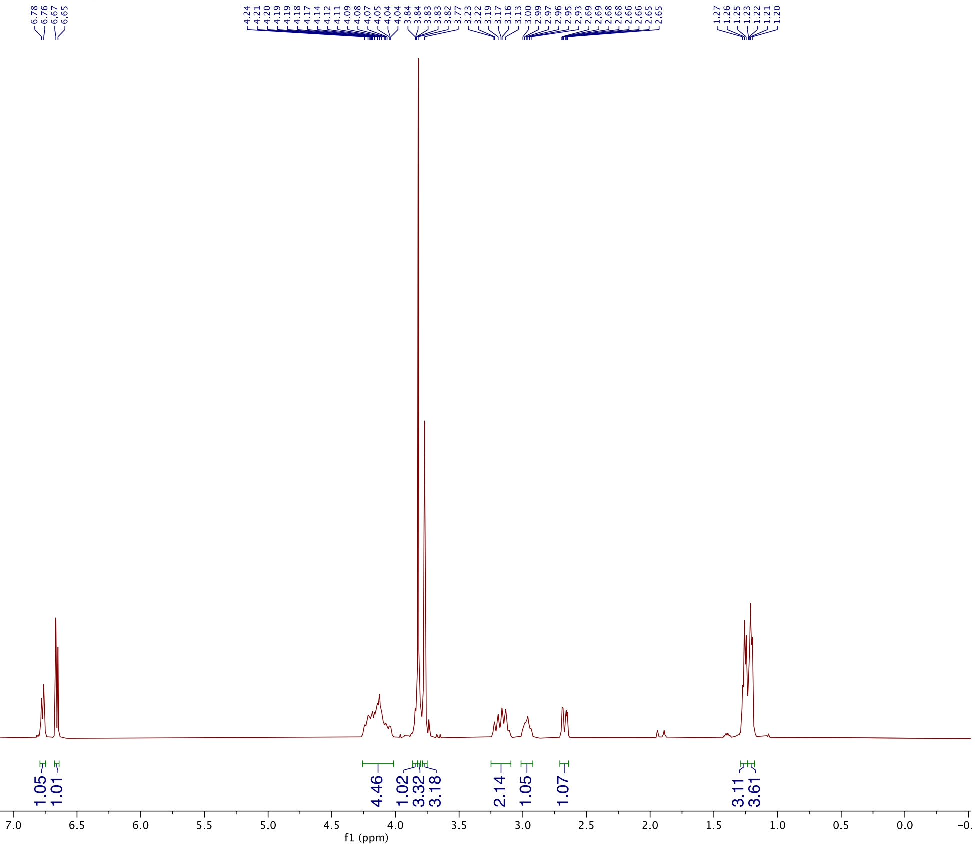

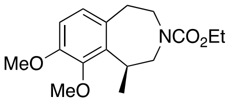

4a

^{13}C NMR (125 MHz, $CDCl_3$)

BRP5-689-K/1
BRP5-689-K 13C

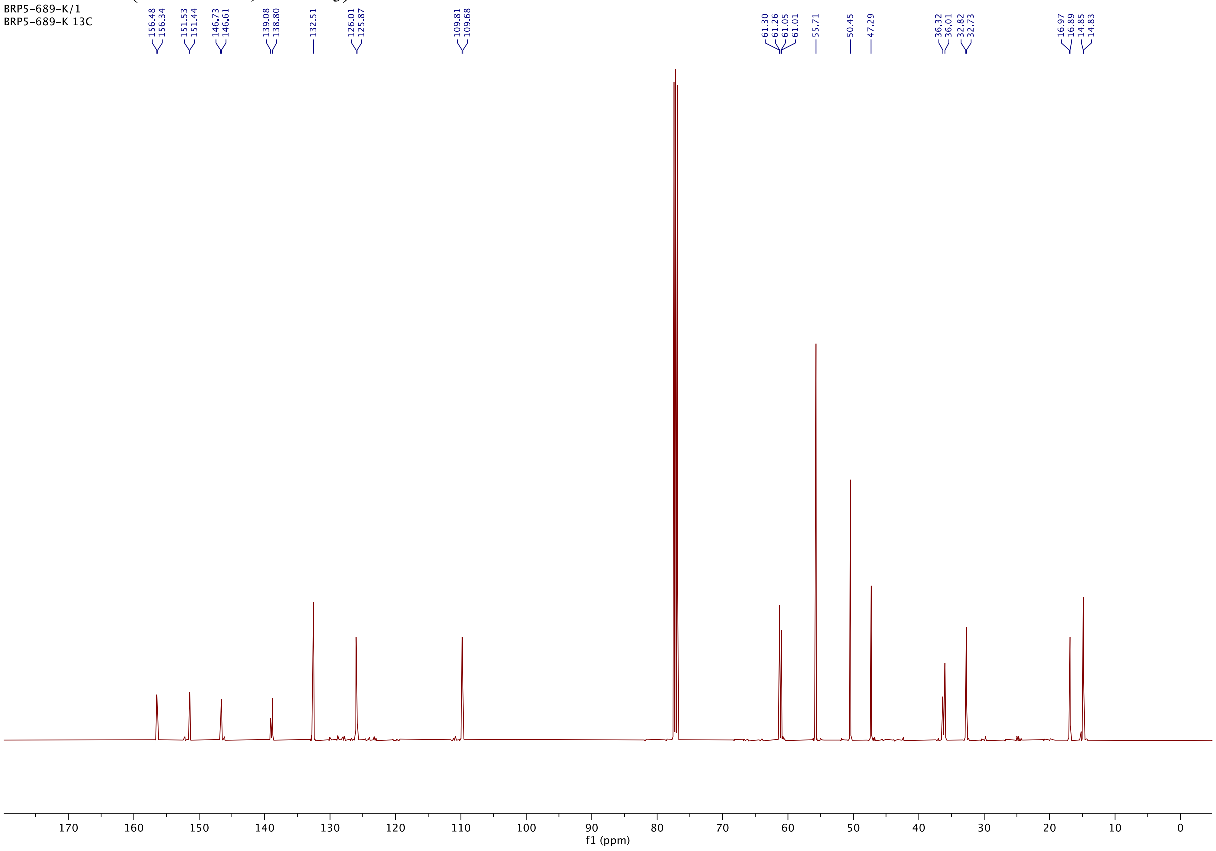

^{1}H NMR (500 MHz, $CDCl_3$)

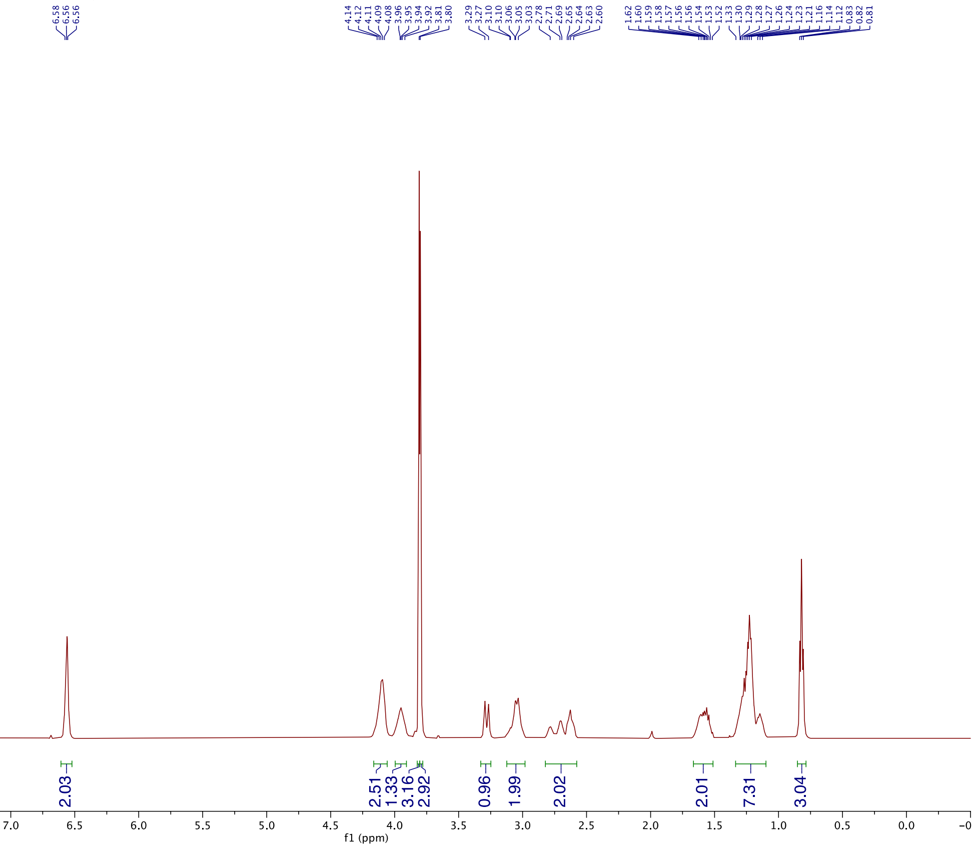

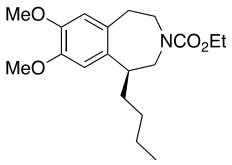

4b

^{13}C NMR (125 MHz, $CDCl_3$)

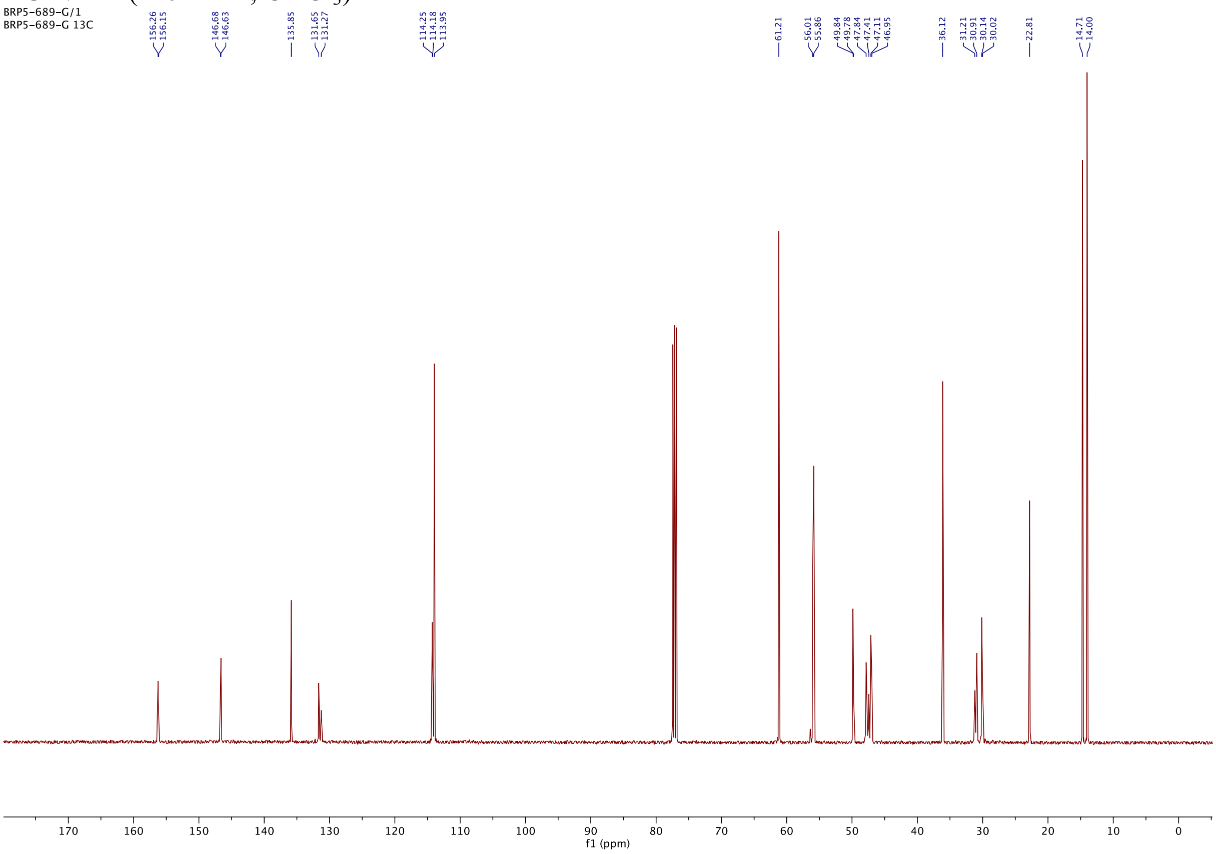

^{1}H NMR (400 MHz, $CDCl_3$)

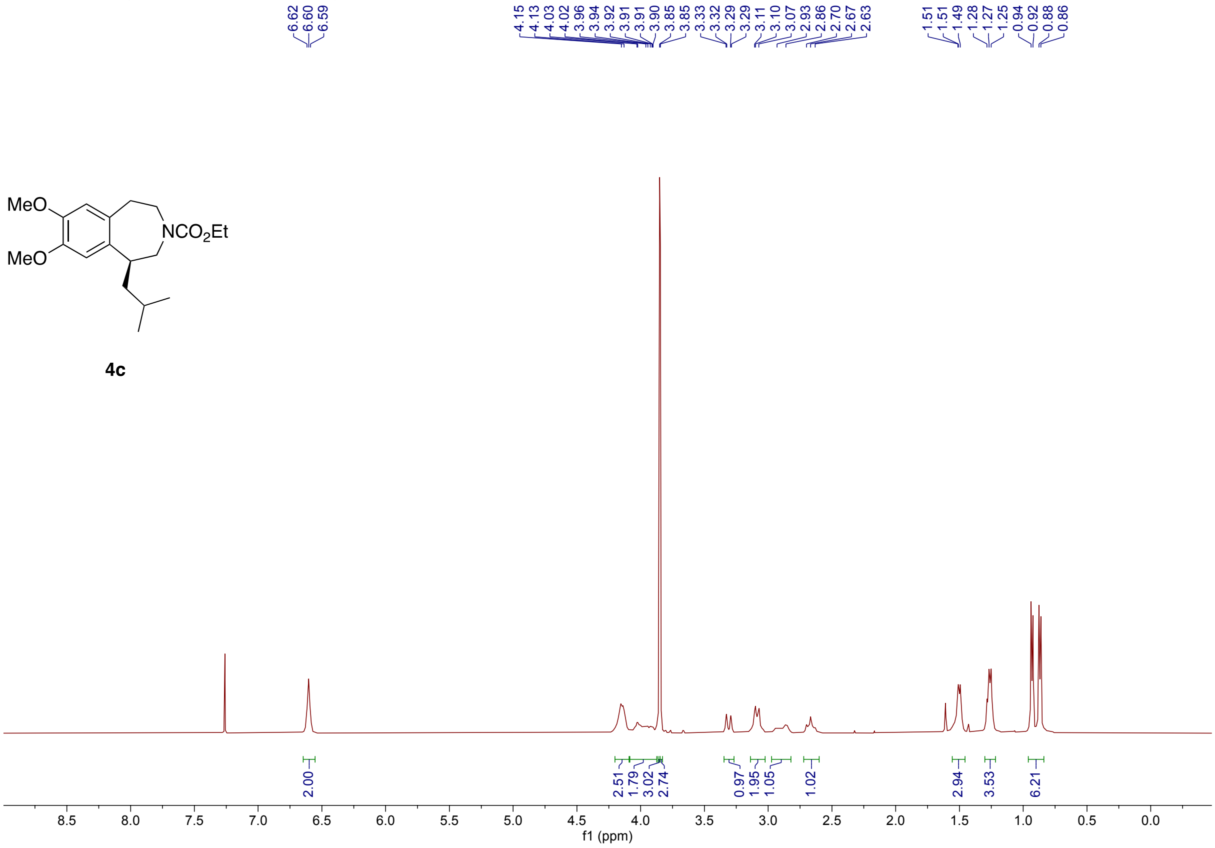

^{13}C NMR (100 MHz, $CDCl_3$)

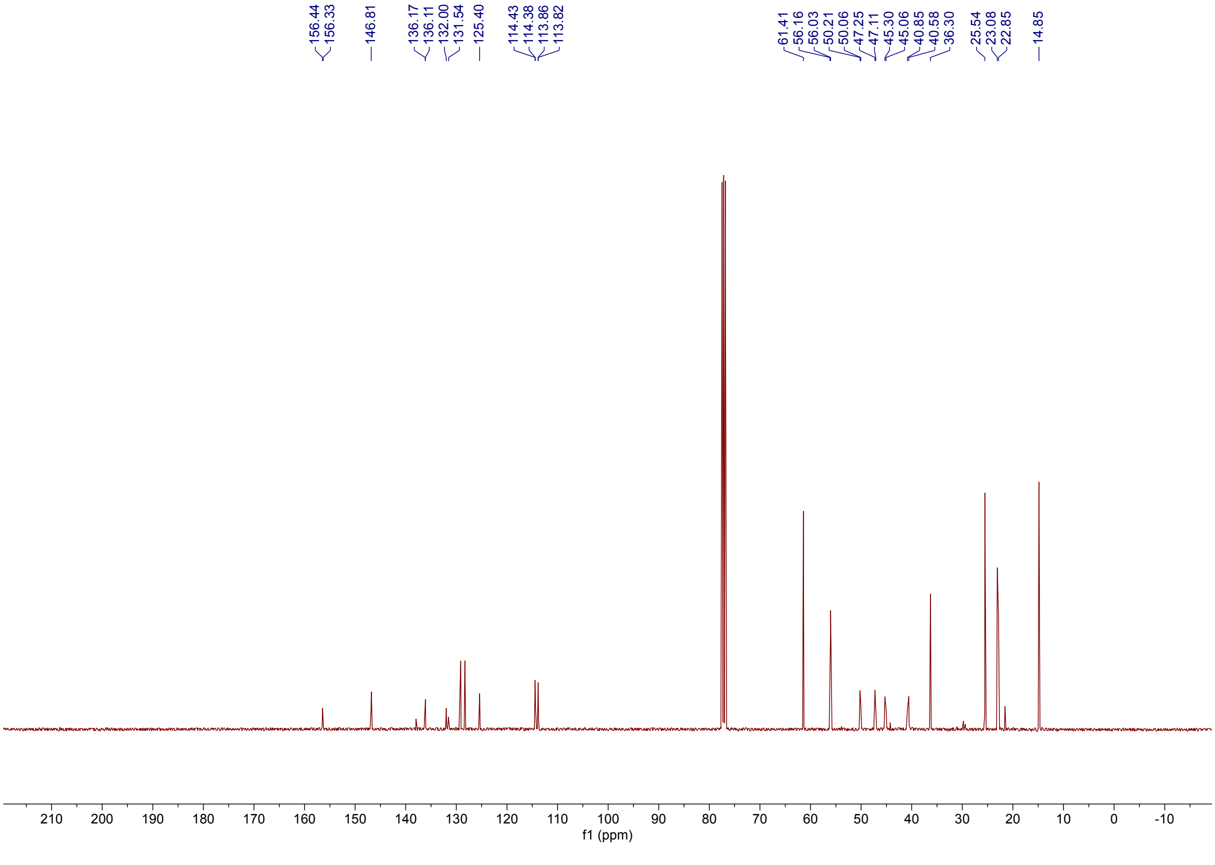

^{1}H NMR (500 MHz, $CDCl_3$)

BRP5-689-I/2
BRP5-689-I 1H

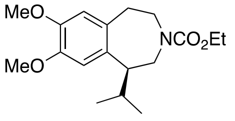

4d

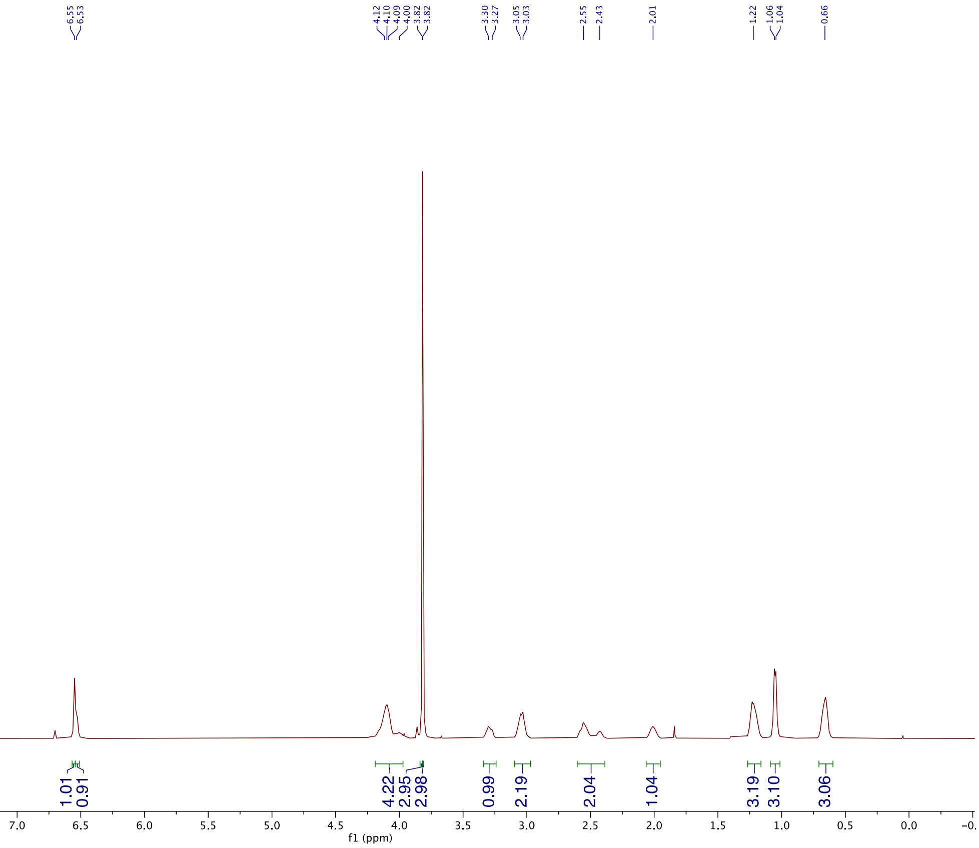

^{13}C NMR (125 MHz, $CDCl_3$)

BRP5-689-I/1
BRP5-689-I 13C

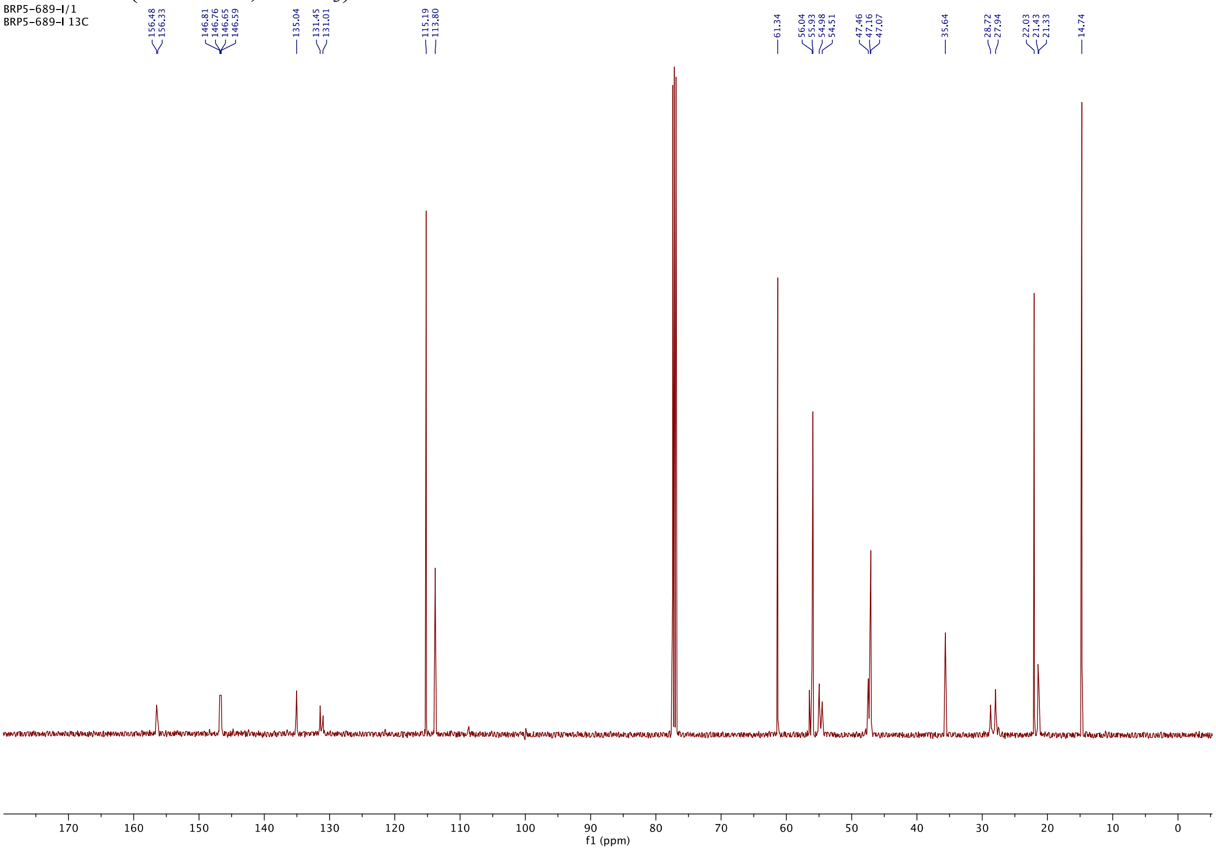

^{1}H NMR (500 MHz, $CDCl_3$)

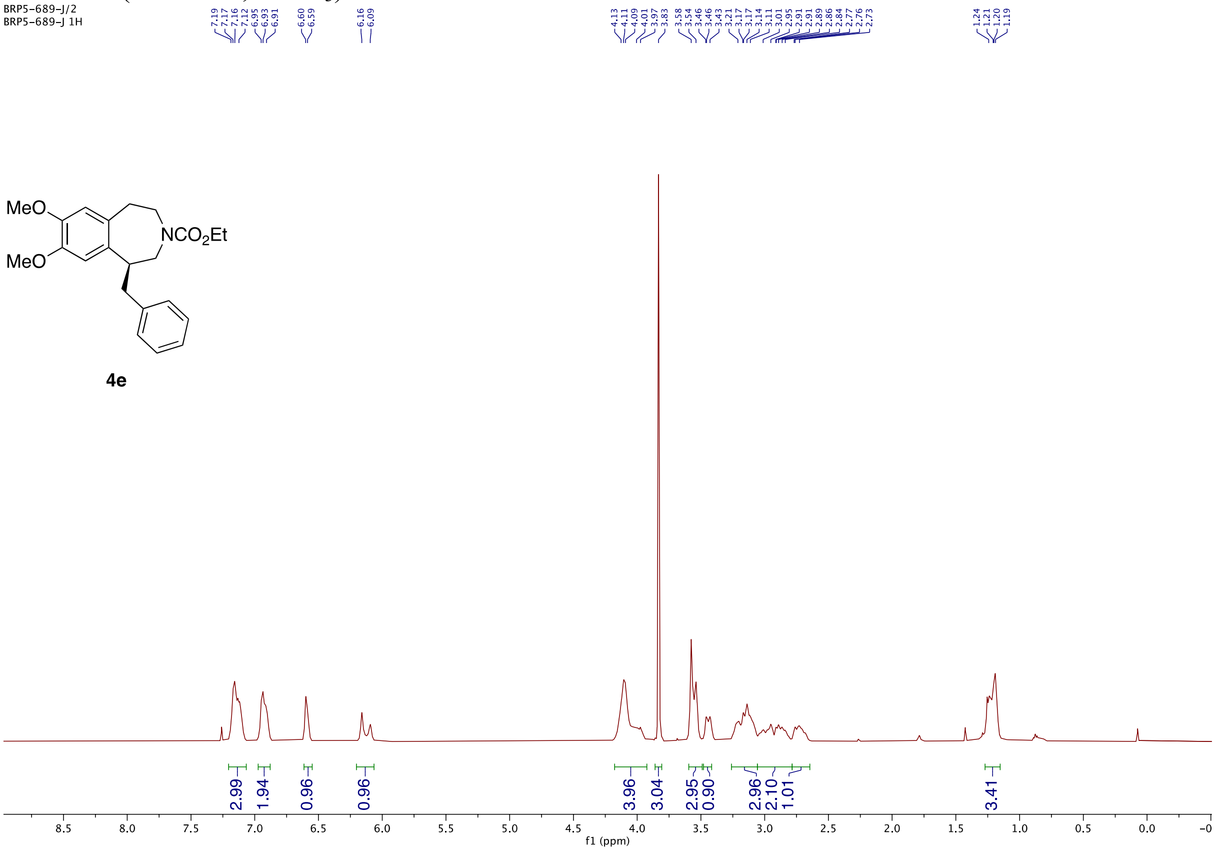

4e

^{13}C NMR (125 MHz, $CDCl_3$)

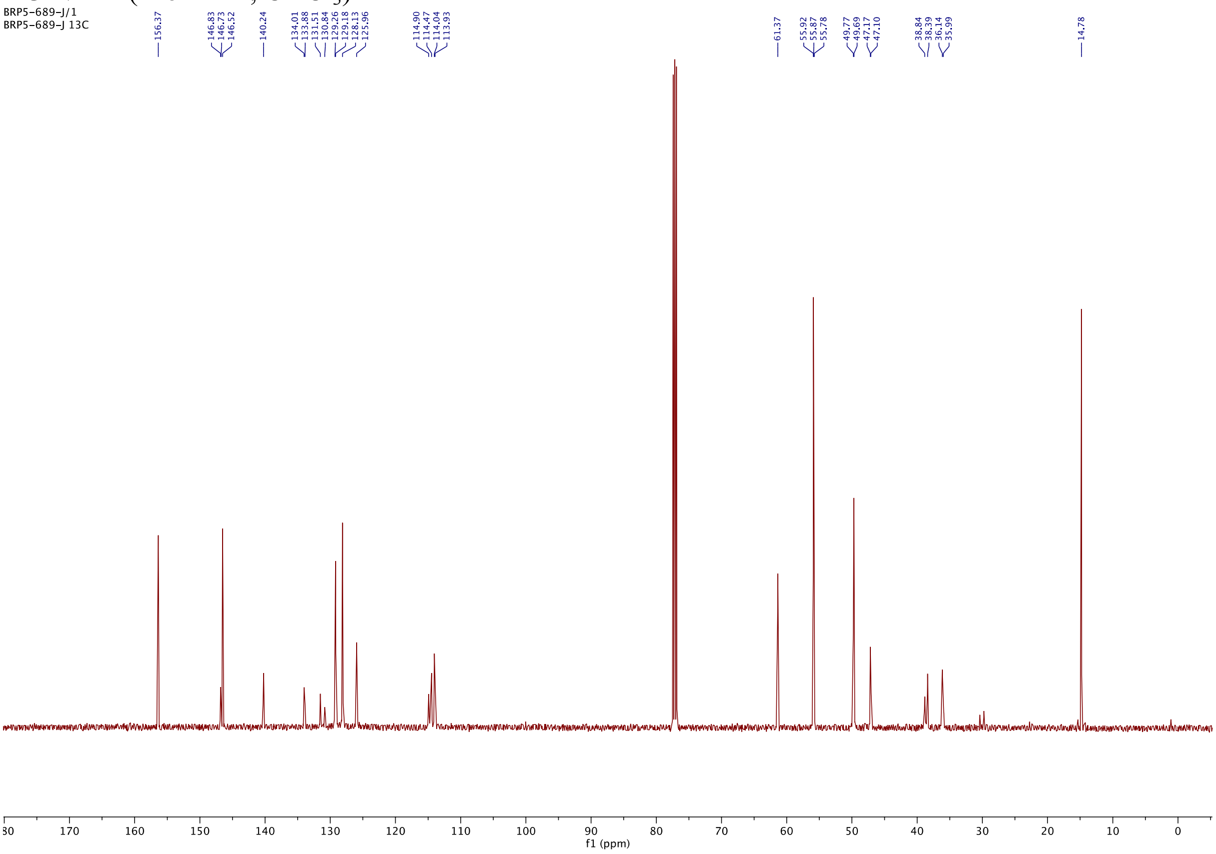

Separation of chiral products

Entry	Product	Separation method	Optical rotation	*ee* (%)
1	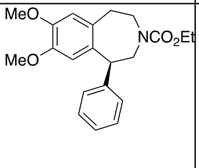 **2a**	SFC: Chiralcel OJ-H column (90% CO_2 10% MeOH), 2.0 mL/min, t_R = 6.91 min (major) / 7.97 min (minor)	$[\alpha]_D^{25}$ = + 33 (c = 0.1, $CHCl_3$)	99
2	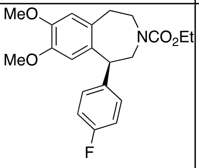 **2b**	SFC: Chiralcel OJ-H column (90% CO_2 10% MeOH), 2.0 mL/min, t_R = 5.70 min (major) / 7.63 min (minor)	$[\alpha]_D^{25}$ = + 37 (c = 0.1, $CHCl_3$)	99
3	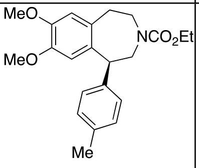 **2c**	SFC: Chiralcel OJ-H column (90% CO_2 10% MeOH), 2.0 mL/min, t_R = 5.73 min (major) / 7.01 min (minor)	$[\alpha]_D^{26}$ = + 29 (c = 0.1, $CHCl_3$)	96
4	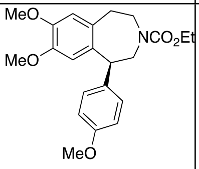 **2d**	SFC: Chiralcel OJ-H column (90% CO_2 10% MeOH), 2.0 mL/min, t_R = 7.93 min (major) / 11.68 min (minor)	$[\alpha]_D^{26}$ = + 30 (c = 0.1, $CHCl_3$)	96
5	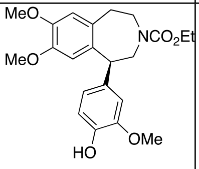 **2e**	SFC: Chiralcel OJ-H column (80% CO_2 20% MeOH), 2.0 mL/min, t_R = 5.40 min (major) / 6.91 min (minor)	$[\alpha]_D^{25}$ = + 35 (c = 0.1, $CHCl_3$)	99
6	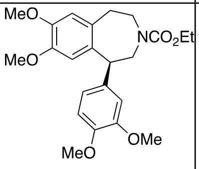 **2f**	SFC: Chiralcel OJ-H column (90% CO_2 10% MeOH), 2.0 mL/min, t_R = 9.88 min (major) / 12.82 min (minor)	$[\alpha]_D^{25}$ = + 31 (c = 0.1, $CHCl_3$)	99
7	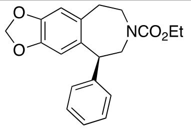 **2g**	SFC: Chiralcel OJ-H column (90% CO_2 10% MeOH), 2.0 mL/min, t_R = 7.48 min (minor) / 8.11 min (major)	$[\alpha]_D^{26}$ = + 18 (c = 0.1, $CHCl_3$)	95
8	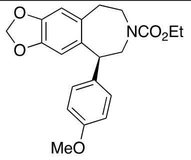 **2h**	SFC: Chiralcel OJ-H column (90% CO_2 10% MeOH), 2.0 mL/min, t_R = 8.13 min (major) / 8.96 min (minor)	$[\alpha]_D^{25}$ = + 14 (c = 0.1, $CHCl_3$)	96
9	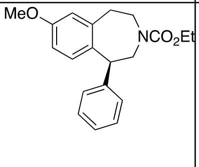 **2i**	SFC: Chiralcel OZ-H column (90% CO_2 10% MeOH), 2.0 mL/min, t_R = 13.38 min (major) / 14.93 min (minor)	$[\alpha]_D^{27}$ = + 27 (c = 0.1, $CHCl_3$)	99

10	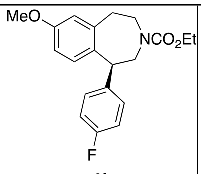 **2j**	SFC: Chiralcel OJ-H column (90% CO_2 10% MeOH), 2.0 mL/min, t_R = 5.25 min (major) / 6.44 min (minor)	$[\alpha]_D^{26}$ = + 31 (c = 0.1, $CHCl_3$)	96
11	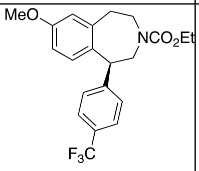 **2k**	SFC: Chiralcel OJ-H column (90% CO_2 10% MeOH), 2.0 mL/min, t_R = 3.81 min (major) / 4.67 min (minor)	$[\alpha]_D^{27}$ = + 27 (c = 0.1, $CHCl_3$)	96
12	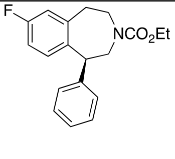 **2l**	SFC: Chiralcel OZ-H column (90% CO_2 10% MeOH), 2.0 mL/min, t_R = 8.11 min (major) / 8.87 min (minor)	$[\alpha]_D^{26}$ = + 44 (c = 0.1, $CHCl_3$)	94
13	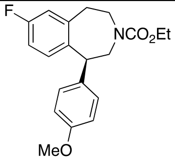 **2m**	SFC: Chiralcel OJ-H column (90% CO_2 10% MeOH), 2.0 mL/min, t_R = 5.70 min (major) / 7.32 min (minor)	$[\alpha]_D^{26}$ = + 37 (c = 0.1, $CHCl_3$)	99
14	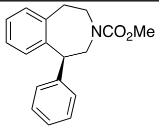 **2n**	SFC: Chiralcel OZ-H column (90% CO_2 10% MeOH), 2.0 mL/min, t_R = 10.92 min (major) / 12.64 min (minor)	$[\alpha]_D^{26}$ = + 54 (c = 0.1, $CHCl_3$)	99
15	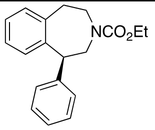 **2o**	SFC: Chiralcel OZ-H column (90% CO_2 10% MeOH), 2.0 mL/min, t_R = 10.45 min (major) / 12.16 min (minor)	$[\alpha]_D^{26}$ = + 51 (c = 0.1, $CHCl_3$)	96
16	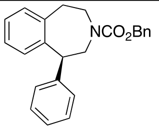 **2p**	SFC: Chiralpak IF column (90% CO_2 10% MeOH), 2.0 mL/min, t_R = 10.98 min (major) / 11.91 min (minor)	$[\alpha]_D^{26}$ = + 47 (c = 0.1, $CHCl_3$)	97
17	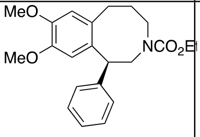 **2q**	SFC: Chiralcel OJ-H column (90% CO_2 10% MeOH), 2.0 mL/min, t_R = 5.36 min (minor) / 6.13 min (major)	$[\alpha]_D^{27}$ = - 98 (c = 0.1, $CHCl_3$)	95
18	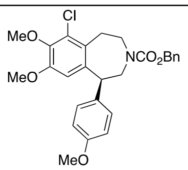 **2s**	SFC: Chiralpak IA column (90% CO_2 10% MeOH), 2.0 mL/min, t_R = 10.53 min (major) / 11.72 min (minor)	$[\alpha]_D^{27}$ = + 10 (c = 0.1, $CHCl_3$)	99
19	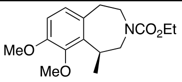 **4a**	SFC: Chiralcel OJ-H column (90% CO_2 10% MeOH), 2.0 mL/min, t_R = 5.61 min (minor) / 6.35 min (major)	$[\alpha]_D^{25}$ = - 33 (c = 0.1, $CHCl_3$)	91

20	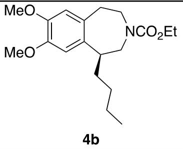 4b	SFC: Chiralcel OJ-H column (90% CO_2 10% MeOH), 2.0 mL/min, t_R = 4.86 min (minor) / 5.31 min (major)	$[\alpha]_D^{24}$ = - 2 (c = 0.1, $CHCl_3$)	99
21	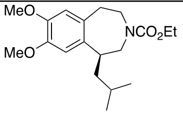 4c	SFC: Chiralcel OJ-H column (90% CO_2 10% MeOH), 2.0 mL/min, t_R = 4.04 min (mmajor) / 4.38 min (minor)	$[\alpha]_D^{24}$ = + 3 (c = 0.1, $CHCl_3$)	94
22	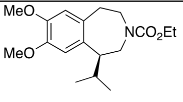 4d	SFC: Chiralcel OJ-H column (90% CO_2 10% MeOH), 2.0 mL/min, t_R = 4.92 min (major) / 5.73 min (minor)	$[\alpha]_D^{24}$ = + 2 (c = 0.1, $CHCl_3$)	93
23	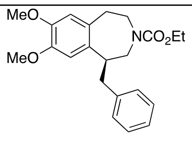 4e	SFC: Chiralpak AS-H column (90% CO_2 10% MeOH), 2.0 mL/min, t_R = 8.51 min (major) / 9.59 min (minor)	$[\alpha]_D^{25}$ = + 49 (c = 0.1, $CHCl_3$)	99

Chromatograms

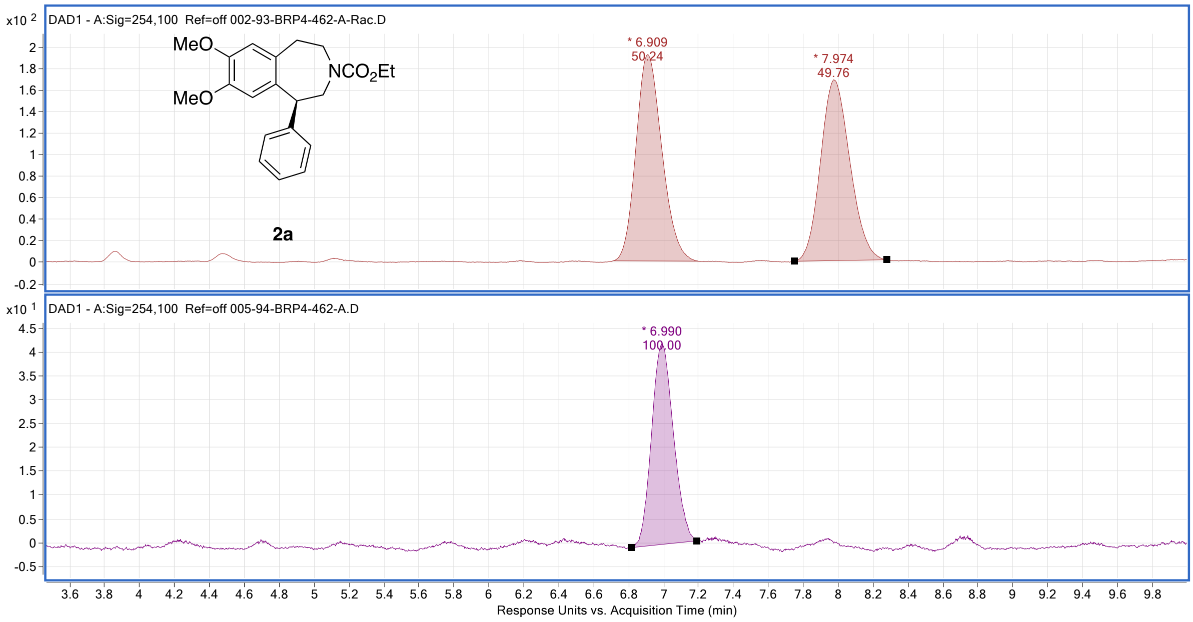

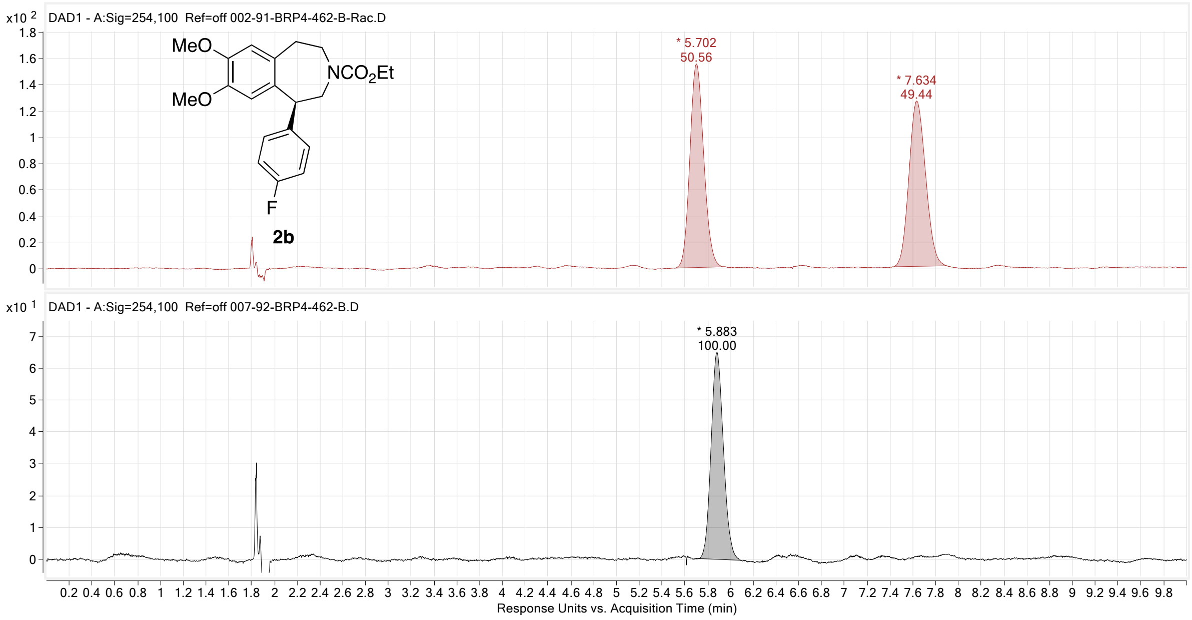

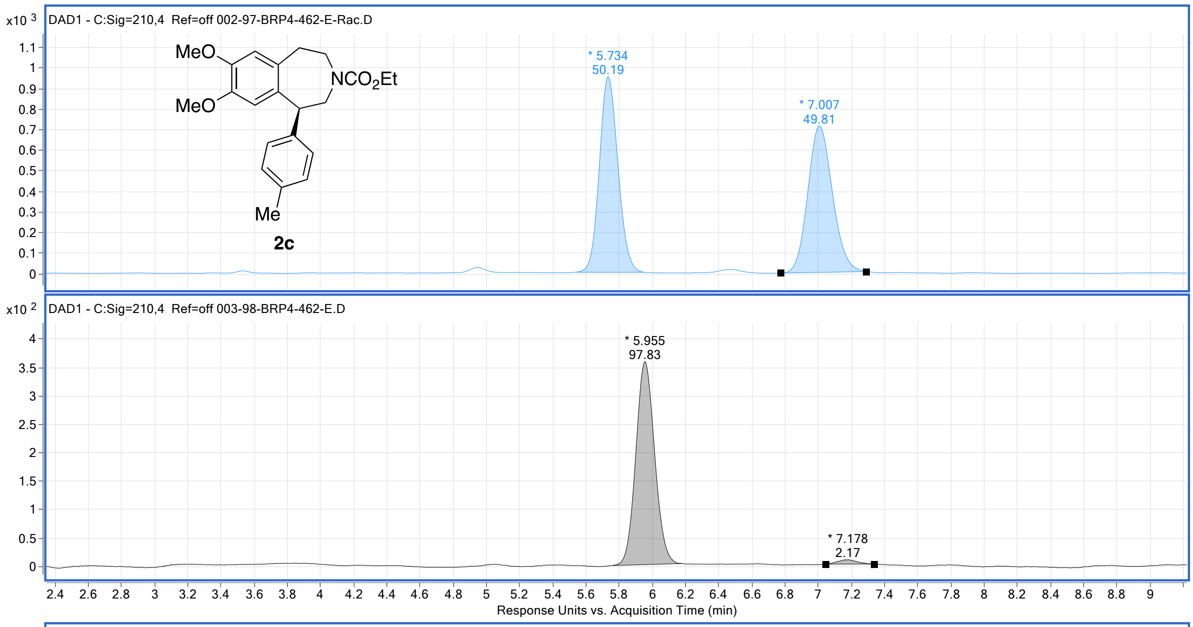
x10 3
DAD1 - C:Sig=210,4 Ref=off 002-97-BRP4-462-E-Rac.D
MeO
MeO
NCO2Et
Me
2c
* 5.734
50.19
* 7.007
49.81
x10 2
DAD1 - C:Sig=210,4 Ref=off 003-98-BRP4-462-E.D
* 5.955
97.83
* 7.178
2.17
Response Units vs. Acquisition Time (min)

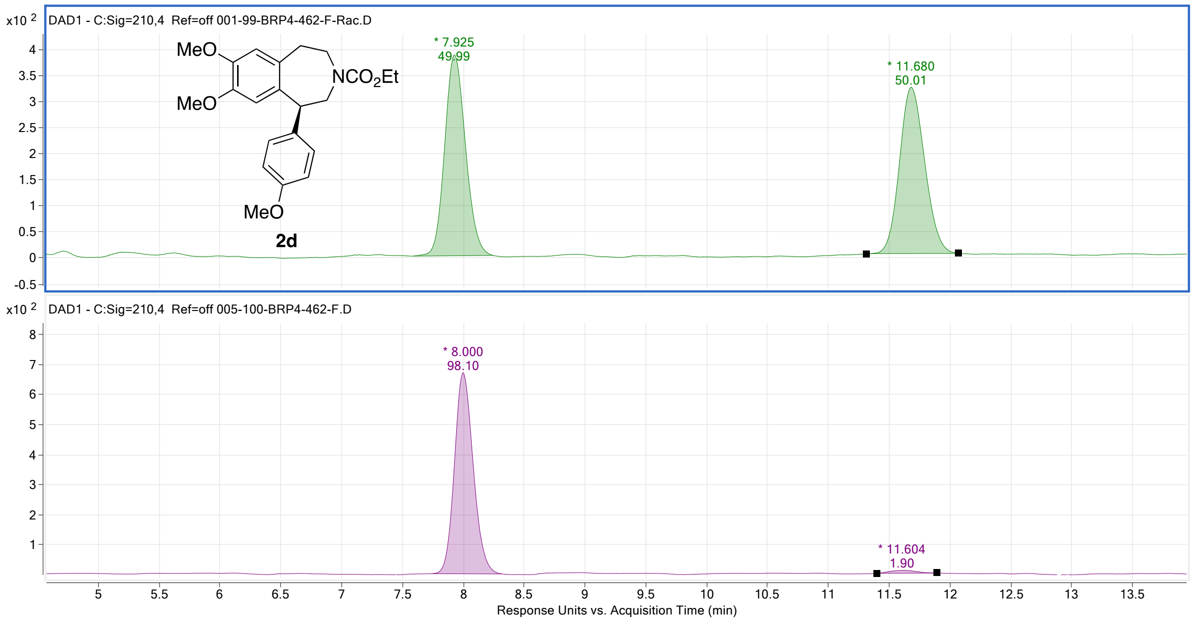
x10 2
DAD1 - C:Sig=210,4 Ref=off 001-99-BRP4-462-F-Rac.D
MeO
MeO
NCO2Et
MeO
2d
* 7.925
49.99
* 11.680
50.01
x10 2
DAD1 - C:Sig=210,4 Ref=off 005-100-BRP4-462-F.D
* 8.000
98.10
* 11.604
1.90
Response Units vs. Acquisition Time (min)

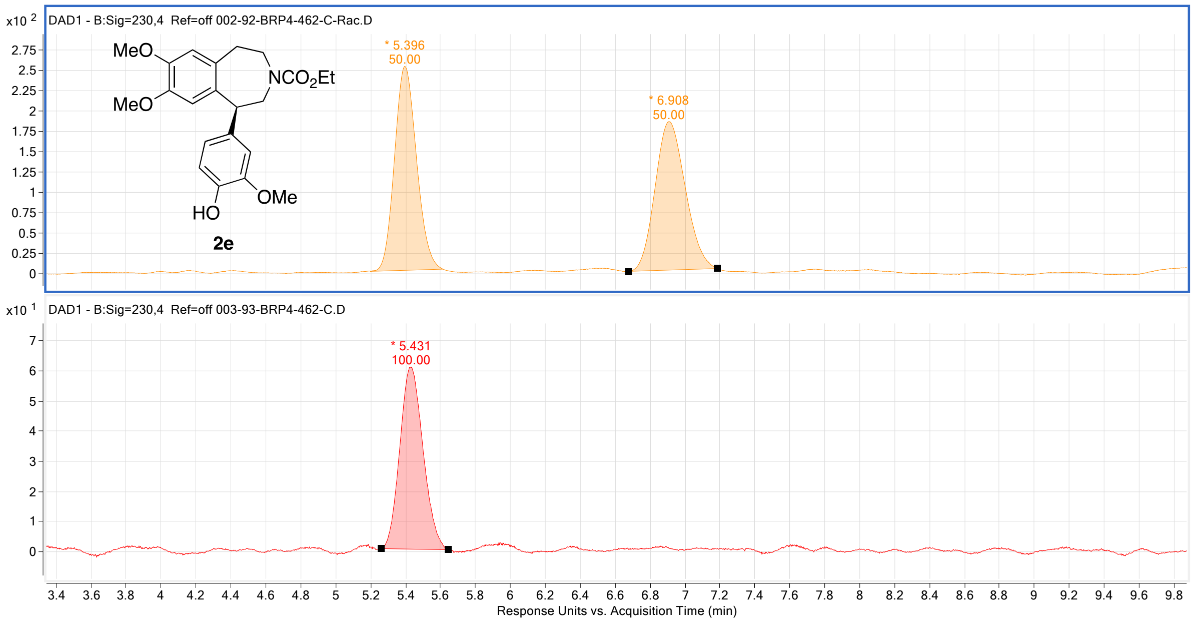
x10 2
DAD1 - B:Sig=230,4 Ref=off 002-92-BRP4-462-C-Rac.D
MeO
MeO
NCO2Et
HO
OMe
2e
* 5.396
50.00
* 6.908
50.00
x10 1
DAD1 - B:Sig=230,4 Ref=off 003-93-BRP4-462-C.D
* 5.431
100.00
Response Units vs. Acquisition Time (min)

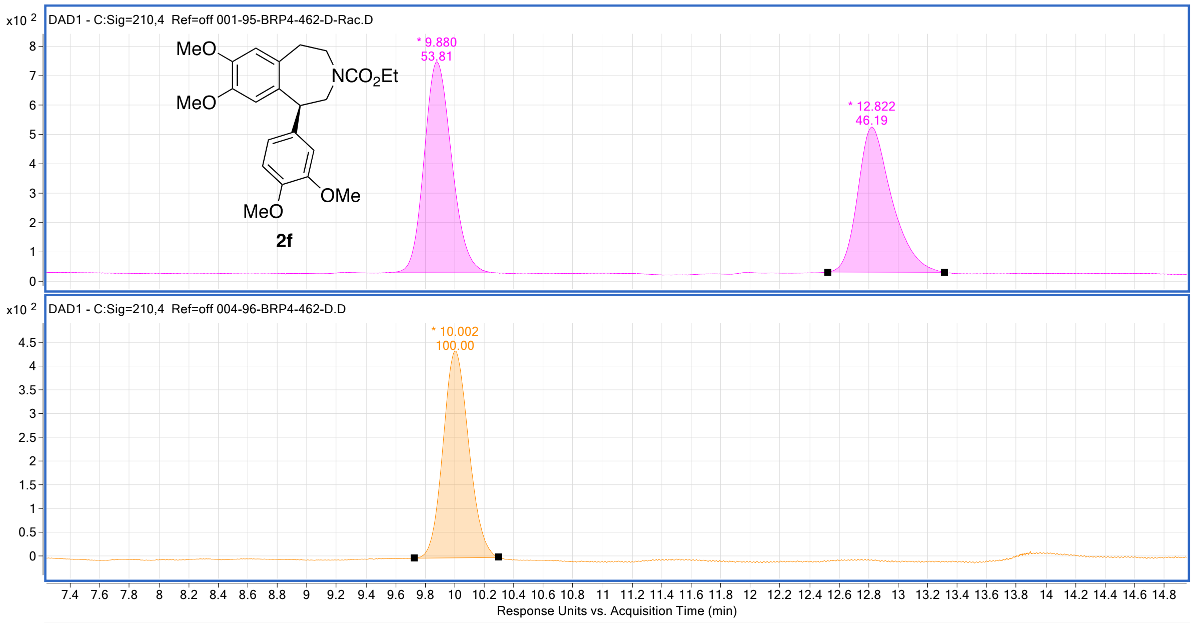
DAD1 - C:Sig=210,4 Ref=off 001-95-BRP4-462-D-Rac.D
* 9.880
53.81
* 12.822
46.19
MeO
MeO
NCO2Et
OMe
MeO
2f
DAD1 - C:Sig=210,4 Ref=off 004-96-BRP4-462-D.D
* 10.002
100.00
Response Units vs. Acquisition Time (min)

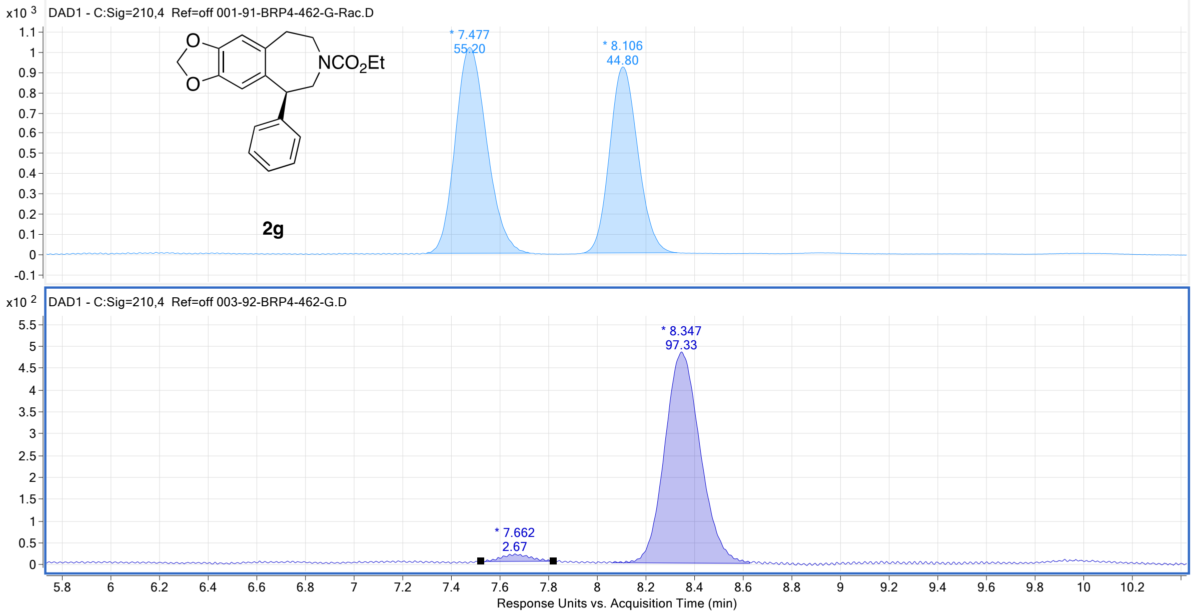
DAD1 - C:Sig=210,4 Ref=off 001-91-BRP4-462-G-Rac.D
* 7.477
55.20
* 8.106
44.80
O
O
NCO2Et
2g
DAD1 - C:Sig=210,4 Ref=off 003-92-BRP4-462-G.D
* 8.347
97.33
* 7.662
2.67
Response Units vs. Acquisition Time (min)

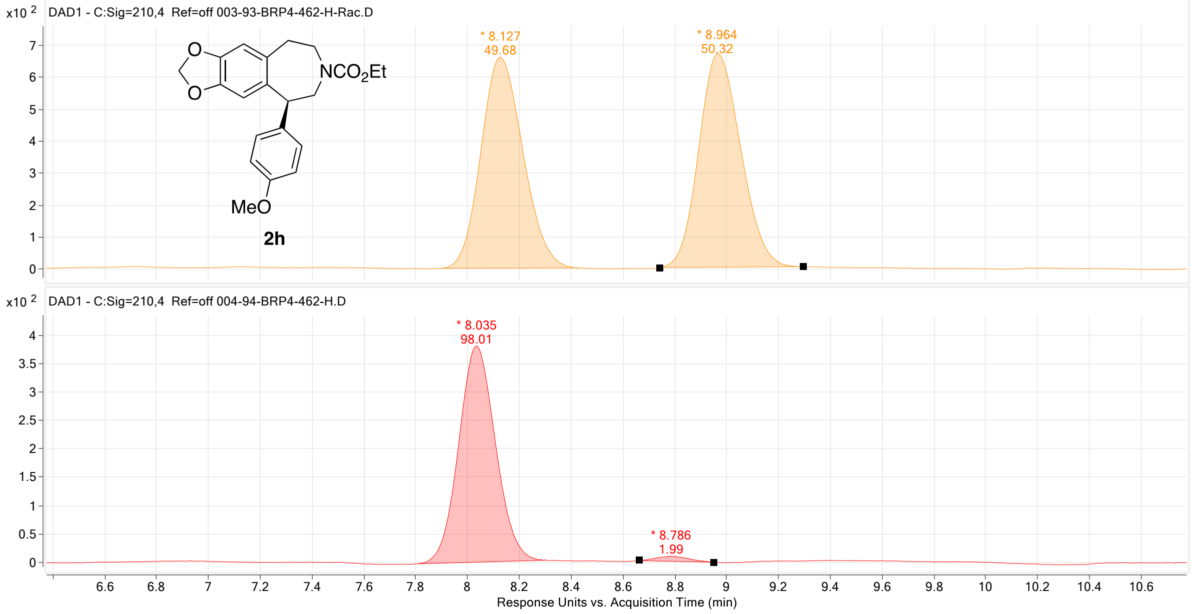
DAD1 - C:Sig=210,4 Ref=off 003-93-BRP4-462-H-Rac.D
* 8.127
49.68
* 8.964
50.32
O
O
NCO2Et
MeO
2h
DAD1 - C:Sig=210,4 Ref=off 004-94-BRP4-462-H.D
* 8.035
98.01
* 8.786
1.99
Response Units vs. Acquisition Time (min)

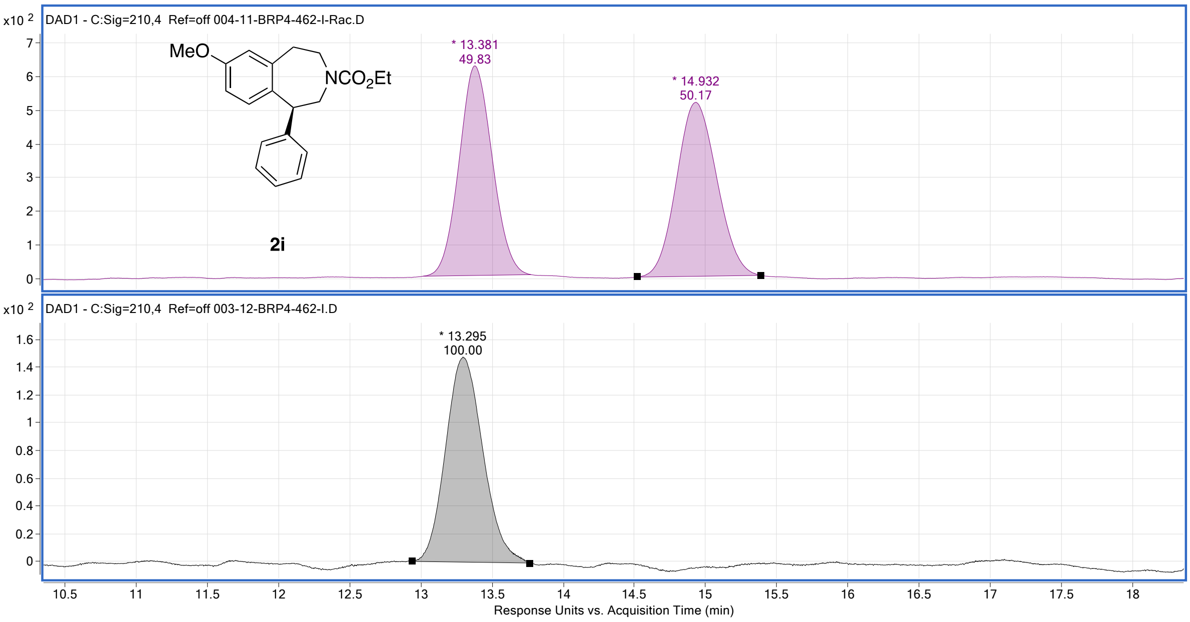
x10 2
DAD1 - C:Sig=210,4 Ref=off 004-11-BRP4-462-I-Rac.D
MeO
NCO2Et
2i
* 13.381
49.83
* 14.932
50.17
DAD1 - C:Sig=210,4 Ref=off 003-12-BRP4-462-I.D
* 13.295
100.00
Response Units vs. Acquisition Time (min)

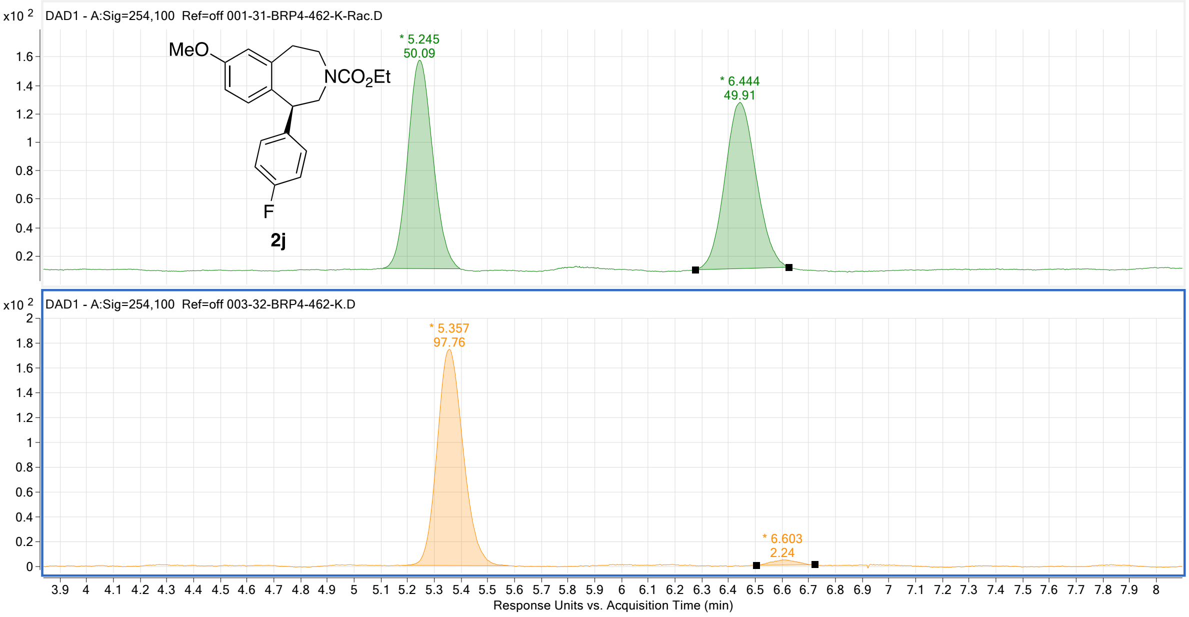
x10 2
DAD1 - A:Sig=254,100 Ref=off 001-31-BRP4-462-K-Rac.D
MeO
NCO2Et
F
2j
* 5.245
50.09
* 6.444
49.91
DAD1 - A:Sig=254,100 Ref=off 003-32-BRP4-462-K.D
* 5.357
97.76
* 6.603
2.24
Response Units vs. Acquisition Time (min)

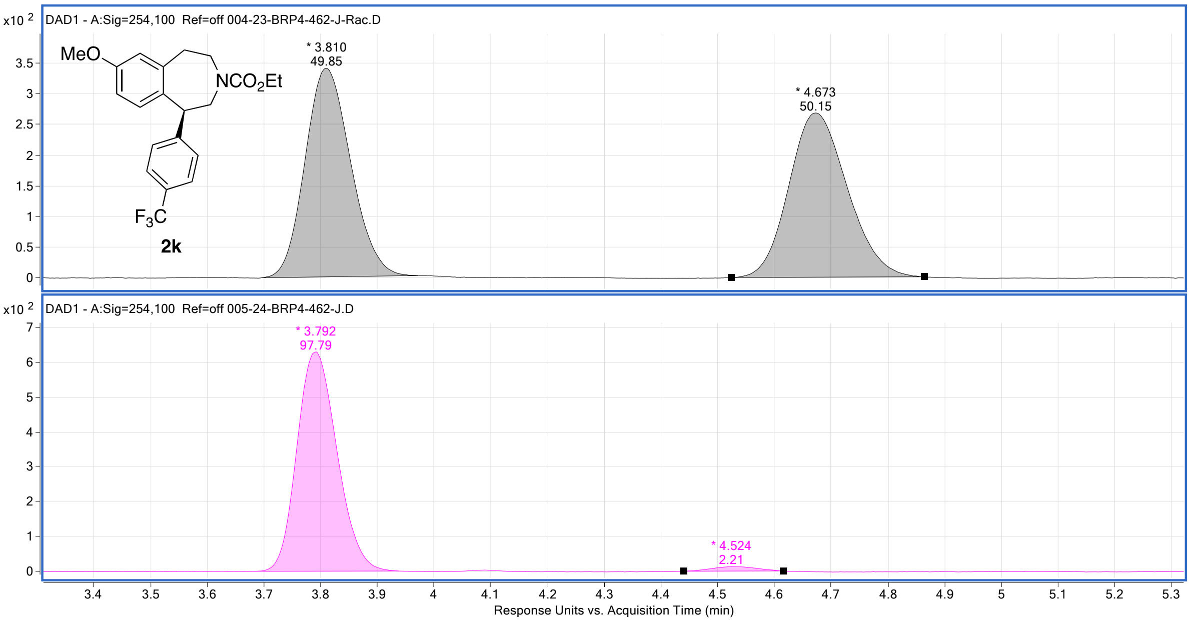
x10 2
DAD1 - A:Sig=254,100 Ref=off 004-23-BRP4-462-J-Rac.D
MeO
NCO2Et
F3C
2k
* 3.810
49.85
* 4.673
50.15
DAD1 - A:Sig=254,100 Ref=off 005-24-BRP4-462-J.D
* 3.792
97.79
* 4.524
2.21
Response Units vs. Acquisition Time (min)

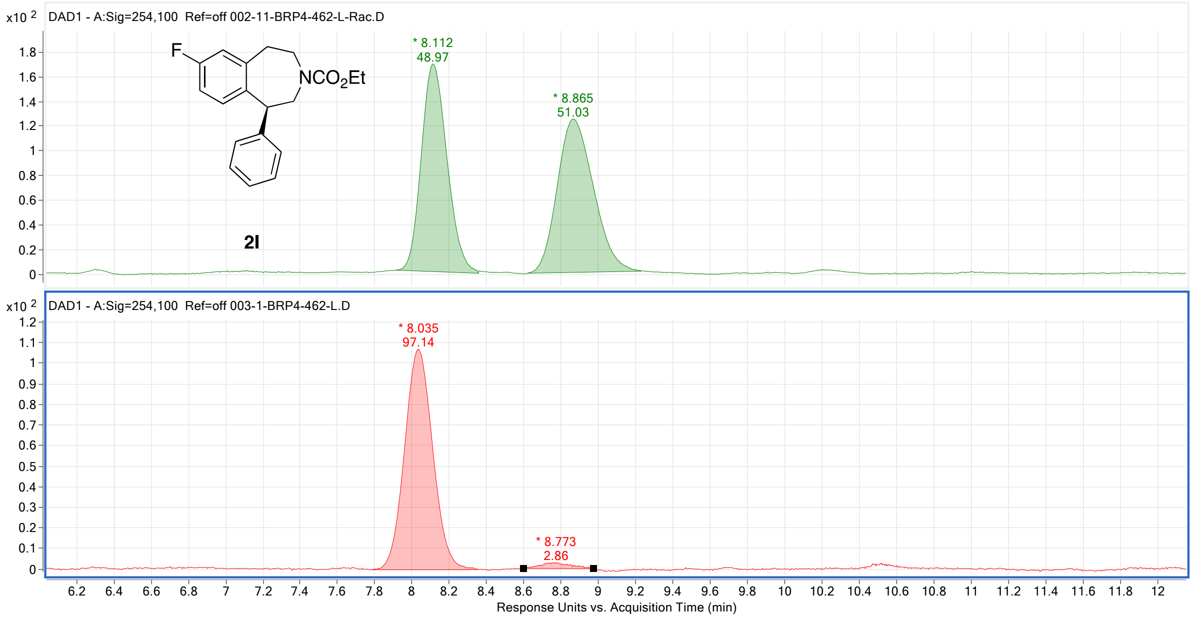
x10 2
DAD1 - A:Sig=254,100 Ref=off 002-11-BRP4-462-L-Rac.D
F
NCO2Et
2l
* 8.112
48.97
* 8.865
51.03
x10 2
DAD1 - A:Sig=254,100 Ref=off 003-1-BRP4-462-L.D
* 8.035
97.14
* 8.773
2.86
Response Units vs. Acquisition Time (min)

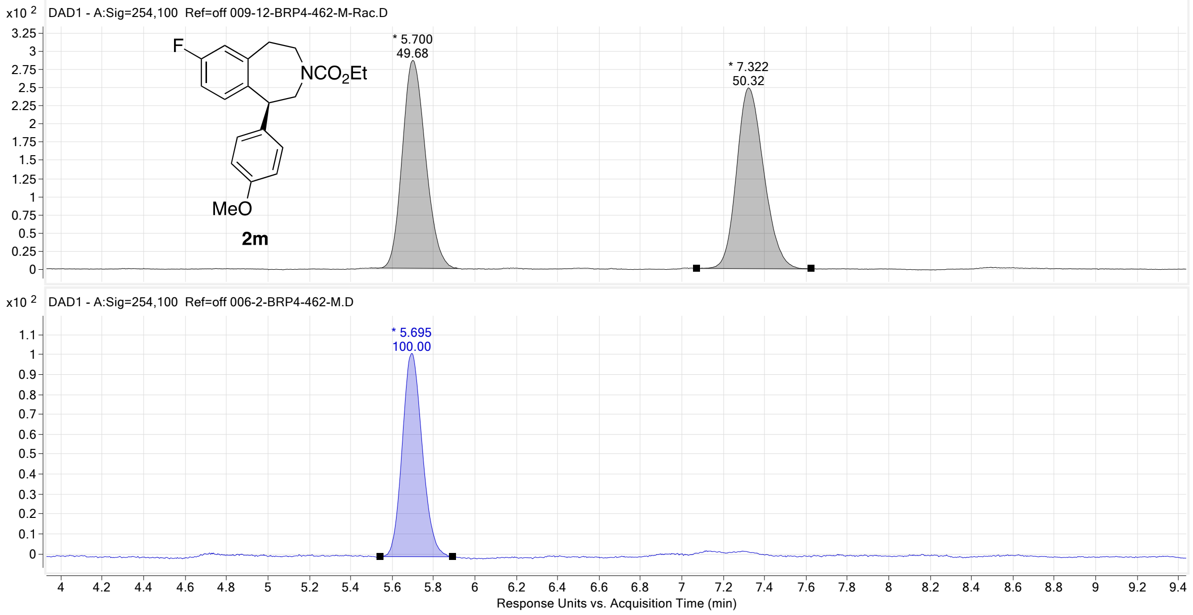
x10 2
DAD1 - A:Sig=254,100 Ref=off 009-12-BRP4-462-M-Rac.D
F
NCO2Et
MeO
2m
* 5.700
49.68
* 7.322
50.32
x10 2
DAD1 - A:Sig=254,100 Ref=off 006-2-BRP4-462-M.D
* 5.695
100.00
Response Units vs. Acquisition Time (min)

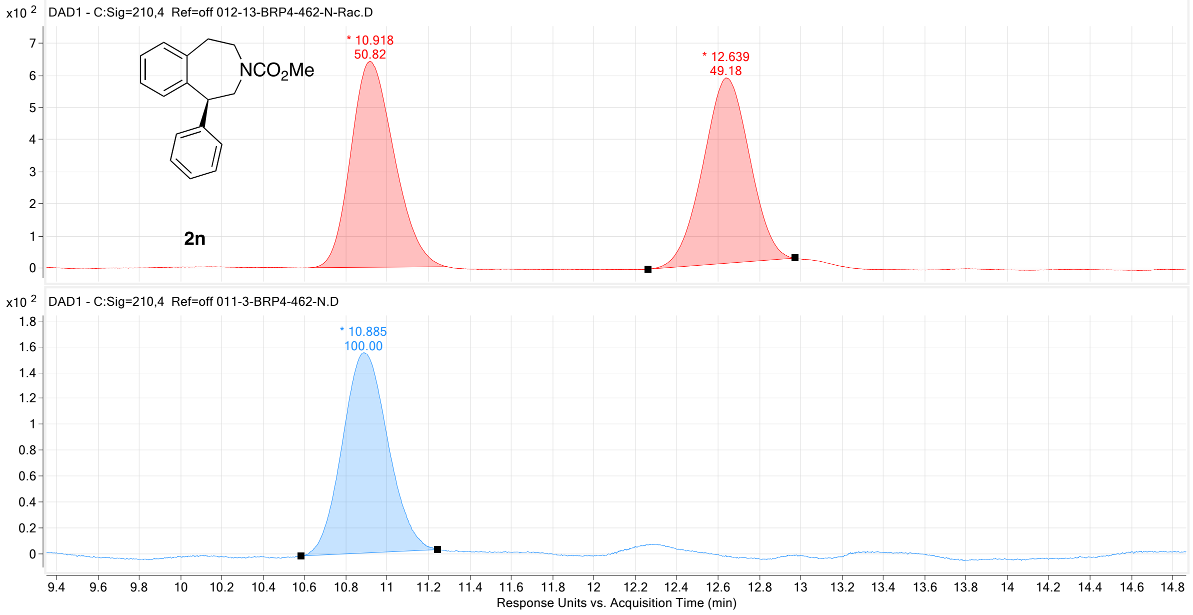
x10 2
DAD1 - C:Sig=210,4 Ref=off 012-13-BRP4-462-N-Rac.D
NCO2Me
2n
* 10.918
50.82
* 12.639
49.18
x10 2
DAD1 - C:Sig=210,4 Ref=off 011-3-BRP4-462-N.D
* 10.885
100.00
Response Units vs. Acquisition Time (min)

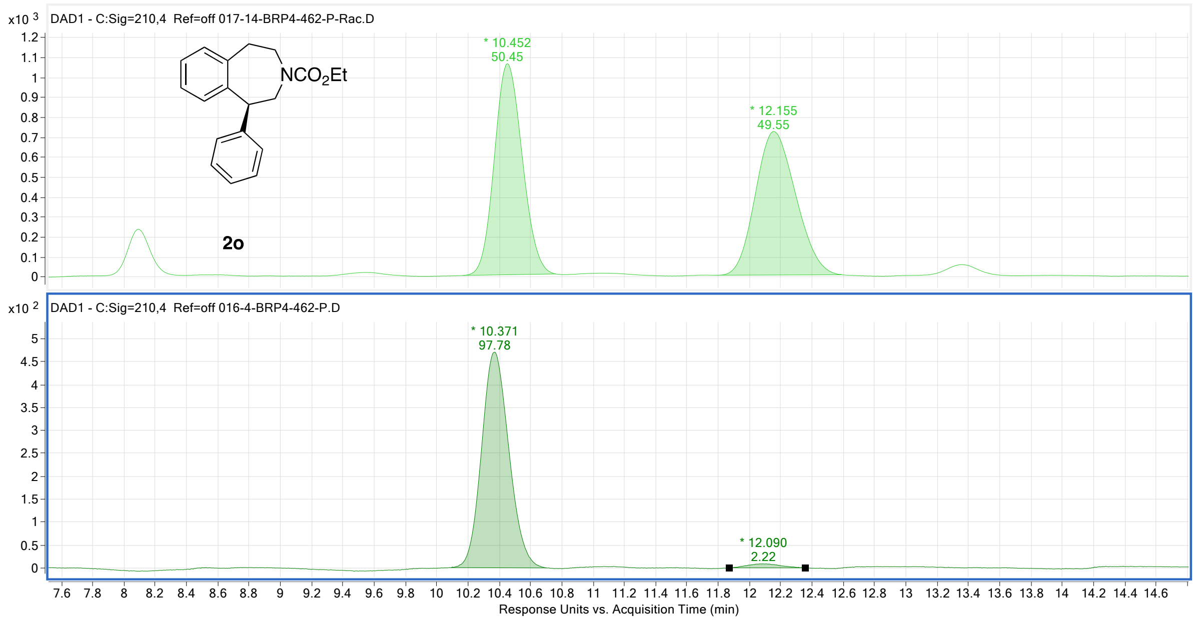
DAD1 - C:Sig=210,4 Ref=off 017-14-BRP4-462-P-Rac.D
x10 3
* 10.452
50.45
* 12.155
49.55
NCO2Et
2o
DAD1 - C:Sig=210,4 Ref=off 016-4-BRP4-462-P.D
x10 2
* 10.371
97.78
* 12.090
2.22
Response Units vs. Acquisition Time (min)

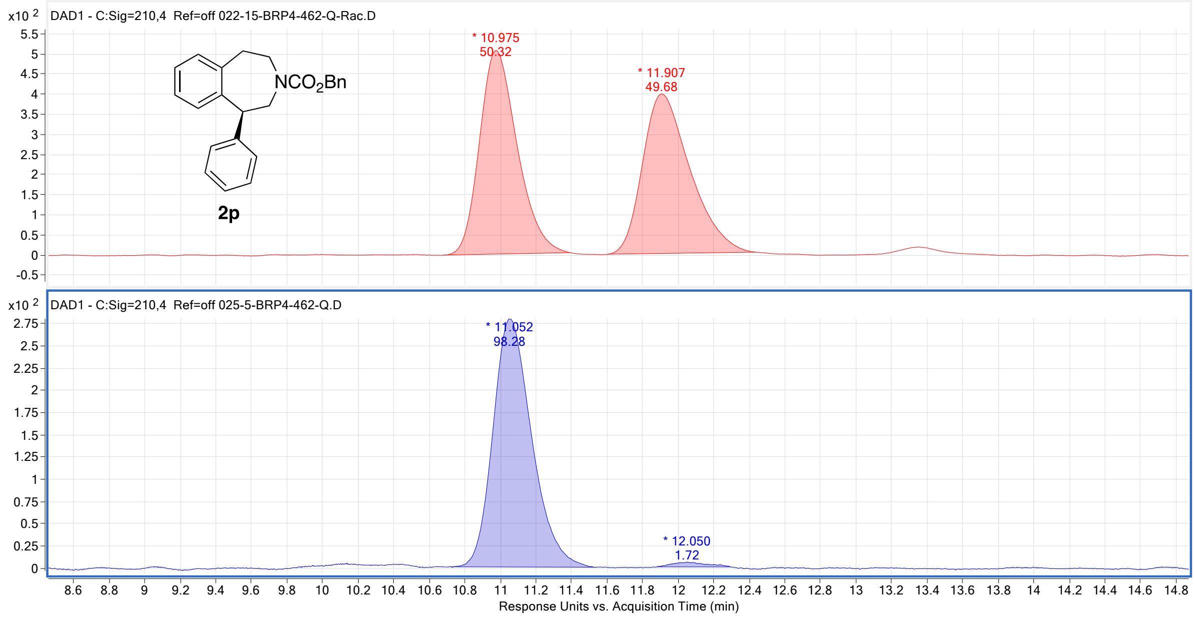
DAD1 - C:Sig=210,4 Ref=off 022-15-BRP4-462-Q-Rac.D
x10 2
* 10.975
50.32
* 11.907
49.68
NCO2Bn
2p
DAD1 - C:Sig=210,4 Ref=off 025-5-BRP4-462-Q.D
x10 2
* 11.052
98.28
* 12.050
1.72
Response Units vs. Acquisition Time (min)

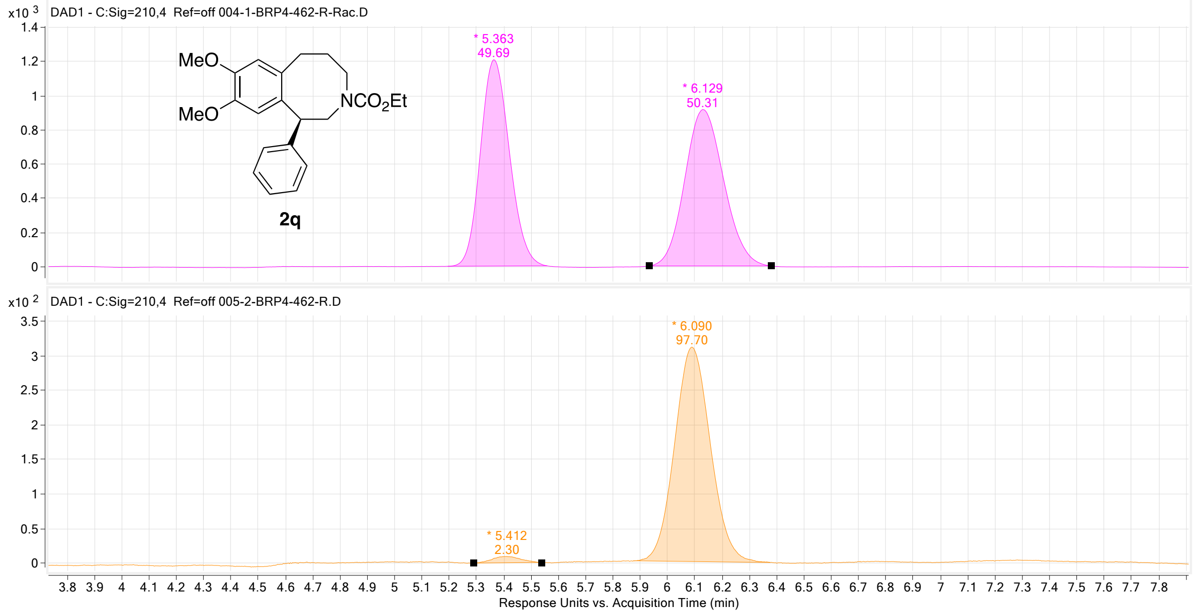
DAD1 - C:Sig=210,4 Ref=off 004-1-BRP4-462-R-Rac.D
x10 3
* 5.363
49.69
* 6.129
50.31
MeO
MeO
NCO2Et
2q
DAD1 - C:Sig=210,4 Ref=off 005-2-BRP4-462-R.D
x10 2
* 6.090
97.70
* 5.412
2.30
Response Units vs. Acquisition Time (min)

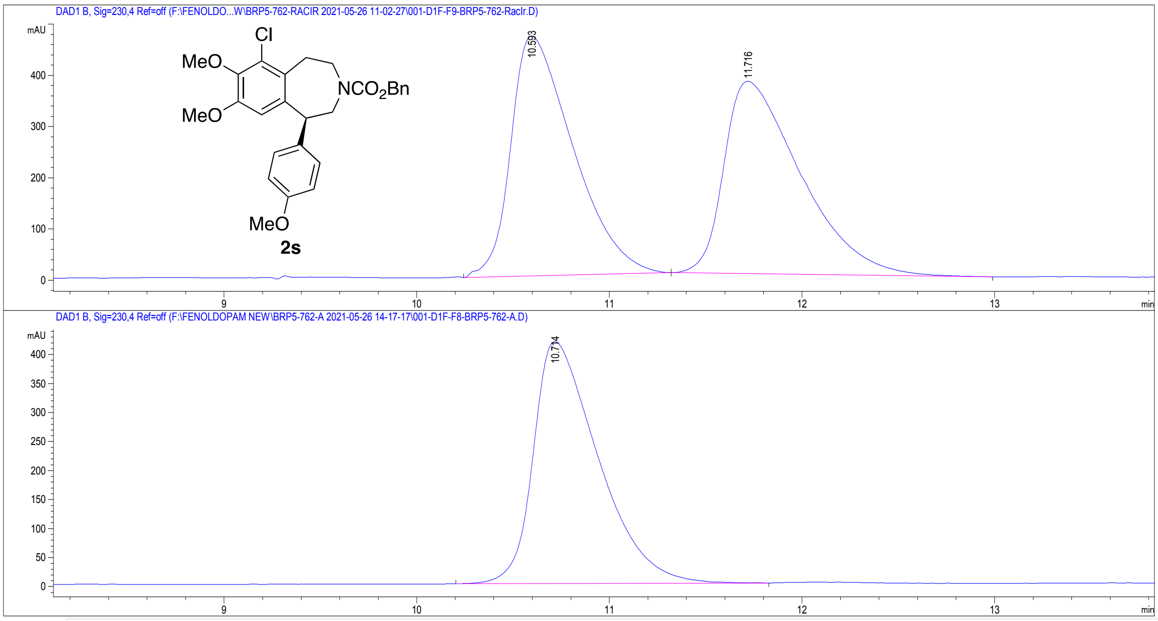
DAD1 B, Sig=230,4 Ref=off (F:\FENOLDO...W\BRP5-762-RACIR 2021-05-26 11-02-27\001-D1F-F9-BRP5-762-RacIr.D)
mAU
10.593
11.716
2s
DAD1 B, Sig=230,4 Ref=off (F:\FENOLDOPAM NEW\BRP5-762-A 2021-05-26 14-17-17\001-D1F-F8-BRP5-762-A.D)
mAU
10.714
min

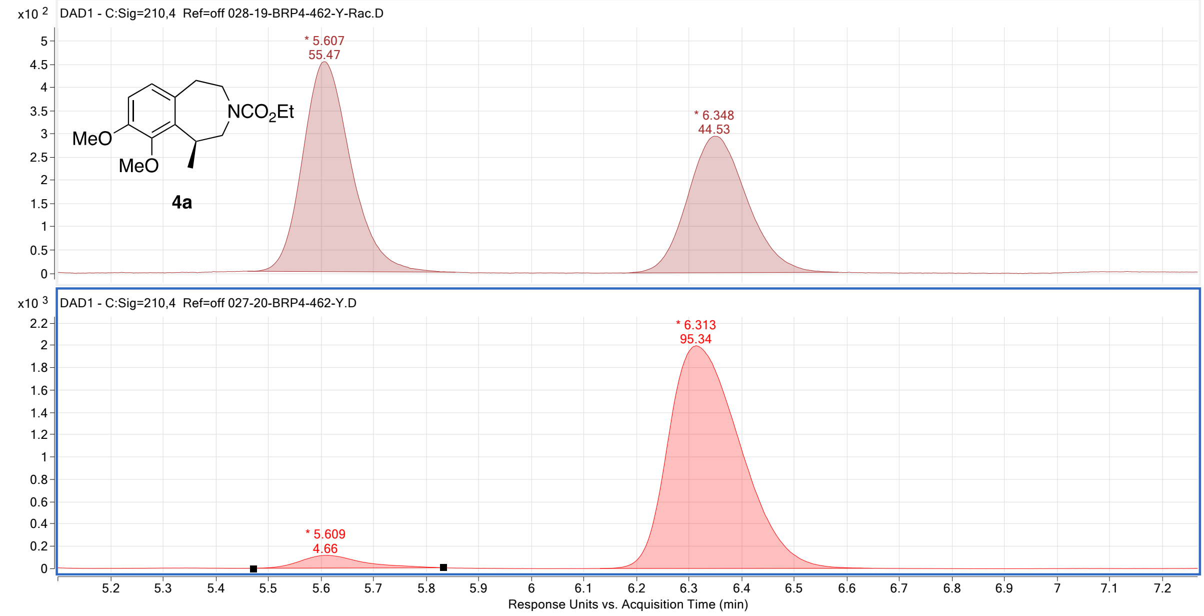
x10 2 DAD1 - C:Sig=210,4 Ref=off 028-19-BRP4-462-Y-Rac.D
* 5.607
55.47
* 6.348
44.53
4a
x10 3 DAD1 - C:Sig=210,4 Ref=off 027-20-BRP4-462-Y.D
* 6.313
95.34
* 5.609
4.66
Response Units vs. Acquisition Time (min)

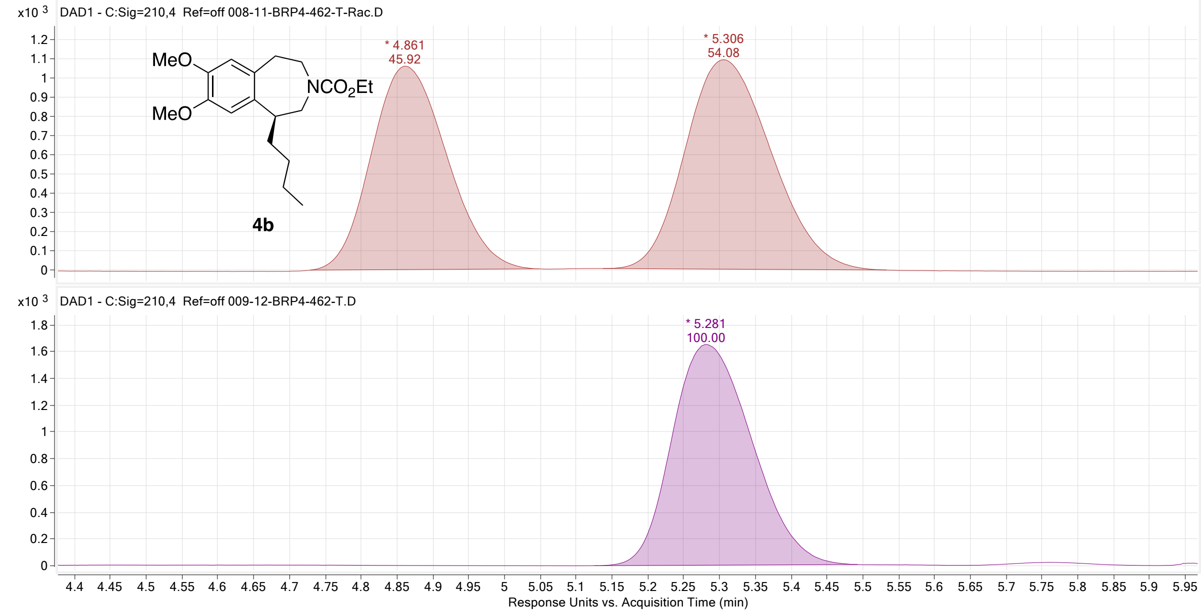
x10 3 DAD1 - C:Sig=210,4 Ref=off 008-11-BRP4-462-T-Rac.D
* 4.861
45.92
* 5.306
54.08
4b
x10 3 DAD1 - C:Sig=210,4 Ref=off 009-12-BRP4-462-T.D
* 5.281
100.00
Response Units vs. Acquisition Time (min)

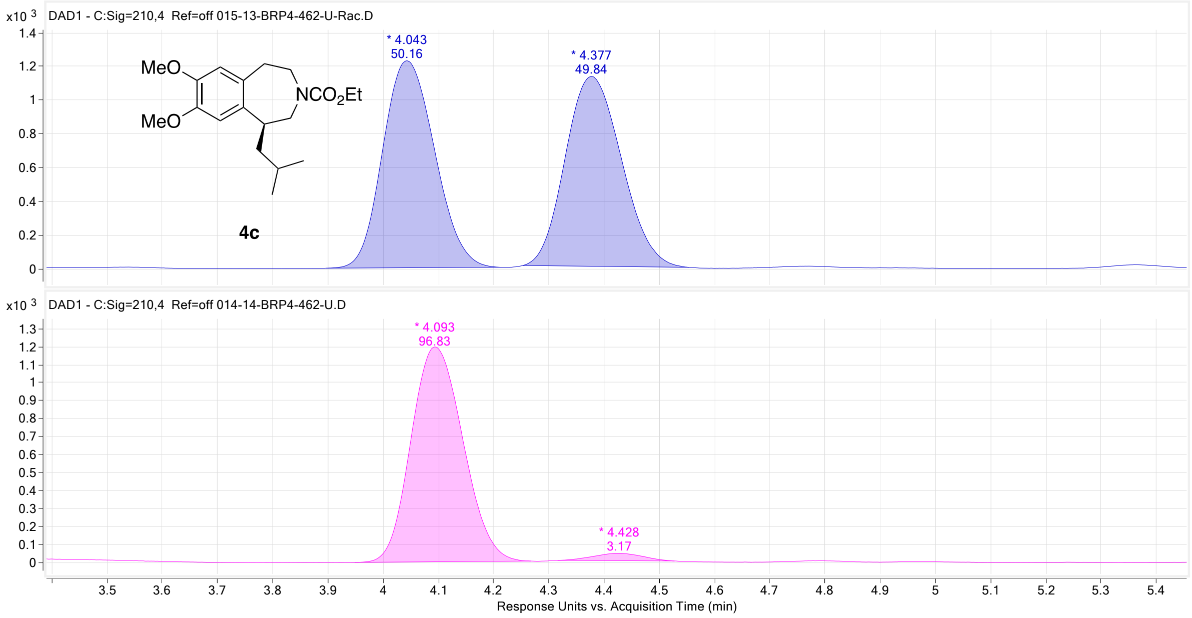
x10 3 DAD1 - C:Sig=210,4 Ref=off 015-13-BRP4-462-U-Rac.D
MeO
MeO
NCO2Et
4c
* 4.043
50.16
* 4.377
49.84
x10 3 DAD1 - C:Sig=210,4 Ref=off 014-14-BRP4-462-U.D
* 4.093
96.83
* 4.428
3.17
Response Units vs. Acquisition Time (min)

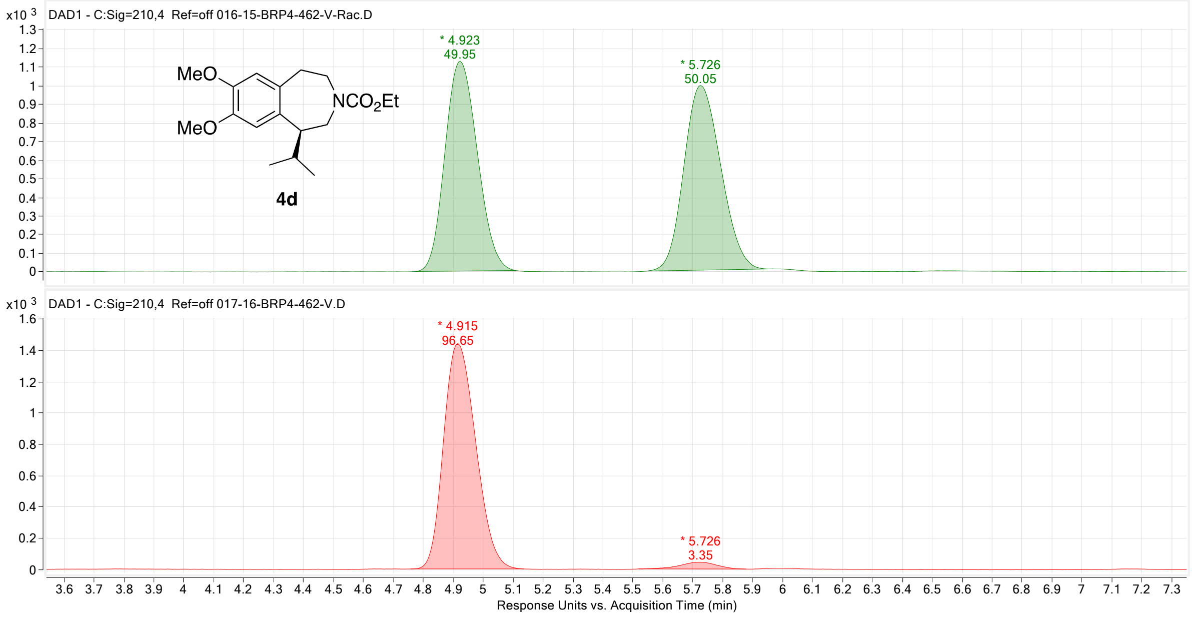
x10 3 DAD1 - C:Sig=210,4 Ref=off 016-15-BRP4-462-V-Rac.D
MeO
MeO
NCO2Et
4d
* 4.923
49.95
* 5.726
50.05
x10 3 DAD1 - C:Sig=210,4 Ref=off 017-16-BRP4-462-V.D
* 4.915
96.65
* 5.726
3.35
Response Units vs. Acquisition Time (min)

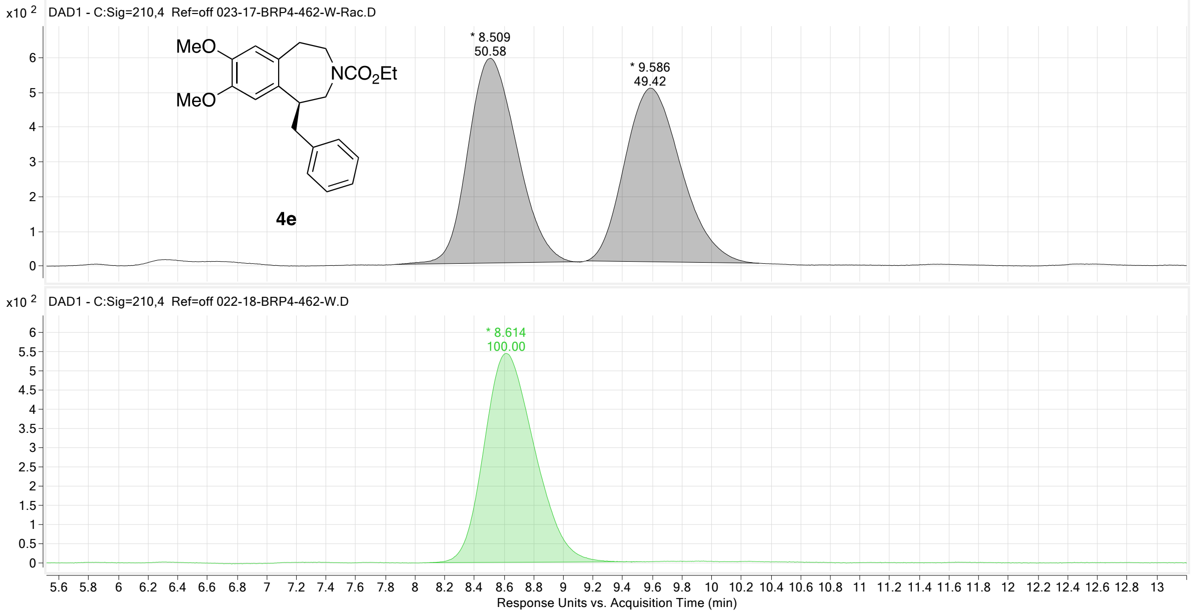
x10 2 DAD1 - C:Sig=210,4 Ref=off 023-17-BRP4-462-W-Rac.D
MeO
MeO
NCO2Et
4e
* 8.509
50.58
* 9.586
49.42
x10 2 DAD1 - C:Sig=210,4 Ref=off 022-18-BRP4-462-W.D
* 8.614
100.00
Response Units vs. Acquisition Time (min)

References

1) Ieawsuwan, W.; Pingaew, R.; Kunkaewom, S.; Ploypradith, P.; Ruchirawat, S. *Asian J. Org. Chem.* **2019**, *8*, 1441-1447.
2) Chang, M.-Y.; Wu, M.-H. *Tetrahedron Lett.*, **2012**, *53*, 3173-3177.
3) Chang, M.-Y.; Chan, C.-K.; Lin, S.-Y.; Hsu, R.-T. *Tetrahedron*, **2012**, *68*, 10272-10279.
4) Niyomchon, S.; Audisio, D.; Luparia, M.; Maulide, N. *Org. Lett.*, **2013**, *15*, 2318-2321.
